# Metal-free direct alkylation of unfunctionalized allylic/benzylic sp^3^ C–H bonds *via* photoredox induced radical cation deprotonation†
†Electronic supplementary information (ESI) available: Experimental procedures, characterization data and NMR spectra of all new compounds; ORTEP drawing of compound **41** (major diastereomer). CCDC 1506724. For ESI and crystallographic data in CIF or other electronic format see DOI: 10.1039/c7sc00953d



**DOI:** 10.1039/c7sc00953d

**Published:** 2017-04-28

**Authors:** Rong Zhou, Haiwang Liu, Hairong Tao, Xingjian Yu, Jie Wu

**Affiliations:** a Department of Chemistry , National University of Singapore , 3 Science Drive 3 , 117543 , Republic of Singapore . Email: chmjie@nus.edu.sg; b College of Chemistry and Chemical Engineering , Taiyuan University of Technology , Taiyuan , 030024 , China; c College of Chemistry , Beijing Normal University , Beijing , China

## Abstract

Despite notable recent efforts, a catalytic and convenient strategy for the direct alkylation of unactivated allylic or benzylic sp^3^ C–H bonds remains a formidable challenge facing the synthesis community. We herein report an unprecedented allylic/benzylic alkylation using only an organo-photoredox catalyst, which enables coupling of a broad scope of alkenes/arenes and electron-deficient alkenes in an atom- and redox-economic manner. A photoredox induced alkene/arene radical cation deprotonation is proposed to smoothly generate the key allylic and benzylic radical intermediates. It represents the first C–C bond formation *via* radical cation deprotonation under visible light conditions. The resulting products can be easily scaled up and directly converted to γ,δ-unsaturated or α,β-diaryl-acids, -esters, -amides, -pyrazoles, -isoxazoles, as well as lactones, which enables this mild and selective sp^3^ C–H alkylation to rapidly access complex bioactive molecules.

## Abstract 2

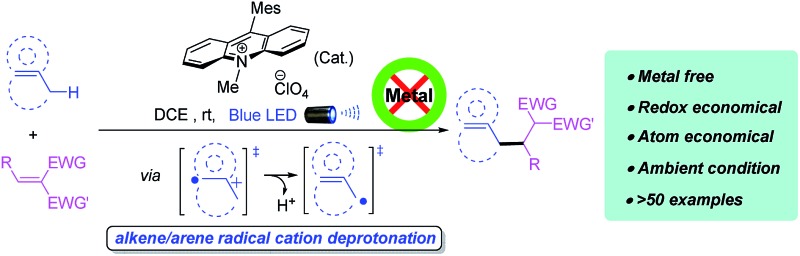
An unprecendented direct alkylation of unfunctionalized allylic/benzylic sp^3^ C–H bonds *via* photoredox induced radical cation deprotonation is disclosed.

## Introduction

**Scheme 1:**
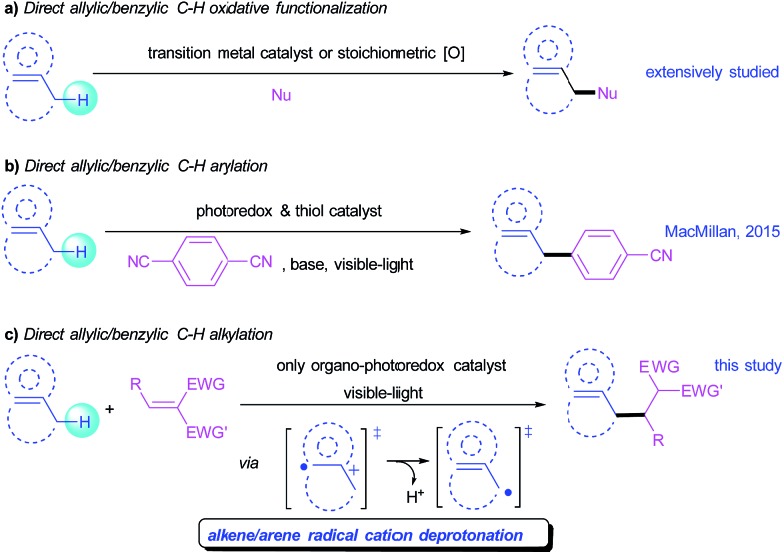
Direct functionalization of allylic/benzylic sp^3^ C–H bonds.

Direct C–C bond formation from non-activated C–H bonds has profoundly affected organic synthesis, maximizing atom and step economy and enabling novel disconnections in the retrosynthesis of carbon skeletons. Although substantial progress has been achieved in this research field, the development of general and mild strategies for the engagement of sp^3^ C–H bonds in C–C bond forming reactions has proved difficult. In this context, allylic and benzylic C–H activations have attracted a special interest because these C–H bonds can be activated relatively more easily than the other sp^3^ C–H bonds. Even though protocols for oxidative functionalization of allylic and benzylic C–H bonds have been intensively studied (Scheme 1a),^1^ the engagement of these substrates in C–C bond formation is still limited, which normally involves a nucleophilic or carbenoid counterpart under transition-metal catalysis.^2^ An orthogonal approach for C–C bond formation of these sp^3^ C–H bonds is the reaction of allylic/benzylic radicals generated from homolytic hydrogen abstraction with electrophilic Michael acceptors.^3^ However, reported examples were limited to benzylic alkylations,^4^ and suffered from narrow substrate scopes, harsh conditions, requiring excess radical initiators, or use of harmful and equipment-demanding UV light.^3^ Nonetheless, a metal-free, mild and broadly applicable strategy for both allylic and benzylic alkylation remains underdeveloped but is highly desirable.

Visible-light-mediated photoredox catalysis has attracted increasing attention over the past decade because of its unique reactivity patterns and high efficiency, enabling many previously unattainable transformations *via* single-electron transfer (SET) process.^5^ A series of new C–C bond forming reactions from native C–H bonds have been developed using this novel activation mode. In particular, the MacMillan group recently reported a direct arylation of allylic/benzylic sp^3^ C–H bonds through the combination of a photoredox and a thiol organic catalysis that promotes a hydrogen atom transfer (HAT) to access allylic/benzylic radicals (Scheme 1b).^6^

Among visible-light-mediated photoredox reactions, radical cation species effectively generated through reductive quenching of the excited photocatalyst *via* an SET process, have frequently been involved to achieve important transformations. For example, alkene radical cations have been successfully applied to [2 + 2],^7^ [4 + 2] cycloadditions,^8^ and anti-Markovnikov alkene hydrofunctionalization.^9^ Moreover, arene radical cations have been accessed for amination without the need for prefunctionalization of the aromatic component, as described by the Nicewicz group.^10^ Deprotonation is a typical reaction pathway for π-C–H^+^˙ type radical cations, because the acidity is inherently augmented.^11^ This process is appealing for the convenient generation of allylic or benzylic radicals. However, useful synthetic transformations based on deprotonation of radical cations generated by photo-induced SET are scarce. This scarcity is mainly a consequence of the inefficient deprotonation that competes unfavorably with the radical ion pair induced back electron transfer.^12^ Other challenges for a practical radical cation deprotonation process include side-reactions such as dimerization or further oxidation of the generated radicals.^11,12^

## Results and discussion

### Designing strategy

Considering the ease of accessing π-C–H^+^˙ type radical cations under photoredox condition, we envisioned that an alkene/arene radical cation generated *via* the visible-light induced SET might accomplish a smooth deprotonation to form an allylic/benzylic radical, which would subsequently undergo C–C bond formations with Michael acceptors, thereby achieving direct alkylation of allylic/benzylic sp^3^ C–H bonds. However, to realize such allylic/benzylic radical formation, the photo-catalyst should be capable of oxidizing a wide range of alkenes and arenes, can minimize the unproductive back electron transfer, and does not interact with the generated allylic/benzylic radical.

**Fig. 1:**
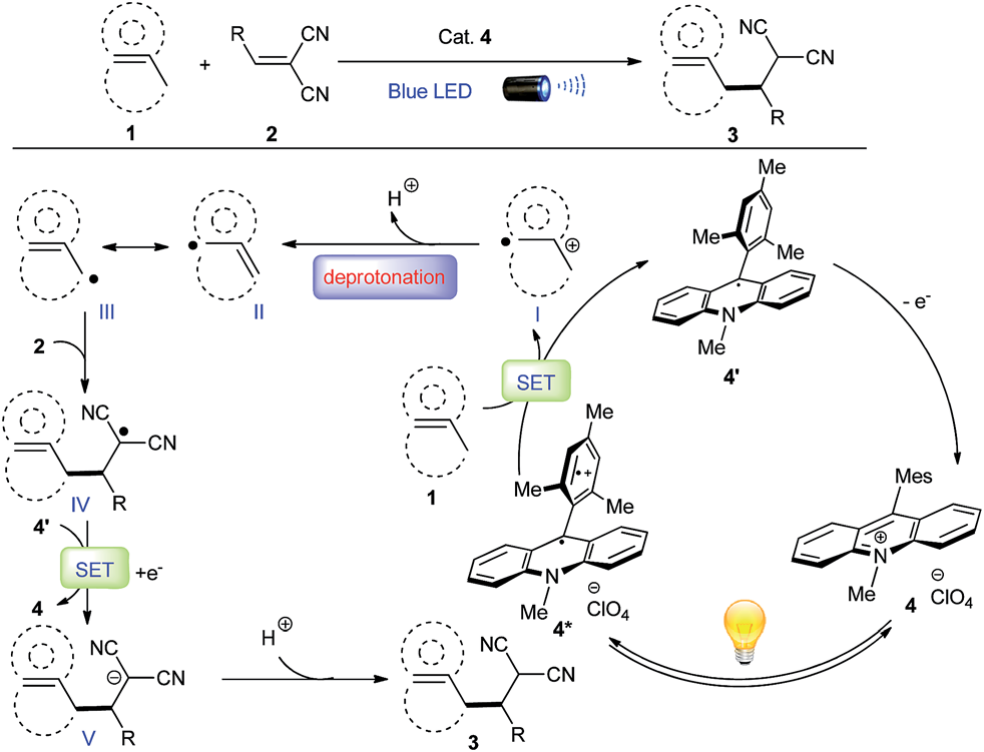
Proposed radical cation deprotonation for alkylation of allylic/benzylic C–H bonds.

Recent reports indicate organo-photoredox catalyst 9-mesityl-10-methylacridinium perchlorate [Acr^+^-Mes]ClO_4_ (**4**) may be a good photooxidant that fulfils all of the aforementioned requirements. Both alkenes and arenes can be conveniently oxidized to radical cation **I** by 4* because of its high excited state oxidizing power (
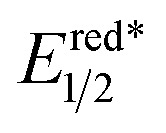
 = +2.06 V *vs.* SCE in MeCN) (Fig. 1).^9,10^ The reduced form of the acridinium catalyst **4′** would not be actively involved in the unproductive back electron transfer or interact with radical cation **I** because it exists in the neutral radical form with a sterically bulky substituent.^9*b*^ Both Fukuzumi and Lei's studies have demonstrated that benzyl radical cations generated by this catalyst gave the corresponding benzyl radicals *via* deprotonation, which were subsequently oxidized by O_2_ to deliver aldehydes and ketones.^13^ However, C–C bond formation has never been achieved *via* this mild radical generation pathway. We proposed that radical cation **I** would favor deprotonation to deliver radical **II**, which is in equilibrium with radical **III**. Nucleophilic addition of the less-hindered allylic/benzylic radical **III** to Michael acceptors such as methylene-malononitriles **2** would furnish alkyl radical **IV**. Single-electron reduction of this electron-deficient radical **IV** by the reduced form of the acridinium catalyst **4′** (*E*ox1/2 = –0.57 V *vs.* SCE in MeCN) would deliver alkylation product **3** after protonation, while regenerating photocatalyst **4**.

### Optimization of reaction conditions

We initially attempted the proposed alkylation protocol by exposing tetramethylethylene (**5**) and benzylidenemalononitrile (**6**) to blue LED light (*λ*_max_ = 425 nm) in the presence of **4** (5 mol%) in DCE (0.1 M) for 18 h at ambient temperature (Table 1, entry 1), which afforded alkylation product **7** in 72% isolated yield, with no detection of [2 + 2] cycloaddition byproducts. An excess amount of tetramethylethylene (4 equiv.) was required to achieve a full conversion of the Michael acceptor. Examination of a range of photocatalysts and solvents revealed that **4** and DCE was the most effective combination (entries 2–7). Remarkably, only the strong photo-oxidants **4** and 2,4,6-tri(*p*-tolyl)pyrylium tetrafluoroborate gave alkylation product **7** in good yields (entries 1 and 2), as no reaction occurred with weaker oxidants (entries 3 and 4); this result is consistent with our hypothesis because tetramethylethylene **5** (*E*ox1/2 = +1.22 V *vs.* Ag/AgNO_3_ in MeCN)^14^ requires a relatively strong oxidant for the generation of its radical cation. A higher yield (91%) was obtained when the reaction concentration was diluted to 0.05 M and the catalyst loading was reduced to 2.5 mol% (entry 8). When this visible-light-mediated reaction was conducted in our recently developed “stop-flow” micro-tubing (SFMT) reactors instead of in batch flasks,^15^ the required reaction time was substantially reduced from 18 h to 5 h and a similar yield was obtained (entry 9). This result underscores the remarkable light-irradiation efficiency achieved through the use of the SFMT reactors. Finally, no product formation was detected in the absence of either photocatalysts or light, demonstrating the need for all of these components (Table S1, ESI†).

**Table 1:** Optimization for allylic alkylation

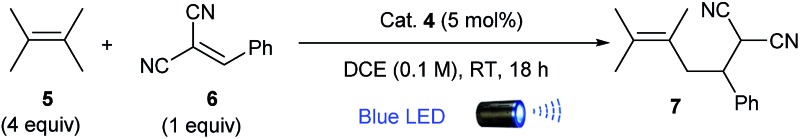		
Entry	Modifications	Yield^*a*^ (%)
1	None	72
2	2,4,6-Tri(*p*-tolyl)pyrylium tetrafluoroborate instead of **4**	70
3	Ir(ppy)_2_(dtbbpy)PF_6_, Ir(ppy)_3_, Eosin Y instead of **4**	na
4	Ru(bpy)_3_Cl_2_ instead of **4**	Trace
5	DCM instead of DCE	54
6	Acetone instead of DCE	21
7	MeCN, DMSO, DMF instead of DCE	Trace
8	0.05 M concentration, 2.5 mol% **4**	91
9	0.05 M concentration, 2.5 mol% **4**, SFMT reactor, 5 h	90

^*a*^Isolated yield; na = no reaction.

### Scope of Michael acceptors for allylic alkylation

**Scheme 2:**
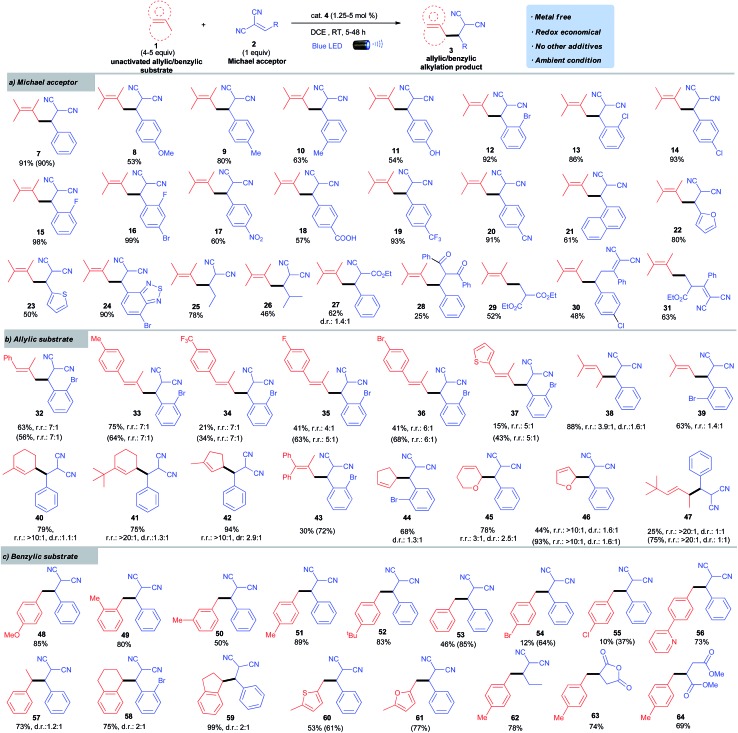
Run in a 0.2 mmol scale with respect to the Michael acceptor. Isolated yields. Regio- and diastereoselectivity were based on ^1^H NMR analysis. Regioselectivity shown as the major isomer *vs.* sum of minor isomers. Diastereoselectivity only shown for the major regioisomer. Results shown in parentheses were obtained for reactions in SFMT reactors.

Adopting the optimized batch conditions described in entry 8 (Table 1), we sought to determine the generality of the allylic alkylation by evaluating various methylene-malononitriles (Scheme 2a). “Stop-flow” conditions (Table 1, entry 9) were applied in cases where conversions were low in batch reactors. Experiments probing the scope of methylene-malononitriles revealed that a variety of electron-rich (**8** to **11**) and electron-deficient arenes (**12** to **20**) on the Michael acceptor were well tolerated, even with functional groups such as phenols (**11**), aryl halides (**12** to **16**), and acids (**18**). Sterically demanding arenes (**21**) and heterocycles such as furan (**22**), thiophene (**23**), and benzothiadiazole (**24**) were readily accommodated. Substrates with alkyl instead of aryl substituents on the methylene-malononitrile participated in this coupling reaction smoothly (**25** and **26**). The change of one nitrile group in malononitrile moiety to ester was feasible (**27**). However, other Michael acceptors such as an unsaturated diketone afforded only sluggish reactivity (**28**). Interestingly, the alkylation using the Michael acceptor with a terminal alkene moiety went smoothly (**29**). Notably, diene Michael acceptors can be successfully applied to this transformation to give 1,6-addition type products (**30** and **31**).

### Scope of allylic compounds

We next examined the scope of allylic substrates for this convenient alkylation reaction. As shown in Scheme 2b, a wide range of tri-substituted alkenes could be used in this photoredox process because they were sufficiently electron-rich to be oxidized by **4** (**32** to **42**). Both acyclic and cyclic olefins were readily accommodated. A series of 2-methyl arylpropenes provided high alkylation efficiency while exhibiting good regioselectivity (**32** to **37**). The use of 2-methyl-2-pentene led predominantly to the formation of the branched alkylation product (**38**) with moderate selectivity. The regioselectivity with 2-methyl-2-butene (**39**) was low compared to that achieved with 2-methyl-2-pentene (**38**). Remarkably, cyclic substrates bearing alkyl substituents served as excellent allylic coupling partners with excellent regioselectivity favoring the less hindered cyclic methylene (**40** to **42**).^16^ Tetra-substituted alkenes with unhindered allylic C–H bonds could be oxidized effectively using the SFMT reactors under visible light followed by C–C bond formation (**43**). In batch reactors, the reaction was slow, most likely due to the increased steric hindrance. Disubstituted olefins, including cyclic (**44** to **46**) and acyclic alkenes (**47**), can also participate in the photoredox alkylation. Notably, despite the high oxidation potential of cyclopentene (+2.32 V *vs.* SCE in MeCN),^17^ it afforded product **44** in a good yield. Overall, the observed regioselectivity was attributed to both electronic and steric effects. The most stable allylic radical would be preferentially formed through deprotonation of the selectively generated radical cation intermediate, which subsequently underwent alkylation with its least hindered resonance. To the best of our knowledge, this study represents the first example of effective alkylation of allylic sp^3^ C–H bonds with electron-deficient alkenes.^4^

### Scope of benzylic compounds

This alkylation protocol was further extended to benzylic substrates (Scheme 2c). Toluene moieties with electron-donating substituents were good candidates for the alkylation (**48** to **52**). Electron-neutral and electron-poor arenes reacted much slower than electron-rich arenes because of their increased oxidative potential (*e.g. E*ox1/2toluene = +2.36 V *vs.* SCE in MeCN).^17^ However, the use of the SFMT reactor could accelerate the reaction and give satisfactory conversions (**53** to **55**). Notably, 2-(*p*-tolyl)pyridine uneventfully afforded its alkylation product in good yield (**56**). In addition to toluenes, ethylbenzene, tetralin, and indane were all suitable candidates and the reactions proceeded smoothly to give alkylation products in good to excellent yields (**57** to **59**). Methyl-substituted heterocycles such as thiophenes and furans were alkylated in good yields with the assistance of the SFMT reactor (**60**, **61**). Incorporation of an alkyl substituent instead of the aryl substituent in methylene-malononitriles, or changing of the Michael acceptor to maleic anhydride and dimethyl maleate, was also feasible (**62** to **64**). This photoredox induced allylic/benzylic alkylation therefore represents a metal-free, atom-, and redox-economical C–H functionalization protocol.

### Synthetic utilities of alkylation products

**Scheme 3:**
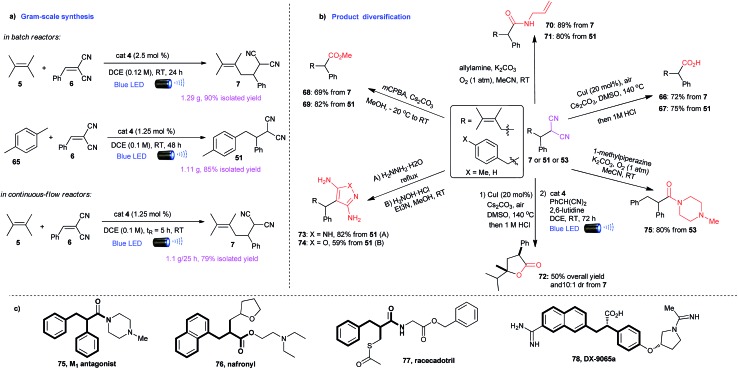
Synthetic applications of alkylation products.

To further demonstrate the synthetic utility of this methodology, the alkylations were amenable to scale-up to gram quantities in both batch reactors and continuous-flow reactors (Scheme 3a). The resulted alkylation products can be directly transformed to γ,δ-unsaturated or α,β-diaryl acids (**66**, **67**),^18^ esters (**68**, **69**),^19^ amides (**70**, **71**),^20^ and lactones (**72**) in a convenient manner (Scheme 3b). These building blocks are core structures for a variety of drug molecules, such as nafronyl (**76**),^21^ racecadotril (**77**),^22^ and DX-9065a (**78**)^23^ (Scheme 3c), enabling a convenient way to synthesize these pharmaceutical compounds and their derivatives. The opportunities to effect synthetic streamline with this general C–H reactivity are further illustrated in a facile synthesis of **75** (Scheme 3b), a representative compound of a group of M_1_ antagonists.^24^ In addition, the malononitrile moieties can be directly converted to diaminopyrazoles (**73**), that are important intermediates for preparing therapeutically interesting pyrazolo[1,5-α]pyrimidines,^25^ and aminoisoxazoles (**74**) with potential hypoglycemic activity.^26^

### Control experiments to support the proposed mechanism

Several control experiments were performed to gain further insights into the reaction pathway (for details, see ESI†). First, no alkylation products were observed when the reaction was performed in the presence of radical scavengers such as TEMPO and hydroquinone, suggesting a radical process was involved. Furthermore, as indicated by the luminescence quenching experiments, both alkene **5** and 4-methylanisole can quench the excited state of acridinium (4*), whereas methylene-malononitrile **6** cannot; this result is in agreement with our proposed mechanism (Fig. 1). Several deuterium-labeling experiments were investigated, revealing that the anion **V** would most likely abstract a proton from the solvent rather than from substrate **1**. The intermolecular competition experiment established that the magnitude of the kinetic isotopic effect (KIE) given by *k*_H_/*k*_D_ was 3.3, indicating that the deprotonation might be involved in the rate-limiting step. We also questioned whether the alkene/arene radical cation **I** generated *via* photoredox mediated SET process could accomplish an intermolecular H-atom abstraction from another molecule of **1**; in this case, the radical cation itself served as a HAT catalyst.^27^ However, the quantum chemistry calculation using a composite CBS-QB3 method, did not support this process (Schemes S1 and S2, ESI†).

## Conclusions

Overall, we have developed a visible-light-mediated alkylation of unactivated allylic/benzylic sp^3^ C–H bonds, which represents one of the most green and sustainable protocols featuring merits such as metal- and additive-free, atom- and redox-economical, and works effectively with a wide range of substrates (>55 examples). Use of SFMT reactors effectively enhanced the efficiency of light-promoted transformations and broadened the substrate scope. The alkylation products can be conveniently converted into γ,δ-unsaturated or α,β-diaryl-acids, -esters, -amides, -pyrazoles, -isoxazoles, as well as lactones for further elaborating valuable pharmaceutical molecules. This visible-light photoredox induced radical cation deprotonation process represents a powerful strategy and will likely find more applications for selective activation of native C–H bonds as latent nucleophilic handles.
