# Supplementary material for: Metal-free direct alkylation of unfunctionalized allylic/benzylic sp3 C–H bonds via photoredox induced radical cation deprotonation
Source: Chem Sci. 2017 Apr 28;8(6):4654–9. doi: 10.1039/c7sc00953d (PMC5618257; doi:10.1039/c7sc00953d)
Supplement: Supplementary file 1 [file SC-008-C7SC00953D-s001.pdf]

Electronic Supplementary Material (ESI) for Chemical Science.
This journal is © The Royal Society of Chemistry 2017

Metal-free direct alkylation of unfunctionalized allylic/benzylic sp^3 C–H bonds via photoredox induced radical cation deprotonation

Rong Zhou,[a,b] Haiwang Liu,[a] Hairong Tao,[c] Xingjian Yu[a] and Jie Wu*[a]

[a] Department of Chemistry, National University of Singapore, 3 Science Drive 3, Republic of Singapore, 117543. E-mail: chmjie@nus.edu.sg

[b] College of Chemistry and Chemical Engineering, Taiyuan University of Technology, Taiyuan, China, 030024.

[c] College of Chemistry, Beijing Normal University, Beijing, China.

Table of Contents

I. General Information S2

II. Survey of Reaction Conditions S2

III. Typical Procedure for Alkylation of Allylic sp^3 C–H Bonds in Batch S3

IV. Typical Procedure for Alkylation of Allylic sp^3 C–H Bonds in "Stop-Flow Micro Tubing" Reactor S4

V. Typical Procedure for Alkylation of Benzylic sp^3 C–H Bonds in Batch S5

VI. Typical Procedure for Alkylation of Benzylic sp^3 C–H Bonds in "Stop-Flow Micro Tubing" Reactor S5

VII. Large-Scale Continuous-Flow Synthesis of Compound **7** S6

VIII. Control Experiments for Mechanistic Investigation S6

IX. Proposed Mechanism Involving Hydrogen Atom Transfer (HAT) Process and the DFT Calculation Results S12

X. Analytical Data of the Products S16

XI. Supplementary References S49

XII. ORTEP Drawings for the Major Diastereomer of **41** S50

XIII. ^{1}H, ^{13}C NMR Spectra of Products S52

XIV. NOESY, HMQC, and HMBC Spectra of Representative Compounds S194

I. General Information

Chemicals and solvents were purchased from commercial suppliers and used as received. ^{1}H NMR, ^{13}C NMR spectra were recorded on a Bruker ACF300 (300 MHz), AV-III400 (400 MHZ) or AMX500 (500 MHz) spectrometer. Chemical shifts were calibrated using residual undeuterated solvent as an internal reference ($CDCl_3$: 7.26 ppm ^{1}H NMR, 77.0 ppm ^{13}C NMR). Multiplicity was indicated as follows: s (singlet), d (doublet), t (triplet), q (quartet), m (multiplet), dd (doublet of doublet), br s (broad singlet). All high resolution mass spectra (HRMS) were obtained on a Finnigan/MAT 95XL-T spectrometer. All GC analysis was performed on Aglilent 7820A&5977E GC-MS. X-ray crystal diffraction data were collected on a Bruker D8 Venture Single Crystal X-ray diffractometer. The Blue LED strips (2 meter, 20 W) were purchased from Inwares Pte Ltd (Singapore). The Asia Syringe Pump was purchased from Syrris Company (UK) for continuous flow setup. The Tefzel shut-off valves, and HPFA micro tubings were purchased from IDEX Health & Science (Oak Harbor, WA). Further visualization was achieved by staining with iodine.

II. Survey of Reaction Conditions

Table S1. Survey of conditions for model reaction[a]

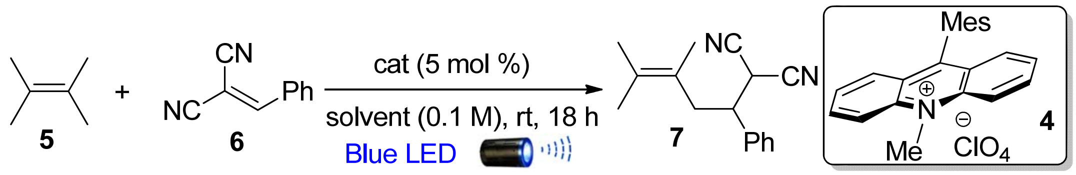

entry	catalyst	solvent	yield[b] (%)
1	**4**	DCE	72
2	2,4,6-tri(*p*-tolyl)pyrylium tetrafluoroborate	DCE	70
3	$Ir(ppy)_2(dtbpy)_3PF_6$	DCE	na
4	$Ir(ppy)_3$	DCE	na
5	Eosin Y	DCE	na
6	$Ru(bpy_{)3}Cl_2$	DCE	trace
7	$Ru(bpz)_3(PF_6)_2$	DCE	trace
8	**4**	DCM	54
9	**4**	acetone	21

10	**4**	MeCN	trace
11	**4**	DMSO	trace
12	**4**	DMF	trace
13[c]	**4**	DCE	84
14[c,d]	**4**	DCE	91
15[c,d,e]	**4**	DCE	90
16	no catalyst	DCE	na
17[f]	**4**	DCE	na

[a] Unless otherwise noted, reaction of **5** (95 µL, 0.8 mmol), **6** (31 mg, 0.2 mmol), and catalyst (0.01 mmol) were carried out in 2.0 mL of the solvent. [b] Isolated yields. [c] 4.0 mL DCE was used. [d] The catalyst loading was changed to 2.5 mol %. [e] The reaction was conducted using a "stop-flow micro tubing" reactor for 5 hrs. [f] The reaction was conducted in dark.

III. Typical Procedure for Alkylation of Allylic sp^3 C–H Bonds in Batch

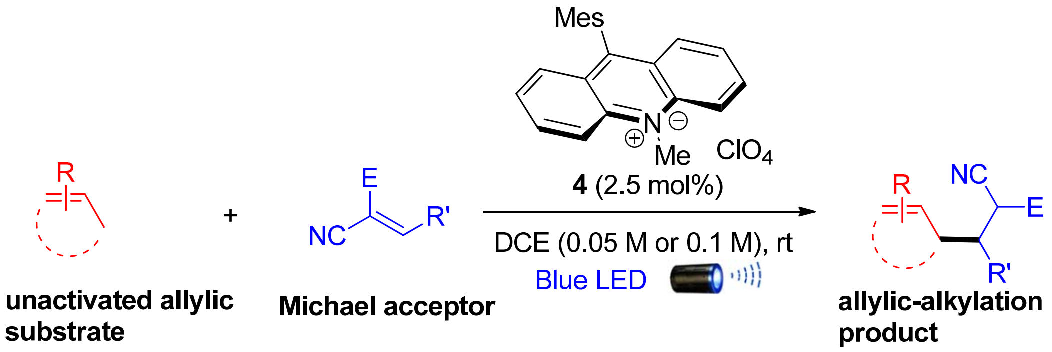

To a 25 mL Schlenk tube equipped with a magnetic stir bar was added the corresponding Michael acceptor (0.2 mmol, 1.0 equiv.), and 9-mesityl-10-methylacridinium perchlorate **4** (for **7-31**, and **38-47**, 0.005 mmol, 0.025 equiv.; for **32-37**, 0.01 mmol, 0.05 equiv.). The resulting mixture was sealed and degassed via vacuum evacuation and subsequent backfill with argon for three times. Then anhydrous DCE (for **7-31**, and **38-47**, 4.0 mL; for **32-37**, 2.0 mL) was added, and finally allylic substrate (0.8 mmol, 4.0 equiv.) was added. After that, the reaction was placed under a blue LED (2 meter strips, 20 W) with an argon balloon and irradiated for 18-48 hrs. The solvent was removed on a rotary evaporator under reduced pressure and the residue was subjected to column chromatography isolation on silica gel by gradient elution with hexane / ethyl acetate

(15:1-3:1) to give the alkylation product.

IV. Typical Procedure for Alkylation of Allylic sp^3 C–H Bonds in "Stop-Flow Micro Tubing" Reactor

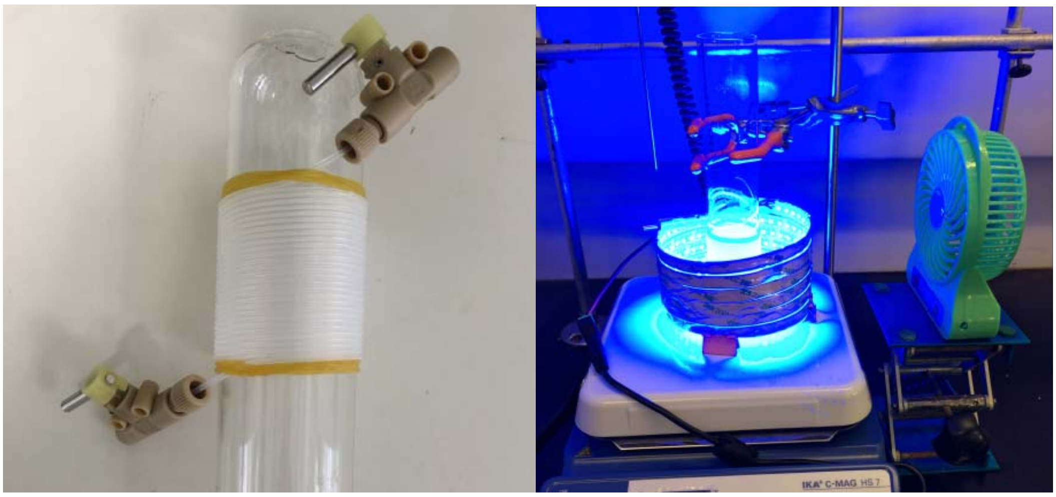

Figure S1. "Stop-flow micro tubing" reactor and reaction set up

Under Argon atomsphere, a solution of Michael acceptor (0.2 mmol), 9-mesityl-10-methylacridinium perchlorate **4** (for **32-37**, 0.01 mmol, 0.05 equiv.; for **7**, **43**, **46**, **47** 0.005 mmol, 0.025 equiv.), and allylic substrate (0.8 mmol, 4.0 equiv.) in anhydrous DCE (2.0 mL, for **7**, 4.0 mL) was introduced into a silicon septa vial. Then the solution was pumped into a stop-flow micro tubing (SFMT) reactor (total volume 1.5 mL) which was made of HPFA tubing (O.D. 1/16", I.D. 0.03", 340 cm) via a syringe. The valves were closed, and the SFMT was placed under a blue LED and irradiated for 5-48 hrs. The solvent was removed on a rotary evaporator under reduced pressure and the residue was subjected to column chromatography isolation on silica gel by gradient elution with hexane / ethyl acetate (15:1-3:1) to give the alkylation product.

V. Typical Procedure for Alkylation of Benzylic sp^3 C–H Bonds in Batch

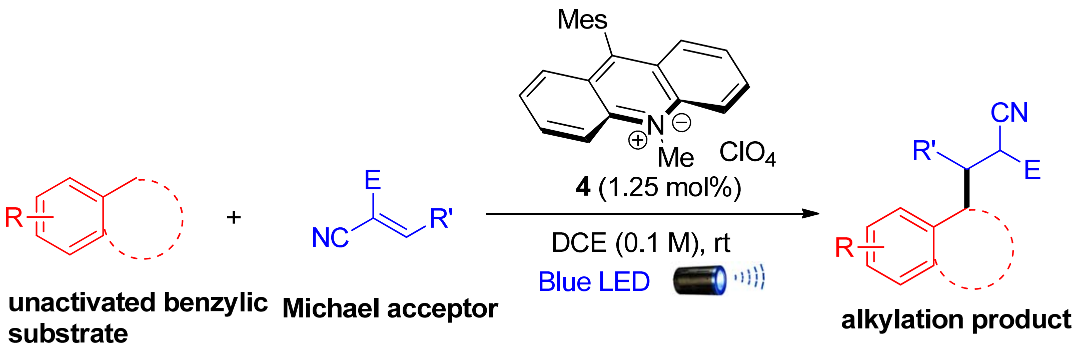

To a 25 mL Schlenk tube equipped with a magnetic stir bar was added the corresponding Michael acceptor (0.2 mmol, 1.0 equiv.), and 9-mesityl-10-methylacridinium perchlorate **4** (0.0025 mmol, 0.0125 equiv.). The resulting mixture was sealed and degassed via vacuum evacuation and subsequent backfill with argon for three times. Then anhydrous DCE (2.0 mL) was added, and finally benzylic substrate (1.0 mmol, 5.0 equiv.) was added. After that, the reaction was placed under a blue LED with an argon balloon and irradiated for 30-48 hrs. The solvent was removed on a rotary evaporator under reduced pressure and the residue was subjected to column chromatography isolation on silica gel by gradient elution with hexane / ethyl acetate (15:1-3:1) to give the alkylation product.

VI. Typical Procedure for Alkylation of Benzylic sp^3 C–H Bonds in "Stop-Flow Micro Tubing" Reactor

Under Argon atomsphere, a solution of Michael acceptor (0.2 mmol), 9-mesityl-10-methylacridinium perchlorate **4** (0.0025 mmol, 0.0125 equiv.), and benzylic substrate (1.0 mmol, 5.0 equiv.) in anhydrous DCE (2.0 mL) was introduced into a silicon septa vial. Then the solution was pumped into a stop-flow micro tubing (SFMT) reactor (total volume 1.5 mL) which was made of HPFA tubing (O.D. 1/16", I.D. 0.03", 340 cm) via a syringe. The valves were closed, and the SFMT was placed under a blue LED and irradiated for 30-48 hrs. The solvent was removed on a rotary evaporator under reduced pressure and the residue was subjected to column chromatography isolation on silica gel by gradient elution with hexane / ethyl acetate (15:1-3:1) to give the alkylation product.

VII. Large-Scale Continuous-Flow Synthesis of Compound 7

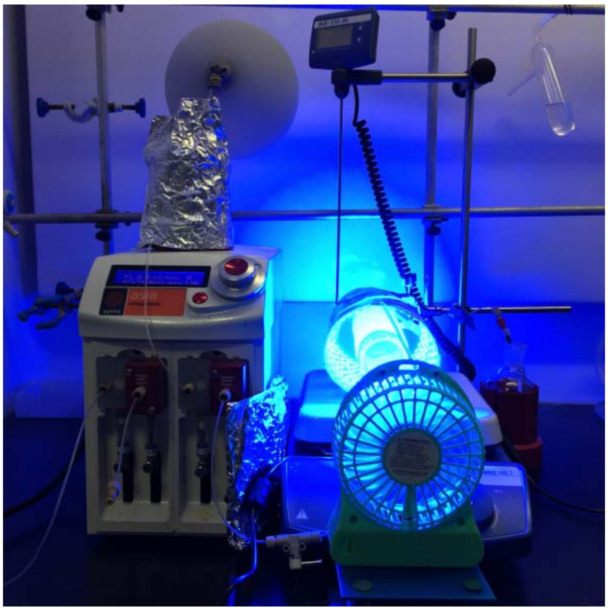

Figure S2. Continuous-flow setup for the synthesis of compound **7**

Under Argon atomsphere, a solution of 2-benzylidenemalononitrile **6** (1.23 g, 8.0 mmol), 9-mesityl-10-methylacridinium perchlorate **4** (41.1 mg, 0.075 mmol, 0.0125 equiv.), and tetramethylethylene **5** (4.0 mL, 32 mmol, 4.0 equiv.) in anhydrous DCE (80.0 mL) was introduced into a round bottom flask. As shown in Fig. S2, an Asian pump was filled with the mixture and then attached to the flow apparatus. The tubing (HPFA tubing O.D. 1/8", I.D. 0.062", 600 cm) was placed under a blue LED. The flow apparatus itself was set up with t_R = 5.0 hrs, and flow rate = 39 μL/min. After equilibration with 5 hrs, the solution was collected for 25 hrs. The solvent was removed on a rotary evaporator under reduced pressure and the residue was subjected to column chromatography isolation on silica gel by gradient elution with hexane / ethyl acetate (15:1-10:1) to give the product **7** (1.1 g) in 79% yield.

VIII. Control Experiments for Mechanistic Investigation

a) Radical inhibition experiments

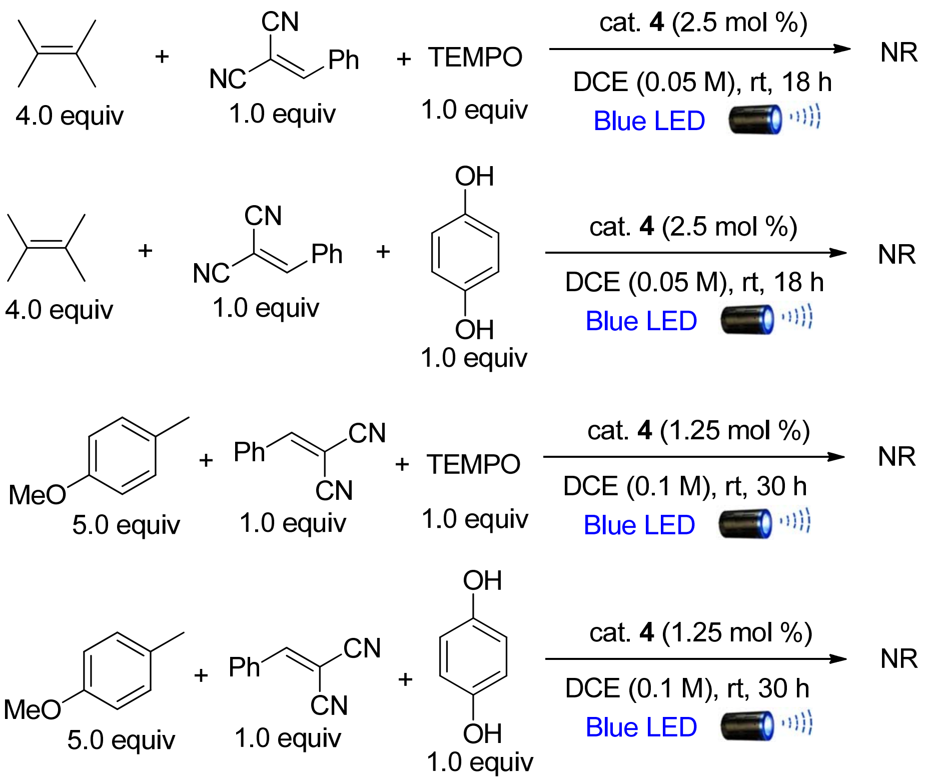

When TEMPO (1.0 equiv.) or hydroquinone (1.0 equiv.) was introduced into the model reaction, no corresponding product was observed according to both TLC and GC-Mass analysis. This result showed that a free radical process was involved.

b) Stern-Volmer fluorescense quenching experiments

In a typical experiment, a solution of 9-mesityl-10-methylacridinium perchlorate **4** in DCE (1.25×10^{-3} M) was added the appropriate amount of quencher in a quartz cuvette. Then the emission of the sample was collected. The emission intensity at 510 nm was collected with excited wavelength of 450 nm.

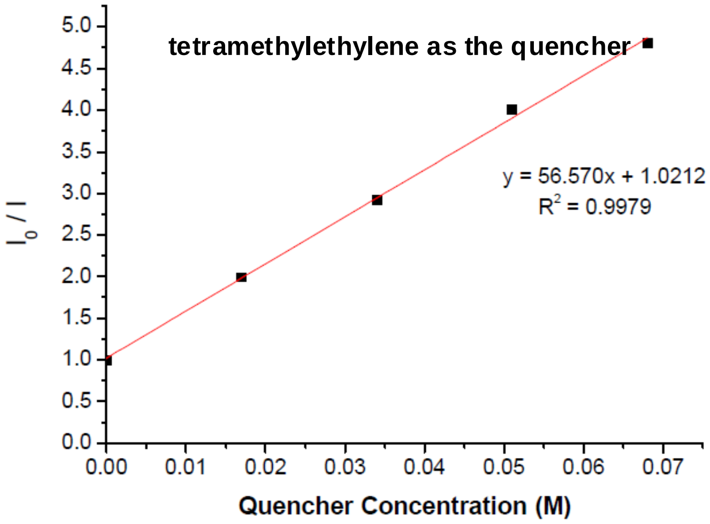

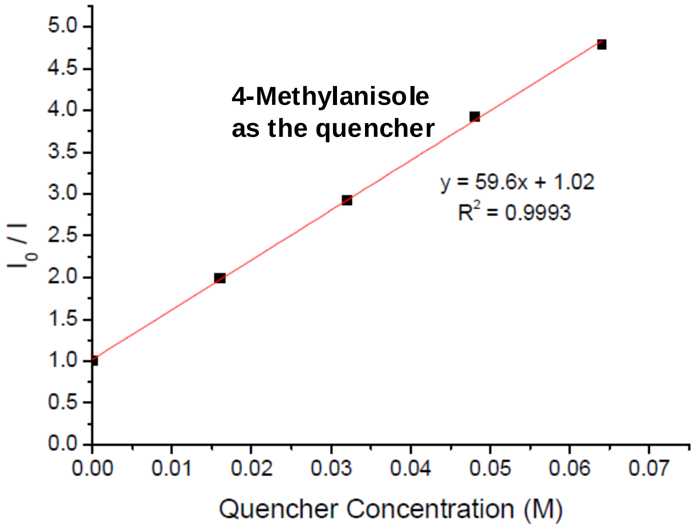

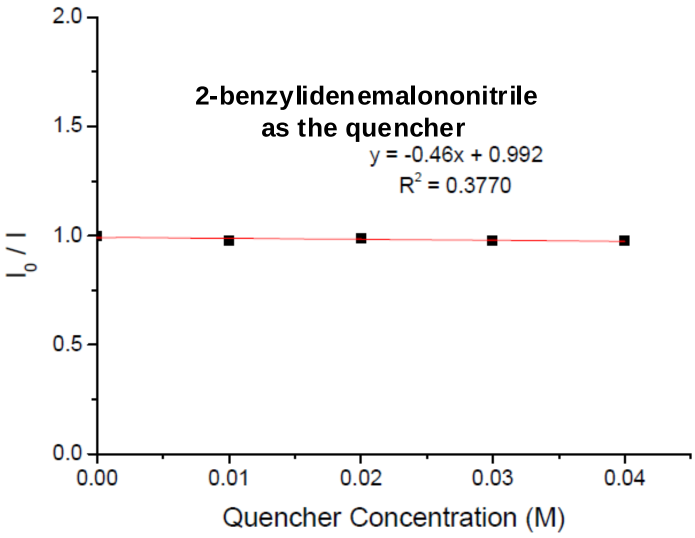

c) Deuterium-labeling experiments

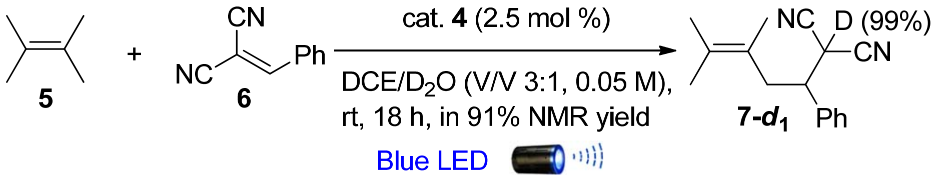

To a 25 mL Schlenk tube equipped with a magnetic stir bar was added 2-benzylidenemalononitrile **6** (31.0 mg, 0.2 mmol), 9-mesityl-10-methylacridinium perchlorate **4** (2.1 mg, 0.005 mmol, 0.025 equiv.). The resulting mixture was sealed and degassed via vacuum evacuation and subsequent backfill with argon for three times. Then anhydrous DCE and D_2O (V/V = 3/1, 4.0 mL) were added, and finally tetramethylethylene **5** (95.0 µL, 0.8 mmol, 4.0 equiv.) was added. After that, the reaction was placed under a blue LED with an argon balloon and irradiated for 18 hrs. The solvent was removed on a rotary evaporator under reduced pressure and

the residue was used for crude ^{1}H NMR test with 1,3,5-trimethoxybenzene as the internal standard (Figure S3). It gave the deuterated product **7-*d*$_1$** in 91% NMR yield. Furthermore, when product **7** was subjected to the same reaction conditions, no H/D exchange occurred.

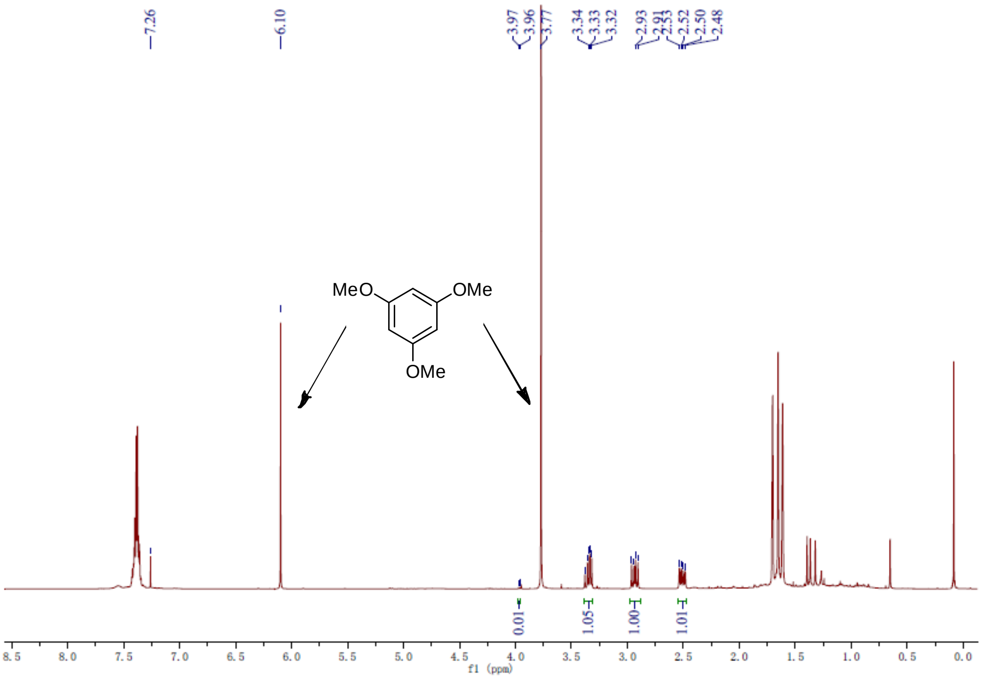

Figure S3. ^{1}H NMR spectra of the crude mixture of **7-*d*$_1$**

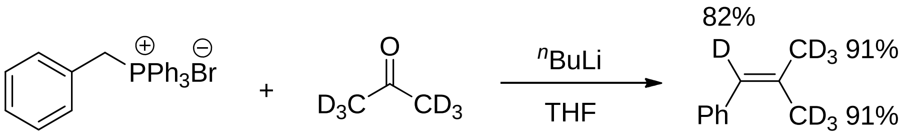

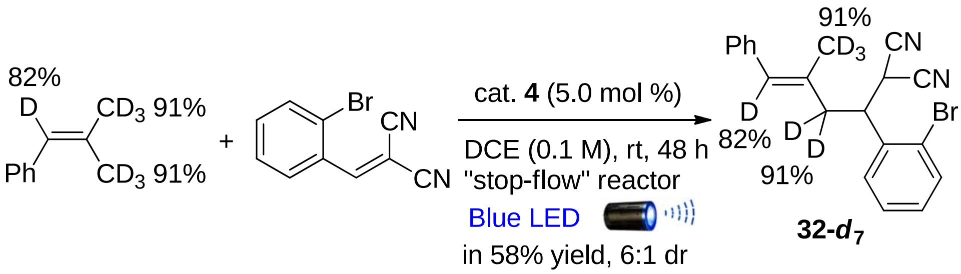

32-*d*$_7$

To a THF (50 mL) solution of benzyltriphenylphosphonium bromide (4.32 g, 10 mmol) was added ^{n}BuLi (2.0 M, 5.1 mL, 10.1 mmol) at 0 ^{o}C. The resulting mixture was stirred at 0 ^{0}C for 2 hrs until the solution became clear. After acetone-*d*$_6$ (0.83 mL, 11 mmol) was added, the solution was heated to 50 ^{0}C for 24 hrs. Then, the reaction was quenched by aqueous NH_4Cl solution. The mixture was extracted with ethyl acetate, dried over anhydrous Na_2SO_4, and purified using column chromatography isolation on silica gel by gradient elution with hexane / ethyl acetate (15:1-10:1)

to give 2-methyl-1-phenyl-1-propene-d_7 (0.9 g) in 65% yield. ^{1}H NMR (400 MHz, $CDCl_3$) δ 7.35 – 7.29 (m, 2H), 7.26 – 7.21 (m, 2H), 7.21 – 7.16 (m, 1H), 6.28 (s, 0.18H), 1.92 – 1.81 (m, 0.53H) (Figure S4).

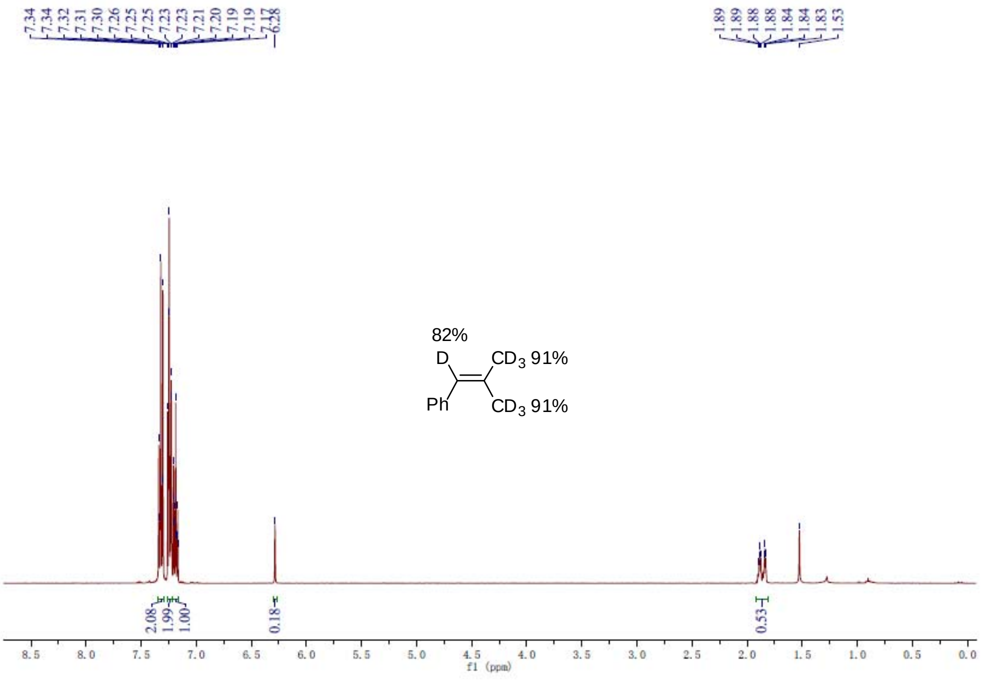

Figure S4. ^{1}H NMR spectra of 2-methyl-1-phenyl-1-propene-d_7

Under Argon atomsphere, a solution of 2-(2-bromobenzylidene)malononitrile (0.2 mmol), 9-mesityl-10-methylacridinium perchlorate **4** (0.01 mmol, 0.05 equiv.), and 2-methyl-1-phenyl-1-propene-d_7 (111 mg, 0.8 mmol, 4.0 equiv.) in anhydrous DCE (2.0 mL) was introduced into a silicon septa vial. Then the solution was pumped into a stop-flow micro tubing (SFMT) reactor (total volume 1.5 mL) which was made of HPFA tubing (O.D. 1/16", I.D. 0.03", 340 cm) via a syringe. The valves were closed, and the SFMT was placed under a blue LED and irradiated for 48 hrs. The solvent was removed on a rotary evaporator under reduced pressure and the residue was subjected to column chromatography isolation on silica gel by gradient elution with hexane / ethyl acetate (15:1-10:1) to give the alkylation product **32-d_7** (32 mg) in 58% yield and 6:1 rr with the *E*-isomer being the major. ^{1}H NMR (400 MHz, $CDCl_3$) δ 7.66 (dd, *J* = 8.0, 1.2 Hz, 1H), 7.53 (dd, *J* = 7.9, 1.5 Hz, 1H), 7.41 (td, *J* = 7.7, 1.2 Hz, 1H), 7.33 – 7.28 (m, 2H), 7.25 – 7.18 (m, 2H), 7.18 – 7.11 (m, 2H), 6.41 (s, 0.18H), 4.23 (d, *J* = 4.8 Hz, 1H), 4.09 (d, *J* = 4.8 Hz, 1H), 2.95 (d, *J* = 7.2 Hz, 0.09H), 2.82 (d, *J* = 8.3 Hz, 0.09H), 1.90 – 1.82 (m, 0.27H) (Figure S5).

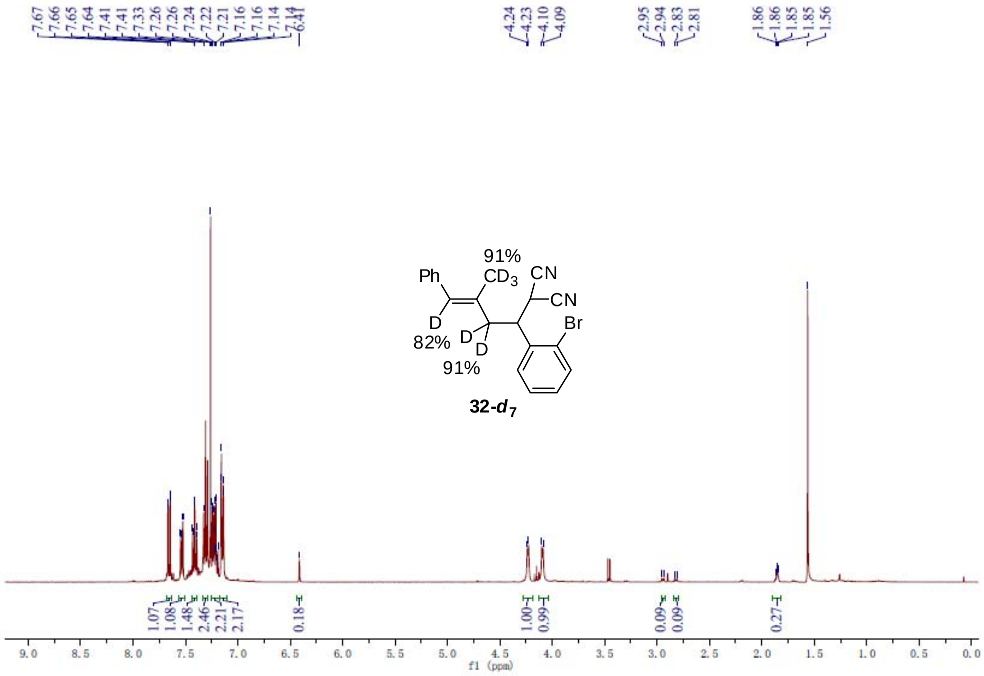

Figure S5. ^{1}H NMR spectra of product **32-*d*$_7$**

The above deuterium-labeling results strongly imply that the anion intermediate **V** would most likely abstract proton from solvent instead of substrate **1**.

d) Kinetic isotope effect experiments

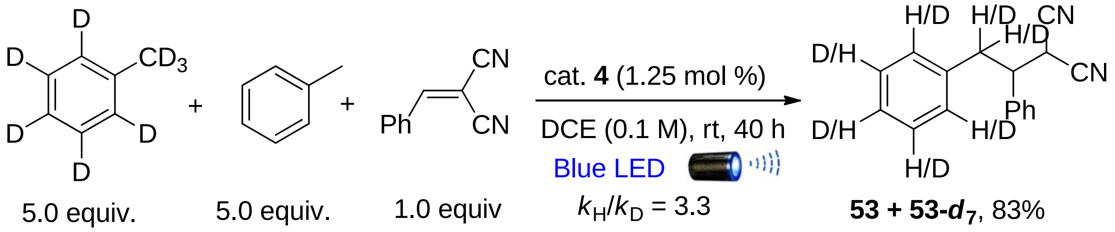

Under Argon atomsphere, a solution of 2-benzylidenemalononitrile **6** (31.0 mg, 0.2 mmol), 9-mesityl-10-methylacridinium perchlorate **4** (1.0 mg, 0.0025 mmol, 0.0125 equiv.), toluene (106 µL, 1.0 mmol, 5.0 equiv.), and toluene-d_8 (106 µL, 1.0 mmol, 5.0 equiv.) in anhydrous DCE (2.0 mL) was introduced into a silicon septa vial. Then the solution was pumped into a stop-flow micro tubing (SFMT) reactor (total volume 1.5 mL) which was made of HPFA tubing (O.D. 1/16", I.D. 0.03", 340 cm) via a syringe. The valves were closed, and the SFMT was placed under a blue LED and irradiated for 40 hrs. The solvent was removed on a rotary evaporator under reduced pressure and the residue was subjected to column chromatography isolation on silica gel by gradient elution with hexane / ethyl acetate (15:1-10:1) to give the desired product **53** and **53-*d*$_7$** (30.6 mg) in 83% yield. The ^{1}H NMR of the product showed that the position of the benzyl was 1.54 H, thus the

value of the isotopic effects was k_H/k_D = 3.3 (Figure S6).

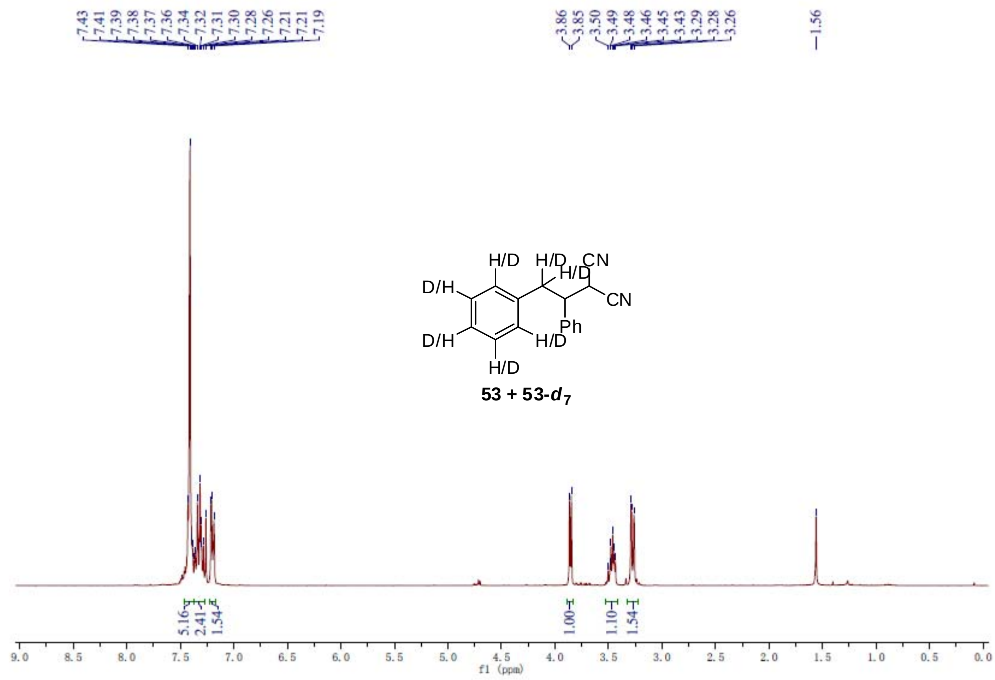

Figure S6. ^{1}H NMR spectra of product **53 + 53-*d*$_7$**

IX. Proposed Mechanism Involving Hydrogen Atom Transfer (HAT) Process and the DFT Calculation Results

A mechanism involving hydrogen atom transfer process was also proposed to rationalize the alkylation of allylic/benzylic sp^3 C–H bonds. As shown in Scheme S1, the radical cation **VI** generated via photoredox mediated SET process can accomplish an intermolecular H-atom abstraction from another molecule of **5**, in which case the radical cation itself served as an HAT catalyst. However, the quantum chemistry calculation using a composite method, CBS-QB3, did not support this process, as the proposed new generated C–H bond in HAT product **VII** is much weaker than the allylic C–H bond in **7** (72.4 kcal / mol vs 83.9 kcal / mol) (Scheme S2).

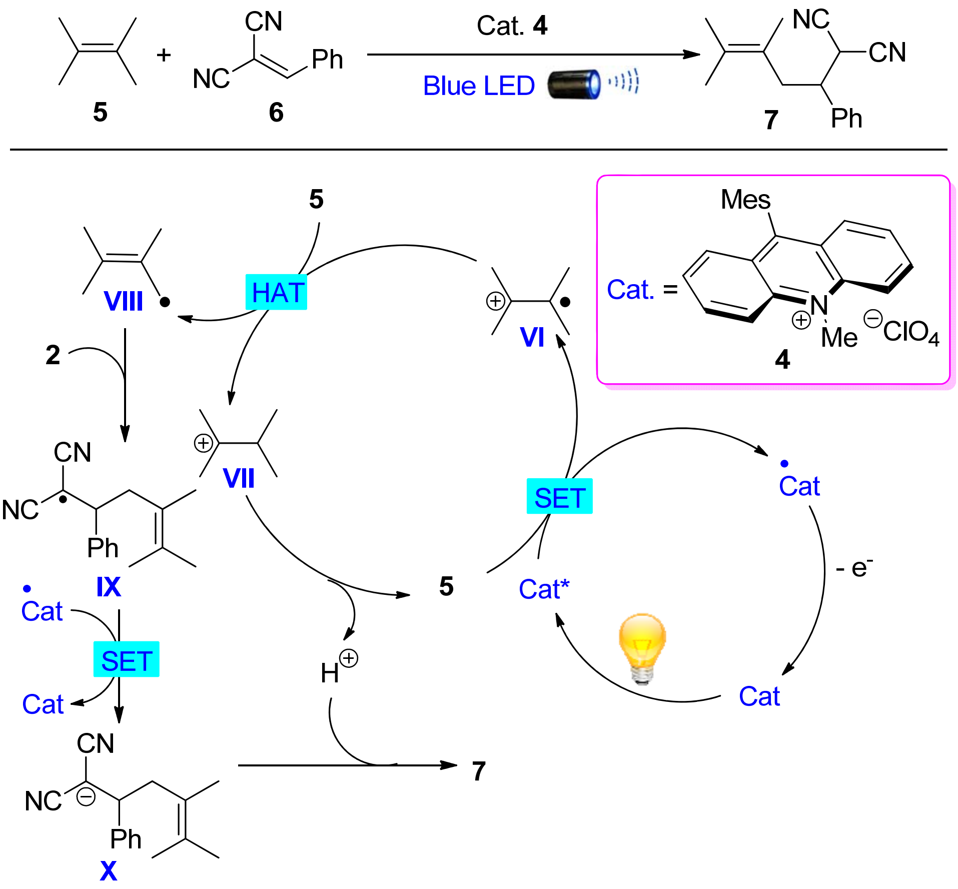

Scheme S1. Proposed hydrogen atom transfer mechanism

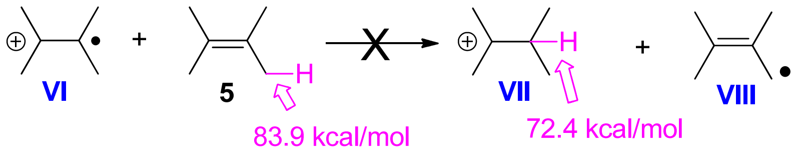

Scheme S2. Calculated C–H bond dissociation energy

Computational details (Methodology):

The quantum chemistry calculations are performed using the Gaussian 09 program[1]. A composite method, CBS-QB3[2,3] is used to carry out the calculations of geometry, frequency and energy within the Polarizable Continuum Model (PCM)[4]. The CBS-QB3 method calculates geometries and frequencies at the B3LYP/CBSB7 level of theory, and the energy is obtained by energy calculations at the CCSD(T)/6-31+G(d') and MP4SDQ/CBSB4 levels of theories and extrapolations to the complete basis set limit. All molecular structures have no imaginary frequencies and are local minima on the potential energy surfaces. Expression (a), formulated by Blanksby and Ellison[5] is used to calculate the bond dissociation energy (BDE) for a dissociation

process X–Y → X$^{\bullet}$ + Y$^{\bullet}$

D_0(X-Y) = E_0 (X) + E_0(Y) – E_0(X-Y)   (a)

where D_0(X-Y), E_0(X), E_0(Y) and E_0(X-Y) denote the BDE to be calculated, computed electronic energies at 0 K with zero-point energy (ZPE) corrections for X, Y and X-Y, respectively.

Table S2. Optimized geometries (Angstrom) and Energies (Hartree) for species involved in the BDE calculations (CBS-QB3, SCRF = (solvent = CH_2ClCH_2Cl, PCM))

**VII** ⟶ **VI** + H•			
**VII** 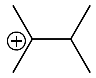	CBS-QB3 (0 K)= -235.712831<br>SCFR = (solvent = CH_2ClCH_2Cl, PCM)		
	1.704025	0.852496	-0.521040
	2.669610	0.417421	-0.782943
	1.427986	1.558771	-1.306607
	1.830595	1.401667	0.413553
	-1.802205	-0.773065	-0.495752
	-2.329353	-0.213315	-1.288757
	-1.476679	-1.724774	-0.909396
	-2.543432	-0.899651	0.297895
	-0.703970	0.101270	-0.080074
	-1.010470	1.334887	0.645735
	-2.015541	1.359130	1.059645
	-0.254860	1.569143	1.399083
	-0.920982	2.143826	-0.100785
	0.674435	-0.272784	-0.404723
	0.658985	-0.881664	-1.313880
	1.048365	-1.275704	0.765314
	0.361907	-2.119165	0.826261
	2.043323	-1.650485	0.522426
	1.087367	-0.763499	1.726748
**VI** 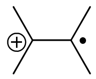	CBS-QB3 (0 K)= -235.097591<br>SCFR = (solvent = CH_2ClCH_2Cl, PCM)		
	-1.478296	1.260321	0.141107
	-2.442377	1.074099	0.616634
	-0.939826	2.044478	0.670851
	-1.701406	1.635399	-0.871120
	1.478387	-1.260352	0.141153
	2.442179	-1.074157	0.617290
	0.939776	-2.044815	0.670283
	1.702165	-1.634947	-0.871108
	0.712553	-0.000039	0.000067

	1.478329 1.260330 -0.140794
	2.442291 1.074225 -0.616616
	0.939801 2.044736 -0.670101
	1.701725 1.634968 0.871535
	-0.712547 -0.000035 -0.000060
	-1.478416 -1.260286 -0.141470
	-0.939715 -2.044795 -0.670427
	-1.702639 -1.634847 0.870707
	-2.442027 -1.073975 -0.617945
H$^{\bullet}$	CBS-QB3 (0 K)= -0.499834<br>SCFR = (solvent = CH_2ClCH_2Cl, PCM)
	0.000000 0.000000 0.000000
**5 → VIII** + H$^{\bullet}$	
**5**<br>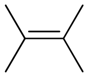	CBS-QB3 (0 K)=-235.333327<br>SCFR = (solvent = CH_2ClCH_2Cl, PCM)
	-1.526181 1.249877 0.005092
	-2.209886 1.237299 0.862438
	-0.960890 2.178362 0.047457
	-2.158988 1.283666 -0.890182
	1.526271 -1.249868 0.005093
	2.210095 -1.237157 0.862341
	0.961072 -2.178397 0.047624
	2.158961 -1.283693 -0.890265
	0.673189 -0.000022 0.000002
	1.526181 1.249877 -0.005093
	2.209942 1.237258 -0.862393
	0.960893 2.178361 -0.047539
	2.158929 1.283709 0.890222
	-0.673190 -0.000022 -0.000001
	-1.526271 -1.249868 -0.005092
	-0.961068 -2.178399 -0.047526
	-2.159034 -1.283643 0.890214
	-2.210024 -1.237206 -0.862397
**VIII**<br>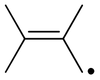	CBS-QB3 (0 K)=-234.699798<br>SCFR = (solvent = CH_2ClCH_2Cl, PCM)
	-0.651651 -0.054669 -0.000044
	0.742439 0.163211 -0.000019
	-1.266686 -1.423667 0.000044
	-1.915719 -1.557411 0.876107
	-1.915514 -1.557619 -0.876138
	-0.535781 -2.230902 0.000229
	-1.618769 1.101091 -0.000034
	-2.651795 0.747945 -0.000873

	-1.488798	1.742883	0.879497
	-1.487676	1.743877	-0.878655
	1.696873	-1.020481	-0.000028
	2.732634	-0.677627	-0.000064
	1.555995	-1.654515	0.880276
	1.555939	-1.654540	-0.880306
	1.284749	1.433464	0.000044
	0.670794	2.324763	0.000082
	2.358184	1.579454	0.000062

X. Analytical Data of the Products

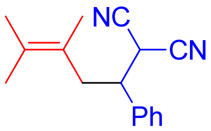

2-(3,4-Dimethyl-1-phenylpent-3-en-1-yl)malononitrile (**7**)

Following the typical procedure **III**, tetramethylethylene **5** (95 μL, 0.8 mmol, 4.0 equiv), 2-benzylidenemalononitrile **6** (31 mg, 0.2 mmol, 1.0 equiv), and 9-mesityl-10-methylacridinium perchlorate **4** (2.1 mg, 0.005 mmol, 0.025 equiv) in DCE (4.0 mL) were employed to give the product **7** (43 mg) in 91% yield as a colorless oil. ^{1}H NMR (300 MHz, CDCl$_3$) δ 7.54 – 7.33 (m, 5H), 4.00 (d, J = 5.1 Hz, 1H), 3.38 (ddd, J = 8.7, 6.7, 5.2 Hz, 1H), 2.98 (dd, J = 13.9, 8.7 Hz, 1H), 2.55 (dd, J = 13.9, 6.7 Hz, 1H), 1.75 (s, 3H), 1.70 (s, 3H), 1.66 (s, 3H); ^{13}C NMR (75 MHz, CDCl$_3$) δ 137.1, 130.1, 128.9, 128.8, 127.9, 122.6, 112.4, 111.9, 45.4, 37.3, 28.5, 20.9, 20.6, 18.3; HRMS–ESI [M – H]$^-$ Calcd for $C_{16}H_{17}N_2$ 237.1397, found 237.1395.

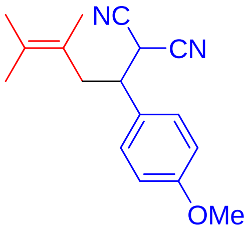

2-(1-(4-Methoxyphenyl)-3,4-dimethylpent-3-en-1-yl)malononitrile (**8**)

Following the typical procedure **III**, tetramethylethylene **5** (95 μL, 0.8 mmol, 4.0 equiv), 2-(4-methoxybenzylidene)malononitrile (37 mg, 0.2 mmol, 1.0 equiv), and 9-mesityl-10-methylacridinium perchlorate **4** (2.1 mg, 0.005 mmol, 0.025 equiv) in DCE (4.0 mL) were employed to give the product **8** (28.4 mg) in 53% yield as a colorless oil. ^{1}H NMR (400 MHz, CDCl$_3$) δ 7.33 – 7.27 (m, 2H), 6.95 – 6.89 (m, 2H), 3.91 (d, J = 5.0 Hz, 1H), 3.82 (s, 3H), 3.30 (ddd, J = 8.8, 6.6, 5.0 Hz, 1H), 2.91 (dd, J = 14.0, 8.8 Hz, 1H), 2.47 (dd, J = 14.0, 6.7 Hz,

1H), 1.71 (s, 3H), 1.66 (s, 3H), 1.61 (s, 3H); ^{13}C NMR (101 MHz, $CDCl_3$) δ 159.8, 129.9, 129.1, 122.7, 114.3, 112.6, 112.0, 55.3, 44.8, 37.3, 28.8, 20.9, 20.7, 18.3; HRMS–ESI [M – H]$^-$ Calcd for $C_{17}H_{19}N_2O$ 267.1503, found 267.1503.

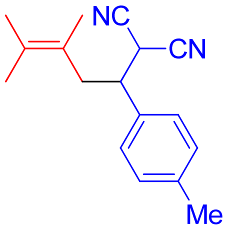

2-(3,4-Dimethyl-1-(p-tolyl)pent-3-en-1-yl)malononitrile (9)

Following the typical procedure **III**, tetramethylethylene **5** (95 μL, 0.8 mmol, 4.0 equiv), 2-(4-methylbenzylidene)malononitrile (34 mg, 0.2 mmol, 1.0 equiv), and 9-mesityl-10-methylacridinium perchlorate **4** (2.1 mg, 0.005 mmol, 0.025 equiv) in DCE (4.0 mL) were employed to give the product **9** (40.2 mg) in 80% yield as a colorless oil. ^{1}H NMR (300 MHz, $CDCl_3$) δ 7.37 – 7.20 (m, 4H), 3.97 (d, *J* = 5.0 Hz, 1H), 3.35 (ddd, *J* = 8.8, 6.5, 5.2 Hz, 1H), 2.97 (dd, *J* = 14.0, 8.8 Hz, 1H), 2.52 (dd, *J* = 14.1, 6.8 Hz, 1H), 2.41 (s, 3H), 1.76 (s, 3H), 1.70 (s, 3H), 1.66 (s, 3H); ^{13}C NMR (75 MHz, $CDCl_3$) δ 138.6, 134.1, 129.9, 129.6, 127.8, 122.7, 112.5, 111.9, 45.1, 37.2, 28.6, 21.1, 20.9, 20.6, 18.3; HRMS–ESI [M – H]$^-$ Calcd for $C_{17}H_{19}N_2$ 251.1554, found 251.1549.

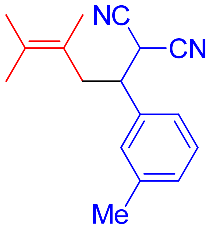

2-(3,4-Dimethyl-1-(m-tolyl)pent-3-en-1-yl)malononitrile (10)

Following the typical procedure **III**, tetramethylethylene **5** (95 μL, 0.8 mmol, 4.0 equiv), 2-(3-methylbenzylidene)malononitrile (34 mg, 0.2 mmol, 1.0 equiv), and 9-mesityl-10-methylacridinium perchlorate **4** (2.1 mg, 0.005 mmol, 0.025 equiv) in DCE (4.0 mL) were employed to give the product **10** (32 mg) in 63% yield as a colorless oil. ^{1}H NMR (300 MHz, $CDCl_3$) δ 7.34 – 7.24 (m, 1H), 7.24 – 7.09 (m, 3H), 3.94 (d, *J* = 5.1 Hz, 1H), 3.41 – 3.21 (m, 1H), 2.93 (dd, *J* = 14.0, 8.8 Hz, 1H), 2.48 (dd, *J* = 13.9, 6.5 Hz, 1H), 2.38 (s, 3H), 1.71 (s, 3H), 1.66 (s, 3H), 1.62 (s, 3H); ^{13}C NMR (75 MHz, $CDCl_3$) δ 138.7, 137.1, 130.0, 129.5, 128.8, 128.6, 124.9, 122.7, 112.5, 111.9, 45.3, 37.3, 28.5, 21.4, 20.9, 20.7, 18.3; HRMS–ESI [M – H]$^-$ Calcd for

$C_{17}H_{19}N_2$ 251.1554, found 251.1544.

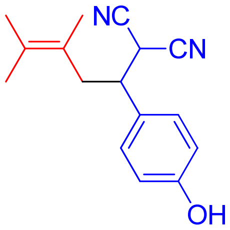

2-(1-(4-Hydroxyphenyl)-3,4-dimethylpent-3-en-1-yl)malononitrile (11)

Following the typical procedure **III**, tetramethylethylene **5** (95 μL, 0.8 mmol, 4.0 equiv), 2-(4-hydroxybenzylidene)malononitrile (34 mg, 0.2 mmol, 1.0 equiv), and 9-mesityl-10-methylacridinium perchlorate **4** (2.1 mg, 0.005 mmol, 0.025 equiv) in DCE (4.0 mL) were employed to give the product **11** (27.4 mg) in 54% yield as a colorless oil. ^{1}H NMR (300 MHz, $CDCl_3$) δ 7.28 (d, J = 8.5 Hz, 2H), 6.88 (d, J = 8.3 Hz, 2H), 5.40 (s, 1H), 3.97 (d, J = 4.9 Hz, 1H), 3.42 – 3.24 (m, 1H), 2.94 (dd, J = 14.0, 8.9 Hz, 1H), 2.50 (dd, J = 14.0, 6.5 Hz, 1H), 1.75 (s, 3H), 1.70 (s, 3H), 1.65 (s, 3H); ^{13}C NMR (75 MHz, $CDCl_3$) δ 156.1, 130.0, 129.3, 122.7, 115.9, 112.6, 111.9, 104.9, 44.8, 37.3, 28.8, 20.9, 20.7, 18.3; HRMS–ESI [M – H]$^-$ Calcd for $C_{16}H_{17}N_2O$ 253.1346, found 253.1348.

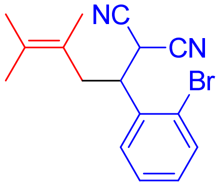

2-(1-(2-Bromophenyl)-3,4-dimethylpent-3-en-1-yl)malononitrile (12)

Following the typical procedure **III**, tetramethylethylene **5** (95 μL, 0.8 mmol, 4.0 equiv), 2-(2-bromobenzylidene)malononitrile (47 mg, 0.2 mmol, 1.0 equiv), and 9-mesityl-10-methylacridinium perchlorate **4** (2.1 mg, 0.005 mmol, 0.025 equiv) in DCE (4.0 mL) were employed to give the product **12** (58.4 mg) in 92% yield as a colorless oil. ^{1}H NMR (300 MHz, $CDCl_3$) δ 7.67 (d, J = 8.0 Hz, 1H), 7.54 (d, J = 7.6 Hz, 1H), 7.42 (t, J = 7.6 Hz, 1H), 7.34 – 7.20 (m, 1H), 4.18 (dd, J = 13.0, 7.4 Hz, 1H), 4.06 (d, J = 5.2 Hz, 1H), 2.91 (dd, J = 14.0, 7.4 Hz, 1H), 2.68 (dd, J = 14.0, 7.8 Hz, 1H), 1.77 (s, 3H), 1.67 (s, 6H); ^{13}C NMR (75 MHz, $CDCl_3$) δ 136.5, 133.6, 130.1, 130.0, 128.4, 128.1, 125.1, 122.1, 111.9, 111.7, 43.1, 36.6, 27.7, 20.9, 20.7, 18.3; HRMS–ESI [M – H]$^-$ Calcd for $C_{16}H_{16}BrN_2$ 315.0502, found 315.0499.

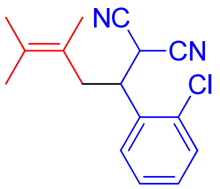

2-(1-(2-Chlorophenyl)-3,4-dimethylpent-3-en-1-yl)malononitrile (13)

Following the typical procedure **III**, tetramethylethylene **5** (95 μL, 0.8 mmol, 4.0 equiv), 2-(2-chlorobenzylidene)malononitrile (38 mg, 0.2 mmol, 1.0 equiv), and 9-mesityl-10-methylacridinium perchlorate **4** (2.1 mg, 0.005 mmol, 0.025 equiv) in DCE (4.0 mL) were employed to give the product **13** (47 mg) in 86% yield as a colorless oil. ^{1}H NMR (300 MHz, $CDCl_3$) δ 7.46 – 7.34 (m, 3H), 7.34 – 7.24 (m, 1H), 4.00 (d, *J* = 5.2 Hz, 1H), 3.43 – 3.25 (m, 1H), 2.94 (dd, *J* = 14.0, 8.7 Hz, 1H), 2.53 (dd, *J* = 13.9, 6.8 Hz, 1H), 1.74 (s, 3H), 1.70 (s, 3H), 1.66 (s, 3H); ^{13}C NMR (75 MHz, $CDCl_3$) δ 139.1, 134.9, 130.5, 130.3, 129.1, 128.2, 126.1, 122.1, 112.1, 111.6, 45.0, 37.2, 28.3, 20.9, 20.7, 18.3; HRMS–ESI [M – H]$^-$ Calcd for $C_{16}H_{16}ClN_2$ 271.1007, found 271.0998.

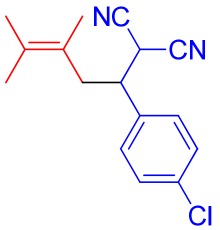

2-(1-(4-Chlorophenyl)-3,4-dimethylpent-3-en-1-yl)malononitrile (14)

Following the typical procedure **III**, tetramethylethylene **5** (95 μL, 0.8 mmol, 4.0 equiv), 2-(4-chlorobenzylidene)malononitrile (38 mg, 0.2 mmol, 1.0 equiv), and 9-mesityl-10-methylacridinium perchlorate **4** (2.1 mg, 0.005 mmol, 0.025 equiv) in DCE (4.0 mL) were employed to give the product **14** (50.5 mg) in 93% yield as a colorless oil. ^{1}H NMR (300 MHz, $CDCl_3$) δ 7.48 – 7.27 (m, 4H), 3.95 (d, *J* = 5.0 Hz, 1H), 3.33 (ddd, *J* = 8.7, 6.7, 5.1 Hz, 1H), 2.90 (dd, *J* = 14.0, 8.7 Hz, 1H), 2.48 (dd, *J* = 14.0, 6.7 Hz, 1H), 1.70 (s, 3H), 1.65 (s, 3H), 1.61 (s, 3H); ^{13}C NMR (75 MHz, $CDCl_3$) δ 135.5, 134.8, 130.4, 129.3, 129.2, 122.2, 112.2, 111.7, 44.8, 37.1, 28.4, 20.9, 20.7, 18.3; HRMS–ESI [M – H]$^-$ Calcd for $C_{16}H_{16}ClN_2$ 271.1007, found 271.0999.

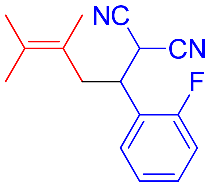

2-(1-(2-Fluorophenyl)-3,4-dimethylpent-3-en-1-yl)malononitrile (**15**)

Following the typical procedure **III**, tetramethylethylene **5** (95 μL, 0.8 mmol, 4.0 equiv), 2-(2-fluorobenzylidene)malononitrile (35 mg, 0.2 mmol, 1.0 equiv), and 9-mesityl-10-methylacridinium perchlorate **4** (2.1 mg, 0.005 mmol, 0.025 equiv) in DCE (4.0 mL) were employed to give the product **15** (50 mg) in 98% yield as a pale yellow oil. ^{1}H NMR (300 MHz, $CDCl_3$) δ 7.47 – 7.32 (m, 2H), 7.27 – 7.06 (m, 2H), 4.11 (d, J = 6.9 Hz, 1H), 3.84 – 3.71 (m, 1H), 2.89 (dd, J = 13.8, 7.2 Hz, 1H), 2.63 (dd, J = 13.8, 8.3 Hz, 1H), 1.67 (s, 3H), 1.65 (s, 3H); ^{13}C NMR (75 MHz, $CDCl_3$) δ160.7 (d, J = 246.2 Hz), 130.4 (d, J = 8.7 Hz), 130.0, 129.2 (d, J = 3.7 Hz), 124.7 (d, J = 3.5 Hz), 124.3 (d, J = 13.3 Hz), 122.3, 115.9 (d, J = 22.5 Hz), 112.1, 111.9, 39.2, 36.8, 27.5 (d, J = 2.7 Hz), 20.8, 20.4, 18.3; HRMS–APCI [M + H]$^+$ Calcd for $C_{16}H_{18}FN_2$ 257.1449, found 257.1451.

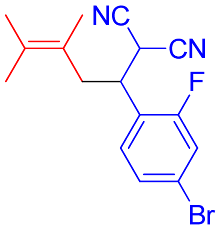

2-(1-(4-Bromo-2-fluorophenyl)-3,4-dimethylpent-3-en-1-yl)malononitrile (**16**)

Following the typical procedure **III**, tetramethylethylene **5** (95 μL, 0.8 mmol, 4.0 equiv), 2-(4-bromo-2-fluorobenzylidene)malononitrile (50 mg, 0.2 mmol, 1.0 equiv), and 9-mesityl-10-methylacridinium perchlorate **4** (2.1 mg, 0.005 mmol, 0.025 equiv) in DCE (4.0 mL) were employed to give the product **16** (66 mg) in 99% yield as a colorless oil. ^{1}H NMR (600 MHz, $CDCl_3$) δ 7.30 – 7.17 (m, 3H), 3.96 (d, J = 6.6 Hz, 1H), 3.68 – 3.59 (m, 1H), 2.75 (dd, J = 13.8, 7.4 Hz, 1H), 2.48 (dd, J = 13.8, 8.2 Hz, 1H), 1.57 (s, 3H), 1.55 (s, 3H), 1.53 (s, 3H); ^{13}C NMR (151 MHz, $CDCl_3$) δ 160.4 (d, J = 251.1 Hz), 130.4, 130.2 (d, J = 4.3 Hz), 128.1 (d, J = 3.6 Hz), 123.4 (d, J = 13.6 Hz), 123.0 (d, J = 10.0 Hz), 121.9, 119.7 (d, J = 25.8 Hz), 111.8, 111.6, 38.6, 36.5, 27.3 (d, J = 2.4 Hz), 20.9, 20.5, 18.2; HRMS–ESI [M – H]$^-$ Calcd for $C_{16}H_{15}BrFN_2$ 333.0408, found 333.0411.

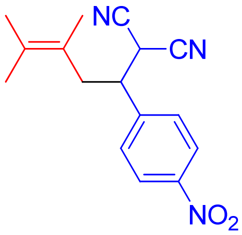

2-(3,4-Dimethyl-1-(4-nitrophenyl)pent-3-en-1-yl)malononitrile (**17**)

Following the typical procedure **III**, tetramethylethylene **5** (95 μL, 0.8 mmol, 4.0 equiv), 2-(4-nitrobenzylidene)malononitrile (40 mg, 0.2 mmol, 1.0 equiv), and 9-mesityl-10-methylacridinium perchlorate **4** (2.1 mg, 0.005 mmol, 0.025 equiv) in DCE (4.0 mL) were employed to give the product **17** (34.2 mg) in 60% yield as a colorless oil. ^{1}H NMR (300 MHz, $CDCl_3$) δ 8.32 (d, J = 8.8 Hz, 2H), 7.62 (d, J = 8.8 Hz, 2H), 4.08 (d, J = 5.1 Hz, 1H), 3.52 (ddd, J = 8.6, 6.8, 5.2 Hz, 1H), 2.99 (dd, J = 14.0, 8.7 Hz, 1H), 2.59 (dd, J = 14.0, 6.8 Hz, 1H), 1.77 (s, 3H), 1.70 (s, 3H), 1.67 (s, 3H); ^{13}C NMR (75 MHz, $CDCl_3$) δ 148.2, 144.1, 131.1, 129.1, 124.1, 121.6, 111.8, 111.3, 45.0, 37.1, 27.9, 20.9, 20.7, 18.2; HRMS–ESI [M – H]$^-$ Calcd for $C_{16}H_{16}N_3O_2$ 282.1248, found 282.1241.

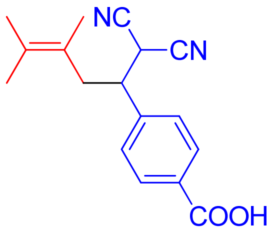

4-(1,1-Dicyano-4,5-dimethylhex-4-en-2-yl)benzoic acid (**18**)

Following the typical procedure **III**, tetramethylethylene **5** (95 μL, 0.8 mmol, 4.0 equiv), 4-(2,2-dicyanovinyl)benzoic acid (40 mg, 0.2 mmol, 1.0 equiv), and 9-mesityl-10-methylacridinium perchlorate **4** (2.1 mg, 0.005 mmol, 0.025 equiv) in DCE (4.0 mL) were employed to give the product **18** (32.2 mg) in 57% yield as a colorless oil. ^{1}H NMR (300 MHz, $CDCl_3$) δ 8.16 (d, J = 8.2 Hz, 2H), 7.49 (d, J = 8.2 Hz, 2H), 4.01 (d, J = 5.2 Hz, 1H), 3.51 – 3.35 (m, 1H), 2.95 (dd, J = 13.9, 8.4 Hz, 1H), 2.55 (dd, J = 13.9, 6.8 Hz, 1H), 1.69 (s, 3H), 1.65 (s, 3H), 1.62 (s, 3H); ^{13}C NMR (75 MHz, $CDCl_3$) δ 170.9, 142.9, 130.9, 130.6, 129.8, 128.2, 122.0, 112.1, 111.6, 45.3, 37.2, 28.2, 20.9, 20.7, 18.3; HRMS–ESI [M – H]$^-$ Calcd for $C_{17}H_{17}N_2O_2$ 281.1296, found 281.1300.

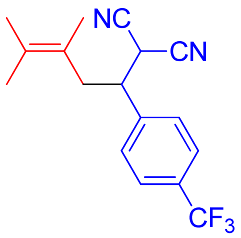

2-(3,4-Dimethyl-1-(4-(trifluoromethyl)phenyl)pent-3-en-1-yl)malononitrile (**19**)

Following the typical procedure **III**, tetramethylethylene **5** (95 μL, 0.8 mmol, 4.0 equiv), 2-(4-(trifluoromethyl)benzylidene)malononitrile (45 mg, 0.2 mmol, 1.0 equiv), and

9-mesityl-10-methylacridinium perchlorate **4** (2.1 mg, 0.005 mmol, 0.025 equiv) in DCE (4.0 mL) were employed to give the product **19** (57 mg) in 93% yield as a colorless oil. ^{1}H NMR (400 MHz, $CDCl_3$) δ 7.68 (d, J = 8.2 Hz, 2H), 7.51 (d, J = 8.2 Hz, 2H), 3.99 (d, J = 5.0 Hz, 1H), 3.41 (ddd, J = 8.9, 6.6, 5.1 Hz, 1H), 2.95 (dd, J = 14.0, 8.9 Hz, 1H), 2.51 (dd, J = 14.0, 6.6 Hz, 1H), 1.71 (s, 3H), 1.66 (s, 3H), 1.62 (s, 3H); ^{13}C NMR (101 MHz, $CDCl_3$) δ 140.9, 131.1 (q, J = 32.8 Hz), 130.8, 128.5, 125.9 (q, J = 3.7 Hz), 123.8 (q, J = 272.3 Hz), 121.9, 112.1, 111.5, 45.1, 37.1, 28.2, 20.9, 20.7, 18.3; HRMS–ESI [M – H]$^-$ Calcd for $C_{17}H_{16}F_3N_2$ 305.1271, found 305.1266.

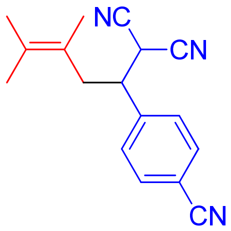

2-(1-(4-Cyanophenyl)-3,4-dimethylpent-3-en-1-yl)malononitrile (**20**)

Following the typical procedure **III**, tetramethylethylene **5** (95 µL, 0.8 mmol, 4.0 equiv), 2-(4-cyanobenzylidene)malononitrile (36 mg, 0.2 mmol, 1.0 equiv), and 9-mesityl-10-methylacridinium perchlorate **4** (2.1 mg, 0.005 mmol, 0.025 equiv) in DCE (4.0 mL) were employed to give the product **20** (48 mg) in 91% yield as a colorless oil. ^{1}H NMR (400 MHz, $CDCl_3$) δ 7.71 (d, J = 8.1 Hz, 2H), 7.50 (d, J = 8.1 Hz, 2H), 4.02 (d, J = 5.1 Hz, 1H), 3.46 – 3.35 (m, 1H), 2.91 (dd, J = 14.0, 8.7 Hz, 1H), 2.51 (dd, J = 14.0, 6.8 Hz, 1H), 1.68 (s, 3H), 1.65 (s, 3H), 1.61 (s, 3H); ^{13}C NMR (101 MHz, $CDCl_3$) δ 142.2, 132.7, 130.9, 128.9, 121.7, 118.1, 112.9, 111.9, 111.4, 45.2, 36.9, 27.9, 20.9, 20.6, 18.2; HRMS–ESI [M – H]$^-$ Calcd for $C_{17}H_{16}N_3$ 262.1350, found 262.1341.

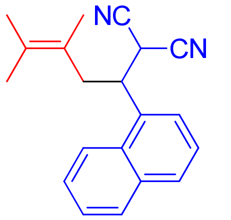

2-(3,4-Dimethyl-1-(naphthalen-1-yl)pent-3-en-1-yl)malononitrile (**21**)

Following the typical procedure **III**, tetramethylethylene **5** (95 µL, 0.8 mmol, 4.0 equiv), 2-(naphthalen-1-ylmethylene)malononitrile (41 mg, 0.2 mmol, 1.0 equiv), and 9-mesityl-10-methylacridinium perchlorate **4** (2.1 mg, 0.005 mmol, 0.025 equiv) in DCE (4.0 mL) were employed to give the product **21** (35.1 mg) in 61% yield as a colorless oil. ^{1}H NMR (600

MHz, $CDCl_3$) δ 8.00 (d, *J* = 8.5 Hz, 1H), 7.92 (d, *J* = 8.1 Hz, 1H), 7.87 (d, *J* = 8.2 Hz, 1H), 7.67 (d, *J* = 7.2 Hz, 1H), 7.63 – 7.57 (m, 1H), 7.57 – 7.50 (m, 2H), 4.41 (d, *J* = 4.3 Hz, 1H), 4.11 (d, *J* = 4.8 Hz, 1H), 3.11 (dd, *J* = 14.0, 7.7 Hz, 1H), 2.72 (dd, *J* = 13.8, 7.2 Hz, 1H), 1.76 (s, 3H), 1.60 (s, 3H), 1.56 (s, 3H); ^{13}C NMR (151 MHz, $CDCl_3$) δ 134.0, 133.4, 131.2, 129.9, 129.4, 129.2, 126.9, 126.1, 125.3, 124.7, 122.6, 121.5, 112.3, 112.2, 38.4, 37.5, 28.5, 20.9, 20.8, 18.5; HRMS–ESI [M – H]$^-$ Calcd for $C_{20}H_{19}N_2$ 287.1554, found 287.1550.

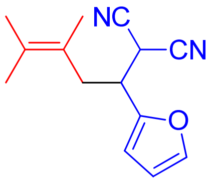

2-(1-(Furan-2-yl)-3,4-dimethylpent-3-en-1-yl)malononitrile (**22**)

Following the typical procedure **III**, tetramethylethylene **5** (95 μL, 0.8 mmol, 4.0 equiv), 2-(furan-2-ylmethylene)malononitrile (29 mg, 0.2 mmol, 1.0 equiv), and 9-mesityl-10-methylacridinium perchlorate **4** (2.1 mg, 0.005 mmol, 0.025 equiv) in DCE (4.0 mL) were employed to give the product **22** (36 mg) in 80% yield as a colorless oil. ^1H NMR (400 MHz, $CDCl_3$) δ 7.43 (s, 1H), 6.36 (t, *J* = 5.1 Hz, 2H), 3.99 (d, *J* = 5.3 Hz, 1H), 3.57 – 3.45 (m, 1H), 2.80 (dd, *J* = 13.9, 8.0 Hz, 1H), 2.59 (dd, *J* = 13.9, 7.5 Hz, 1H), 1.68 (s, 3H), 1.66 (s, 3H), 1.61 (s, 3H); ^{13}C NMR (101 MHz, $CDCl_3$) δ 150.1, 142.9, 130.5, 121.9, 112.0, 111.5, 110.7, 108.9, 39.5, 35.8, 26.9, 20.9, 20.5, 18.0; HRMS–ESI [M – H]$^-$ Calcd for $C_{14}H_{15}N_2O$ 227.1190, found 227.1192.

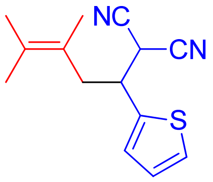

2-(3,4-Dimethyl-1-(thiophen-2-yl)pent-3-en-1-yl)malononitrile (**23**)

Following the typical procedure **III**, tetramethylethylene **5** (95 μL, 0.8 mmol, 4.0 equiv), 2-(thiophen-2-ylmethylene)malononitrile (32 mg, 0.2 mmol, 1.0 equiv), and 9-mesityl-10-methylacridinium perchlorate **4** (2.1 mg, 0.005 mmol, 0.025 equiv) in DCE (4.0 mL) were employed to give the product **23** (24.4 mg) in 50% yield as a colorless oil. ^1H NMR (300 MHz, $CDCl_3$) δ 7.31 (d, *J* = 5.1 Hz, 1H), 7.16 (d, *J* = 3.5 Hz, 1H), 7.10 – 7.00 (m, 1H), 3.98 (d, *J* = 4.4 Hz, 1H), 3.79 – 3.61 (m, 1H), 2.93 (dd, *J* = 14.0, 9.1 Hz, 1H), 2.58 (dd, *J* = 14.0, 6.3 Hz, 1H), 1.74 (s, 3H), 1.68 (s, 3H), 1.65 (s, 3H); ^{13}C NMR (75 MHz, $CDCl_3$) δ 139.5, 130.9, 127.2, 126.5, 125.6, 122.2, 112.3, 111.6, 41.1, 38.7, 29.1, 20.9, 20.7, 18.1; HRMS–ESI [M – H]$^-$ Calcd

for $C_{14}H_{15}N_2S$ 243.0961, found 243.0954.

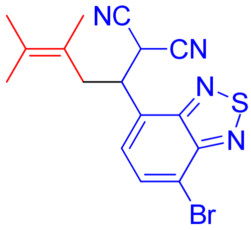

2-(1-(7-Bromobenzo[c][1,2,5]thiadiazol-4-yl)-3,4-dimethylpent-3-en-1-yl)malononitrile (**24**)

Following the typical procedure **III**, tetramethylethylene **5** (95 μL, 0.8 mmol, 4.0 equiv), 2-((7-bromobenzo[c][1,2,5]thiadiazol-4-yl)methylene)malononitrile (59 mg, 0.2 mmol, 1.0 equiv), and 9-mesityl-10-methylacridinium perchlorate **4** (2.1 mg, 0.005 mmol, 0.025 equiv) in DCE (4.0 mL) were employed to give the product **24** (69 mg) in 92% yield as a pale yellow oil. ^{1}H NMR (500 MHz, $CDCl_3$) δ 7.87 (d, J = 7.4 Hz, 1H), 7.43 (d, J = 7.5 Hz, 1H), 4.85 (d, J = 8.3 Hz, 1H), 4.11 – 4.01 (m, 1H), 2.99 – 2.86 (m, 2H), 1.57 (s, 6H), 1.41 (s, 3H); ^{13}C NMR (126 MHz, $CDCl_3$) δ 153.6, 152.7, 131.9, 130.1, 129.8, 129.4, 122.1, 114.9, 112.1, 111.9, 43.4, 36.7, 27.0, 20.7, 20.3, 18.3; HRMS–ESI $[M + H]^+$ Calcd for $C_{16}H_{16}BrN_4S$ 375.0274, found 375.0266.

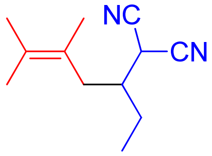

2-(5,6-Dimethylhept-5-en-3-yl)malononitrile (**25**)

Following the typical procedure **III**, tetramethylethylene **5** (95 μL, 0.8 mmol, 4.0 equiv), 2-propylidenemalononitrile (22 mg, 0.2 mmol, 1.0 equiv), and 9-mesityl-10-methylacridinium perchlorate **4** (2.1 mg, 0.005 mmol, 0.025 equiv) in DCE (4.0 mL) were employed to give the product **25** (29.5 mg) in 78% yield as a colorless oil. ^{1}H NMR (400 MHz, $CDCl_3$) δ 3.77 (d, J = 3.4 Hz, 1H), 2.36 – 2.21 (m, 2H), 2.14 – 2.02 (m, 1H), 1.80 – 1.58 (m, 11H), 1.07 (t, J = 7.4 Hz, 3H); ^{13}C NMR (101 MHz, $CDCl_3$) δ 129.9, 122.9, 112.9, 111.9, 41.4, 35.9, 26.0, 24.6, 20.9, 20.7, 18.2, 11.4; HRMS–ESI $[M + H]^+$ Calcd for $C_{12}H_{19}N_2$ 191.1543, found 191.1544.

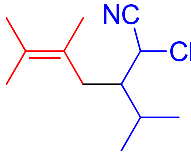

2-(2,5,6-Trimethylhept-5-en-3-yl)malononitrile (**26**)

Following the typical procedure **III**, tetramethylethylene **5** (95 μL, 0.8 mmol, 4.0 equiv), 2-(2-methylpropylidene)malononitrile (24 mg, 0.2 mmol, 1.0 equiv), and

9-mesityl-10-methylacridinium perchlorate **4** (2.1 mg, 0.005 mmol, 0.025 equiv) in DCE (4.0 mL) were employed to give the product **26** (18.8 mg) in 46% yield as a colorless oil. ^{1}H NMR (400 MHz, $CDCl_3$) δ 3.80 (d, *J* = 3.1 Hz, 1H), 2.38 (dd, *J* = 14.3, 10.1 Hz, 1H), 2.24 (dd, *J* = 11.4, 5.2 Hz, 1H), 2.06 – 1.96 (m, 2H), 1.74 (s, 3H), 1.71 (s, 3H), 1.63 (s, 3H), 1.15 (d, *J* = 6.6 Hz, 3H), 1.09 (d, *J* = 6.5 Hz, 3H); ^{13}C NMR (151 MHz, $CDCl_3$) δ 130.3, 123.2, 113.5, 112.4, 45.4, 33.8, 30.3, 23.9, 21.1, 21.0, 20.7, 19.4, 18.1; HRMS–ACPI $[M + H]^+$ Calcd for $C_{13}H_{21}N_2$ 205.1699, found 205.1696.

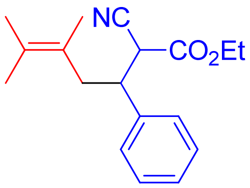

Ethyl 2-cyano-5,6-dimethyl-3-phenylhept-5-enoate (**27**)

Following the typical procedure **III**, tetramethylethylene **5** (95 μL, 0.8 mmol, 4.0 equiv), ethyl 2-cyano-3-phenylacrylate (41 mg, 0.2 mmol, 1.0 equiv), and 9-mesityl-10-methylacridinium perchlorate **4** (2.1 mg, 0.005 mmol, 0.025 equiv) in DCE (4.0 mL) were employed to give the product **27** (35.1 mg) in 62% yield as a diastereomeric mixture with 1.4:1 dr; colorless oil; ^{1}H NMR (400 MHz, $CDCl_3$) δ 7.29 – 7.17 (m, $5H_{Major}$), 7.29 – 7.17 (m, $5H_{Minor}$), 4.03 (q, *J* = 7.1 Hz, $2H_{Major}$), 4.03 (q, *J* = 7.1 Hz, 2 H_{Minor}), 3.71 (d, *J* = 5.2 Hz, $1H_{Major}$), 3.62 (d, *J* = 6.4 Hz, H_{Minor}), 3.49 – 3.38 (m, $1H_{Major}$), 3.49 – 3.38 (m, $1H_{Minor}$), 2.79 (dd, *J* = 13.9, 9.6 Hz, $1H_{Major}$), 2.68 (dd, *J* = 13.6, 5.8 Hz, $1H_{Minor}$), 2.49 (dd, *J* = 13.6, 9.3 Hz, $1H_{Minor}$), 2.34 (dd, *J* = 13.9, 6.1 Hz, 1 H_{Major}), 1.65 (s, $3H_{Major}$), 1.58 (s, $6H_{Major}$), 1.48 (s, $3H_{Minor}$), 1.47 (s, $3H_{Minor}$), 1.43 (s, $3H_{Minor}$), 1.08 (t, *J* = 7.2 Hz, $3H_{Minor}$), 1.05 (t, *J* = 7.1 Hz, $3H_{Major}$); ^{13}C NMR (101 MHz, $CDCl_3$) δ 165.8, 165.2, 139.7, 139.0, 129.1, 128.6, 128.5, 128.2, 128.1, 127.9, 127.8, 127.6, 123.5, 123.4, 116.2, 115.8, 62.6, 62.5, 44.8, 44.5, 44.2, 42.9, 38.2, 37.8, 20.9, 20.7, 20.6, 20.4, 18.5, 18.3, 13.8, 13.7; HRMS–ESI $[M + Na]^+$ Calcd for $C_{18}H_{23}NO_2Na$ 308.1621, found 308.1620.

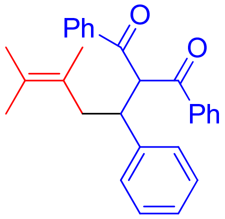

2-(3,4-Dimethyl-1-phenylpent-3-en-1-yl)-1,3-diphenylpropane-1,3-dione (**28**)

Following the typical procedure **III**, tetramethylethylene **5** (95 μL, 0.8 mmol, 4.0 equiv),

2-benzylidene-1,3-diphenylpropane-1,3-dione (63 mg, 0.2 mmol, 1.0 equiv), and 9-mesityl-10-methylacridinium perchlorate **4** (2.1 mg, 0.005 mmol, 0.025 equiv) in DCE (4.0 mL) were employed to give the product **28** (19.6 mg) in 25% yield as a colorless oil. ^{1}H NMR (400 MHz, $CDCl_3$) δ 8.08 – 8.00 (m, 2H), 7.73 – 7.66 (m, 2H), 7.49 (ddd, *J* = 6.7, 3.9, 1.3 Hz, 1H), 7.44 – 7.37 (m, 2H), 7.36 – 7.30 (m, 1H), 7.25 – 7.17 (m, 2H), 7.14 – 7.08 (m, 2H), 7.06 – 6.99 (m, 2H), 6.96 (ddd, *J* = 7.1, 3.8, 1.3 Hz, 1H), 5.66 (d, *J* = 10.7 Hz, 1H), 4.05 (ddd, *J* = 10.6, 8.7, 6.4 Hz, 1H), 2.37 – 2.26 (m, 2H), 1.33 (s, 3H), 1.30 (s, 3H), 1.26 (s, 3H); ^{13}C NMR (101 MHz, $CDCl_3$) δ 194.5, 141.5, 137.4, 137.2, 133.4, 132.9, 128.9, 128.8, 128.6, 128.5, 128.3, 127.9, 126.9, 126.4, 124.8, 64.5, 46.2, 40.1, 20.5, 20.2, 18.5; HRMS–ESI [M – H]$^-$ Calcd for $C_{28}H_{27}O_2$ 395.2017, found 395.2014.

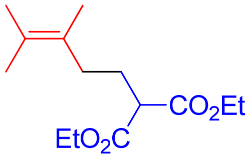

Diethyl 2-(3,4-dimethylpent-3-en-1-yl)malonate (**29**)

Following the typical procedure **III**, tetramethylethylene **5** (95 μL, 0.8 mmol, 4.0 equiv), diethyl 2-methylenemalonate (35 mg, 0.2 mmol, 1.0 equiv), and 9-mesityl-10-methylacridinium perchlorate **4** (2.1 mg, 0.005 mmol, 0.025 equiv) in DCE (4.0 mL) were employed to give the product **29** (26.6 mg) in 52% yield as a colorless oil. ^{1}H NMR (300 MHz, $CDCl_3$) δ 4.25 – 4.12 (m, 4H), 3.27 (t, *J* = 7.2 Hz, 1H), 2.06 (dd, *J* = 9.0, 5.4 Hz, 2H), 2.00 – 1.87 (m, 2H), 1.62 (s, 9H), 1.26 (t, *J* = 7.1 Hz, 6H); ^{13}C NMR (75 MHz, $CDCl_3$) δ 169.5, 126.1, 125.6, 61.2, 51.7, 32.0, 27.2, 20.6, 20.1, 18.1, 14.1; GC/MS (m/z, relative intensity) 256 (M^+, 3), 173 (48), 161 (25), 96 (100), 81 (48), 55 (25).

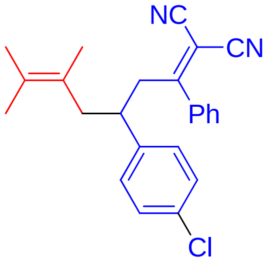

2-(3-(4-Chlorophenyl)-5,6-dimethyl-1-phenylhept-5-en-1-ylidene) malononitrile (**30**)

Following the typical procedure **III**, tetramethylethylene **5** (95 μL, 0.8 mmol, 4.0 equiv), (*E*)-2-(3-(4-chlorophenyl)-1-phenylallylidene)malononitrile (58 mg, 0.2 mmol, 1.0 equiv), and 9-mesityl-10-methylacridinium perchlorate **4** (2.1 mg, 0.005 mmol, 0.025 equiv) in DCE (4.0 mL)

were employed to give the product **30** (36 mg) in 48% yield as a pale yellow oil. ^{1}H NMR (300 MHz, $CDCl_3$) δ 7.65 – 7.42 (m, 3H), 7.26 (d, *J* = 7.9 Hz, 4H), 6.93 (d, *J* = 8.3 Hz, 2H), 3.37 – 3.19 (m, 2H), 2.78 – 2.60 (m, 1H), 2.51 (dd, *J* = 13.5, 8.3 Hz, 1H), 2.24 (dd, *J* = 13.4, 7.0 Hz, 1H), 1.65 (s, 3H), 1.59 (s, 3H), 1.47 (s, 3H); ^{13}C NMR (75 MHz, $CDCl_3$) δ 178.7, 140.3, 134.3, 132.8, 131.9, 129.1, 128.8, 128.7, 127.9, 127.4, 124.2, 112.6, 112.4, 85.7, 43.5, 42.5, 41.8, 20.7, 20.5, 18.5; HRMS–ESI [M – H]$^-$ Calcd for $C_{24}H_{22}ClN_2$ 373.1477, found 373.1479.

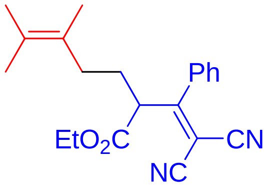

Ethyl 2-(2,2-dicyano-1-phenylvinyl)-5,6-dimethylhept-5-enoate (**31**)

Following the typical procedure **III**, tetramethylethylene **5** (95 μL, 0.8 mmol, 4.0 equiv), ethyl 4,4-dicyano-2-methylene-3-phenylbut-3-enoate (51 mg, 0.2 mmol, 1.0 equiv), and 9-mesityl-10-methylacridinium perchlorate **4** (2.1 mg, 0.005 mmol, 0.025 equiv) in DCE (4.0 mL) were employed to give the product **31** (42 mg) in 63% yield as a pale yellow oil. ^{1}H NMR (300 MHz, $CDCl_3$) δ 7.57 – 7.41 (m, 3H), 7.36 (dd, *J* = 5.9, 4.8 Hz, 2H), 4.21 (q, *J* = 7.2 Hz, 2H), 4.03 (t, *J* = 7.0 Hz, 1H), 2.23 – 2.09 (m, 1H), 2.09 – 1.91 (m, 2H), 1.62 (s, 3H), 1.54 (s, 6H), 1.29 – 1.21 (m, 3H); ^{13}C NMR (75 MHz, $CDCl_3$) δ 176.1, 168.9, 133.4, 131.7, 129.0, 127.6, 126.6, 125.2, 112.2, 111.9, 88.9, 62.2, 51.9, 32.1, 28.5, 20.6, 20.1, 17.9, 13.9; HRMS–ESI [M – H]$^-$ Calcd for $C_{21}H_{23}N_2O_2$ 335.1765, found 335.1768.

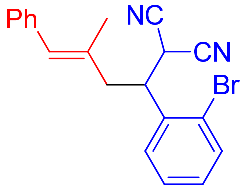

(*E*)-2-(1-(2-bromophenyl)-3-methyl-4-phenylbut-3-en-1-yl) malononitrile (**32**)

In batch: following the typical procedure **III**, (2-methylprop-1-en-1-yl)benzene (106 mg, 0.8 mmol, 4.0 equiv), 2-(2-bromobenzylidene)malononitrile (47 mg, 0.2 mmol, 1.0 equiv), and 9-mesityl-10-methylacridinium perchlorate **4** (4.1 mg, 0.01 mmol, 0.05 equiv) in DCE (2.0 mL) were employed to give the product **32** and a minor uncharacterized isomeric product (combined 45.6 mg) in 63% yield and 7:1 rr as colorless oil. In "stop-flow micro tubing" reactor: following the typical procedure **IV** using a HPFA tubing (total volume 1.5 mL) to afford product **32** and a minor uncharacterized isomeric product (combined 30.5 mg) in 56% yield and 7:1 rr. ^{1}H NMR

(400 MHz, $CDCl_3$) δ 7.66 (dd, *J* = 8.0, 1.3 Hz, 1H), 7.54 (dd, *J* = 7.9, 1.5 Hz, 1H), 7.41 (td, *J* = 7.6, 1.2 Hz, 1H), 7.34 – 7.28 (m, 2H), 7.27 – 7.18 (m, 2H), 7.14 (d, *J* = 7.2 Hz, 2H), 6.41 (s, 1H), 4.32 – 4.20 (m, 1H), 4.09 (d, *J* = 4.8 Hz, 1H), 2.97 (dd, *J* = 14.1, 7.4 Hz, 1H), 2.84 (dd, *J* = 14.0, 8.3 Hz, 1H), 1.89 (d, *J* = 1.3 Hz, 3H); ^{13}C NMR (101 MHz, $CDCl_3$) δ 137.2, 135.8, 133.7, 132.6, 130.3, 129.9, 128.8, 128.3, 128.1, 126.7, 125.1, 111.8, 111.3, 42.3, 27.9, 17.6; HRMS–ESI [M – H]$^-$ Calcd for $C_{20}H_{16}BrN_2$ 363.0502, found 363.0508.

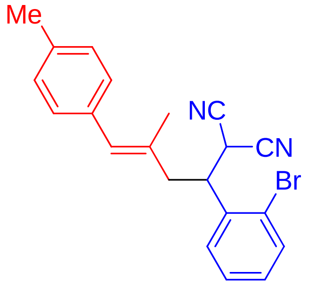

(*E*)-2-(1-(2-bromophenyl)-3-methyl-4-(p-tolyl)but-3-en-1-yl)malononitrile (**33**)

In batch: following the typical procedure **III**, 1-methyl-4-(2-methylprop-1-en-1-yl)benzene (117 mg, 0.8 mmol, 4.0 equiv), 2-(2-bromobenzylidene)malononitrile (47 mg, 0.2 mmol, 1.0 equiv), and 9-mesityl-10-methylacridinium perchlorate **4** (4.1 mg, 0.01 mmol, 0.05 equiv) in DCE (2.0 mL) were employed to give the product **33** and a minor uncharacterized isomeric product (combined 56.7 mg) in 75% yield and 7:1 rr as colorless oil. In "stop-flow micro tubing" reactor: following the typical procedure **IV** using a HPFA tubing (total volume 1.5 mL) to afford product **33** and a minor uncharacterized isomeric product (combined 36.2 mg) in 64% yield and 7:1 rr. ^1H NMR (400 MHz, $CDCl_3$) δ 7.65 (dd, *J* = 8.0, 1.3 Hz, 1H), 7.54 (dd, *J* = 7.9, 1.5 Hz, 1H), 7.41 (td, *J* = 7.6, 1.2 Hz, 1H), 7.23 (ddd, *J* = 8.0, 7.5, 1.6 Hz, 1H), 7.12 (d, *J* = 7.9 Hz, 2H), 7.05 (d, *J* = 8.1 Hz, 2H), 6.38 (s, 1H), 4.29 – 4.20 (m, 1H), 4.09 (d, *J* = 4.9 Hz, 1H), 2.95 (ddd, *J* = 13.9, 7.5, 0.8 Hz, 1H), 2.82 (dd, *J* = 14.0, 8.2 Hz, 1H), 2.33 (s, 3H), 1.89 (d, *J* = 1.3 Hz, 3H); ^{13}C NMR (101 MHz, $CDCl_3$) δ 136.4, 135.9, 134.3, 133.7, 131.8, 130.2, 129.9, 128.8, 128.7, 128.3, 125.1, 111.8, 111.3, 42.4, 27.9, 21.1, 17.6; HRMS–ACPI [M + H]$^+$ Calcd for $C_{21}H_{20}BrN_2$ 379.0804, found 379.0805.

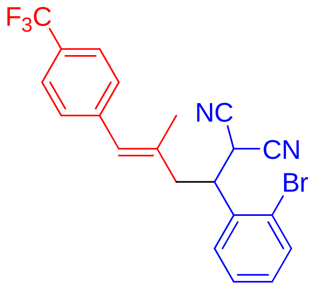

(*E*)-2-(1-(2-bromophenyl)-3-methyl-4-(4-(trifluoromethyl)phenyl)but-3-en-1-yl)malononitrile (34)

In batch: following the typical procedure **III**, 1-(2-methylprop-1-en-1-yl)-4-(trifluoromethyl)benzene (160 mg, 0.8 mmol, 4.0 equiv), 2-(2-bromobenzylidene)malononitrile (47 mg, 0.2 mmol, 1.0 equiv), and 9-mesityl-10-methylacridinium perchlorate **4** (4.1 mg, 0.01 mmol, 0.05 equiv) in DCE (2.0 mL) were employed to give the product **34** and a minor uncharacterized isomeric product (combined 18.6 mg) in 21% yield and 7:1 rr as colorless oil. In "stop-flow micro tubing" reactor: following the typical procedure **IV** using a HPFA tubing (total volume 1.5 mL) to afford product **34** and a minor uncharacterized isomeric product (combined 22 mg) in 34% yield and 7:1 rr. ^{1}H NMR (600 MHz, $CDCl_3$) δ 7.66 (d, *J* = 8.1 Hz, 1H), 7.53 (dd, *J* = 19.7, 7.8 Hz, 3H), 7.42 (t, *J* = 7.5 Hz, 1H), 7.29 – 7.19 (m, 3H), 6.41 (s, 1H), 4.25 (br s, 1H), 4.08 (br s, 1H), 3.01 (dd, *J* = 14.0, 7.1 Hz, 1H), 2.87 (dd, *J* = 13.8, 8.6 Hz, 1H), 1.88 (s, 3H); ^{13}C NMR (151 MHz, $CDCl_3$) δ 140.7, 135.6, 135.0, 133.8, 130.9, 130.4, 128.9, 128.7, 128.6 (q, *J* = 33.5 Hz), 128.4, 125.1 (q, *J* = 3.8 Hz), 124.1 (q, *J* = 272.9 Hz), 111.7, 111.1, 42.1, 28.0, 17.7; HRMS–ESI [M – H]$^-$ Calcd for $C_{21}H_{15}BrF_3N_2$ 431.0376, found 431.0378.

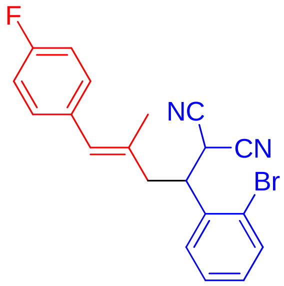

(*E*)-2-(1-(2-bromophenyl)-4-(4-fluorophenyl)-3-methylbut-3-en-1-yl)malononitrile (**35**)

In batch: following the typical procedure **III**, 1-fluoro-4-(2-methylprop-1-en-1-yl)benzene (120 mg, 0.8 mmol, 4.0 equiv), 2-(2-bromobenzylidene)malononitrile (47 mg, 0.2 mmol, 1.0 equiv), and 9-mesityl-10-methylacridinium perchlorate **4** (4.1 mg, 0.01 mmol, 0.05 equiv) in DCE (2.0 mL) were employed to give the product **35** and a minor uncharacterized isomeric product

(combined 31.1 mg) in 41% yield and 4:1 rr as colorless oil. In "stop-flow micro tubing" reactor: following the typical procedure **IV** using a HPFA tubing (total volume 1.5 mL) to afford product **35** and a minor uncharacterized isomeric product (combined 36 mg) in 63% yield and 5:1 rr. ^{1}H NMR (400 MHz, $CDCl_3$) δ 7.66 (dd, *J* = 8.0, 1.3 Hz, 1H), 7.52 (dd, *J* = 7.9, 1.5 Hz, 1H), 7.41 (td, *J* = 7.6, 1.2 Hz, 1H), 7.24 (ddd, *J* = 8.0, 7.5, 1.9 Hz, 1H), 7.16 – 7.07 (m, 2H), 7.03 – 6.94 (m, 2H), 6.37 (s, 1H), 4.28 – 4.20 (m, 1H), 4.09 (d, *J* = 4.7 Hz, 1H), 2.96 (ddd, *J* = 14.0, 7.5, 0.8 Hz, 1H), 2.83 (dd, *J* = 14.0, 8.2 Hz, 1H), 1.86 (d, *J* = 1.2 Hz, 3H); ^{13}C NMR (101 MHz, $CDCl_3$) δ161.5 (d, *J* = 246.4 Hz), 135.9, 133.8, 133.2 (d, *J* = 3.4 Hz), 132.7 (d, *J* = 1.2 Hz), 130.4, 130.3 (d, *J* = 2.9 Hz), 128.9, 128.4, 125.1, 115.1(d, *J* = 21.4 Hz), 111.8, 111.2, 42.2, 27.9, 17.5; HRMS–ESI [M – H]$^-$ Calcd for $C_{20}H_{15}BrFN_2$ 381.0408, found 381.0410.

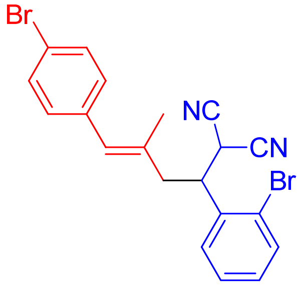

(*E*)-2-(1-(2-bromophenyl)-4-(4-bromophenyl)-3-methylbut-3-en-1-yl)malononitrile (36)

In batch: following the typical procedure **III**, 1-bromo-4-(2-methylprop-1-en-1-yl)benzene (169 mg, 0.8 mmol, 4.0 equiv), 2-(2-bromobenzylidene)malononitrile (47 mg, 0.2 mmol, 1.0 equiv), and 9-mesityl-10-methylacridinium perchlorate **4** (4.1 mg, 0.01 mmol, 0.05 equiv) in DCE (2.0 mL) were employed to give the product **36** and a minor uncharacterized isomeric product (combined 36.4 mg) in 41% yield and 6:1 rr as colorless oil. In "stop-flow micro tubing" reactor: following the typical procedure **IV** using a HPFA tubing (total volume 1.5 mL) to afford product **36** and a minor uncharacterized isomeric product (combined 45 mg) in 68% yield and 6:1 rr. ^{1}H NMR (400 MHz, $CDCl_3$) δ 7.66 (dd, *J* = 8.0, 1.2 Hz, 1H), 7.51 (dd, *J* = 7.9, 1.5 Hz, 1H), 7.45 – 7.40 (m, 3H), 7.27 – 7.22 (m, 1H), 7.00 (d, *J* = 8.4 Hz, 2H), 6.33 (s, 1H), 4.29 – 4.19 (m, 1H), 4.08 (d, *J* = 4.7 Hz, 1H), 2.96 (dd, *J* = 13.7, 6.9 Hz, 1H), 2.83 (dd, *J* = 14.0, 8.3 Hz, 1H), 1.86 (d, *J* = 1.3 Hz, 3H); ^{13}C NMR (101 MHz, $CDCl_3$) δ 136.0, 135.8, 133.8, 133.6, 131.3, 130.4, 128.8, 128.4, 125.1, 120.6, 111.7, 111.2, 42.2, 27.9, 17.6; HRMS–ACPI [M + H]$^+$ Calcd for $C_{20}H_{17}Br_2N_2$ 442.9753, found 442.9746.

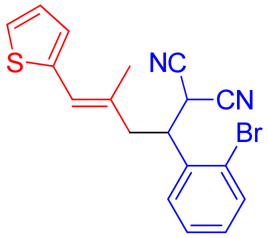

(*E*)-2-(1-(2-bromophenyl)-3-methyl-4-(thiophen-2-yl)but-3-en-1-yl)malononitrile (37)

In batch: following the typical procedure **III**, 2-(2-methylprop-1-en-1-yl)thiophene (111 mg, 0.8 mmol, 4.0 equiv), 2-(2-bromobenzylidene)malononitrile (47 mg, 0.2 mmol, 1.0 equiv), and 9-mesityl-10-methylacridinium perchlorate **4** (4.1 mg, 0.01 mmol, 0.05 equiv) in DCE (2.0 mL) were employed to give the product **37** and a minor uncharacterized isomeric product (combined 11.1 mg) in 15% yield and 5:1 rr as pale yellow oil. In "stop-flow micro tubing" reactor: following the typical procedure **IV** using a HPFA tubing (total volume 1.5 mL) to afford product **37** and a minor uncharacterized isomeric product (combined 23.8 mg) in 43% yield and 5:1 rr. ^{1}H NMR (600 MHz, $CDCl_3$) δ 7.65 (dd, J = 8.0, 1.0 Hz, 1H), 7.53 (d, J = 7.6 Hz, 1H), 7.41 (t, J = 7.6 Hz, 1H), 7.26 – 7.20 (m, 2H), 7.00 (dd, J = 5.0, 3.6 Hz, 1H), 6.94 (d, J = 3.3 Hz, 1H), 6.55 (s, 1H), 4.26 – 4.18 (m, 1H), 4.07 (s, 1H), 2.95 (dd, J = 14.1, 7.7 Hz, 1H), 2.84 (dd, J = 14.0, 7.9 Hz, 1H), 2.02 (s, 3H); ^{13}C NMR (151 MHz, $CDCl_3$) δ 139.9, 135.7, 133.8, 130.9, 130.3, 128.4, 128.2, 127.4, 126.8, 125.2, 123.1, 111.7, 111.2, 42.7, 27.8, 18.2; HRMS–ACPI [M + H]$^+$ Calcd for $C_{18}H_{16}BrN_2S$ 371.0212, found 371.0209.

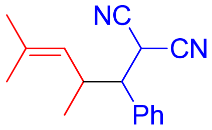

2-(2,4-Dimethyl-1-phenylpent-3-en-1-yl)malononitrile (38)

Following the typical procedure **III**, 2-methylpent-2-ene (99 μL, 0.8 mmol, 4.0 equiv), 2-benzylidenemalononitrile (31 mg, 0.2 mmol, 1.0 equiv), and 9-mesityl-10-methylacridinium perchlorate **4** (2.1 mg, 0.005 mmol, 0.025 equiv) in DCE (4.0 mL) were employed to give the product **38** as inseparable diastereomeric and regioisomeric mixtures (combined 41.9 mg) in 88% yield, 3.9:1 rr, and 1.6:1 dr as colorless oil. Data for the major and minor diastereomers: ^{1}H NMR (600 MHz, $CDCl_3$) δ 7.46 – 7.32 (m, $4H_{major}$), 7.46 – 7.32 (m, $4H_{minor}$), 7.17 (dd, J = 7.8, 1.2 Hz, $1H_{major}$), 7.17 (dd, J = 7.8, 1.2 Hz, $1H_{minor}$), 4.95 (d, J = 10.1 Hz, $1H_{minor}$), 4.78 (d, J = 10.2 Hz, $1H_{major}$), 4.25 (d, J = 4.0 Hz, $1H_{minor}$), 4.10 (d, J = 9.2 Hz, $1H_{major}$), 3.20 (dd, J = 9.2, 6.1 Hz, $1H_{major}$), 3.17 – 3.05 (m, $1H_{major}$), 3.17 – 3.05 (m, $1H_{minor}$), 2.82 (dd, J = 11.2, 4.0 Hz, $1H_{minor}$),

1.82 (dd, J = 15.6, 0.9 Hz, $3H_{major}$), 1.69 – 1.65 (m, $3H_{minor}$), 1.67 (dd, J = 4.2, 1.0 Hz, $3H_{major}$), 1.67 (dd, J = 4.2, 1.0 Hz, $3H_{minor}$), 0.99 (d, J = 6.7 Hz, $3H_{major}$), 0.82 (d, J = 6.5 Hz, $3H_{minor}$); ^{13}C NMR (151 MHz, $CDCl_3$) δ 136.4, 135.9, 135.3, 134.8, 132.5, 129.1, 128.7, 128.6, 128.5, 127.9, 126.6, 123.8, 112.8, 112.5, 112.2, 112.1, 52.9, 52.3, 34.9, 34.4, 28.5, 27.0, 26.1, 26.0, 19.8, 19.3, 18.4, 18.3; HRMS–ESI [M – H]$^-$ Calcd for $C_{16}H_{17}N_2$ 237.1397, found 237.1392.

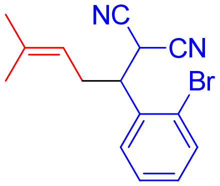

2-(1-(2-Bromophenyl)-4-methylpent-3-en-1-yl)malononitrile (**39**)

Following the typical procedure **III**, 2-methylbut-2-ene (67 μL, 0.8 mmol, 4.0 equiv), 2-(2-bromobenzylidene)malononitrile (47 mg, 0.2 mmol, 1.0 equiv), and 9-mesityl-10-methylacridinium perchlorate **4** (2.1 mg, 0.005 mmol, 0.025 equiv) in DCE (4.0 mL) were employed to give the product **39** as inseparable regioisomeric mixtures (combined 38 mg) in 63% yield and 1.4:1 rr as colorless oil. Data for the major and minor regioisomers: ^1H NMR (600 MHz, $CDCl_3$) δ 7.60 – 7.53 (m, $1H_{major}$), 7.60 – 7.53 (m, $1H_{minor}$), 7.41 (d, J = 8.0 Hz, $1H_{minor}$), 7.39 – 7.35 (m, $1H_{major}$), 7.34 – 7.28 (m, $1H_{major}$), 7.34 – 7.28 (m, $1H_{minor}$), 7.18 – 7.12 (m, $1H_{major}$), 7.18 – 7.12 (m, $1H_{minor}$), 5.41 – 5.33 (m, $1H_{minor}$), 4.92 (td, J = 6.4, 1.3 Hz, $1H_{major}$), 4.07 – 4.00 (m, $1H_{major}$), 4.07 – 4.00 (m, $1H_{minor}$), 3.95 (d, J = 4.3 Hz, $1H_{minor}$), 3.86 (dd, J = 13.1, 7.3 Hz, $1H_{major}$), 2.78 – 2.64 (m, $2H_{major}$), 2.64 – 2.51 (m, $2H_{minor}$), 1.62 (d, J = 5.5 Hz, $3H_{major}$), 1.60 – 1.55 (m, $3H_{minor}$), 1.52 – 1.47 (m, $3H_{major}$), 1.52 – 1.47 (m, $3H_{minor}$); ^{13}C NMR (151 MHz, $CDCl_3$) δ 137.2, 136.2, 135.9, 133.7, 133.6, 130.2, 130.1, 130.0, 128.3, 128.2, 124.6, 124.3, 118.3, 111.9, 111.7, 111.4, 44.6, 41.5, 29.8, 27.7, 27.6, 25.8, 23.1, 18.1, 15.4, 13.6; HRMS–ESI [M – H]$^-$ Calcd for $C_{15}H_{14}BrN_2$ 301.0346, found 301.0343.

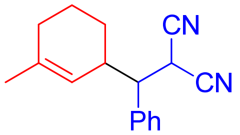

2-((3-Methylcyclohex-2-en-1-yl)(phenyl)methyl)malononitrile (**40**)

Following the typical procedure **III**, 1-methylcyclohex-1-ene (77 mg, 0.8 mmol, 4.0 equiv), 2-benzylidenemalononitrile (31 mg, 0.2 mmol, 1.0 equiv), and 9-mesityl-10-methylacridinium perchlorate **4** (2.1 mg, 0.005 mmol, 0.025 equiv) in DCE (4.0 mL) were employed to give the

product **40** as two separable diastereomers and other trace amount of uncharacterized isomeric products (combined 39.2 mg) in 79% yield, 1.1:1 dr, and >10:1 rr as colorless oil. Data for the major diastereomer: ^{1}H NMR (600 MHz, $CDCl_3$) δ 7.45 – 7.38 (m, 3H), 7.36 (d, *J* = 6.7 Hz, 2H), 4.92 (s, 1H), 4.17 (d, *J* = 4.8 Hz, 1H), 2.94 – 2.83 (m, 2H), 2.01 – 1.95 (m, 1H), 1.90 (s, 2H), 1.87 – 1.81 (m, 1H), 1.67 – 1.57 (m, 2H), 1.56 (s, 3H); ^{13}C NMR (151 MHz, $CDCl_3$) δ 137.6, 136.5, 129.1, 128.8, 128.6, 120.4, 112.0, 111.7, 51.5, 37.3, 29.7, 27.6, 27.5, 23.9, 21.6; HRMS–ESI [M – H]$^-$ Calcd for $C_{17}H_{17}N_2$ 249.1397, found 249.1389.

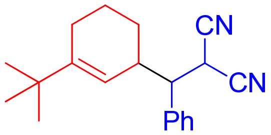

2-((3-(*Tert*-butyl)cyclohex-2-en-1-yl)(phenyl)methyl)malononitrile (**41**)

Following the typical procedure **III**, 1-(*tert*-butyl)cyclohex-1-ene (110 mg, 0.8 mmol, 4.0 equiv), 2-benzylidenemalononitrile (31 mg, 0.2 mmol, 1.0 equiv), and 9-mesityl-10-methylacridinium perchlorate **4** (2.1 mg, 0.005 mmol, 0.025 equiv) in DCE (4.0 mL) were employed to give the product **41** as two separable diastereomers and other trace amount of uncharacterized isomeric products (combined 43.9 mg) in 75% yield, 1.3:1 dr, and >20:1 rr. The major diastereomer is a colorless solid: ^{1}H NMR (600 MHz, $CDCl_3$) δ 7.44 – 7.36 (m, 3H), 7.36 – 7.32 (m, 2H), 5.02 (s, 1H), 4.19 (d, *J* = 5.7 Hz, 1H), 2.95 (dd, *J* = 9.7, 5.7 Hz, 1H), 2.89 (dd, *J* = 7.4, 4.5 Hz, 1H), 2.06 (d, *J* = 17.1 Hz, 1H), 2.00 – 1.93 (m, 1H), 1.93 – 1.78 (m, 2H), 1.55 – 1.48 (m, 1H), 1.28 – 1.20 (m, 1H), 0.90 (s, 9H); ^{13}C NMR (151 MHz, $CDCl_3$) δ 149.2, 136.6, 129.0, 128.7, 128.5, 116.8, 112.1, 111.9, 51.9, 37.7, 35.5, 28.7, 27.7, 27.3, 24.3, 22.1; the minor diastereomer is a colorless oil: ^{1}H NMR (600 MHz, $CDCl_3$) δ 7.43 – 7.37 (m, 3H), 7.37 – 7.32 (m, 2H), 5.57 – 5.52 (m, 1H), 4.22 (d, *J* = 5.0 Hz, 1H), 3.02 (dd, *J* = 10.3, 5.0 Hz, 1H), 2.87 – 2.79 (m, 1H), 2.09 – 1.97 (m, 2H), 1.64 – 1.57 (m, 1H), 1.55 – 1.43 (m, 2H), 1.21 – 1.12 (m, 1H), 1.07 (s, 9H); ^{13}C NMR (151 MHz, $CDCl_3$) δ 151.3, 136.9, 129.1, 128.7, 128.2, 116.0, 112.3, 112.0, 51.7, 36.6, 35.8, 28.9, 27.5, 25.9, 24.6, 20.4; HRMS–ESI [M – H]$^-$ Calcd for $C_{20}H_{23}N_2$ 291.1867, found 291.1870.

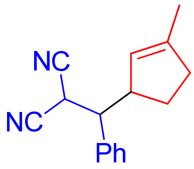

2-((3-Methylcyclopent-2-en-1-yl)(phenyl)methyl)malononitrile (**42**)

Following the typical procedure **III**, 1-methylcyclopent-1-ene (66 mg, 0.8 mmol, 4.0 equiv),

2-benzylidenemalononitrile (31 mg, 0.2 mmol, 1.0 equiv), and 9-mesityl-10-methylacridinium perchlorate **4** (2.1 mg, 0.005 mmol, 0.025 equiv) in DCE (4.0 mL) were employed to give the product **42** as two separable diastereomers and other trace amount of uncharacterized isomeric products (combined 44.4 mg) in 94% yield, 2.9:1 dr, and >10:1 rr. Data for the major diastereomer: ^{1}H NMR (500 MHz, $CDCl_3$) δ 7.45 – 7.33 (m, 5H), 5.45 (s, 1H), 4.11 (d, J = 4.7 Hz, 1H), 3.43 – 3.34 (m, 1H), 2.93 (dd, J = 10.6, 4.7 Hz, 1H), 2.33 – 2.12 (m, 2H), 2.01 – 1.90 (m, 1H), 1.78 (s, 3H), 1.48 – 1.38 (m, 1H); ^{13}C NMR (126 MHz, $CDCl_3$) δ 146.0, 136.9, 129.0, 128.7, 128.4, 123.3, 112.1, 111.8, 51.8, 47.6, 35.6, 29.4, 28.3, 16.8; HRMS–APCI [M + H]$^+$ Calcd for $C_{16}H_{17}N_2$ 237.1386, found 237.1383.

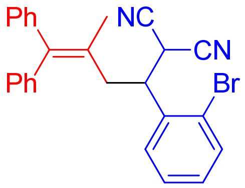

2-(1-(2-Bromophenyl)-3-methyl-4,4-diphenylbut-3-en-1-yl)malononitrile (**43**)

In batch: following the typical procedure **III**, (2-methylprop-1-ene-1,1-diyl)dibenzene (166 mg, 0.8 mmol, 4.0 equiv), 2-(2-bromobenzylidene)malononitrile (47 mg, 0.2 mmol, 1.0 equiv), and 9-mesityl-10-methylacridinium perchlorate **4** (4.1 mg, 0.01 mmol, 0.05 equiv) in DCE (2.0 mL) were employed to give the product **43** (26 mg) in 30% yield as a colorless oil. In "stop-flow micro tubing" reactor: following the typical procedure **IV** using a HPFA tubing (total volume 1.5 mL) to afford product **43** (47.5 mg) in 72% yield. ^{1}H NMR (400 MHz, $CDCl_3$) δ 7.69 – 7.61 (m, 1H), 7.36 – 7.30 (m, 2H), 7.30 – 7.26 (m, 1H), 7.25 – 7.13 (m, 5H), 7.03 (d, J = 6.9 Hz, 2H), 6.96 – 6.89 (m, 1H), 6.88 – 6.83 (m, 2H), 4.18 (br s, 1H), 3.88 (br s, 1H), 3.09 (dd, J = 13.7, 10.5 Hz, 1H), 2.91 (dd, J = 13.7, 5.2 Hz, 1H), 1.73 (s, 3H); ^{13}C NMR (101 MHz, $CDCl_3$) δ 142.4, 142.2, 141.8, 135.1, 133.5, 130.2, 129.6, 129.3, 128.6, 128.4, 128.1, 127.9, 127.0, 126.6, 111.6, 111.1, 36.6, 28.5, 20.1; HRMS–APCI [M + H]$^+$ Calcd for $C_{26}H_{22}BrN_2$ 441.0961, found 441.0953.

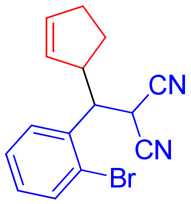

2-((2-Bromophenyl)(cyclopent-2-en-1-yl)methyl)malononitrile (**44**)

Following the typical procedure **III**, cyclopentene (68 mg, 1.0 mmol, 5.0 equiv), 2-(2-bromobenzylidene)malononitrile (47 mg, 0.2 mmol, 1.0 equiv), and

9-mesityl-10-methylacridinium perchlorate **4** (1.0 mg, 0.0025 mmol, 0.0125 equiv) in DCE (2.0 mL) were employed to give the product **44** as inseparable diastereomers (41 mg) in 68% yield and 1.3:1 dr as colorless oil. ^{1}H NMR (400 MHz, $CDCl_3$) δ 7.66 (dd, J = 8.1, 1.1 Hz, 1H_{major}), 7.66 (dd, J = 8.1, 1.1 Hz, 1H_{minor}), 7.57 (ddd, J = 18.2, 7.9, 1.6 Hz, 1H_{major}), 7.57 (ddd, J = 18.2, 7.9, 1.6 Hz, 1H_{minor}), 7.40 (dd, J = 10.9, 4.2 Hz, 1H_{major}), 7.40 (dd, J = 10.9, 4.2 Hz, 1H_{minor}), 7.26 – 7.21 (m, 1H_{major}), 7.26 – 7.21 (m, 1H_{minor}), 6.10 – 6.02 (m, 1H_{minor}), 5.99 – 5.92 (m, 1H_{minor}), 5.88 – 5.81 (m, 1H_{major}), 5.32 – 5.24 (m, 1H_{major}), 4.13 – 4.02 (m, 1H_{major}), 4.13 – 4.02 (m, 1H_{minor}), 3.91 – 3.74 (m, 1H_{major}), 3.91 – 3.74 (m, 1H_{minor}), 3.57 – 3.36 (m, 1H_{major}), 3.57 – 3.36 (m, 1H_{minor}), 2.48 – 2.38 (m, 3H_{major}), 2.37 – 2.30 (m, 2H_{minor}), 1.96 – 1.83 (m, 1H_{minor}), 1.77 – 1.64 (m, 1H_{major}), 1.45 – 1.34 (m, 1H_{minor}); ^{13}C NMR (101 MHz, $CDCl_3$) δ 136.8, 136.5, 135.9, 134.1, 133.7, 133.6, 130.1, 130.06, 130.05, 129.2, 128.5, 128.3, 128.1, 125.9, 125.7, 111.9, 111.8, 111.3, 111.2, 48.5, 48.3, 48.2, 48.0, 32.5, 31.3, 28.9, 28.7, 27.4, 27.3; HRMS–APCI $[M + H]^+$ Calcd for $C_{15}H_{14}BrN_2$ 301.0335, found 301.0328.

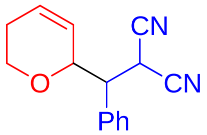

2-((5,6-Dihydro-2*H*-pyran-2-yl)(phenyl)methyl)malononitrile (**45**)

Following the typical procedure **III**, 3,4-dihydro-2*H*-pyran (84 mg, 1.0 mmol, 5.0 equiv), 2-benzylidenemalononitrile (31 mg, 0.2 mmol, 1.0 equiv), and 9-mesityl-10-methylacridinium perchlorate **4** (1.0 mg, 0.0025 mmol, 0.0125 equiv) in DCE (2.0 mL) were employed to give the product **45** as two separable diastereomers and other minor uncharacterized isomeric products (combined 36.9 mg) in 78% yield, 2.5:1 dr, and 3:1 rr as colorless oil. Data for the major diastereomer: ^{1}H NMR (400 MHz, $CDCl_3$) δ 7.49 – 7.36 (m, 5H), 5.94 – 5.85 (m, 1H), 5.38 – 5.31 (m, 1H), 4.62 (d, J = 4.3 Hz, 1H), 4.61 – 4.57 (m, 1H), 4.08 (ddd, J = 11.3, 5.9, 1.4 Hz, 1H), 3.76 (ddd, J = 11.3, 10.1, 3.8 Hz, 1H), 3.14 (dd, J = 10.4, 4.3 Hz, 1H), 2.40 – 2.27 (m, 1H), 2.05 – 1.94 (m, 1H); ^{13}C NMR (101 MHz, $CDCl_3$) δ 133.9, 129.4, 129.3, 128.8, 127.5, 125.8, 112.3, 111.7, 72.8, 63.8, 51.1, 26.3, 24.9; HRMS–ACPI $[M + H]^+$ Calcd for $C_{15}H_{15}N_2O$ 239.1179, found 239.1181.

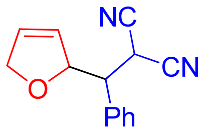

2-((2,5-Dihydrofuran-2-yl)(phenyl)methyl)malononitrile (**46**)

In batch: following the typical procedure **III**, 2,5-dihydrofuran (70 mg, 1.0 mmol, 5.0 equiv), 2-benzylidenemalononitrile (31 mg, 0.2 mmol, 1.0 equiv), and 9-mesityl-10-methylacridinium perchlorate **4** (1.0 mg, 0.0025 mmol, 0.0125 equiv) in DCE (2.0 mL) were employed to give the product **46** as inseparable diastereomers and other trace amount of uncharacterized isomeric products (combined 19.7 mg) in 44% yield, 1.6:1 dr, and >10:1 rr as colorless oil. In "stop-flow micro tubing" reactor: following the typical procedure **IV** using a HPFA tubing (total volume 1.5 mL) to afford product **46** as inseparable diastereomers and other trace amount of uncharacterized isomeric products (combined 31.3 mg) in 93% yield, 1.6:1 dr, and >10:1 rr. ^{1}H NMR (400 MHz, $CDCl_3$) δ 7.47 – 7.40 (m, 5H_{major}), 7.38 – 7.33 (m, 3H_{minor}), 7.26 – 7.23 (m, 2H_{minor}), 6.01 (ddd, *J* = 6.2, 3.7, 1.7 Hz, 1H_{major}), 5.82 (ddd, *J* = 6.2, 3.9, 1.6 Hz, 1H_{minor}), 5.66 (dtd, *J* = 6.3, 2.5, 1.5 Hz, 1H_{minor}), 5.54 (dtd, *J* = 6.3, 2.5, 1.5 Hz, 1H_{major}), 5.43 – 5.36 (m, 1H_{minor}), 5.35 – 5.26 (m, 1H_{major}), 4.79 (dddd, *J* = 13.3, 5.7, 2.4, 1.7 Hz, 1H_{major}), 4.73 (dddd, *J* = 13.3, 4.0, 2.5, 1.7 Hz, 1H_{major}), 4.62 (dddd, *J* = 13.1, 4.2, 2.5, 1.6 Hz, 1H_{minor}), 4.55 – 4.47 (m, 1H_{minor}), 4.51 (d, *J* = 4.4 Hz, 1H_{major}); 4.38 (d, *J* = 10.3 Hz, 1H_{minor}), 3.46 (dd, *J* = 10.3, 2.9 Hz, 1H_{minor}), 3.10 (dd, *J* = 9.4, 4.4 Hz, 1H_{major}); ^{13}C NMR (101 MHz, $CDCl_3$) δ 134.1, 133.9, 129.4, 129.3, 129.2, 129.1, 128.9, 128.8, 128.4, 126.7, 125.7, 112.3, 112.2, 112.1, 111.7, 85.6, 85.5, 76.2, 75.9, 52.3, 51.1, 26.5, 25.6; HRMS–APCI $[M + H]^+$ Calcd for $C_{14}H_{13}N_2O$ 225.1022, found 225.1023.

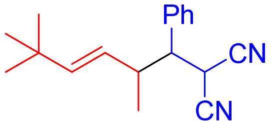

(*E*)-2-(2,5,5-trimethyl-1-phenylhex-3-en-1-yl)malononitrile (**47**)

In batch: following the typical procedure **III**, (*E*)-2,2-dimethylhex-3-ene (112 mg, 1.0 mmol, 5.0 equiv), 2-benzylidenemalononitrile (31 mg, 0.2 mmol, 1.0 equiv), and 9-mesityl-10-methylacridinium perchlorate **4** (1.0 mg, 0.0025 mmol, 0.0125 equiv) in DCE (2.0 mL) were employed to give the product **47** as inseparable diastereomers and other trace amount of uncharacterized isomeric products (combined 13.6 mg) in 25% yield, 1:1 dr, and >20:1 rr as colorless oil. In "stop-flow micro tubing" reactor: following the typical procedure **IV** using a HPFA tubing (total volume 1.5 mL) to afford product **47** as inseparable diastereomers and other trace amount of uncharacterized isomeric products (combined 30 mg) in 75% yield, 1:1 dr, and >20:1 rr. ^{1}H NMR (400 MHz, $CDCl_3$) δ 7.46 – 7.33 (m, 4H_{major}), 7.46 – 7.33 (m, 4H_{minor}),

7.15 – 7.10 (m, 1H$_{major}$), 7.15 – 7.10 (m, 1H$_{minor}$), 5.90 (d, J = 15.5 Hz, 1H), 5.62 (dd, J = 15.7, 0.6 Hz, 1H), 5.17 (dd, J = 15.5, 9.0 Hz, 1H), 4.94 (dd, J = 15.7, 9.3 Hz, 1H), 4.11 (d, J = 3.8 Hz, 1H), 4.05 (d, J = 9.8, 1H), 3.17 (dd, J = 9.8, 5.7 Hz, 1H), 2.90 – 2.75 (m, 3H), 1.07 (s, 9H), 1.02 (d, J = 6.8 Hz, 3H), 0.96 (s, 9H), 0.87 (d, J = 6.1 Hz, 3H); ^{13}C NMR (126 MHz, $CDCl_3$) δ 145.9, 145.4, 136.0, 134.6, 129.2, 128.9, 128.7, 128.6, 128.4, 125.9, 123.5, 112.4, 112.2, 111.7, 51.9, 40.1, 38.9, 33.2, 33.1, 29.5, 29.4, 28.7, 27.2, 19.6, 19.4; HRMS–APCI [M + H]$^+$ Calcd for $C_{18}H_{23}N_2$ 267.1856, found 267.1852.

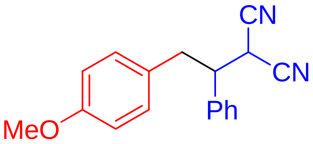

2-(2-(4-Methoxyphenyl)-1-phenylethyl)malononitrile (**48**)

Following the typical procedure **V**, 4-Methylanisole (122 mg, 1.0 mmol, 5.0 equiv), 2-benzylidenemalononitrile (31 mg, 0.2 mmol, 1.0 equiv), and 9-mesityl-10-methylacridinium perchlorate **4** (1.0 mg, 0.0025 mmol, 0.0125 equiv) in DCE (2.0 mL) were employed to give the product **48** (47 mg) in 85% yield as a pale yellow oil. ^{1}H NMR (600 MHz, $CDCl_3$) δ 7.46 – 7.35 (m, 5H), 7.10 (d, J = 8.6 Hz, 2H), 6.86 (d, J = 8.6 Hz, 2H), 3.86 (d, J = 5.2 Hz, 1H), 3.79 (s, 3H), 3.44 – 3.39 (m, 1H), 3.25 – 3.17 (m, 2H); ^{13}C NMR (151 MHz, $CDCl_3$) δ 158.9, 136.5, 129.9, 129.1, 128.9, 128.4, 127.9, 114.5, 112.1, 111.4, 55.2, 48.5, 37.6, 28.3; HRMS–ESI [M – H]$^-$ Calcd for $C_{18}H_{15}N_2O$ 275.1190, found 275.1193.

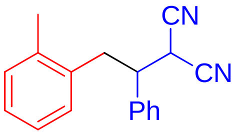

2-(1-Phenyl-2-*o*-tolylethyl)malononitrile (49)

Following the typical procedure **V**, o-xylene (106 mg, 1.0 mmol, 5.0 equiv), 2-benzylidenemalononitrile (31 mg, 0.2 mmol, 1.0 equiv), and 9-mesityl-10-methylacridinium perchlorate **4** (1.0 mg, 0.0025 mmol, 0.0125 equiv) in DCE (2.0 mL) were employed to give the product **49** (42 mg) in 80% yield as a colorless oil. ^{1}H NMR (400 MHz, $CDCl_3$) δ 7.47 – 7.35 (m, 5H), 7.22 – 7.18 (m, 2H), 7.18 – 7.11 (m, 1H), 7.08 (d, J = 7.1 Hz, 1H), 3.92 (d, J = 5.0 Hz, 1H), 3.43 (ddd, J = 8.8, 6.3, 5.1 Hz, 1H), 3.33 (dd, J = 14.1, 8.8 Hz, 1H), 3.23 (dd, J = 14.1, 6.4 Hz, 1H), 2.33 (s, 3H); ^{13}C NMR (101 MHz, $CDCl_3$) δ 136.7, 136.4, 134.8, 131.2, 129.6, 129.2, 129.1, 127.9, 127.7, 126.5, 112.1, 111.6, 46.9, 36.1, 28.5, 19.3; HRMS–APCI [M + H]$^+$ Calcd for

$C_{18}H_{17}N_2$ 261.1386, found 261.1387.

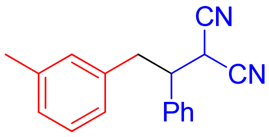

2-(1-Phenyl-2-*m*-tolylethyl)malononitrile (50)

Following the typical procedure **V**, m-xylene (106 mg, 1.0 mmol, 5.0 equiv), 2-benzylidenemalononitrile (31 mg, 0.2 mmol, 1.0 equiv), and 9-mesityl-10-methylacridinium perchlorate **4** (1.0 mg, 0.0025 mmol, 0.0125 equiv) in DCE (2.0 mL) were employed to give the product **50** (26 mg) in 50% yield as a colorless oil. ^{1}H NMR (300 MHz, $CDCl_3$) δ 7.47 – 7.37 (m, 5H), 7.23 (t, J = 7.5 Hz, 1H), 7.10 (d, J = 7.7 Hz, 1H), 7.00 (d, J = 9.0 Hz, 2H), 3.85 (d, J = 4.9 Hz, 1H), 3.45 (td, J = 7.8, 4.9 Hz, 1H), 3.22 (d, J = 7.9 Hz, 2H), 2.34 (s, 3H); ^{13}C NMR (75 MHz, $CDCl_3$) δ 138.9, 136.6, 129.6, 129.2, 129.0, 128.4, 128.0, 125.9, 112.1, 111.4, 48.3, 38.4, 28.4, 21.4; HRMS–APCI $[M + H]^+$ Calcd for $C_{18}H_{17}N_2$ 261.1386, found 261.1383.

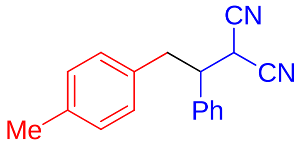

2-(1-Phenyl-2-*p*-tolylethyl)malononitrile (51)

Following the typical procedure **V**, *p*-xylene (106 mg, 1.0 mmol, 5.0 equiv), 2-benzylidenemalononitrile (31 mg, 0.2 mmol, 1.0 equiv), and 9-mesityl-10-methylacridinium perchlorate **4** (1.0 mg, 0.0025 mmol, 0.0125 equiv) in DCE (2.0 mL) were employed to give the product **51** (47 mg) in 89% yield as a colorless oil. ^{1}H NMR (400 MHz, $CDCl_3$) δ 7.48 – 7.35 (m, 5H), 7.14 (d, J = 7.9 Hz, 2H), 7.09 (d, J = 8.1 Hz, 2H), 3.85 (d, J = 5.0 Hz, 1H), 3.48 – 3.38 (m, 1H), 3.23 (d, J = 8.0 Hz, 2H), 2.34 (s, 3H); ^{13}C NMR (101 MHz, $CDCl_3$) δ 137.3, 136.5, 133.5, 129.8, 129.1, 129.0, 128.8, 128.0, 112.1, 111.4, 48.4, 38.1, 28.4, 21.0; HRMS–APCI $[M + H]^+$ Calcd for $C_{18}H_{17}N_2$ 261.1386, found 261.1385.

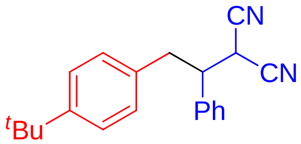

2-(2-(4-*Tert*-butylphenyl)-1-phenylethyl)malononitrile (52)

Following the typical procedure **V**, 1-*tert*-butyl-4-methylbenzene (148 mg, 1.0 mmol, 5.0 equiv), 2-benzylidenemalononitrile (31 mg, 0.2 mmol, 1.0 equiv), and 9-mesityl-10-methylacridinium perchlorate **4** (1.0 mg, 0.0025 mmol, 0.0125 equiv) in DCE (2.0 mL) were employed to give the

product **52** (50 mg) in 83% yield as a colorless oil. ^{1}H NMR (400 MHz, $CDCl_3$) δ 7.46 – 7.40 (m, 5H), 7.37 (d, *J* = 8.4 Hz, 2H), 7.15 (d, *J* = 8.4 Hz, 2H), 3.86 (d, *J* = 4.8 Hz, 1H), 3.48 – 3.40 (m, 1H), 3.23 (d, *J* = 7.9 Hz, 2H), 1.32 (s, 9H); ^{13}C NMR (101 MHz, $CDCl_3$) δ 150.7, 136.6, 133.5, 129.2, 129.0, 128.6, 128.1, 126.1, 112.2, 111.4, 48.3, 37.9, 34.5, 31.3, 28.4; HRMS–APCI [M + H]$^+$ Calcd for $C_{21}H_{23}N_2$ 303.1856, found 303.1862.

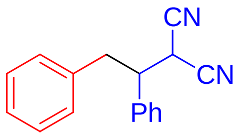

2-(1,2-Diphenylethyl)malononitrile (53)

Following the typical procedure **V**, toluene (92 mg, 1.0 mmol, 5.0 equiv), 2-benzylidenemalononitrile (31 mg, 0.2 mmol, 1.0 equiv), and 9-mesityl-10-methylacridinium perchlorate **4** (1.0 mg, 0.0025 mmol, 0.0125 equiv) in DCE (2.0 mL) were employed to give the product **53** (22.7 mg) in 46% yield as a colorless oil. In "stop-flow micro tubing" reactor: following the typical procedure **VI** using a HPFA tubing (total volume 1.5 mL) to afford product **53** (31.5 mg) in 85% yield. ^{1}H NMR (300 MHz, $CDCl_3$) δ 7.51 – 7.41 (m, 5H), 7.41 – 7.31 (m, 3H), 7.28 – 7.20 (m, 2H), 3.89 (d, *J* = 5.0 Hz, 1H), 3.50 (td, *J* = 7.8, 5.1 Hz, 1H), 3.36 – 3.27 (m, 2H); ^{13}C NMR (101 MHz, $CDCl_3$) δ 136.6, 136.4, 129.2, 129.1, 129.0, 128.9, 128.0, 127.6, 112.0, 111.4, 48.3, 38.5, 28.5; HRMS–APCI [M + H]$^+$ Calcd for $C_{17}H_{15}N_2$ 247.1230, found 247.1226.

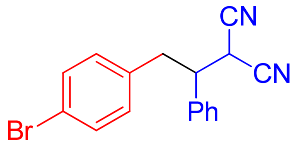

2-(2-(4-Bromophenyl)-1-phenylethyl)malononitrile (54)

Following the typical procedure **V**, 1-bromo-4-methylbenzene (171 mg, 1.0 mmol, 5.0 equiv), 2-benzylidenemalononitrile (31 mg, 0.2 mmol, 1.0 equiv), and 9-mesityl-10-methylacridinium perchlorate **4** (1.0 mg, 0.0025 mmol, 0.0125 equiv) in DCE (2.0 mL) were employed to give the product **54** (8 mg) in 12% yield as a colorless oil. In "stop-flow micro tubing" reactor: following the typical procedure **VI** using a HPFA tubing (total volume 1.5 mL) to afford product **54** (31 mg) in 64% yield. ^{1}H NMR (400 MHz, $CDCl_3$) δ 7.46 – 7.37 (m, 5H), 7.36 – 7.31 (m, 2H), 7.02 (d, *J* = 8.4 Hz, 2H), 3.86 (d, *J* = 5.5 Hz, 1H), 3.43 (td, *J* = 7.8, 5.5 Hz, 1H), 3.24 (qd, *J* = 14.1, 7.8 Hz, 2H); ^{13}C NMR (75 MHz, $CDCl_3$) δ 135.9, 135.5, 132.2, 130.6, 129.3, 129.2, 127.9, 121.5, 111.8, 111.4, 48.2, 37.9, 28.8; HRMS–APCI [M + H]$^+$ Calcd for $C_{17}H_{14}BrN_2$ 325.0335, found 325.0327.

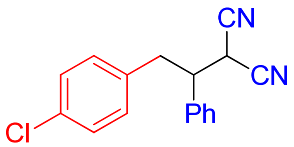

2-(2-(4-Chlorophenyl)-1-phenylethyl)malononitrile (55)

Following the typical procedure **V**, 1-chloro-4-methylbenzene (127 mg, 1.0 mmol, 5.0 equiv), 2-benzylidenemalononitrile (31 mg, 0.2 mmol, 1.0 equiv), and 9-mesityl-10-methylacridinium perchlorate **4** (1.0 mg, 0.0025 mmol, 0.0125 equiv) in DCE (2.0 mL) were employed to give the product **55** (6 mg) in 10% yield as a colorless oil. In "stop-flow micro tubing" reactor: following the typical procedure **VI** using a HPFA tubing (total volume 1.5 mL) to afford product **55** (15.5 mg) in 37% yield. ^{1}H NMR (300 MHz, $CDCl_3$) δ 7.45 – 7.31 (m, 5H), 7.27 (d, J = 7.5 Hz, 2H), 7.08 (d, J = 7.6 Hz, 2H), 3.87 (d, J = 5.1 Hz, 1H), 3.44 (dd, J = 13.8, 7.1 Hz, 1H), 3.35 – 3.14 (m, 2H); ^{13}C NMR (75 MHz, $CDCl_3$) δ 135.9, 135.0, 133.4, 130.3, 129.3, 129.2, 127.9, 111.8, 111.4, 48.2, 37.9, 28.8; HRMS–APCI [M + H]$^+$ Calcd for $C_{17}H_{14}ClN_2$ 281.0840, found 281.0837.

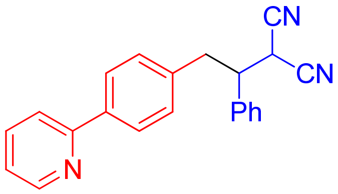

2-(1-Phenyl-2-(4-(pyridin-2-yl)phenyl)ethyl)malononitrile (**56**)

Following the typical procedure **V**, 2-(*p*-tolyl)pyridine (169 mg, 1.0 mmol, 5.0 equiv), 2-benzylidenemalononitrile (31 mg, 0.2 mmol, 1.0 equiv), and 9-mesityl-10-methylacridinium perchlorate **4** (1.0 mg, 0.0025 mmol, 0.0125 equiv) in DCE (2.0 mL) were employed to give the product **56** (47 mg) in 73% yield as a pale yellow oil. ^{1}H NMR (500 MHz, $CDCl_3$) δ 8.70 (d, J = 4.8 Hz, 1H), 7.96 (d, J = 8.2 Hz, 2H), 7.76 (td, J = 7.8, 1.7 Hz, 1H), 7.71 (d, J = 7.9 Hz, 1H), 7.47 – 7.35 (m, 5H), 7.28 (d, J = 8.2 Hz, 2H), 7.25 (ddd, J = 7.0, 4.8, 0.9 Hz, 1H), 3.98 (d, J = 5.5 Hz, 1H), 3.53 (td, J = 7.8, 5.6 Hz, 1H), 3.33 (ddd, J = 28.9, 14.0, 7.9 Hz, 2H); ^{13}C NMR (126 MHz, $CDCl_3$) δ 156.5, 149.5, 138.4, 137.3, 136.8, 136.1, 129.2, 129.0, 128.9, 127.9, 127.4, 122.2, 120.3, 111.9, 111.5, 47.9, 38.1, 28.5; HRMS–ESI [M + Na]$^+$ Calcd for $C_{22}H_{17}N_3Na$ 346.1315, found 346.1315.

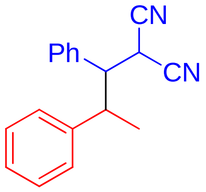

2-(1,2-Diphenylpropyl)malononitrile (57)

Following the typical procedure **V**, ethylbenzene (106 mg, 1.0 mmol, 5.0 equiv), 2-benzylidenemalononitrile (31 mg, 0.2 mmol, 1.0 equiv), and 9-mesityl-10-methylacridinium perchlorate **4** (1.0 mg, 0.0025 mmol, 0.0125 equiv) in DCE (2.0 mL) were employed to give the product **57** as two separable diastereomers (combined 38 mg) in 73% yield and 1.2:1 dr as colorless oil. Data for the major diastereomer: ^{1}H NMR (500 MHz, $CDCl_3$) δ 7.56 – 7.41 (m, 7H), 7.41 – 7.33 (m, 3H), 3.61 (d, J = 4.1 Hz, 1H), 3.41 (dq, J = 13.5, 6.8 Hz, 1H), 3.21 (dd, J = 11.6, 4.0 Hz, 1H), 1.15 (d, J = 6.8 Hz, 3H); ^{13}C NMR (126 MHz, $CDCl_3$) δ 142.4, 135.5, 129.7, 129.3, 129.1, 128.5, 128.1, 127.1, 112.1, 111.4, 53.6, 42.0, 28.7, 20.6; HRMS–APCI $[M + H]^+$ Calcd for $C_{18}H_{17}N_2$ 261.1386, found 261.1388.

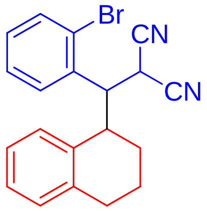

2-((2-Bromophenyl)(1,2,3,4-tetrahydronaphthalen-1-yl)methyl)malononitrile (58)

Following the typical procedure **V**, 1,2,3,4-tetrahydronaphthalene (53 mg, 0.4 mmol, 2.0 equiv), 2-(2-bromobenzylidene)malononitrile (47 mg, 0.2 mmol, 1.0 equiv), and 9-mesityl-10-methylacridinium perchlorate **4** (1.0 mg, 0.0025 mmol, 0.0125 equiv) in DCE (2.0 mL) were employed to give the product **58** as two separable diastereomers (combined 55 mg) in 75% yield and 2:1 dr as colorless oil. The major diastereomer: ^{1}H NMR (300 MHz, $CDCl_3$) δ 7.59 – 7.45 (m, 2H), 7.38 (t, J = 7.5 Hz, 1H), 7.20 (ddd, J = 8.2, 7.4, 1.1 Hz, 1H), 7.16 – 7.04 (m, 2H), 6.91 (t, J = 7.3 Hz, 1H), 6.57 (d, J = 7.8 Hz, 1H), 4.46 – 4.34 (m, 1H), 4.20 (d, J = 6.7 Hz, 1H), 3.60 – 3.45 (m, 1H), 2.81 (t, J = 6.8 Hz, 2H), 2.16 – 1.93 (m, 2H), 1.92 – 1.74 (m, 2H); ^{13}C NMR (75 MHz, $CDCl_3$) δ 137.9, 136.4, 134.9, 133.5, 129.9, 129.5, 128.6, 128.2, 128.0, 127.1, 126.5, 125.4, 112.2, 111.6, 46.9, 40.6, 28.3, 25.9, 25.3, 18.9; the minor diastereomer: ^{1}H NMR (300 MHz, $CDCl_3$) δ 7.73 (dd, J = 11.5, 8.0 Hz, 2H), 7.50 (t, J = 7.5 Hz, 1H), 7.39 – 7.18 (m, 5H), 4.07 (dd, J = 11.6, 4.1 Hz, 1H), 3.97 (d, J = 4.1 Hz, 1H), 3.44 (d, J = 11.7 Hz, 1H), 3.02 (ddd, J = 17.7, 8.5, 3.5 Hz, 1H), 2.93 – 2.76 (m, 1H), 2.06 – 1.86 (m, 1H), 1.80 – 1.46 (m, 3H); ^{13}C NMR (75 MHz, $CDCl_3$) δ 137.5, 136.3, 135.3, 133.9, 130.5, 130.3, 128.9, 128.6, 128.5, 128.1, 126.5, 126.1, 112.1, 111.4, 46.8, 40.7, 28.0, 27.0, 24.9, 17.3; HRMS–APCI $[M + H]^+$ Calcd for $C_{20}H_{18}BrN_2$ 365.0648,

found 365.0645.

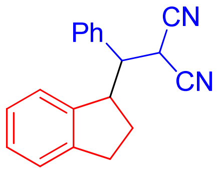

2-((2,3-Dihydro-1H-inden-1-yl)(phenyl)methyl)malononitrile (59)

Following the typical procedure **V**, 2,3-dihydro-1*H*-indene (118 mg, 1.0 mmol, 5.0 equiv), 2-benzylidenemalononitrile (31 mg, 0.2 mmol, 1.0 equiv), and 9-mesityl-10-methylacridinium perchlorate **4** (1.0 mg, 0.0025 mmol, 0.0125 equiv) in DCE (2.0 mL) were employed to give the product **59** as two separable diastereomers (combined 54 mg) in 99% yield and 2:1 dr as colorless oil. The major diastereomer: ^{1}H NMR (400 MHz, $CDCl_3$) δ 7.45 – 7.33 (m, 3H), 7.23 – 7.12 (m, 4H), 7.05 – 6.95 (m, 1H), 6.57 (d, *J* = 7.6 Hz, 1H), 4.20 (d, *J* = 6.9 Hz, 1H), 3.96 (dd, *J* = 14.1, 8.0 Hz, 1H), 3.36 (dd, *J* = 8.7, 6.9 Hz, 1H), 2.83 – 2.71 (m, 1H), 2.56 – 2.42 (m, 2H), 2.00 – 1.86 (m, 1H); ^{13}C NMR (101 MHz, $CDCl_3$) δ 144.8, 141.8, 135.9, 129.1, 129.0, 128.6, 127.7, 126.3, 124.9, 124.5, 112.1, 111.8, 51.1, 46.2, 31.3, 30.9, 28.4; the minor diastereomer: ^{1}H NMR (400 MHz, $CDCl_3$) δ 7.48 – 7.38 (m, 6H), 7.33 – 7.26 (m, 3H), 4.18 (d, *J* = 4.7 Hz, 1H), 3.80 (dd, *J* = 10.8, 7.8 Hz, 1H), 3.06 (dd, *J* = 11.0, 4.7 Hz, 1H), 2.99 – 2.86 (m, 1H), 2.78 (dd, *J* = 16.2, 8.1 Hz, 1H), 2.25 – 2.11 (m, 1H), 1.82 – 1.71 (m, 1H); ^{13}C NMR (75 MHz, $CDCl_3$) δ 144.5, 142.9, 136.3, 129.2, 128.9, 128.6, 128.3, 126.8, 125.8, 124.5, 112.1, 111.9, 49.3, 46.8, 30.5, 30.1, 28.4; HRMS–APCI $[M + H]^+$ Calcd for $C_{19}H_{17}N_2$ 273.1386, found 273.1387.

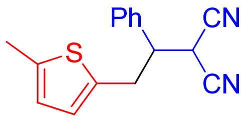

2-(2-(5-Methylthiophen-2-yl)-1-phenylethyl)malononitrile (60)

Following the typical procedure **V**, 2,5-dimethylthiophene (112 mg, 1.0 mmol, 5.0 equiv), 2-benzylidenemalononitrile (31 mg, 0.2 mmol, 1.0 equiv), and 9-mesityl-10-methylacridinium perchlorate **4** (1.0 mg, 0.0025 mmol, 0.0125 equiv) in DCE (2.0 mL) were employed to give the product **60** (28.2 mg) in 53% yield as a pale yellow oil. In "stop-flow micro tubing" reactor: following the typical procedure **IV** using a HPFA tubing (total volume 1.5 mL) to afford product **60** (24.4 mg) in 61% yield. ^{1}H NMR (500 MHz, $CDCl_3$) δ 7.47 – 7.37 (m, 5H), 6.69 (d, *J* = 3.3 Hz, 1H), 6.58 (dd, *J* = 3.2, 0.9 Hz, 1H), 4.01 (d, *J* = 4.7 Hz, 1H), 3.49 – 3.40 (m, 2H), 3.40 – 3.31 (m, 1H), 2.44 (s, 3H); ^{13}C NMR (126 MHz, $CDCl_3$) δ 140.0, 136.1, 135.9, 129.2, 129.1, 128.0, 127.0,

125.3, 111.9, 111.2, 48.4, 32.9, 28.4, 15.3; HRMS–APCI [M + H]$^+$ Calcd for $C_{16}H_{15}N_2S$ 267.0950, found 267.0954.

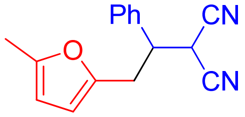

2-(2-(5-Methylfuran-2-yl)-1-phenylethyl)malononitrile (61)

Following the typical procedure **VI** using a HPFA tubing (total volume 1.5 mL) to afford product **61** (29 mg) in 77% yield as a pale yellow oil. ^{1}H NMR (500 MHz, $CDCl_3$) δ 7.47 – 7.36 (m, 5H), 6.05 (d, *J* = 2.9 Hz, 1H), 5.92 – 5.85 (m, 1H), 4.00 (d, *J* = 4.9 Hz, 1H), 3.61 (dt, *J* = 10.2, 5.2 Hz, 1H), 3.28 (dd, *J* = 15.3, 10.0 Hz, 1H), 3.19 (dd, *J* = 15.3, 5.5 Hz, 1H), 2.28 (s, 3H); ^{13}C NMR (126 MHz, $CDCl_3$) δ 142.4, 135.5, 129.7, 129.3, 129.1, 128.5, 128.1, 127.1, 112.1, 111.4, 53.6, 42.0, 28.7, 20.6; HRMS–APCI [M + H]$^+$ Calcd for $C_{16}H_{15}N_2O$ 251.1179, found 251.1184.

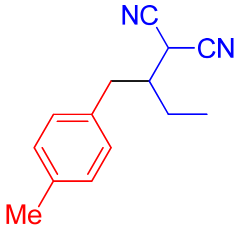

2-(1-*p*-Tolylbutan-2-yl)malononitrile (62)

Following the typical procedure **V**, *p*-xylene (106 mg, 1.0 mmol, 5.0 equiv), 2-propylidenemalononitrile (21 mg, 0.2 mmol, 1.0 equiv), and 9-mesityl-10-methylacridinium perchlorate **4** (1.0 mg, 0.0025 mmol, 0.0125 equiv) in DCE (2.0 mL) were employed to give the product **62** (33 mg) in 78% yield as a colorless oil. ^{1}H NMR (400 MHz, $CDCl_3$) δ 7.16 (d, *J* = 7.9 Hz, 2H), 7.07 (d, *J* = 8.0 Hz, 2H), 3.61 (d, *J* = 4.1 Hz, 1H), 3.00 (dd, *J* = 14.2, 5.7 Hz, 1H), 2.60 (dd, *J* = 14.2, 9.5 Hz, 1H), 2.35 (s, 3H), 2.23 – 2.10 (m, 1H), 1.91 – 1.77 (m, 1H), 1.74 – 1.59 (m, 1H), 1.12 (t, *J* = 7.4 Hz, 3H); ^{13}C NMR (126 MHz, $CDCl_3$) δ 137.1, 133.8, 129.8, 128.7, 112.6, 111.5, 44.4, 36.6, 26.3, 24.3, 21.0, 11.3; HRMS–APCI [M + H]$^+$ Calcd for $C_{14}H_{17}N_2$ 213.1386, found 213.1384.

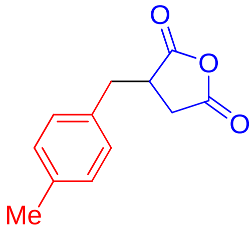

3-(4-Methylbenzyl)dihydrofuran-2,5-dione (63)

Following the typical procedure **V**, *p*-xylene (106 mg, 1.0 mmol, 5.0 equiv),

cyclopent-4-ene-1,3-dione (19.2 mg, 0.2 mmol, 1.0 equiv), and 9-mesityl-10-methylacridinium perchlorate **4** (1.0 mg, 0.0025 mmol, 0.0125 equiv) in DCE (2.0 mL) were employed to give the product **63** (30 mg) in 74% yield as a pale yellow oil. ^{1}H NMR (300 MHz, $CDCl_3$) δ 7.12 (d, J = 7.7 Hz, 2H), 7.06 (d, J = 7.6 Hz, 2H), 3.21 – 3.00 (m, 2H), 2.79 – 2.54 (m, 2H), 2.42 (d, J = 17.0 Hz, 1H), 2.32 (s, 3H); ^{13}C NMR (75 MHz, $CDCl_3$) δ 180.7, 178.5, 136.5, 134.5, 129.4, 128.8, 42.9, 36.9, 34.5, 21.0; HRMS–APCI $[M + H]^+$ Calcd for $C_{12}H_{13}O_3$ 205.0859, found 205.0864.

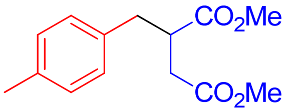

Dimethyl 2-(4-methylbenzyl)succinate (**64**)

Following the typical procedure **V**, *p*-xylene (106 mg, 1.0 mmol, 5.0 equiv), dimethyl maleate (29 mg, 0.2 mmol, 1.0 equiv), and 9-mesityl-10-methylacridinium perchlorate **4** (1.0 mg, 0.0025 mmol, 0.0125 equiv) in DCE (2.0 mL) were employed to give the product **64** (34.5 mg) in 69% yield as a pale yellow oil. ^{1}H NMR (300 MHz, $CDCl_3$) δ 7.09 (d, J = 8.0 Hz, 2H), 7.03 (d, J = 8.1 Hz, 2H), 3.67 (s, 3H), 3.63 (s, 3H), 3.17 – 2.94 (m, 2H), 2.78 – 2.58 (m, 2H), 2.43 (d, J = 4.8 Hz, 1H), 2.31 (s, 3H); ^{13}C NMR (75 MHz, $CDCl_3$) δ 174.7, 172.3, 136.2, 135.0, 129.2, 128.9, 51.8, 51.7, 43.1, 37.3, 34.9, 20.9; HRMS–ESI $[M + Na]^+$ Calcd for $C_{14}H_{18}NaO_4$ 273.1097, found 273.1102.

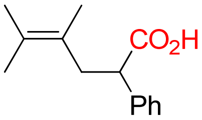

4,5-Dimethyl-2-phenylhex-4-enoic acid (**66**)

In a Schlenk tube equipped with a magnetic stirring bar was charged with alkylation product **7** (238 mg, 1.0 mmol, 1.0 equiv), CuI (38 mg, 0.2 mmol, 0.2 equiv), and Cs_2CO_3 (652 mg, 2.0 mmol, 2.0 equiv) in DMSO (2.0 mL), and the resulting mixture was performed in air at 140 ^{0}C for 18 h. After the reaction was cooled to room temperature, ethyl acetate 5 mL was added and the solution was filtrated. Then HCl (1 M) was added to adjusted the pH to 2～3. The solution was extracted with ethyl acetate (3×5 mL). The combined organic phase was washed with water and brine, dried over anhydrous Na_2SO_4, and purified using column chromatography isolation on silica gel by gradient elution with hexane / ethyl acetate (10:1～2:1) to give the acid **66** (157 mg) as a pale yellow oil in 72% yield. ^{1}H NMR (300 MHz, $CDCl_3$) δ 7.39 – 7.22 (m, 5H), 3.72 (t, J = 7.6 Hz, 1H), 2.89 (dd, J = 13.6, 7.9 Hz, 1H), 2.50 (dd, J = 13.6, 7.4 Hz, 1H), 1.59 (s, 6H), 1.55 (s, 3H);

^{13}C NMR (75 MHz, $CDCl_3$) δ 180.3, 138.5, 128.4, 128.1, 127.5, 127.3, 123.9, 50.4, 38.4, 20.6, 20.3, 18.4; HRMS–ESI [M – H]$^-$ Calcd for $C_{14}H_{17}O_2$ 217.1234, found 217.1237.

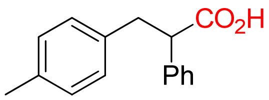

2-Phenyl-3-(*p*-tolyl)propanoic acid (67)

In a Schlenk tube equipped with a magnetic stirring bar was charged with alkylation product **51** (260 mg, 1.0 mmol, 1.0 equiv), CuI (38 mg, 0.2 mmol, 0.2 equiv), and Cs_2CO_3 (652 mg, 2.0 mmol, 2.0 equiv) in DMSO (2.0 mL), and the resulting mixture was performed in air at 140 ^{0}C for 18 h. After the reaction was cooled to room temperature, ethyl acetate 5 mL was added and the solution was filtrated. Then HCl (1 M) was added to adjusted the pH to 2～3. The solution was extracted with ethyl acetate (3×5 mL). The combined organic phase was washed with water and brine, dried over anhydrous Na_2SO_4, and purified using column chromatography isolation on silica gel by gradient elution with hexane / ethyl acetate (10:1～2:1) to give the acid **67** (180 mg) as a pale red solid in 75% yield. ^{1}H NMR (400 MHz, $CDCl_3$) δ 7.37 – 7.25 (m, 5H), 7.03 (q, J = 8.2 Hz, 4H), 3.85 (dd, J = 8.4, 7.0 Hz, 1H), 3.38 (dd, J = 13.9, 8.5 Hz, 1H), 3.01 (dd, J = 13.9, 6.9 Hz, 1H), 2.30 (s, 3H); ^{13}C NMR (101 MHz, $CDCl_3$) δ 179.3, 138.0, 135.9, 135.6, 129.0, 128.7, 128.6, 128.1, 127.5, 53.5, 38.8, 20.9; HRMS–ESI [M + Na]$^+$ Calcd for $C_{16}H_{16}NaO_2$ 263.1043, found 263.1046.

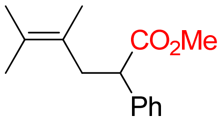

Methyl 4,5-dimethyl-2-phenylhex-4-enoate (68)

At – 20 ^{o}C, MCPBA (78 mg, 0.45 mmol, 1.5 equiv) was added to a mixture of alkylation product **7** (72 mg, 0.3 mmol, 1.0 equiv) and Cs_2CO_3 (103 mg, 0.315 mmol, 1.05 equiv) in MeOH (2.0 mL). After 12 hrs, the reaction was warmed to room temperature and stirred for another 12 hrs. The mixture was filtered through a short column of SiO_2, and purified using column chromatography isolation on silica gel by gradient elution with hexane / ethyl acetate (50:1～30:1) to give the acid **68** (48 mg) as a colorless oil in 69% yield. ^{1}H NMR (500 MHz, $CDCl_3$) δ 7.36 – 7.30 (m, 4H), 7.30 – 7.25 (m, 1H), 3.73 (t, J = 7.7 Hz, 1H), 3.67 (s, 3H), 2.87 (dd, J = 13.5, 8.0 Hz, 1H), 2.52 (dd, J = 13.5, 7.4 Hz, 1H), 1.61 (s, 6H), 1.56 (s, 3H); ^{13}C NMR (126 MHz, $CDCl_3$) δ 174.4, 139.2, 128.4, 127.9, 127.3, 127.1, 124.1, 51.8, 50.4, 38.8, 20.6, 20.2, 18.4; HRMS–ESI [M + Na]$^+$ Calcd for $C_{15}H_{20}NaO_2$ 255.1356, found 255.1354.

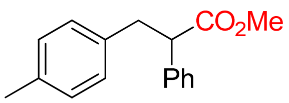

Methyl 2-phenyl-3-(*p*-tolyl)propanoate (69)

At – 20 °C, MCPBA (78 mg, 0.45 mmol, 1.5 equiv) was added to a mixture of alkylation product **51** (78 mg, 0.3 mmol, 1.0 equiv) and Cs_2CO_3 (103 mg, 0.315 mmol, 1.05 equiv) in MeOH (2.0 mL). After 12 hrs, the reaction was warmed to room temperature and stirred for another 12 hrs. The mixture was filtered through a short column of SiO_2, and purified using column chromatography isolation on silica gel by gradient elution with hexane / ethyl acetate (50:1～30:1) to give the acid **69** (62.5 mg) as a colorless oil in 82% yield. ^{1}H NMR (500 MHz, $CDCl_3$) δ 7.34 (d, *J* = 4.3 Hz, 4H), 7.31 – 7.26 (m, 1H), 7.07 (d, *J* = 8.1 Hz, 2H), 7.04 (d, *J* = 8.1 Hz, 2H), 3.86 (dd, *J* = 8.9, 6.6 Hz, 1H), 3.63 (s, 3H), 3.41 (dd, *J* = 13.8, 8.9 Hz, 1H), 3.02 (dd, *J* = 13.8, 6.6 Hz, 1H), 2.32 (s, 3H); ^{13}C NMR (126 MHz, $CDCl_3$) δ 173.9, 138.8, 135.9, 135.8, 129.0, 128.7, 128.6, 127.9, 127.3, 53.7, 51.9, 39.4, 21.0; HRMS–ESI $[M + Na]^+$ Calcd for $C_{17}H_{18}NaO_2$ 277.1199, found 277.1203.

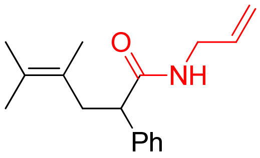

***N*-allyl-4,5-dimethyl-2-phenylhex-4-enamide (70)**

Under O_2 atmosphere, allylamine (57 mg, 1.0 mmol, 2.0 equiv) was added to a solution of alkylation product **7** (119 mg, 0.5 mmol, 1.0 equiv) and K_2CO_3 (138 mg, 1.0 mmol, 2.0 equiv) in CH_3CN (5.0 mL). The resulting mixture was stirred at rt until the compound **7** was completely consumed as monitored by TLC. The solution was filtered, then concentrated and purified using column chromatography isolation on silica gel by gradient elution with hexane / ethyl acetate (10:1～5:1) to give the amide **70** (114 mg) as a pale yellow semi-solid in 89% yield. ^{1}H NMR (500 MHz, $CDCl_3$) δ 7.32 – 7.26 (m, 4H), 7.26 – 7.21 (m, 1H), 5.80 – 5.69 (m, 1H), 5.61 (br s, 1H), 5.09 – 4.98 (m, 2H), 3.90 – 3.82 (m, 1H), 3.81 – 3.73 (m, 1H), 3.46 (t, *J* = 7.5 Hz, 1H), 2.88 (dd, *J* = 13.6, 6.9 Hz, 1H), 2.56 (dd, *J* = 13.6, 8.1 Hz, 1H), 1.56 (s, 6H), 1.50 (s, 3H); ^{13}C NMR (126 MHz, $CDCl_3$) δ 173.2, 140.1, 134.2, 128.4, 128.1, 127.0, 126.8, 124.7, 115.9, 52.1, 41.9, 38.3, 20.6, 20.3, 18.4; HRMS–ESI $[M + Na]^+$ Calcd for $C_{17}H_{23}NNaO$ 280.1672, found 280.1667.

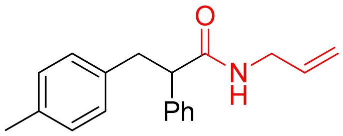

***N*-allyl-2-phenyl-3-(p-tolyl)propanamide** (**71**)

Under O_2 atmosphere, allylamine (57 mg, 1.0 mmol, 2.0 equiv) was added to a solution of alkylation product **51** (130 mg, 0.5 mmol, 1.0 equiv) and K_2CO_3 (138 mg, 1.0 mmol, 2.0 equiv) in CH_3CN (5.0 mL). The resulting mixture was stirred at rt until the compound **51** was completely consumed as monitored by TLC. The solution was filtered, then concentrated and purified using column chromatography isolation on silica gel by gradient elution with hexane / ethyl acetate (10:1～5:1) to give the amide **71** (112 mg) as a colorless oil in 80% yield. ^{1}H NMR (500 MHz, $CDCl_3$) δ 7.35 – 7.27 (m, 4H), 7.27 – 7.22 (m, 1H), 7.06 – 6.98 (m, 4H), 5.72 – 5.60 (m, 2H), 4.98 (d, *J* = 10.3 Hz, 1H), 4.89 (d, *J* = 17.2 Hz, 1H), 3.85 – 3.68 (m, 2H), 3.60 (t, *J* = 7.4 Hz, 1H), 3.51 (dd, *J* = 13.5, 8.1 Hz, 1H), 2.97 (dd, *J* = 13.5, 6.8 Hz, 1H), 2.28 (s, 3H); ^{13}C NMR (126 MHz, $CDCl_3$) δ 172.6, 139.7, 136.5, 135.5, 133.9, 128.9, 128.8, 128.6, 127.9, 127.2, 115.8, 55.59, 41.8, 39.2, 20.9; HRMS–ESI $[M + Na]^+$ Calcd for $C_{19}H_{21}NNaO$ 302.1515, found 302.1518.

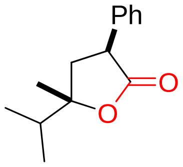

5-Isopropyl-5-methyl-3-phenyldihydrofuran-2(3*H*)-one (**72**)

To a 25 mL Schlenk tube equipped with a magnetic stir bar was added the 4,5-dimethyl-2-phenylhex-4-enoic acid (**66**) (44 mg, 0.2 mmol, 1.0 equiv.), 2-phenylmalononitrile (14.2 mg, 0.1 mmol, 0.5 equiv), and 9-mesityl-10-methylacridinium perchlorate **4** (4.1 mg, 0.01 mmol, 0.05 equiv.). The resulting mixture was sealed and degassed via vacuum evacuation and subsequent backfill with argon for three times. Then anhydrous DCE (0.2 mL) was added, and finally 2,6-lutidine (2.1 mg, 0.02 mmol, 0.1 equiv.) was added. After that, the reaction was placed under a blue LED with an argon balloon and irradiated for 72 hrs. The solvent was removed on a rotary evaporator under reduced pressure and the residue was subjected to column chromatography isolation on silica gel by gradient elution with hexane / ethyl acetate (15:1-10:1) to give the lactone **72** (30.5 mg) as a colorless oil in 70% yield and 10:1 dr. The overall yield of lactone **72** starting from alkylation product **7** is thus calculated as 50%.

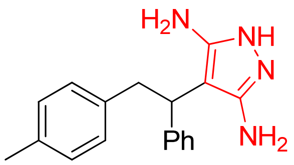

4-(1-Phenyl-2-(*p*-tolyl)ethyl)-1H-pyrazole-3,5-diamine (**73**)

The alkylation product **51** (260 mg, 1.0 mmol, 1.0 equiv) was dissolved in hydrazine hydrate (300 mg, 6.0 mmol, 6.0 equiv). The mixture was heated under reflux for 1.5 h, and then cooled to room temperature. Ethanol was added and the precipitated was collected. Recrystallization from ethyl acetate and hexane gave the pyrazole **73** (240 mg) as a gray solid in 82% yield. ^{1}H NMR (500 MHz, $CDCl_3$) δ 7.37 – 7.27 (m, 4H), 7.21 (t, J = 7.0 Hz, 1H), 7.00 (d, J = 7.8 Hz, 2H), 6.95 (d, J = 7.9 Hz, 2H), 4.37 (br s, 2H), 3.92 (dd, J = 10.5, 5.0 Hz, 1H), 3.33 (dd, J = 12.7, 5.0 Hz, 1H), 3.09 (dd, J = 12.5, 10.8 Hz, 1H), 2.28 (s, 3H); ^{13}C NMR (126 MHz, $CDCl_3$) δ 148.8, 143.3, 137.6, 135.5, 129.0, 128.9, 128.5, 127.6, 126.3, 92.9, 41.6, 38.9, 20.9; HRMS–ESI $[M + Na]^+$ Calcd for $C_{18}H_{20}N_4Na$ 315.1580, found 315.1588.

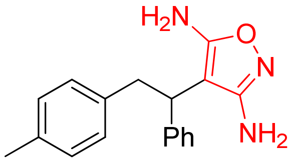

4-(1-Phenyl-2-(*p*-tolyl)ethyl)isoxazole-3,5-diamine (**74**)

To a stirred mixture of hydroxylamine hydrochloride (35 mg, 0.5 mmol, 1.0 equiv) and alkylation product **51** (130 mg, 0.5 mmol, 1.0 equiv) in methanol (3.0 mL), a solution of triethylamine (70 μL, 0.5 mmol, 1.0 equiv) in methanol (1.0 mL) was added dropwise. After the addition has been completed, a second portion of triethylamine (70 uL, 0.5 mmol, 1.0 equiv) was added and the mixture was stirred at room temperature for 48 hrs. The solvent was removed on a rotary evaporator under reduced pressure and the residue was subjected to column chromatography isolation on silica gel by gradient elution with hexane / ethyl acetate (5:1-1:1) to give the isoxazole **74** (86 mg) as a gray solid in 59% yield. ^{1}H NMR (300 MHz, $CDCl_3$) δ 7.37 (d, J = 4.3 Hz, 4H), 7.32 – 7.27 (m, 1H), 7.10 (d, J = 7.9 Hz, 2H), 7.03 (d, J = 8.0 Hz, 2H), 3.86 (dd, J = 11.1, 4.6 Hz, 1H), 3.68 – 3.40 (br s, 2H), 3.35 (dd, J = 12.7, 4.6 Hz, 1H), 3.14 – 3.03 (m, 1H), 2.32 (s, 3H); ^{13}C NMR (101 MHz, $CDCl_3$) δ 164.4, 141.7, 136.9, 136.1, 129.4, 129.1, 128.8, 127.6, 127.0, 86.9, 40.9, 38.6, 21.0.

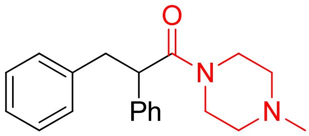

1-(4-Methylpiperazin-1-yl)-2,3-diphenylpropan-1-one (**75**)

Under O_2 atmosphere, 1-methylpiperazine (39 mg, 0.39 mmol, 2.0 equiv) was added to a solution of alkylation product **53** (47.5 mg, 0.19 mmol, 1.0 equiv) and K_2CO_3 (54 mg, 0.39 mmol, 2.0 equiv) in CH_3CN (2.0 mL). The resulting mixture was stirred at rt until the compound **53** was completely consumed as monitored by TLC. The solution was filtered, then concentrated and purified using column chromatography isolation on silica gel by gradient elution with hexane / ethyl acetate / methanol to give the amide **75** (47 mg) as a colorless oil in 80% yield. This is a known compound[6]. ^{1}H NMR (500 MHz, $CDCl_3$) δ 7.33 – 7.28 (m, 2H), 7.27 – 7.21 (m, 5H), 7.20 – 7.16 (m, 1H), 7.13 – 7.08 (m, 2H), 4.02 – 3.95 (m, 1H), 3.77 – 3.67 (m, 1H), 3.61 – 3.47 (m, 2H), 3.39 – 3.30 (m, 2H), 2.98 (dd, J = 13.5, 6.7 Hz, 1H), 2.34 – 2.26 (m, 1H), 2.26 – 2.18 (m, 1H), 2.16 (s, 3H), 2.05 – 1.96 (m, 1H), 1.84 – 1.74 (m, 1H); ^{13}C NMR (101 MHz, $CDCl_3$) δ 170.9, 139.9, 139.7, 129.2, 128.6, 128.1, 127.9, 126.9, 126.1, 54.6, 54.5, 50.8, 45.8, 45.4, 41.9, 41.0.

XI. Supplementary References

(1) M. Frisch, G. Trucks, H. Schlegel, G. Scuseria, M. Robb, J. Cheeseman, G. Scalmani, V. Barone, B. Mennucci, G. Petersson, *Gaussian Inc., Wallingford CT*, **2010**.

(2) J. A. Montgomery Jr., M. J. Frisch, J. W. Ochterski, G. A. Petersson, *J. Chem. Phys.* **1999**, *110,* 2822-2827.

(3) J. A. Montgomery Jr., M. J. Frisch, J. W. Ochterski, G. A. Petersson, *J. Chem. Phys.* **2000**, *112*, 6532-6542.

(4) B. Mennucci, J. Tomasi, *J. Chem. Phys.* **1997**, *106***,** 5151-5158.

(5) S. J. Blanksby, G. B. Ellison, *Acc. Chem. Res.* **2003**, *36*, 255-263.

(6) M. S. Poslusney, C. Sevel, T. J. Utley, T. M. Bridges, R. D. Morrison, N. R. Kett, D. J. Sheffler, C. M. Niswender, J. S. Daniels, P. J. Conn, C. W. Lindsley, M. R. Wood, *Bioorg. Med. Chem. Lett.* **2012**, *22*, 6923-6928.

XII. ORTEP Drawings for the Major Diastereomer of 41

Table S2. Crystal data and structure refinement for 41-major.

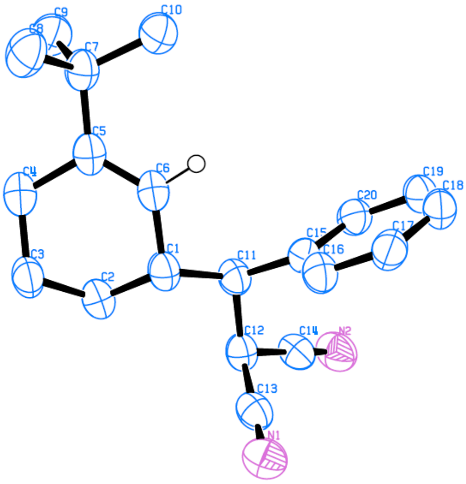

Identification code	**41-major**
Empirical formula	C_{20} $H_{24}N_2$
Formula weight	292.41
Temperature	100(2) K
Wavelength	1.54178 Å
Crystal system	Monoclinic
Space group	$P2_1/n$
Unit cell dimensions	a = 16.6451(18) Å
	b = 5.8536(6) Å
	c = 19.416(2) Å
Volume	1730.6(3) $Å^3$
Z	4
Density (calculated)	1.122 Mg/m^3
Absorption coefficient	0.499 mm^{-1}
F(000)	632
Crystal size	0.594 x 0.046 x 0.043 mm^3
Theta range for data collection	2.963 to 66.678°.
Index ranges	-18<=h<=19, -6<=k<=6, -23<=l<=23
Reflections collected	14377
Independent reflections	3030 [R(int) = 0.1171]
Completeness to theta = 66.678°	99.5 %
Absorption correction	Semi-empirical from equivalents
Max. and min. transmission	0.7528 and 0.4273
Refinement method	Full-matrix least-squares on F^2
Data / restraints / parameters	3030 / 0 / 202

Goodness-of-fit on F^2	1.032
Final R indices [I>2sigma(I)]	R1 = 0.0794, wR2 = 0.1965
R indices (all data)	R1 = 0.1297, wR2 = 0.2432
Extinction coefficient	n/a
Largest diff. peak and hole	0.323 and -0.331 e.Å^{-3}

XIII. ^{1}H, ^{13}C NMR Spectra of Products

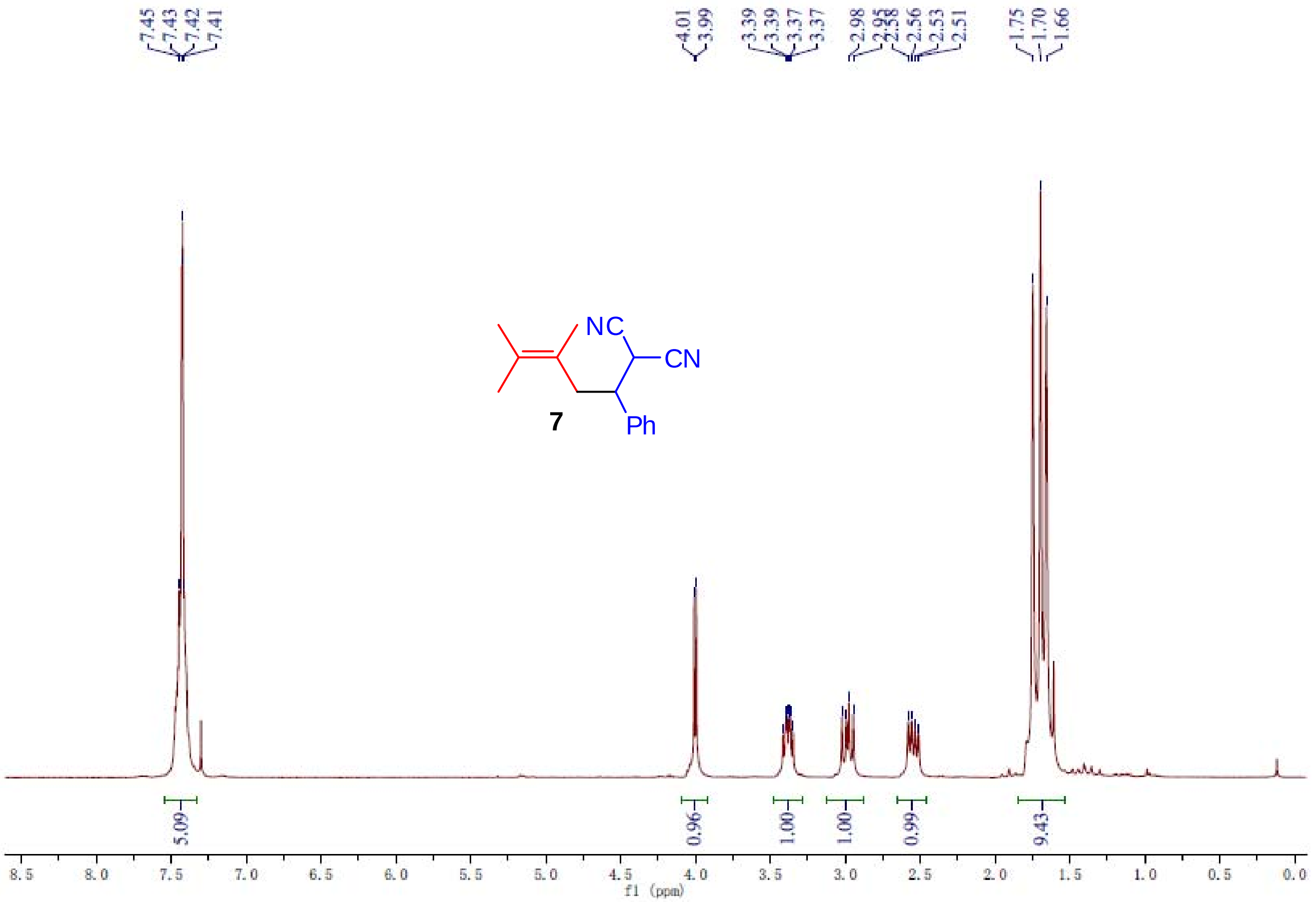

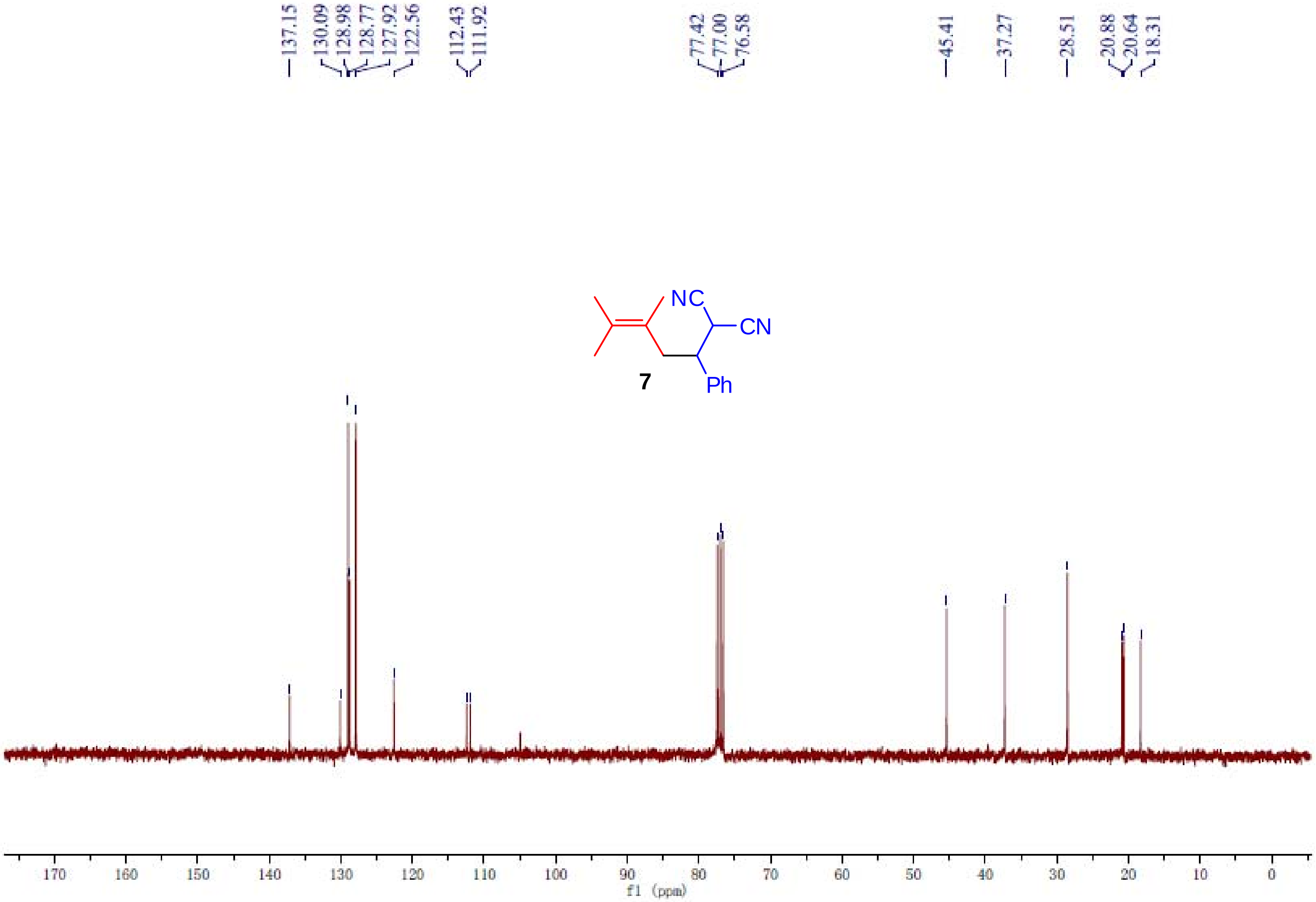
137.15
130.09
128.98
128.77
127.92
122.56
112.43
111.92
77.42
77.00
76.58
45.41
37.27
28.51
20.88
20.64
18.31
NC
CN
Ph
7
170 160 150 140 130 120 110 100 90 80 70 60 50 40 30 20 10 0
f1 (ppm)

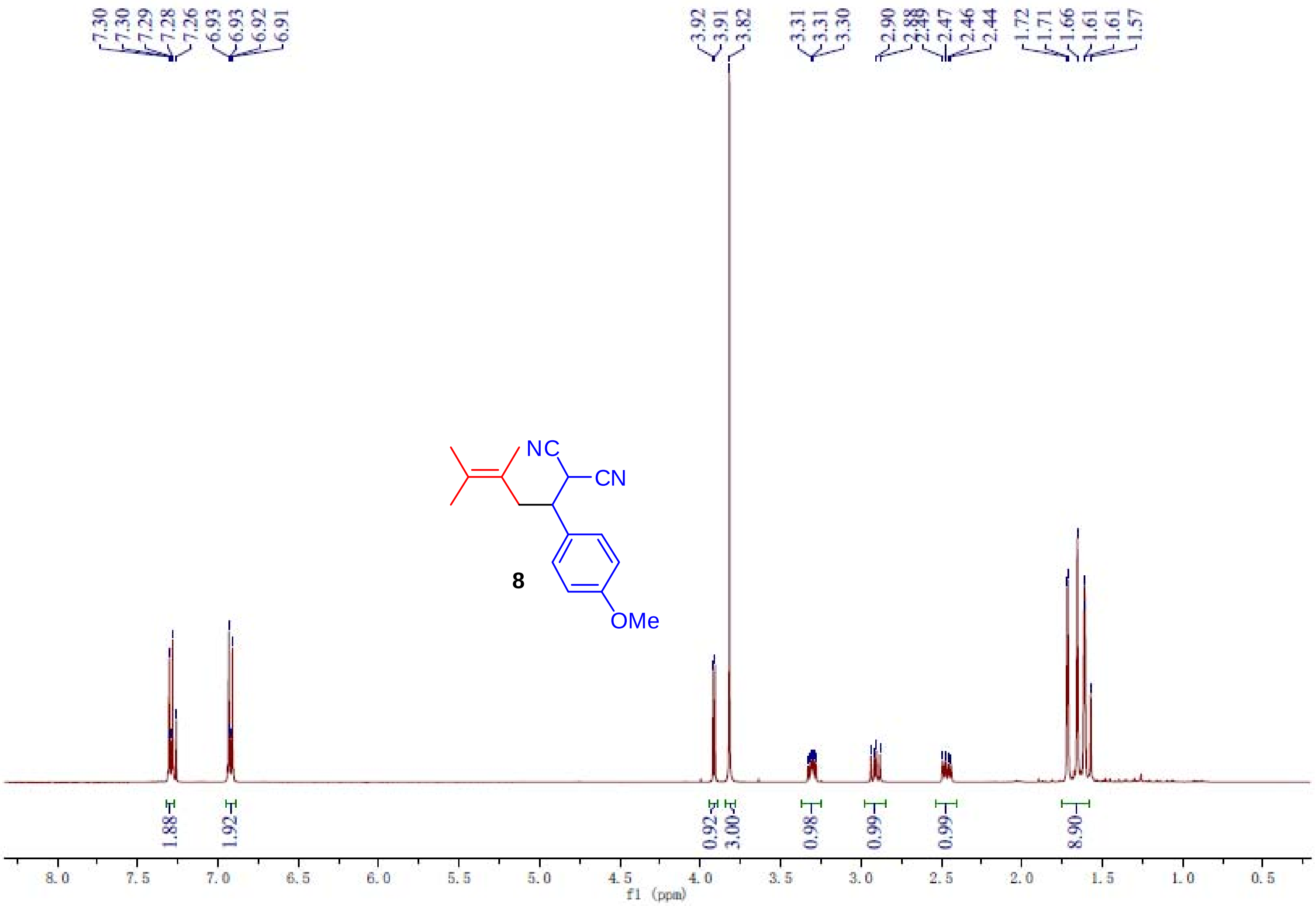

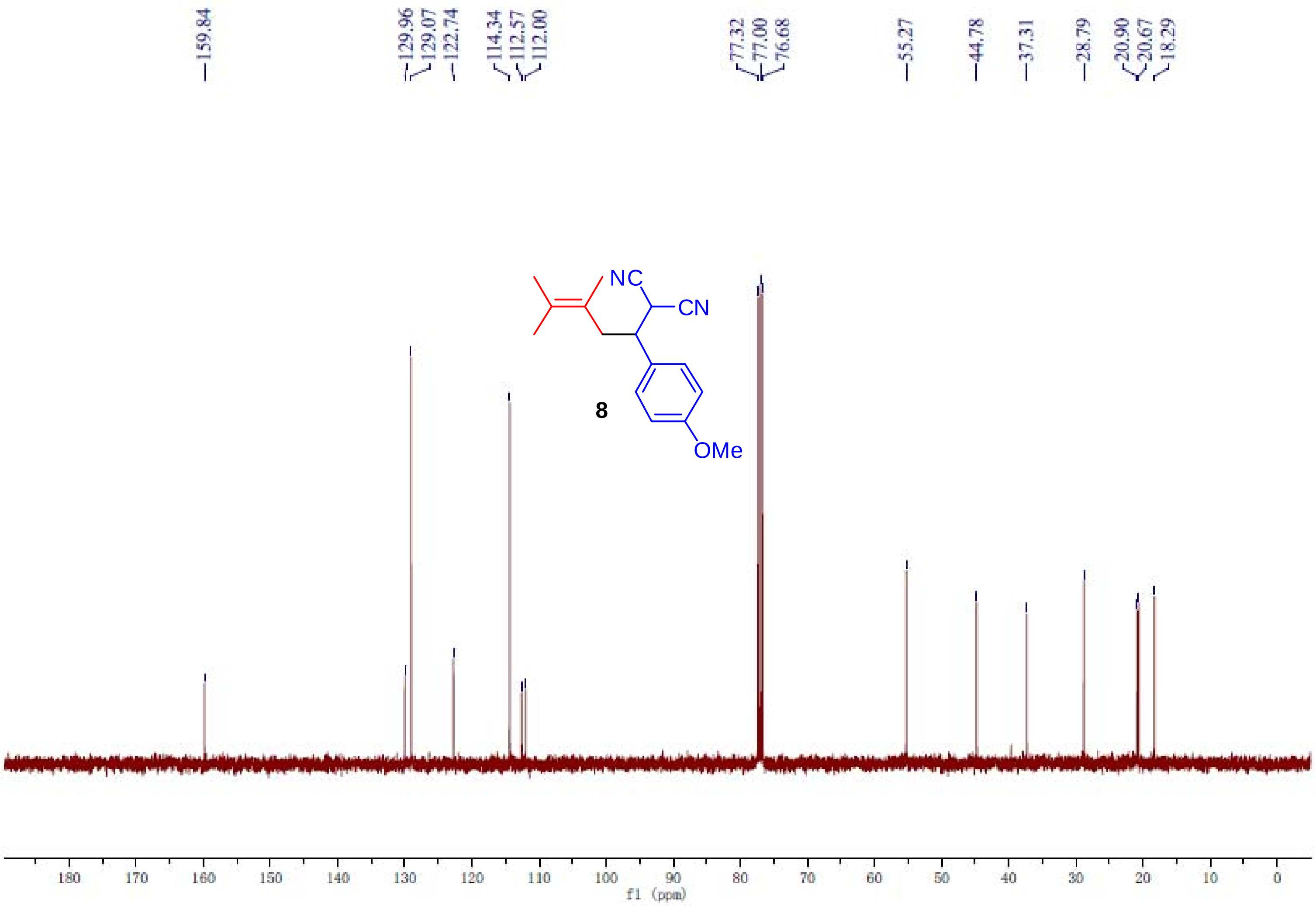
159.84
129.96
129.07
122.74
114.34
112.57
112.00
77.32
77.00
76.68
55.27
44.78
37.31
28.79
20.90
20.67
18.29
NC
CN
8
OMe
f1 (ppm)

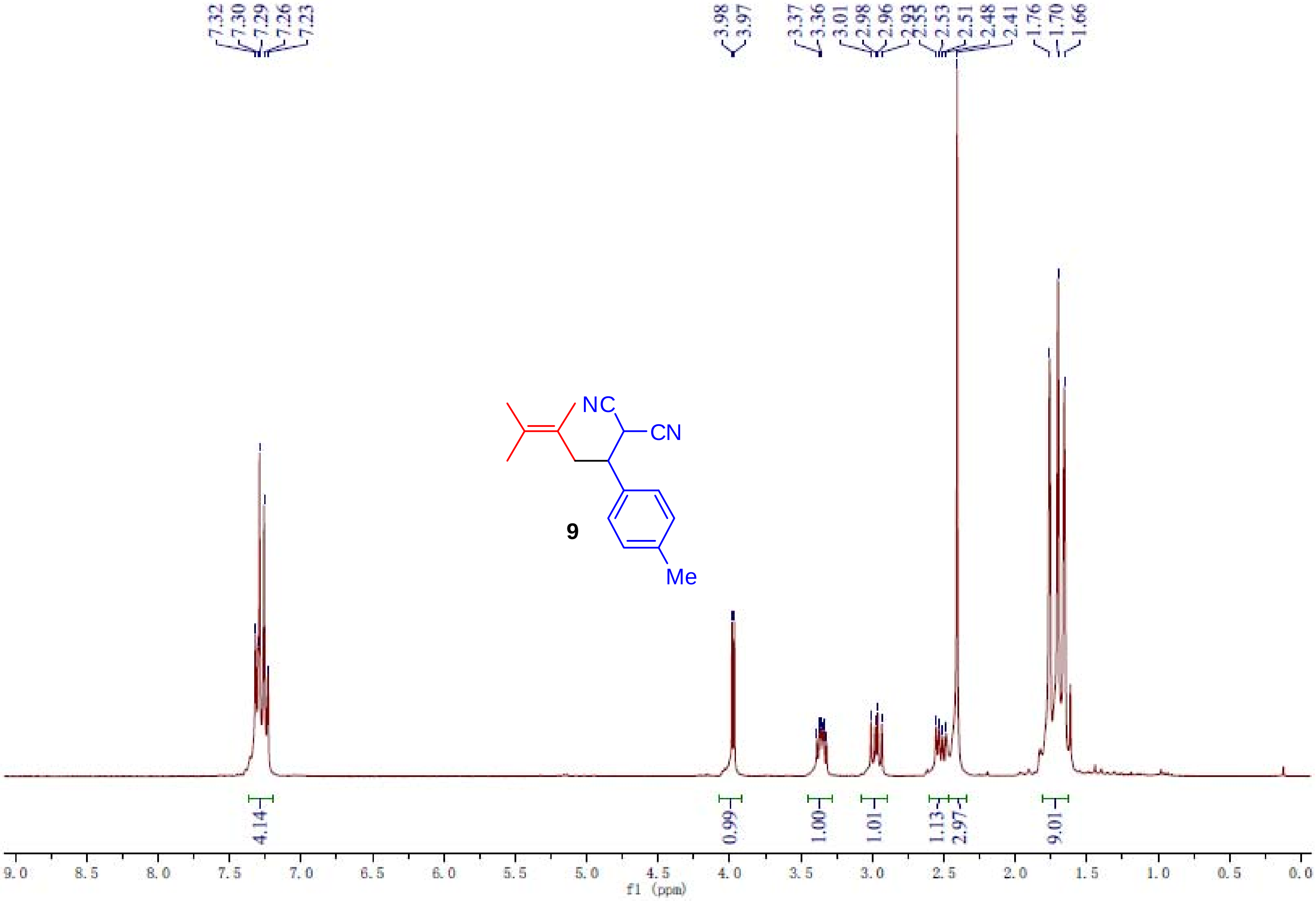
7.32
7.30
7.29
7.26
7.23
3.98
3.97
3.37
3.36
3.01
2.98
2.96
2.93
2.55
2.53
2.51
2.48
2.41
1.76
1.70
1.66
NC
CN
9
Me
4.14
0.99
1.00
1.01
1.13
2.97
9.01
9.0
8.5
8.0
7.5
7.0
6.5
6.0
5.5
5.0
4.5
4.0
3.5
3.0
2.5
2.0
1.5
1.0
0.5
0.0
f1 (ppm)

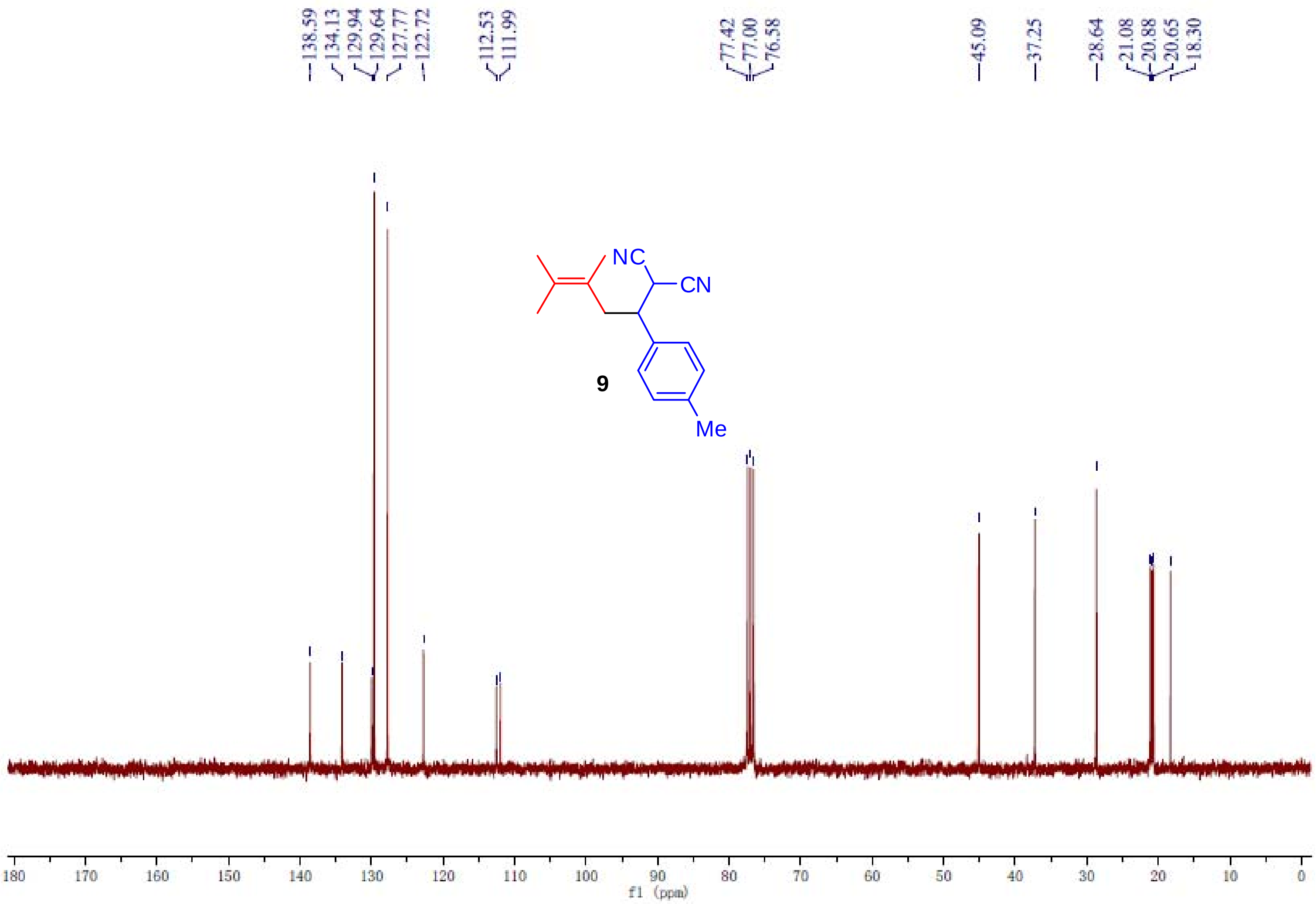

138.59
134.13
129.94
129.64
127.77
122.72
112.53
111.99
77.42
77.00
76.58
45.09
37.25
28.64
21.08
20.88
20.65
18.30
NC
CN
9
Me
180 170 160 150 140 130 120 110 100 90 80 70 60 50 40 30 20 10 0
f1 (ppm)

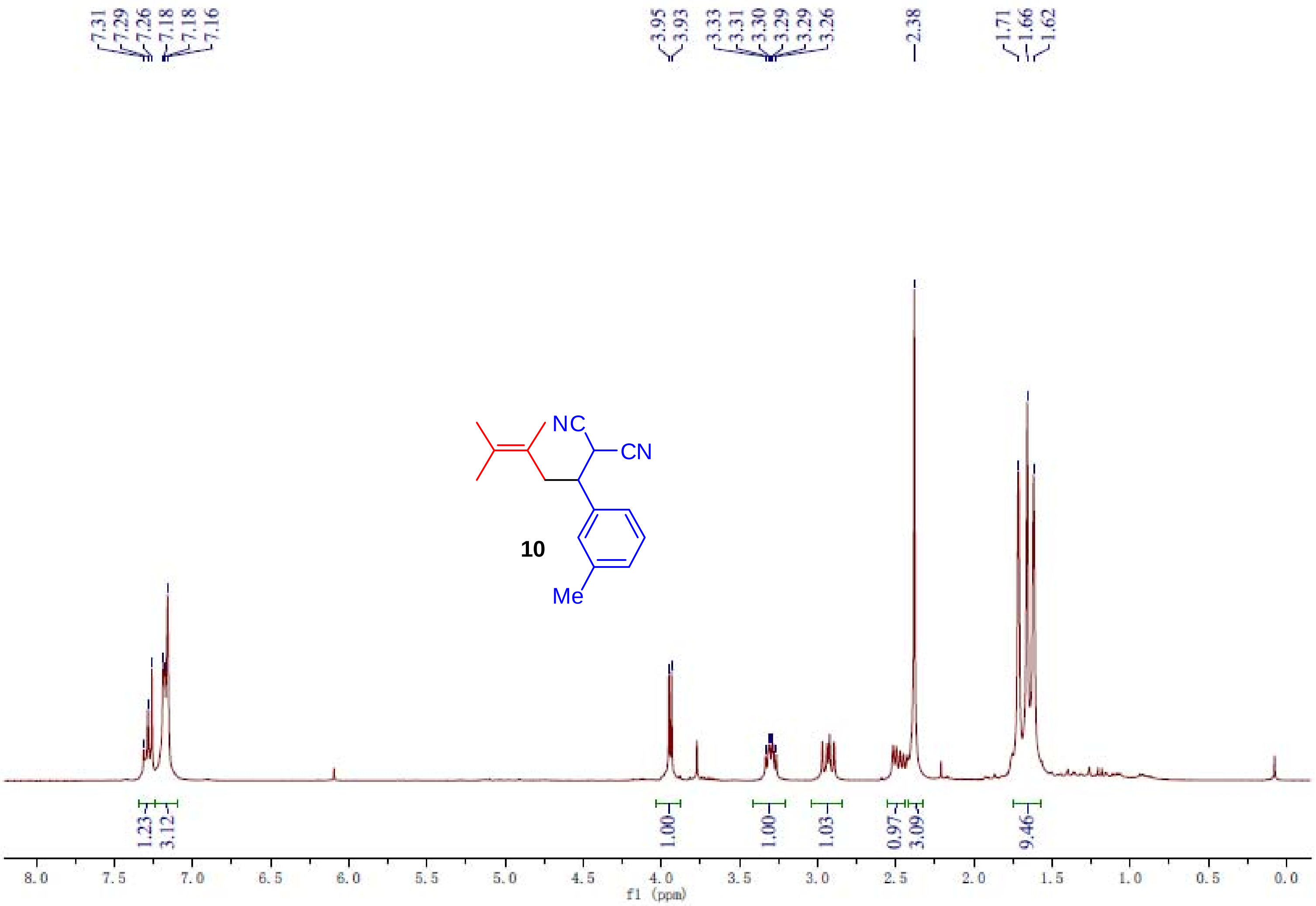
7.31
7.29
7.26
7.18
7.18
7.16
3.95
3.93
3.33
3.31
3.30
3.29
3.29
3.26
2.38
1.71
1.66
1.62
NC
CN
Me
10
1.23
3.12
1.00
1.00
1.03
0.97
3.09
9.46
8.0
7.5
7.0
6.5
6.0
5.5
5.0
4.5
4.0
3.5
3.0
2.5
2.0
1.5
1.0
0.5
0.0
f1 (ppm)

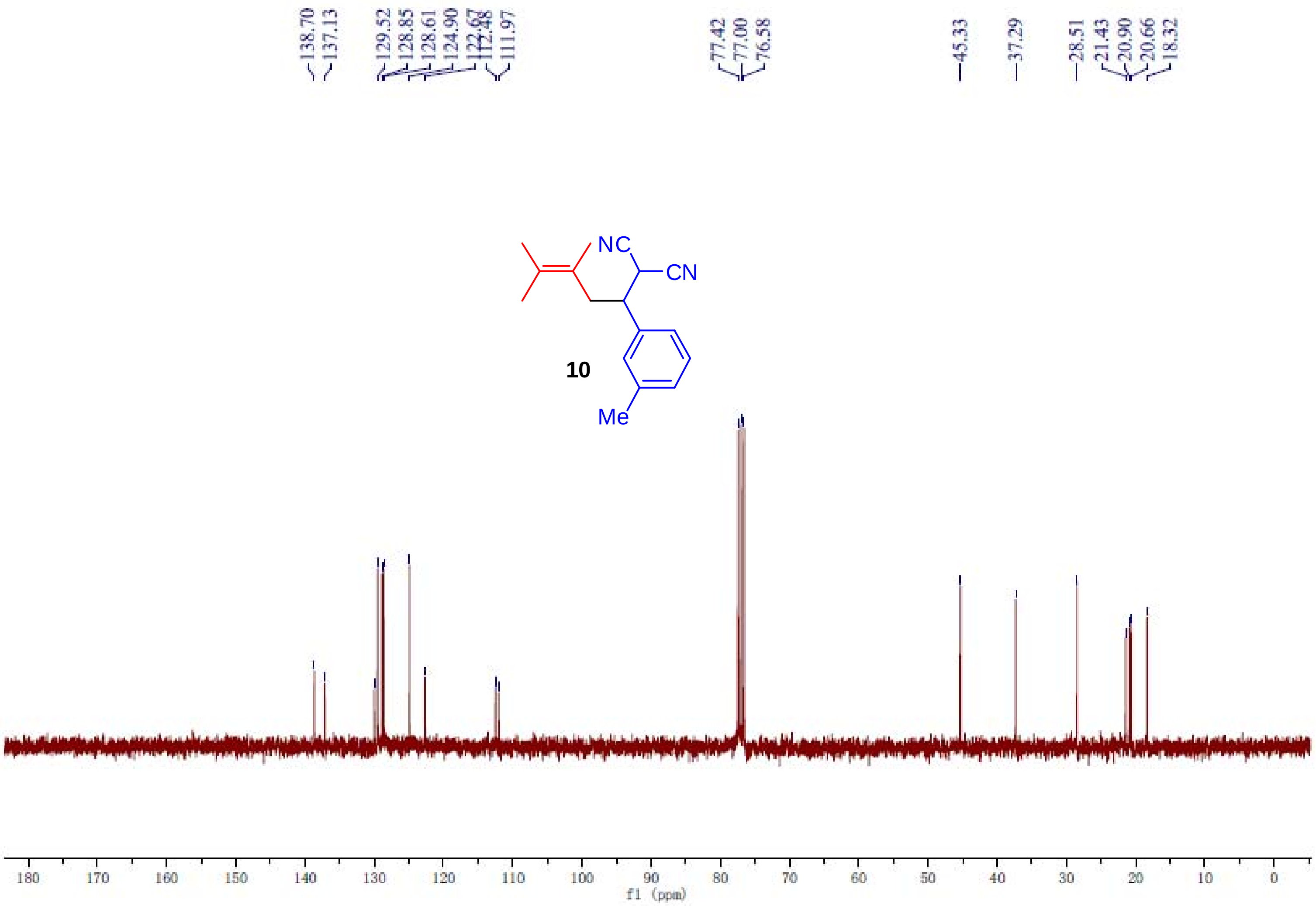

138.70
137.13
129.52
128.85
128.61
124.90
122.67
112.48
111.97
77.42
77.00
76.58
45.33
37.29
28.51
21.43
20.90
20.66
18.32
NC
CN
10
Me
180 170 160 150 140 130 120 110 100 90 80 70 60 50 40 30 20 10 0
f1 (ppm)

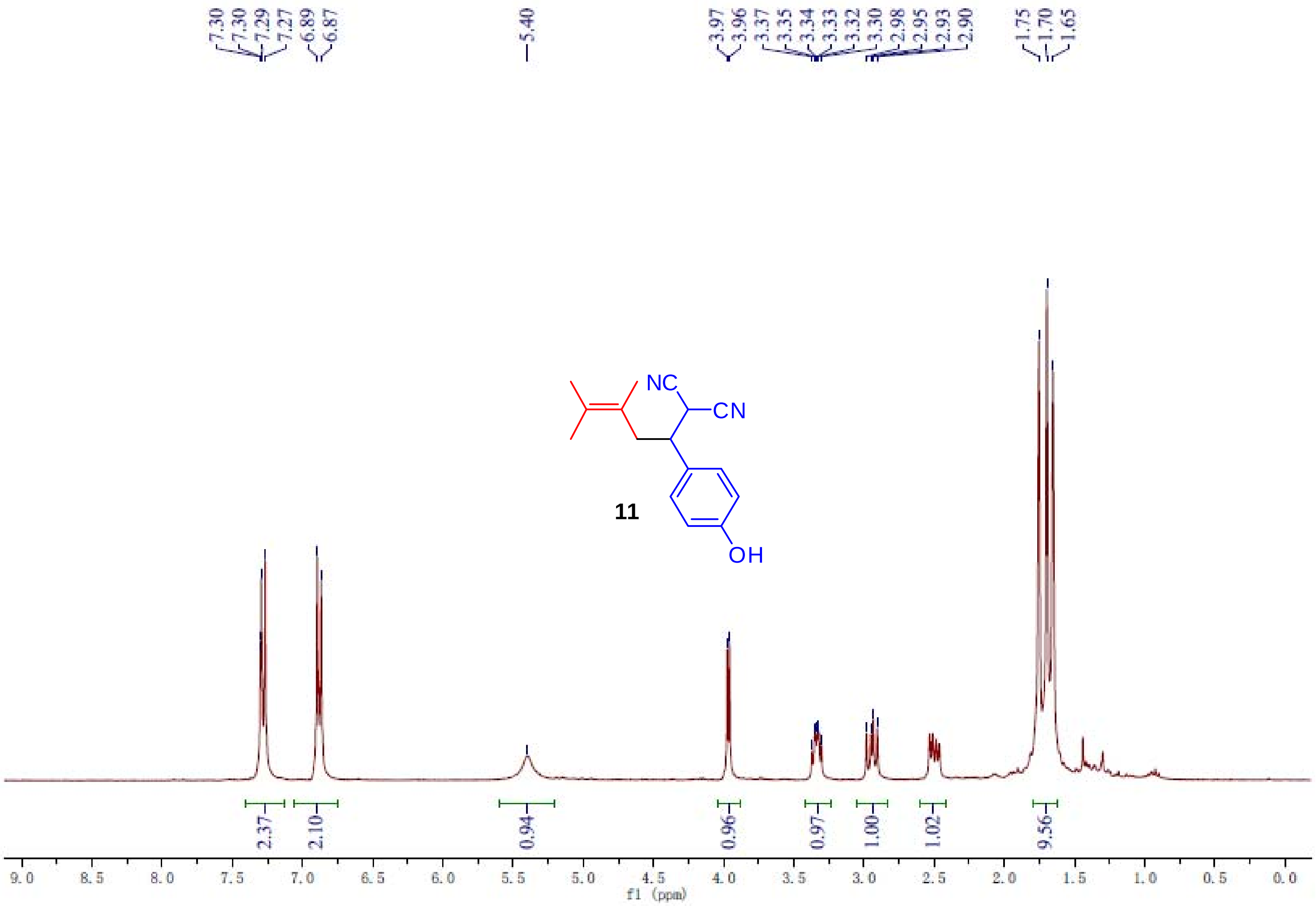
7.30
7.30
7.29
7.27
6.89
6.87
5.40
3.97
3.96
3.37
3.35
3.34
3.33
3.32
3.30
2.98
2.95
2.93
2.90
1.75
1.70
1.65
NC
CN
OH
11
2.37
2.10
0.94
0.96
0.97
1.00
1.02
9.56
9.0
8.5
8.0
7.5
7.0
6.5
6.0
5.5
5.0
4.5
4.0
3.5
3.0
2.5
2.0
1.5
1.0
0.5
0.0
f1 (ppm)

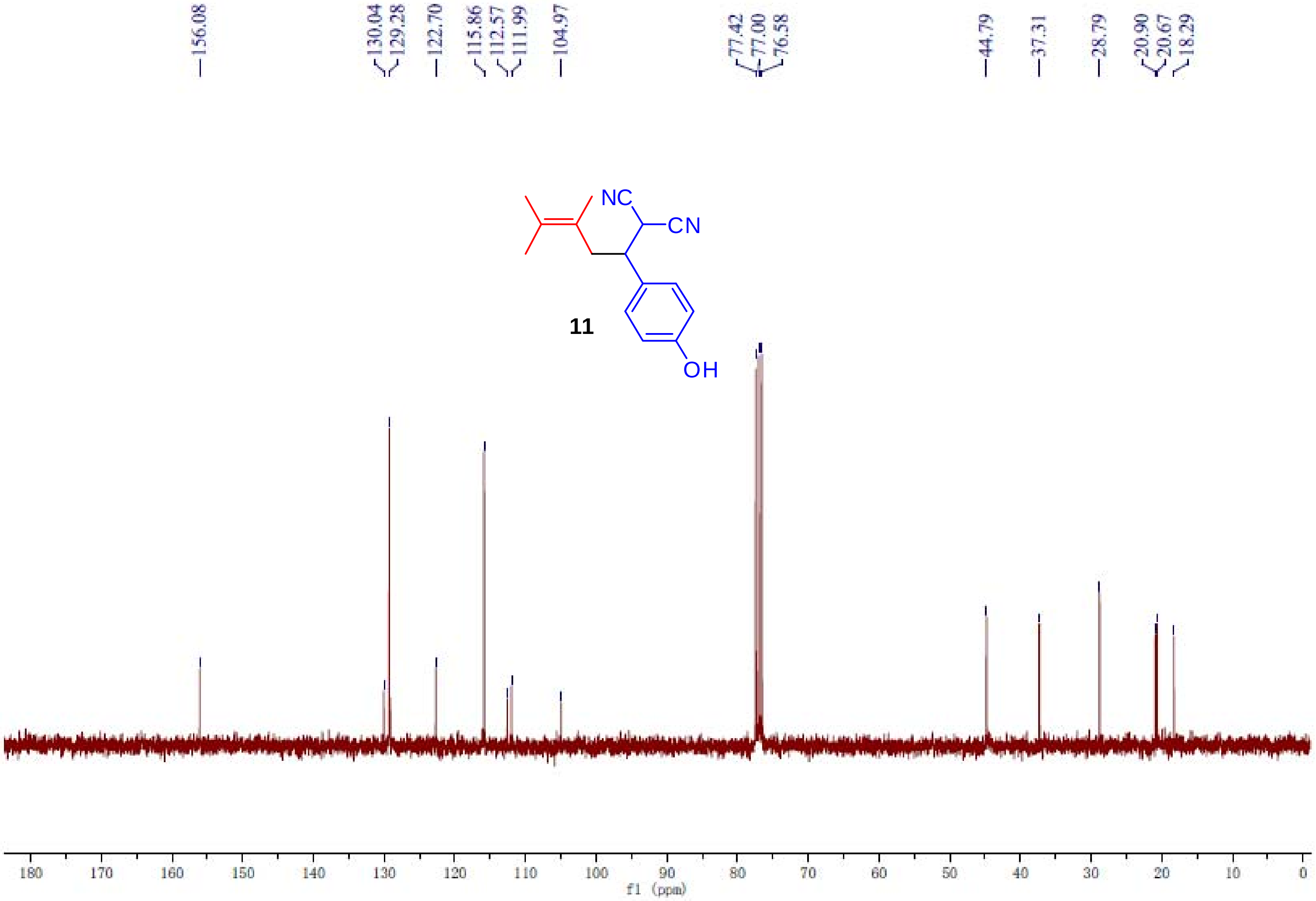
156.08
130.04
129.28
122.70
115.86
112.57
111.99
104.97
77.42
77.00
76.58
44.79
37.31
28.79
20.90
20.67
18.29
NC
CN
OH
11
f1 (ppm)

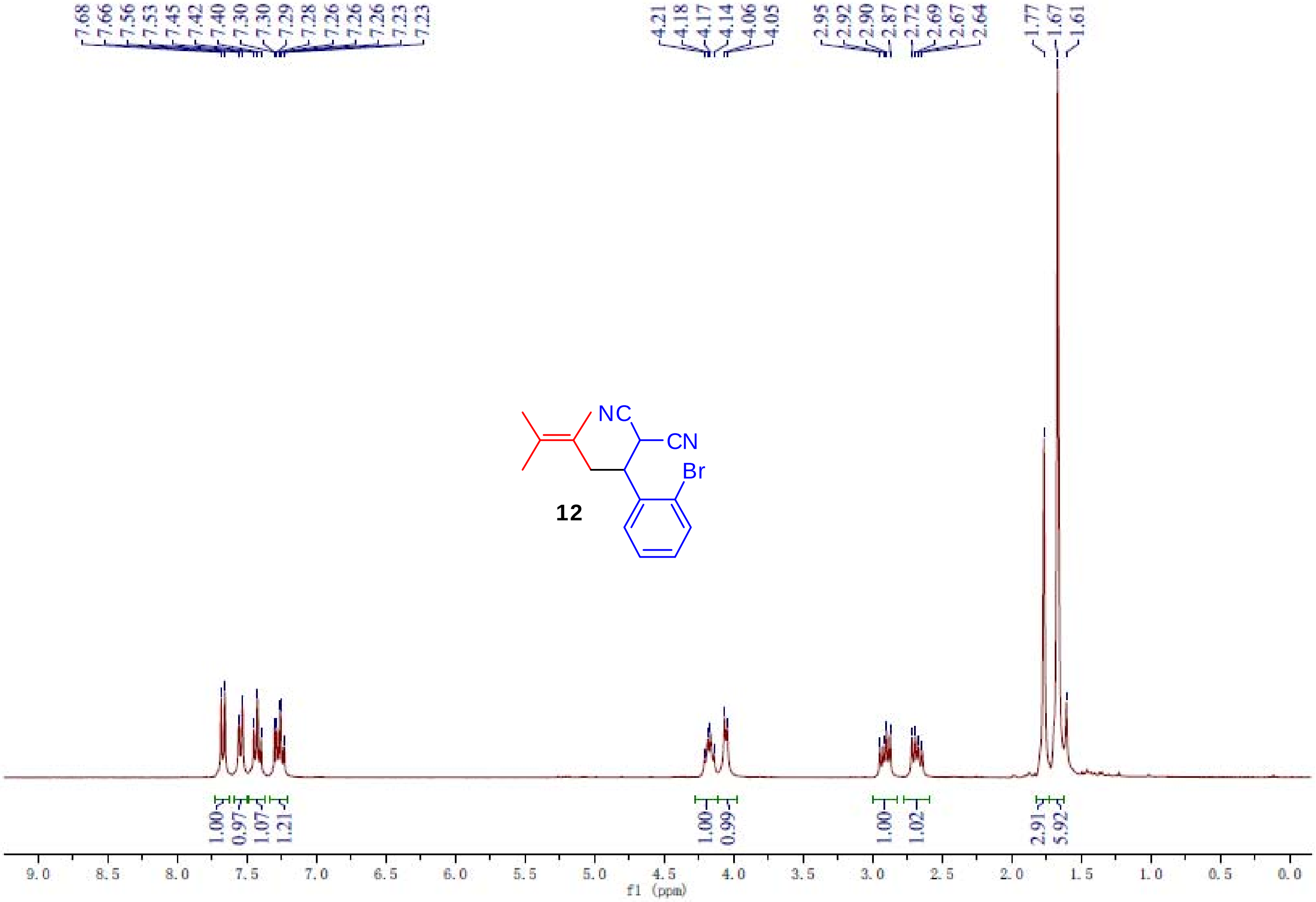
7.68
7.66
7.56
7.53
7.45
7.42
7.40
7.30
7.30
7.29
7.28
7.26
7.26
7.26
7.23
7.23
4.21
4.18
4.17
4.14
4.06
4.05
2.95
2.92
2.90
2.87
2.72
2.69
2.67
2.64
1.77
1.67
1.61
NC
CN
Br
12
1.00
0.97
1.07
1.21
1.00
0.99
1.00
1.02
2.91
5.92
9.0
8.5
8.0
7.5
7.0
6.5
6.0
5.5
5.0
4.5
4.0
3.5
3.0
2.5
2.0
1.5
1.0
0.5
0.0
f1 (ppm)

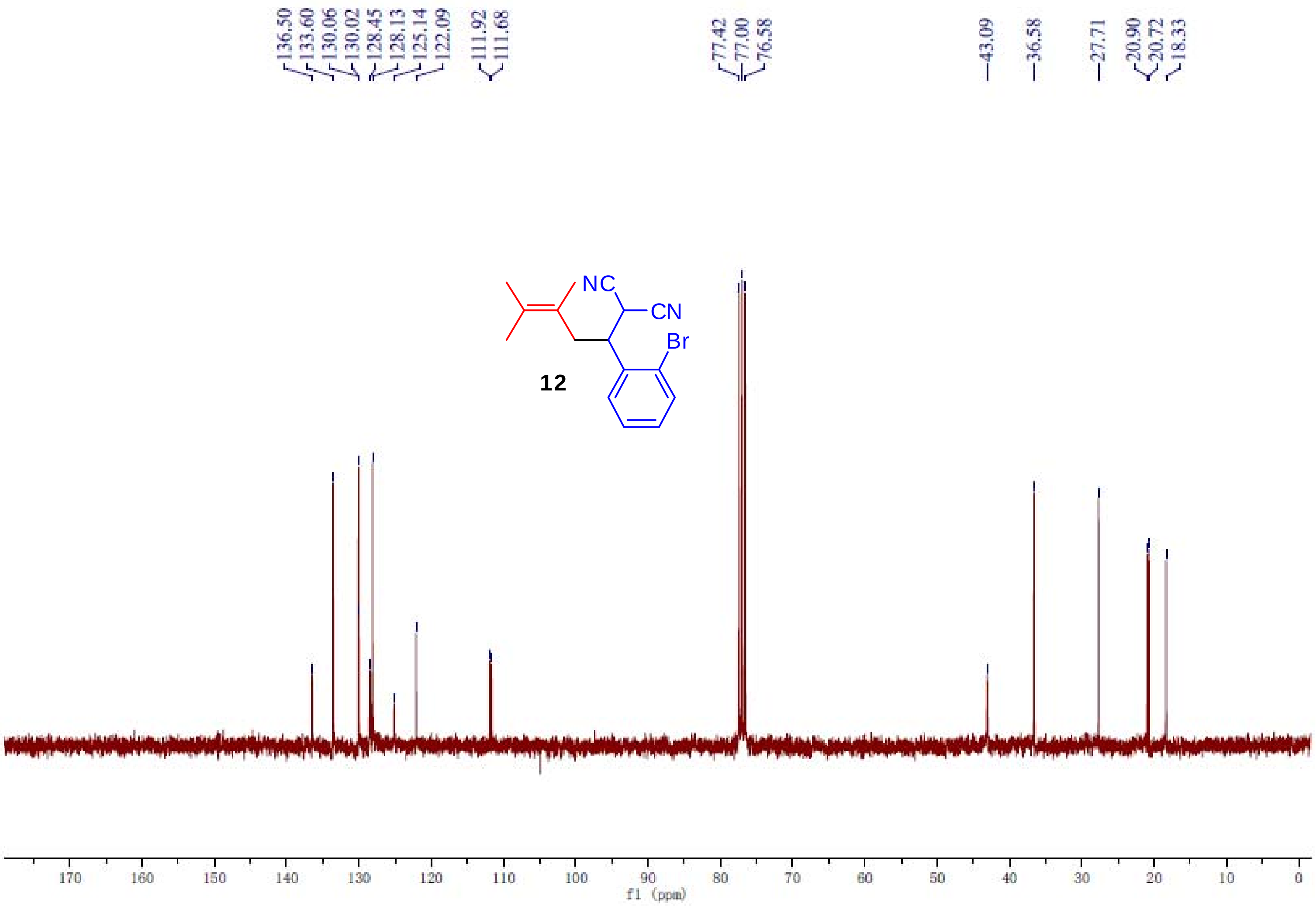

136.50
133.60
130.06
130.02
128.45
128.13
125.14
122.09
111.92
111.68
77.42
77.00
76.58
43.09
36.58
27.71
20.90
20.72
18.33
NC
CN
Br
12
170
160
150
140
130
120
110
100
90
80
70
60
50
40
30
20
10
0
f1 (ppm)

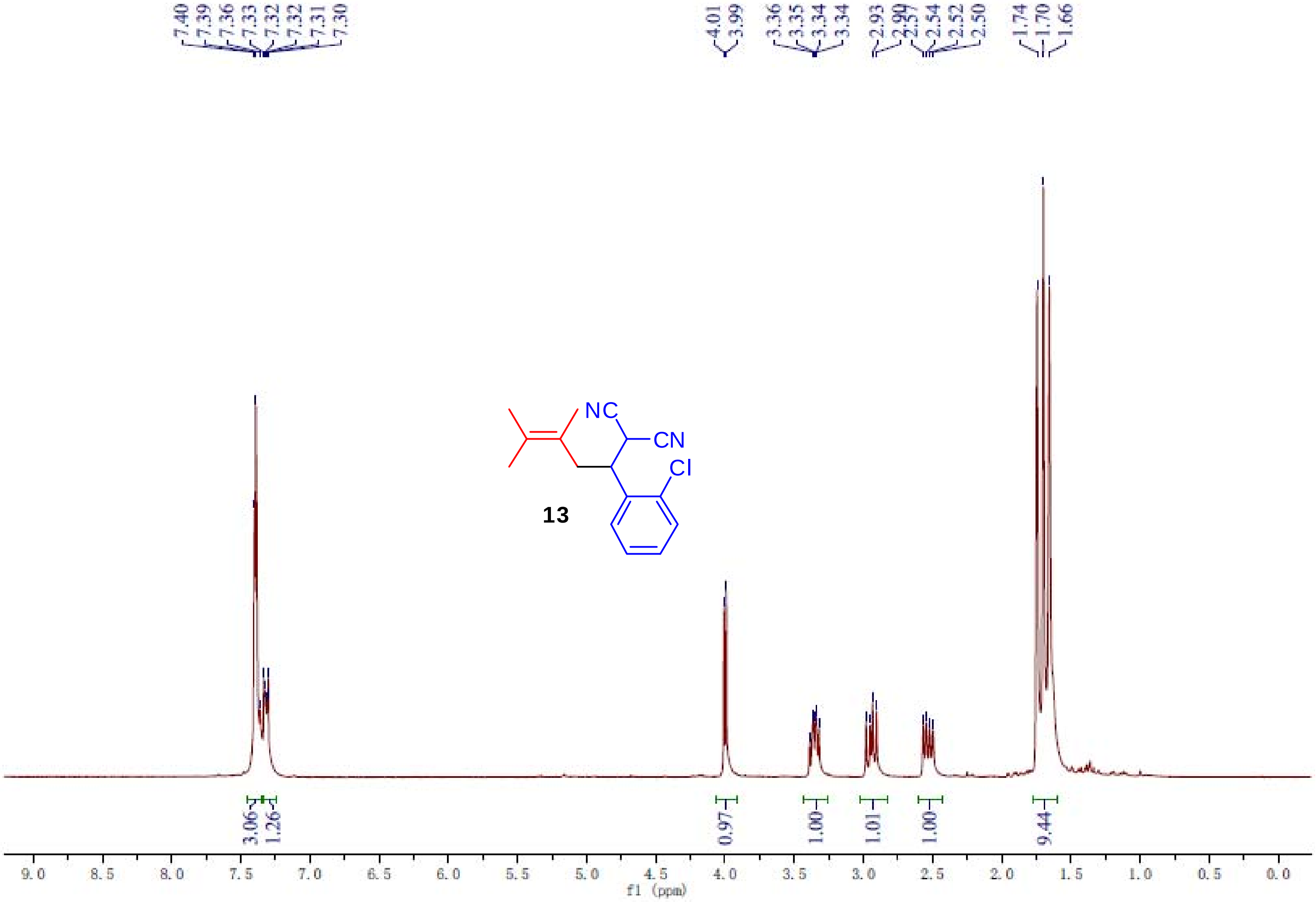
7.40
7.39
7.36
7.33
7.32
7.32
7.31
7.30
4.01
3.99
3.36
3.35
3.34
3.34
2.93
2.90
2.57
2.54
2.52
2.50
1.74
1.70
1.66
NC
CN
Cl
13
3.06
1.26
0.97
1.00
1.01
1.00
9.44
9.0
8.5
8.0
7.5
7.0
6.5
6.0
5.5
5.0
4.5
4.0
3.5
3.0
2.5
2.0
1.5
1.0
0.5
0.0
f1 (ppm)

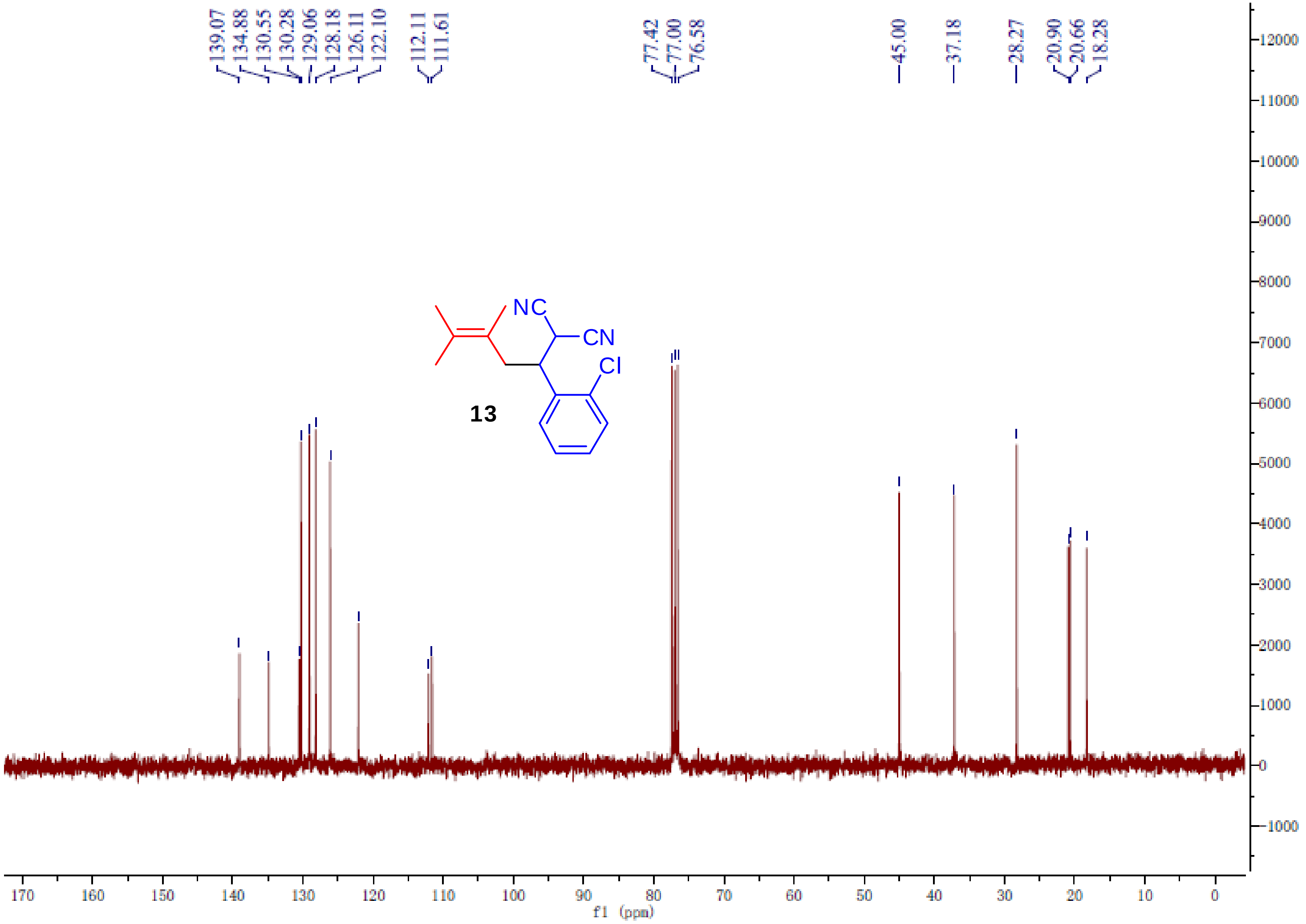
139.07
134.88
130.55
130.28
129.06
128.18
126.11
122.10
112.11
111.61
77.42
77.00
76.58
45.00
37.18
28.27
20.90
20.66
18.28
NC
CN
Cl
13
f1 (ppm)

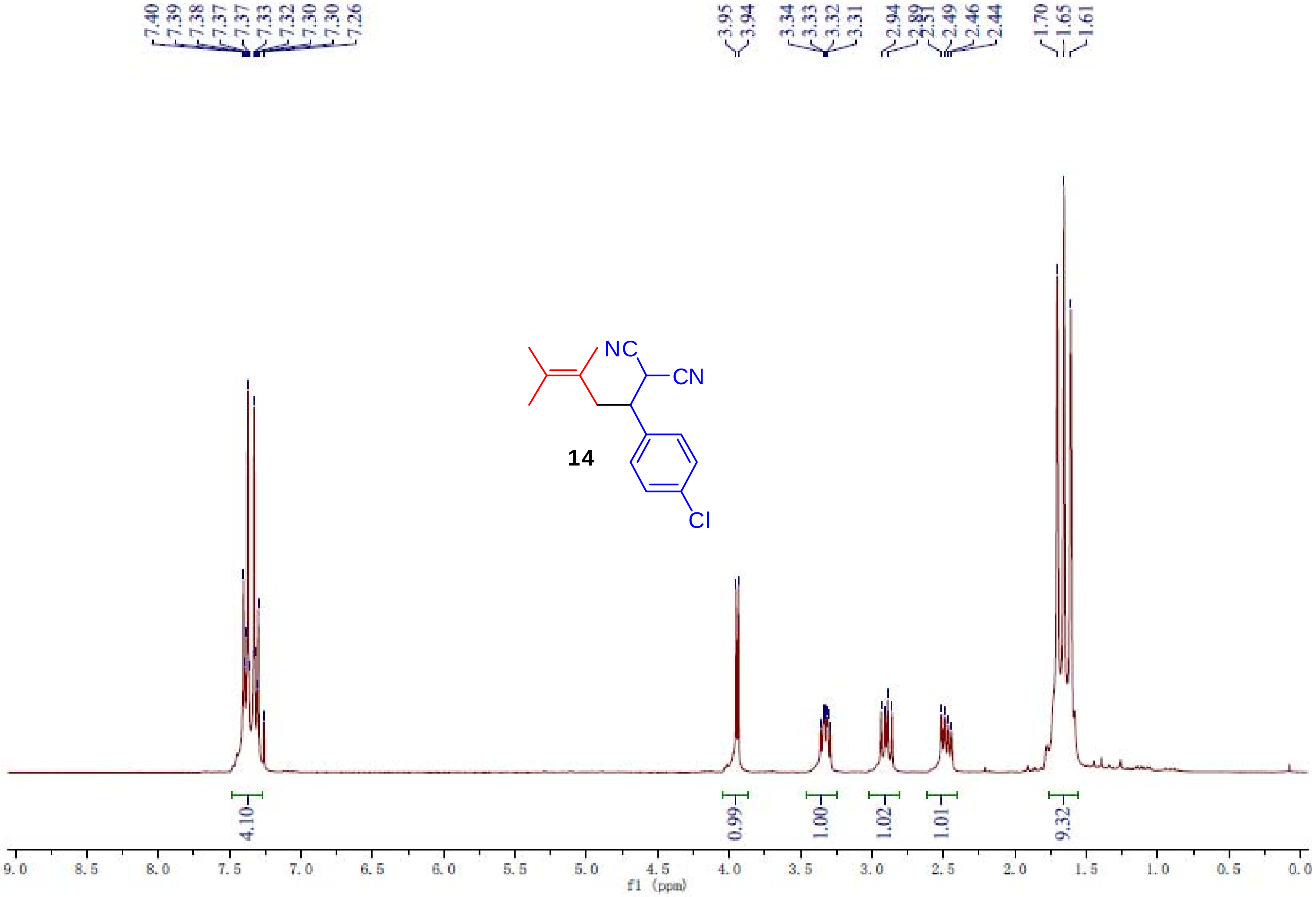
7.40 7.39 7.38 7.37 7.37 7.33 7.32 7.30 7.30 7.26
3.95 3.94
3.34 3.33 3.32 3.31
2.94 2.89 2.51 2.49 2.46 2.44
1.70 1.65 1.61
NC
CN
14
Cl
4.10
0.99
1.00
1.02
1.01
9.32
9.0 8.5 8.0 7.5 7.0 6.5 6.0 5.5 5.0 4.5 4.0 3.5 3.0 2.5 2.0 1.5 1.0 0.5 0.0
f1 (ppm)

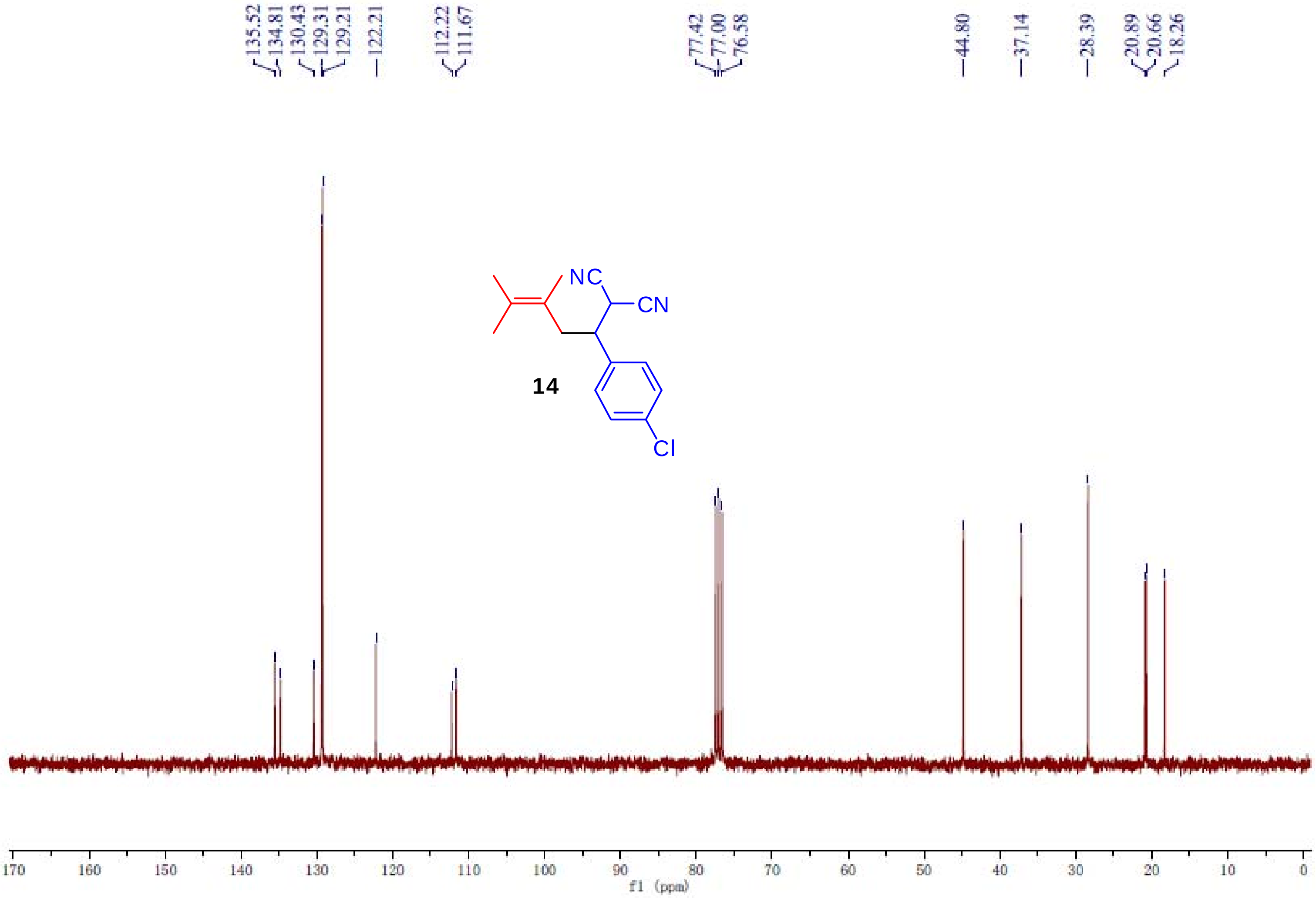
135.52
134.81
130.43
129.31
129.21
122.21
112.22
111.67
77.42
77.00
76.58
44.80
37.14
28.39
20.89
20.66
18.26
NC
CN
Cl
14
170 160 150 140 130 120 110 100 90 80 70 60 50 40 30 20 10 0
f1 (ppm)

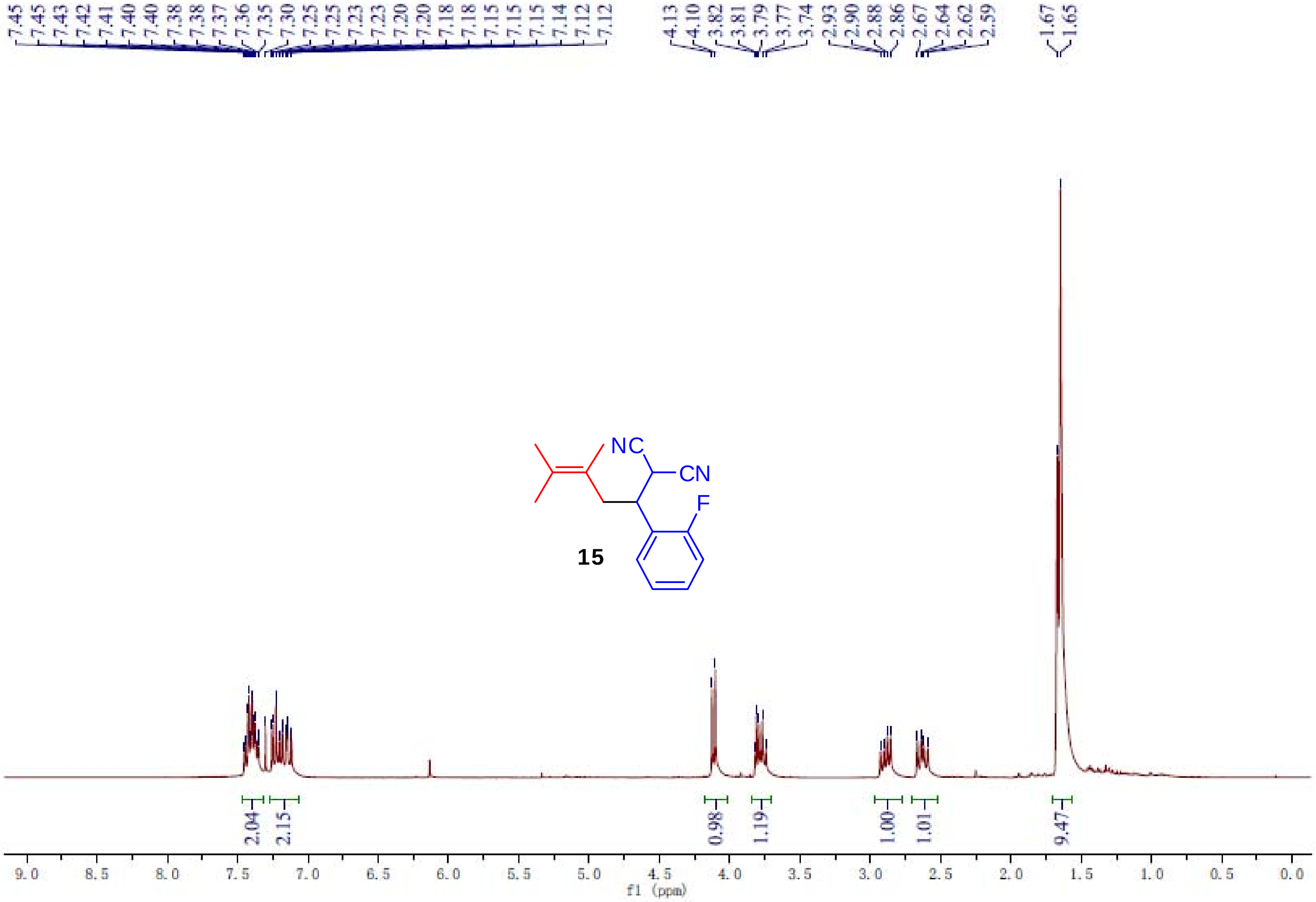
7.45 7.45 7.43 7.42 7.41 7.40 7.40 7.38 7.38 7.37 7.36 7.35 7.30 7.25 7.25 7.23 7.23 7.20 7.20 7.18 7.18 7.15 7.15 7.15 7.14 7.12 7.12
4.13 4.10 3.82 3.81 3.79 3.77 3.74 2.93 2.90 2.88 2.86 2.67 2.64 2.62 2.59
1.67 1.65
NC
CN
F
15
2.04 2.15 0.98 1.19 1.00 1.01 9.47
9.0 8.5 8.0 7.5 7.0 6.5 6.0 5.5 5.0 4.5 4.0 3.5 3.0 2.5 2.0 1.5 1.0 0.5 0.0
f1 (ppm)

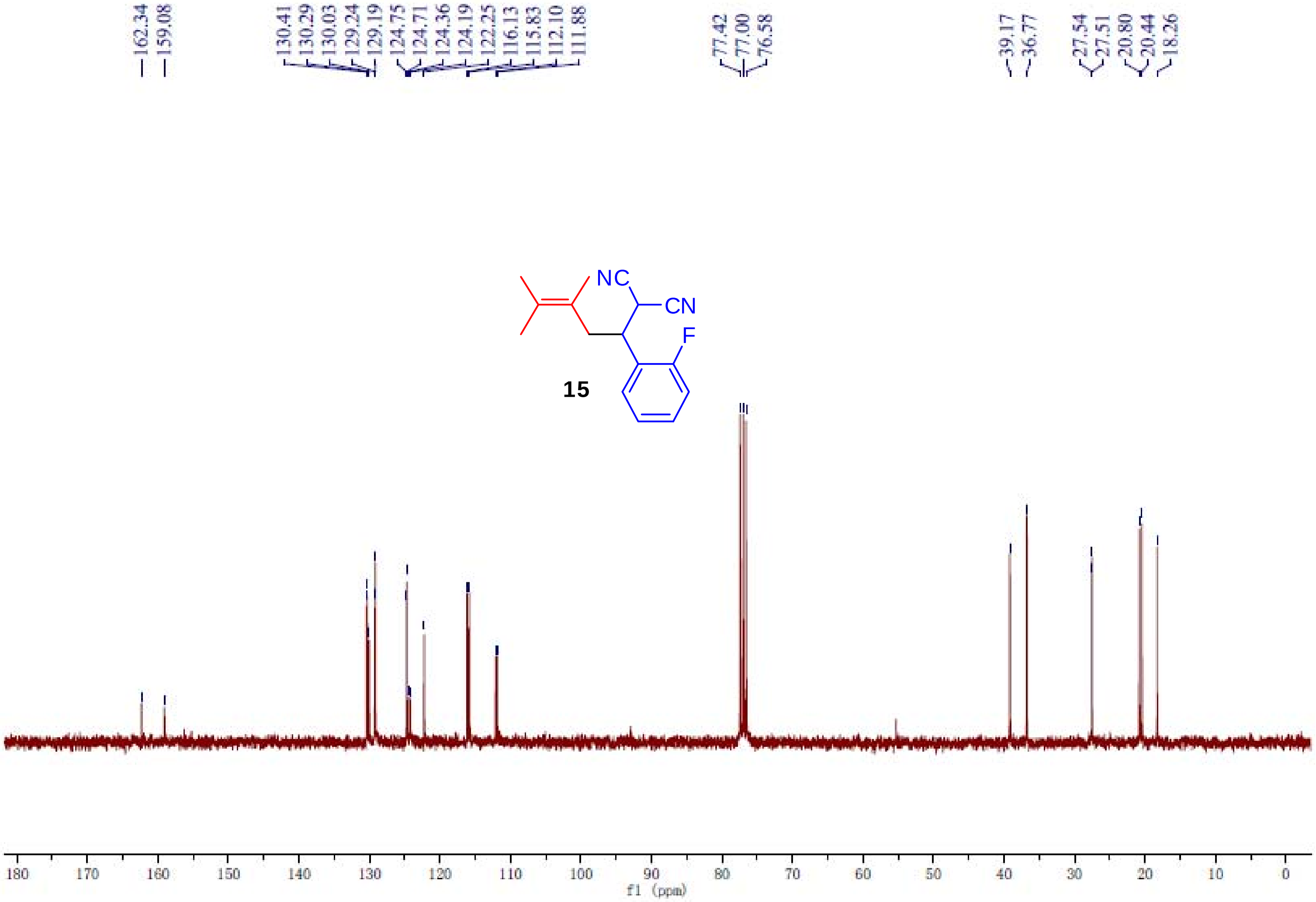
162.34
159.08
130.41
130.29
130.03
129.24
129.19
124.75
124.71
124.36
124.19
122.25
116.13
115.83
112.10
111.88
77.42
77.00
76.58
39.17
36.77
27.54
27.51
20.80
20.44
18.26
NC
CN
F
15
180
170
160
150
140
130
120
110
100
90
80
70
60
50
40
30
20
10
0
f1 (ppm)

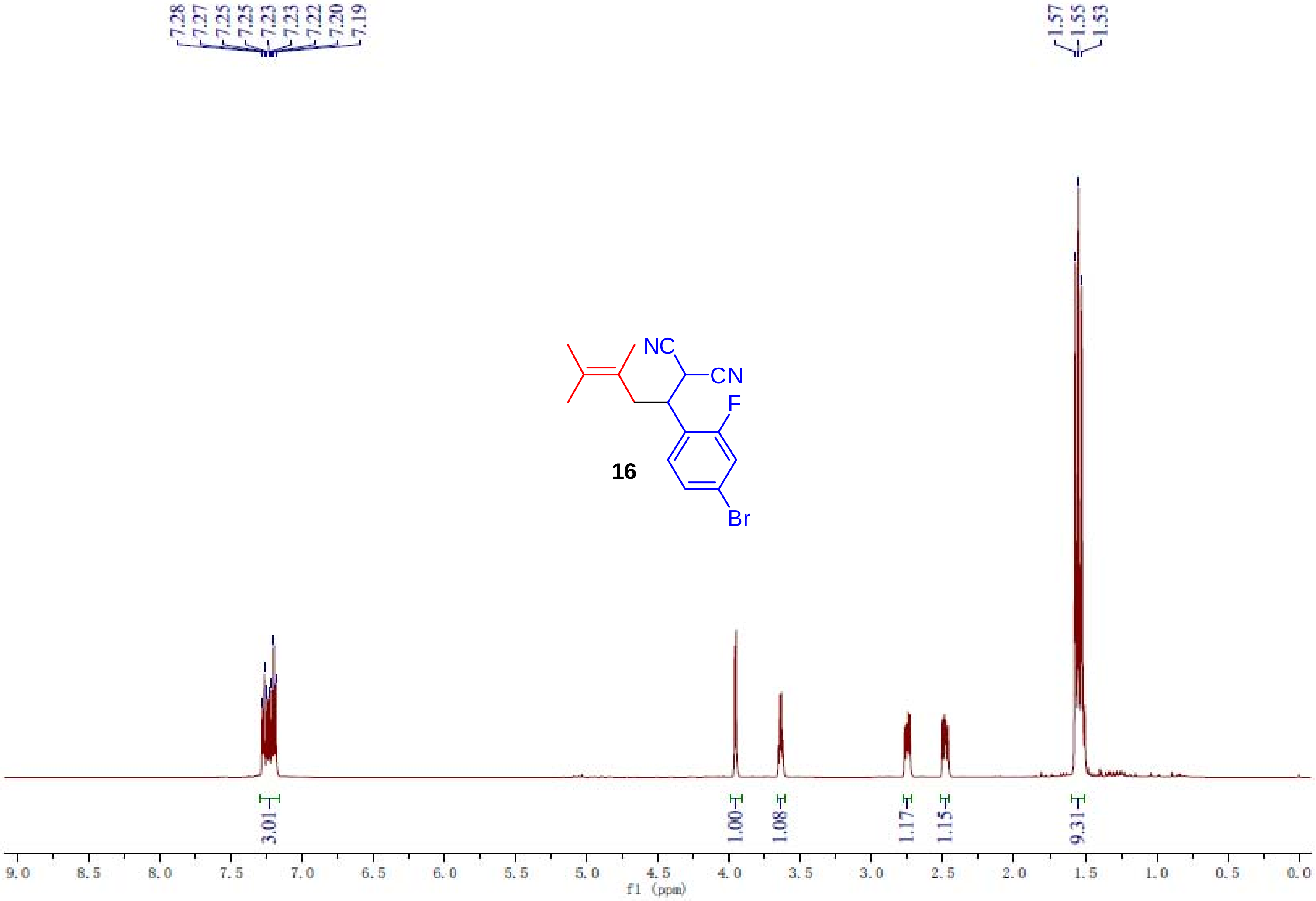

7.28
7.27
7.25
7.25
7.23
7.23
7.22
7.20
7.19
1.57
1.55
1.53
NC
CN
F
Br
16
3.01
1.00
1.08
1.17
1.15
9.31
9.0
8.5
8.0
7.5
7.0
6.5
6.0
5.5
5.0
4.5
4.0
3.5
3.0
2.5
2.0
1.5
1.0
0.5
0.0
f1 (ppm)

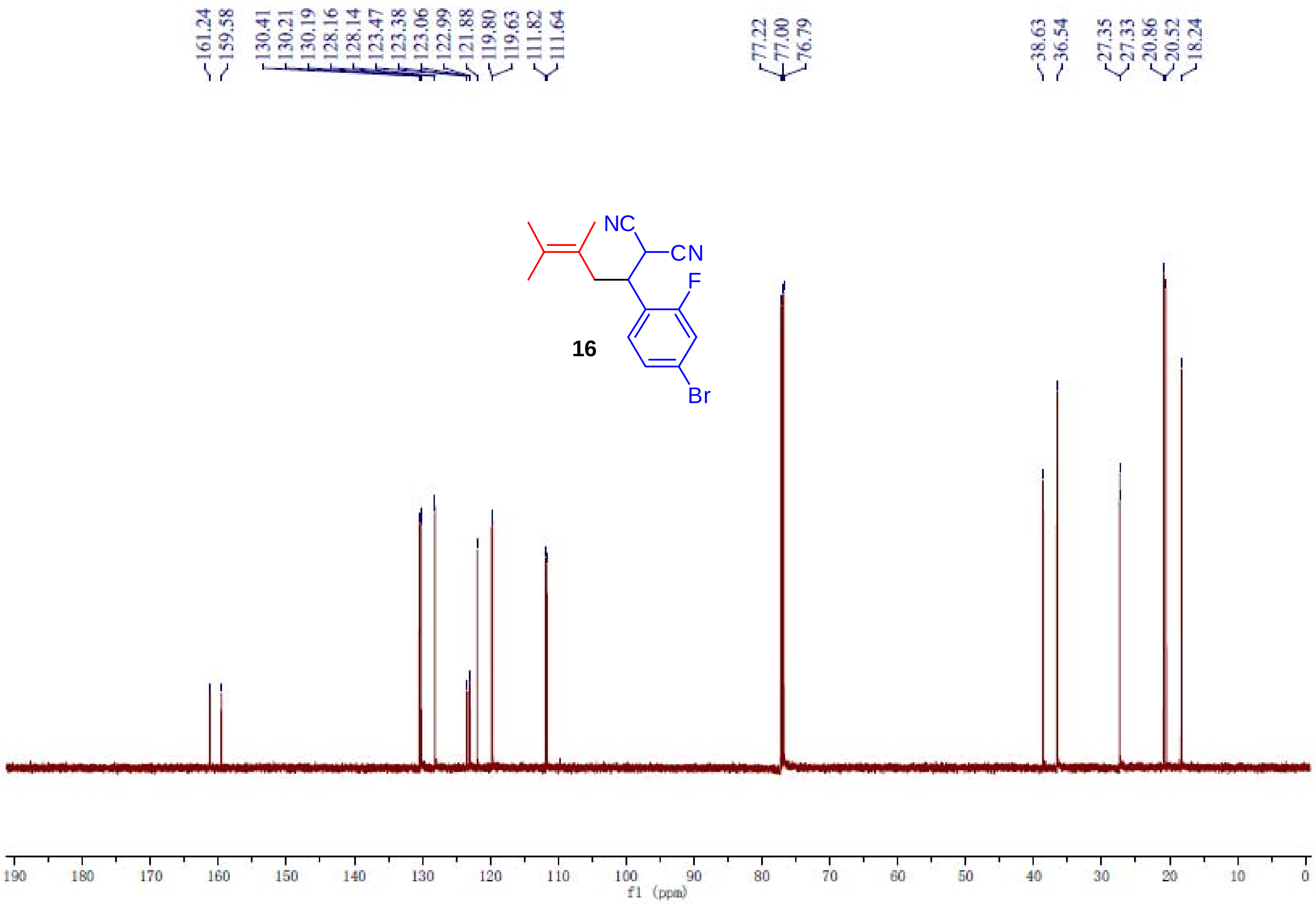
161.24
159.58
130.41
130.21
130.19
128.16
128.14
123.47
123.38
123.06
122.99
121.88
119.80
119.63
111.82
111.64
77.22
77.00
76.79
38.63
36.54
27.35
27.33
20.86
20.52
18.24
NC
CN
F
Br
16
f1 (ppm)

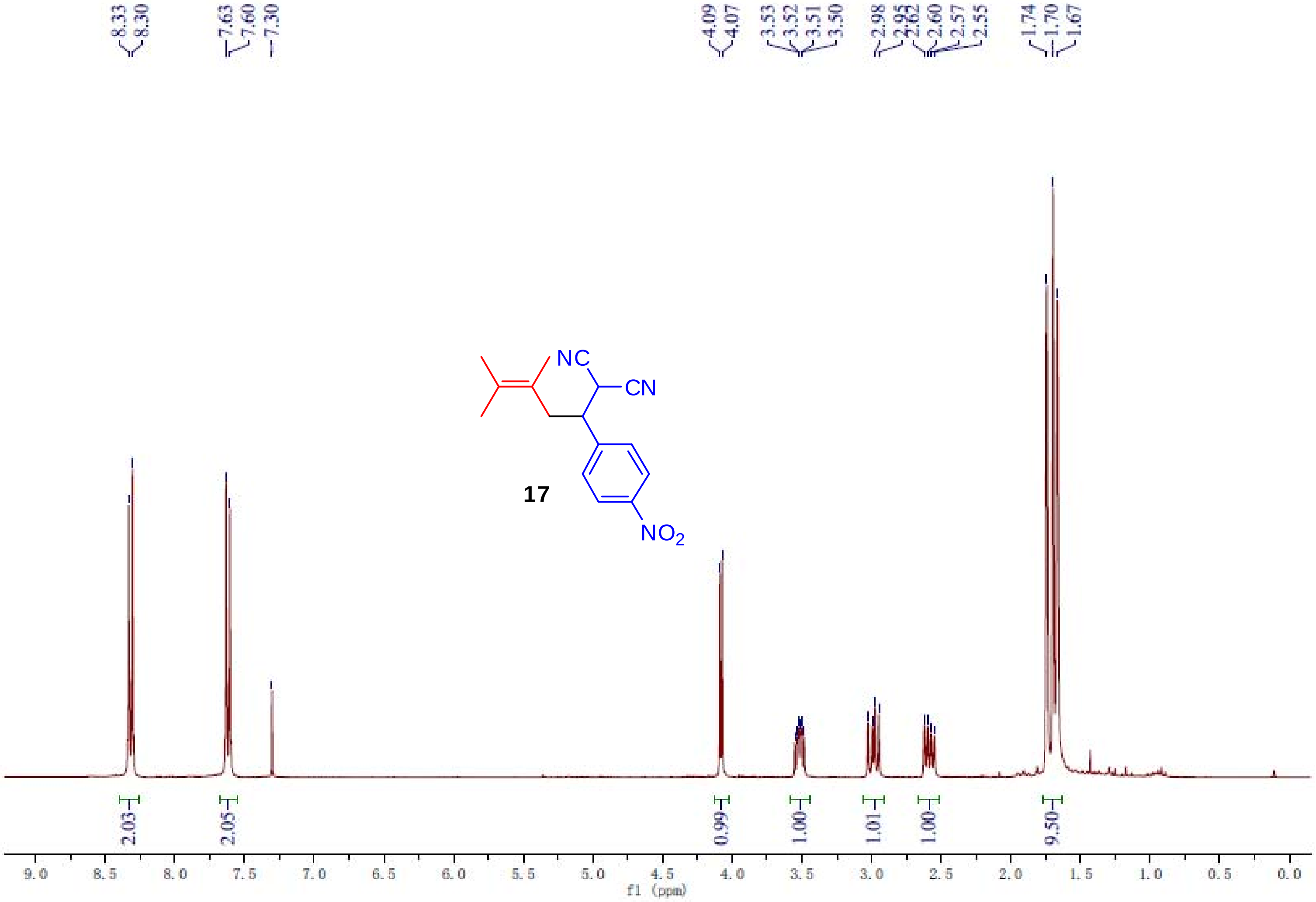
8.33
8.30
7.63
7.60
7.30
4.09
4.07
3.53
3.52
3.51
3.50
2.98
2.95
2.62
2.60
2.57
2.55
1.74
1.70
1.67
NC
CN
NO2
17
2.03
2.05
0.99
1.00
1.01
1.00
9.50
9.0
8.5
8.0
7.5
7.0
6.5
6.0
5.5
5.0
4.5
4.0
3.5
3.0
2.5
2.0
1.5
1.0
0.5
0.0
f1 (ppm)

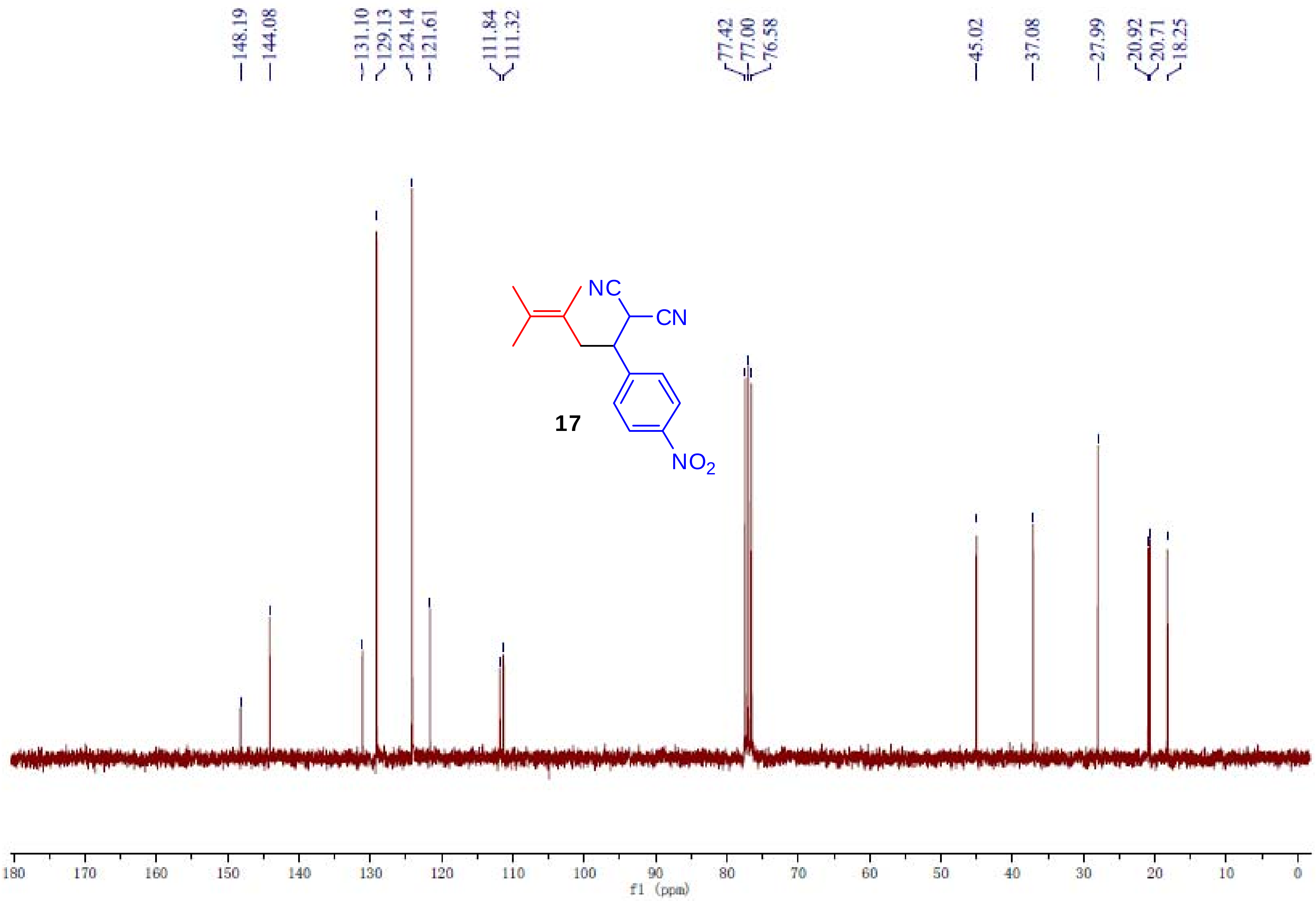
148.19
144.08
131.10
129.13
124.14
121.61
111.84
111.32
77.42
77.00
76.58
45.02
37.08
27.99
20.92
20.71
18.25
NC
CN
NO2
17
180 170 160 150 140 130 120 110 100 90 80 70 60 50 40 30 20 10 0
f1 (ppm)

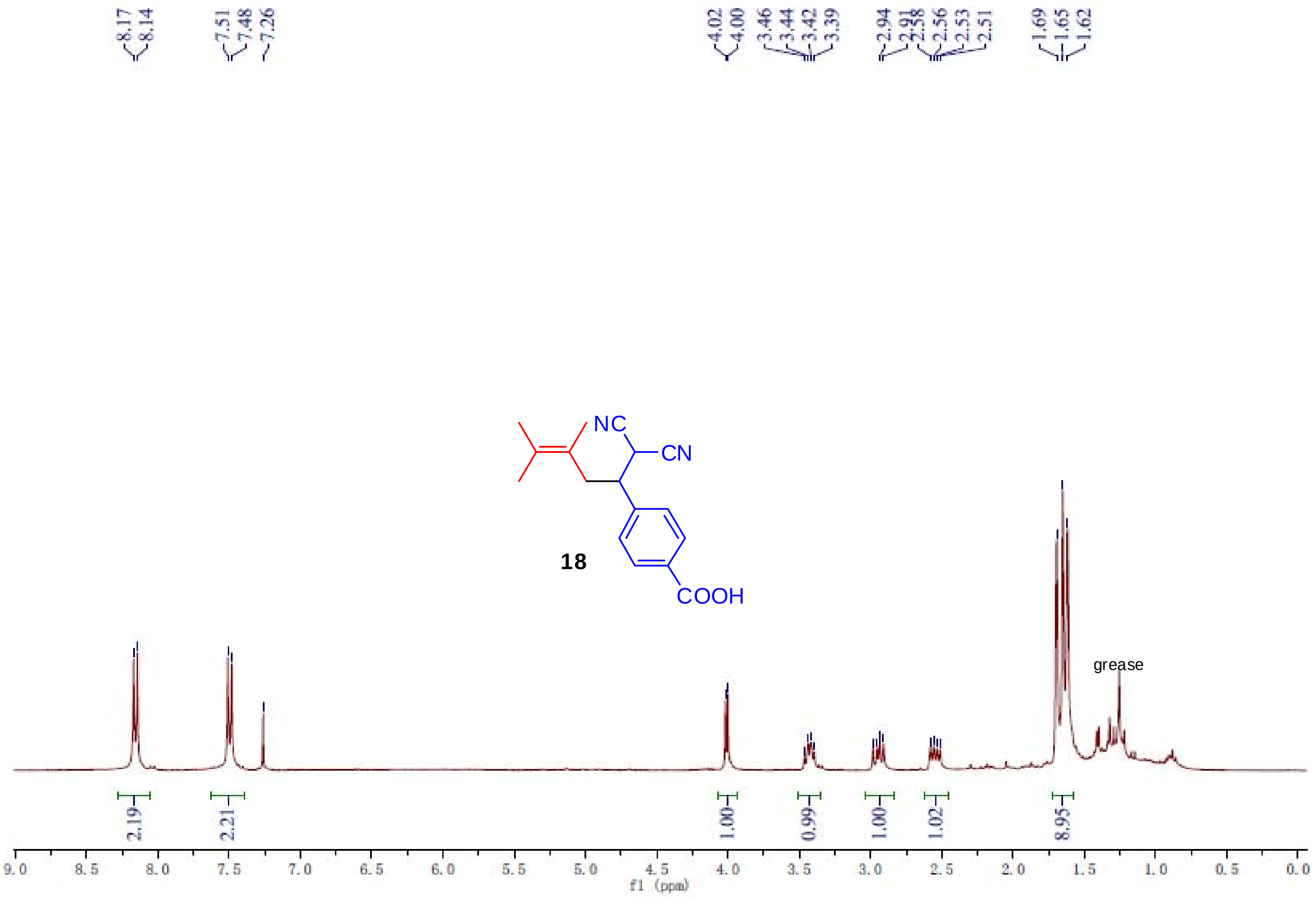

8.17
8.14
7.51
7.48
7.26
4.02
4.00
3.46
3.44
3.42
3.39
2.94
2.91
2.58
2.56
2.53
2.51
1.69
1.65
1.62
NC
CN
18
COOH
grease
2.19
2.21
1.00
0.99
1.00
1.02
8.95
9.0
8.5
8.0
7.5
7.0
6.5
6.0
5.5
5.0
4.5
4.0
3.5
3.0
2.5
2.0
1.5
1.0
0.5
0.0
f1 (ppm)

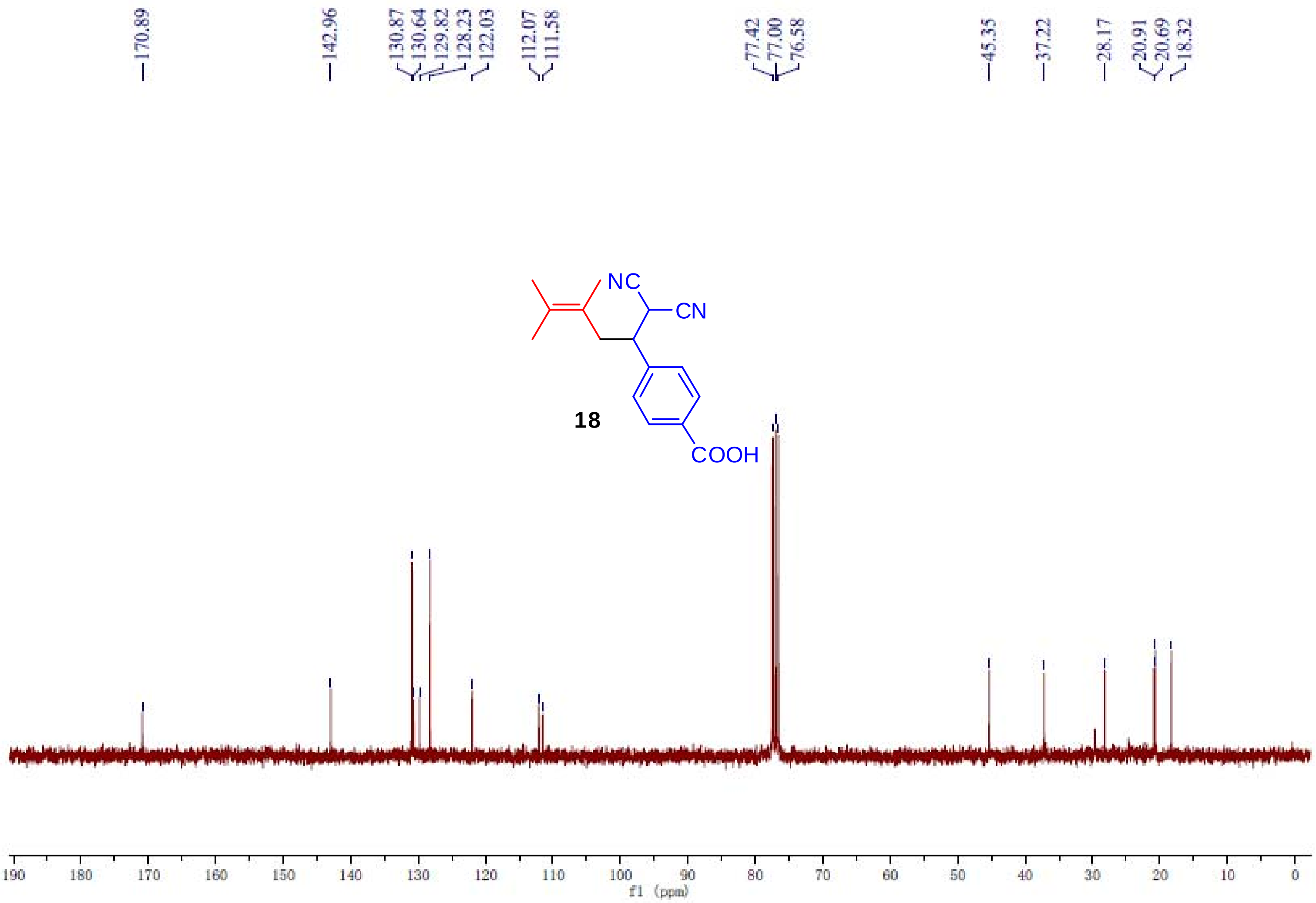

170.89
142.96
130.87
130.64
129.82
128.23
122.03
112.07
111.58
77.42
77.00
76.58
45.35
37.22
28.17
20.91
20.69
18.32
NC
CN
COOH
18
190 180 170 160 150 140 130 120 110 100 90 80 70 60 50 40 30 20 10 0
f1 (ppm)

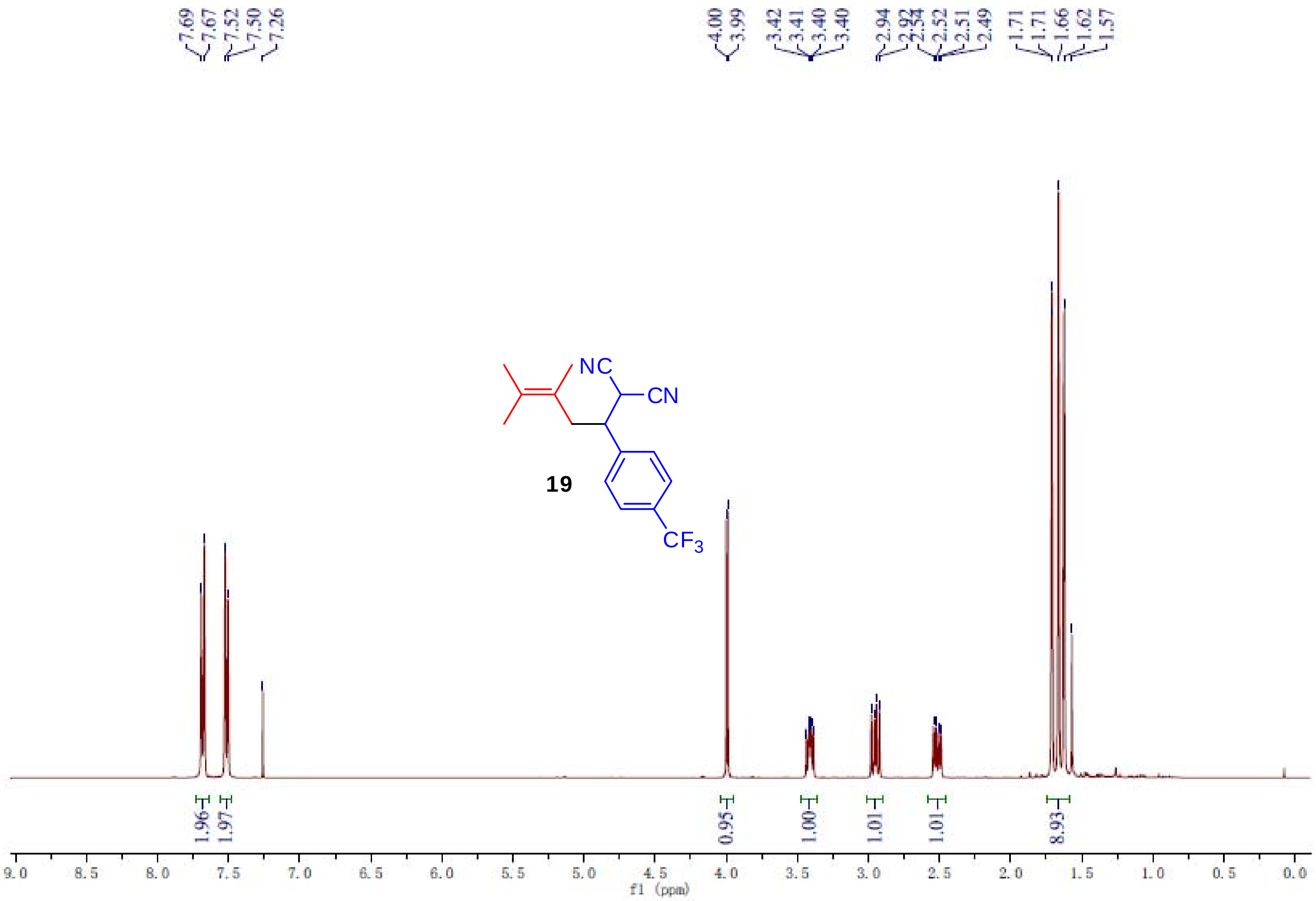
7.69
7.67
7.52
7.50
7.26
4.00
3.99
3.42
3.41
3.40
3.40
2.94
2.92
2.54
2.52
2.51
2.49
1.71
1.71
1.66
1.62
1.57
NC
CN
CF3
19
1.96
1.97
0.95
1.00
1.01
1.01
8.93
9.0
8.5
8.0
7.5
7.0
6.5
6.0
5.5
5.0
4.5
4.0
3.5
3.0
2.5
2.0
1.5
1.0
0.5
0.0
f1 (ppm)

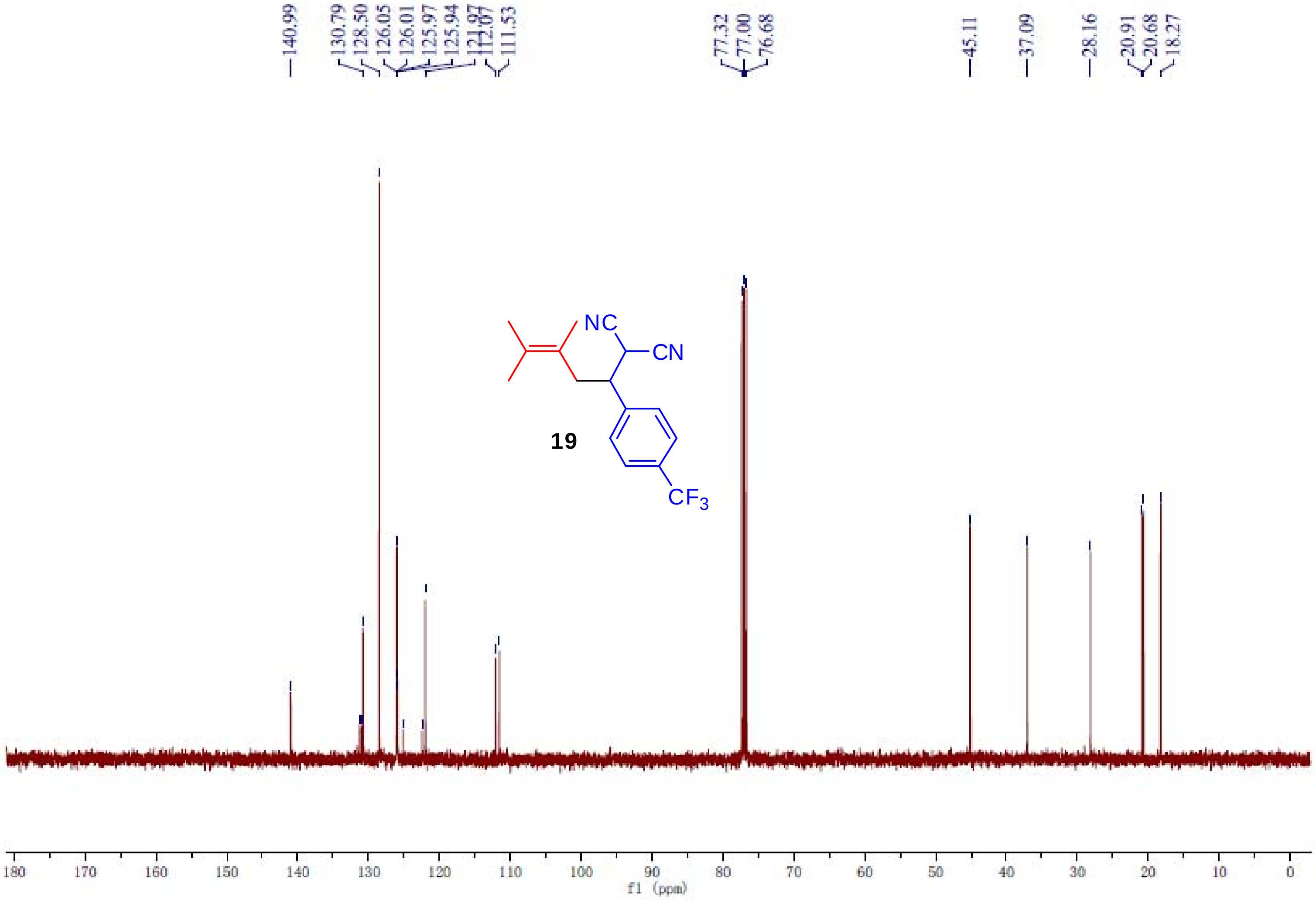
140.99
130.79
128.50
126.05
126.01
125.97
125.94
121.97
112.07
111.53
77.32
77.00
76.68
45.11
37.09
28.16
20.91
20.68
18.27
NC
CN
CF3
19
f1 (ppm)

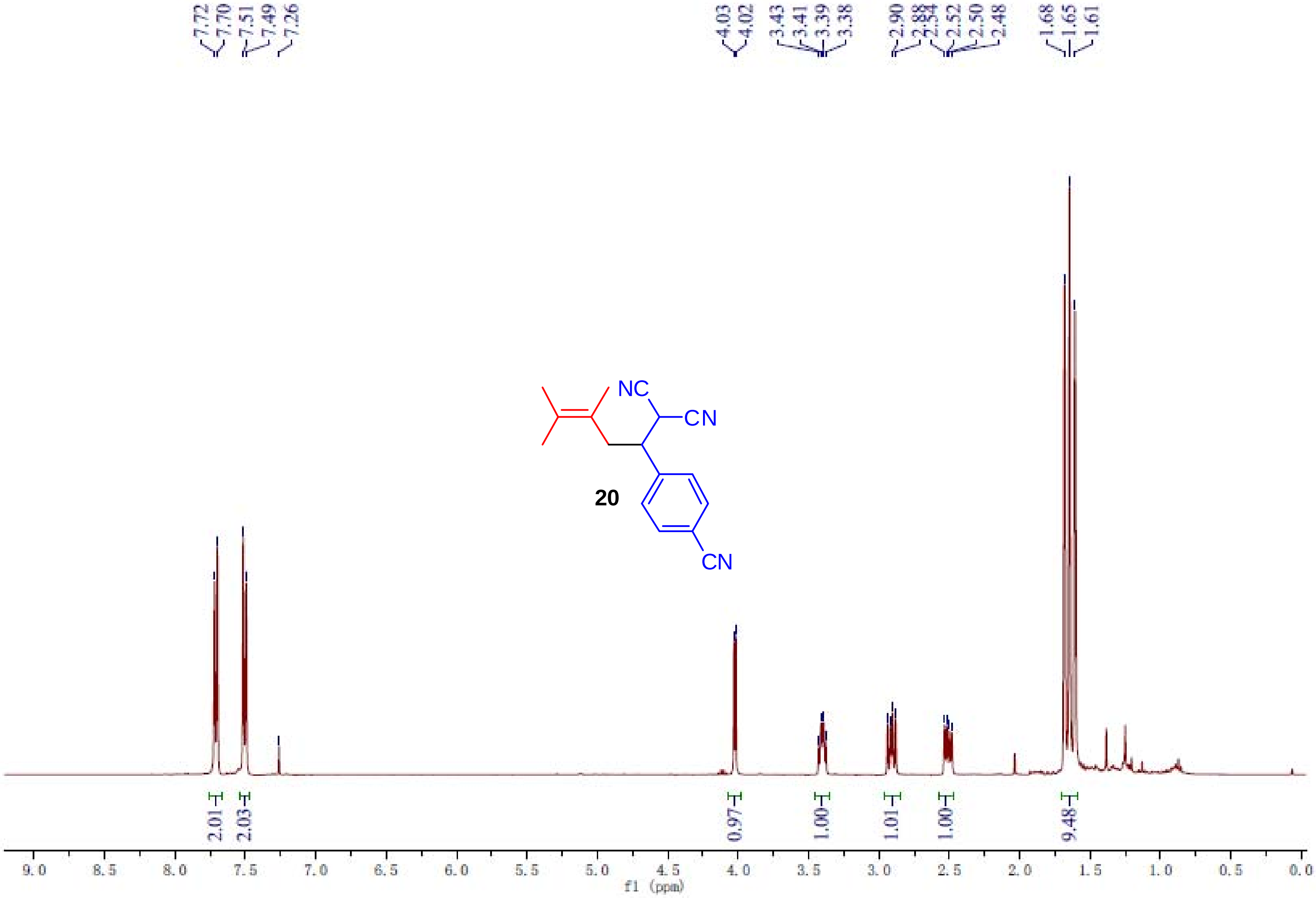
7.72
7.70
7.51
7.49
7.26
4.03
4.02
3.43
3.41
3.39
3.38
2.90
2.88
2.54
2.52
2.50
2.48
1.68
1.65
1.61
NC
CN
20
CN
2.01
2.03
0.97
1.00
1.01
1.00
9.48
9.0
8.5
8.0
7.5
7.0
6.5
6.0
5.5
5.0
4.5
4.0
3.5
3.0
2.5
2.0
1.5
1.0
0.5
0.0
f1 (ppm)

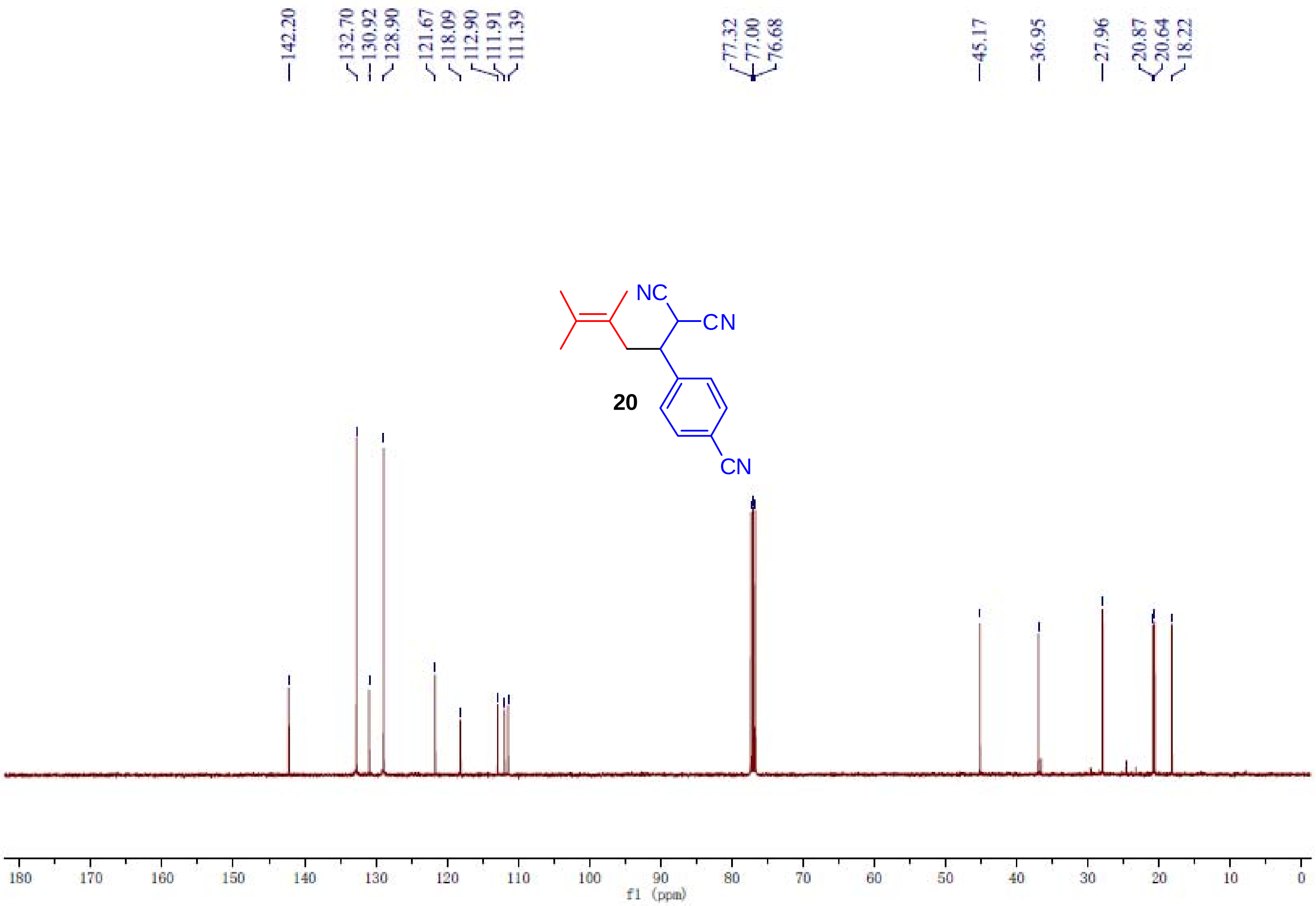
142.20
132.70
130.92
128.90
121.67
118.09
112.90
111.91
111.39
77.32
77.00
76.68
45.17
36.95
27.96
20.87
20.64
18.22
NC
CN
CN
20
180 170 160 150 140 130 120 110 100 90 80 70 60 50 40 30 20 10 0
f1 (ppm)

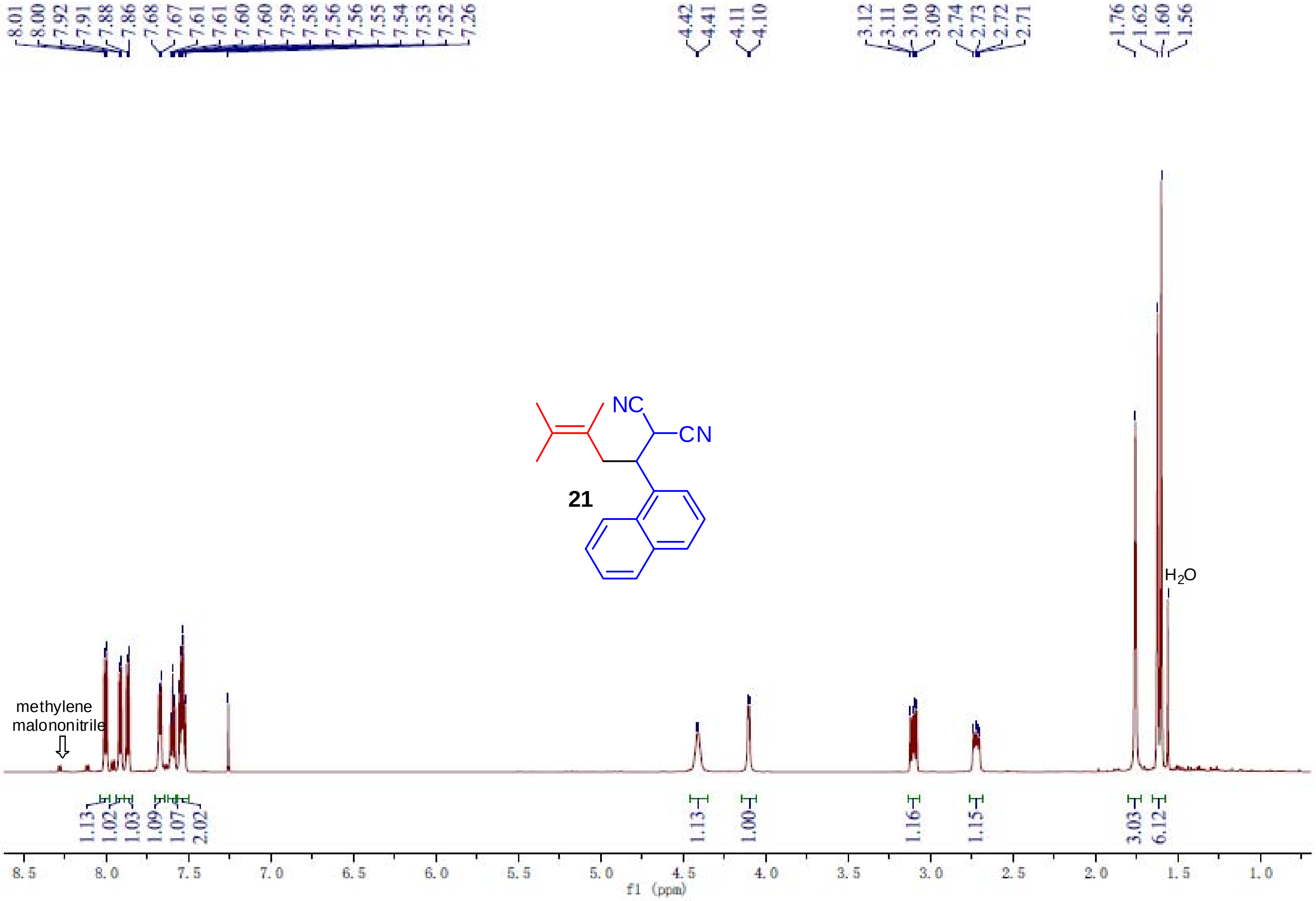

8.01 8.00 7.92 7.91 7.88 7.86 7.68 7.67 7.61 7.61 7.60 7.60 7.59 7.58 7.56 7.56 7.55 7.54 7.53 7.52 7.26
4.42 4.41 4.11 4.10
3.12 3.11 3.10 3.09 2.74 2.73 2.72 2.71
1.76 1.62 1.60 1.56
NC
CN
21
H2O
methylene malononitrile
1.13 1.02 1.03 1.09 1.07 2.02 1.13 1.00 1.16 1.15 3.03 6.12
8.5 8.0 7.5 7.0 6.5 6.0 5.5 5.0 4.5 4.0 3.5 3.0 2.5 2.0 1.5 1.0
f1 (ppm)

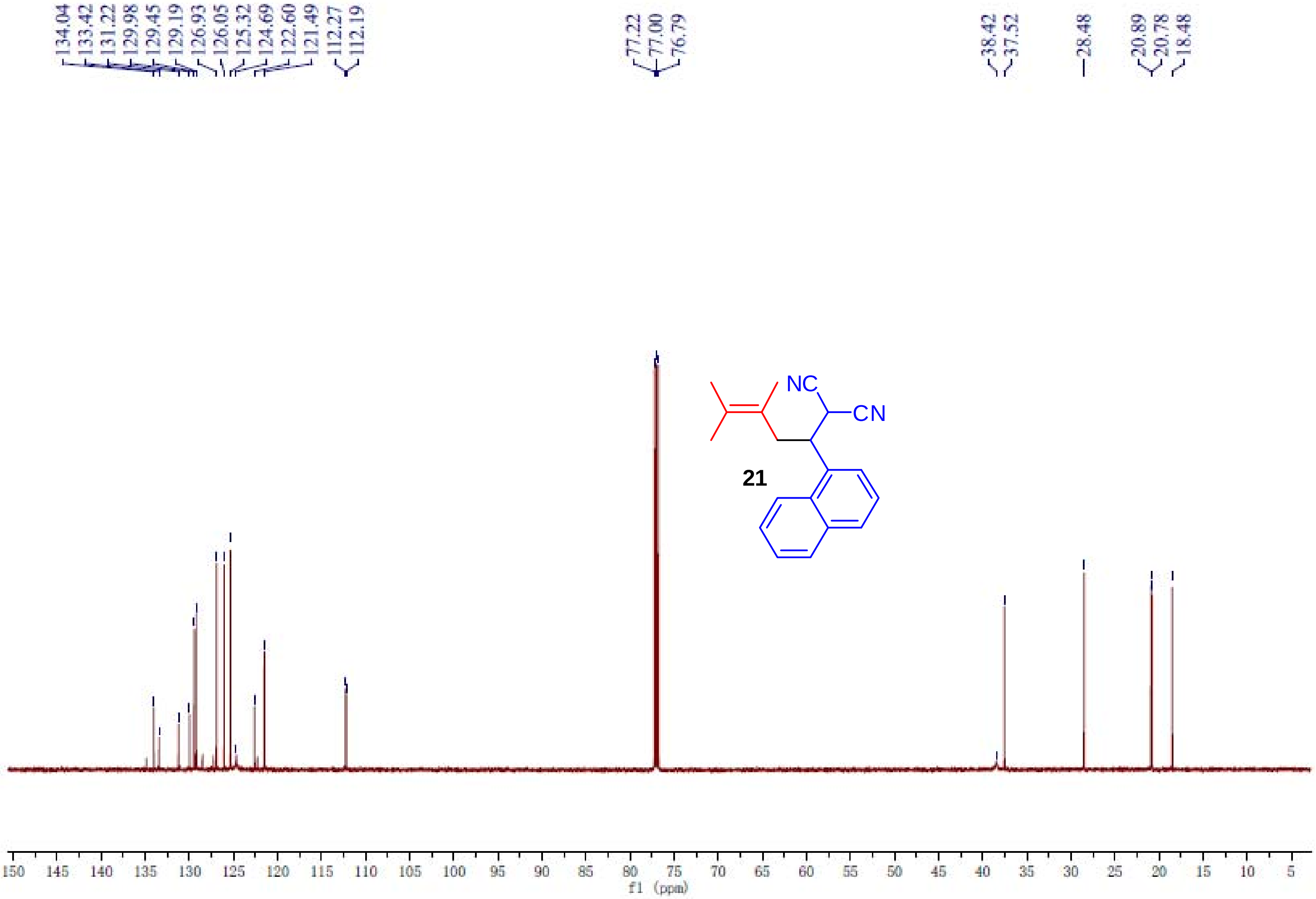
134.04
133.42
131.22
129.98
129.45
129.19
126.93
126.05
125.32
124.69
122.60
121.49
112.27
112.19
77.22
77.00
76.79
38.42
37.52
28.48
20.89
20.78
18.48
NC
CN
21
150 145 140 135 130 125 120 115 110 105 100 95 90 85 80 75 70 65 60 55 50 45 40 35 30 25 20 15 10 5
f1 (ppm)

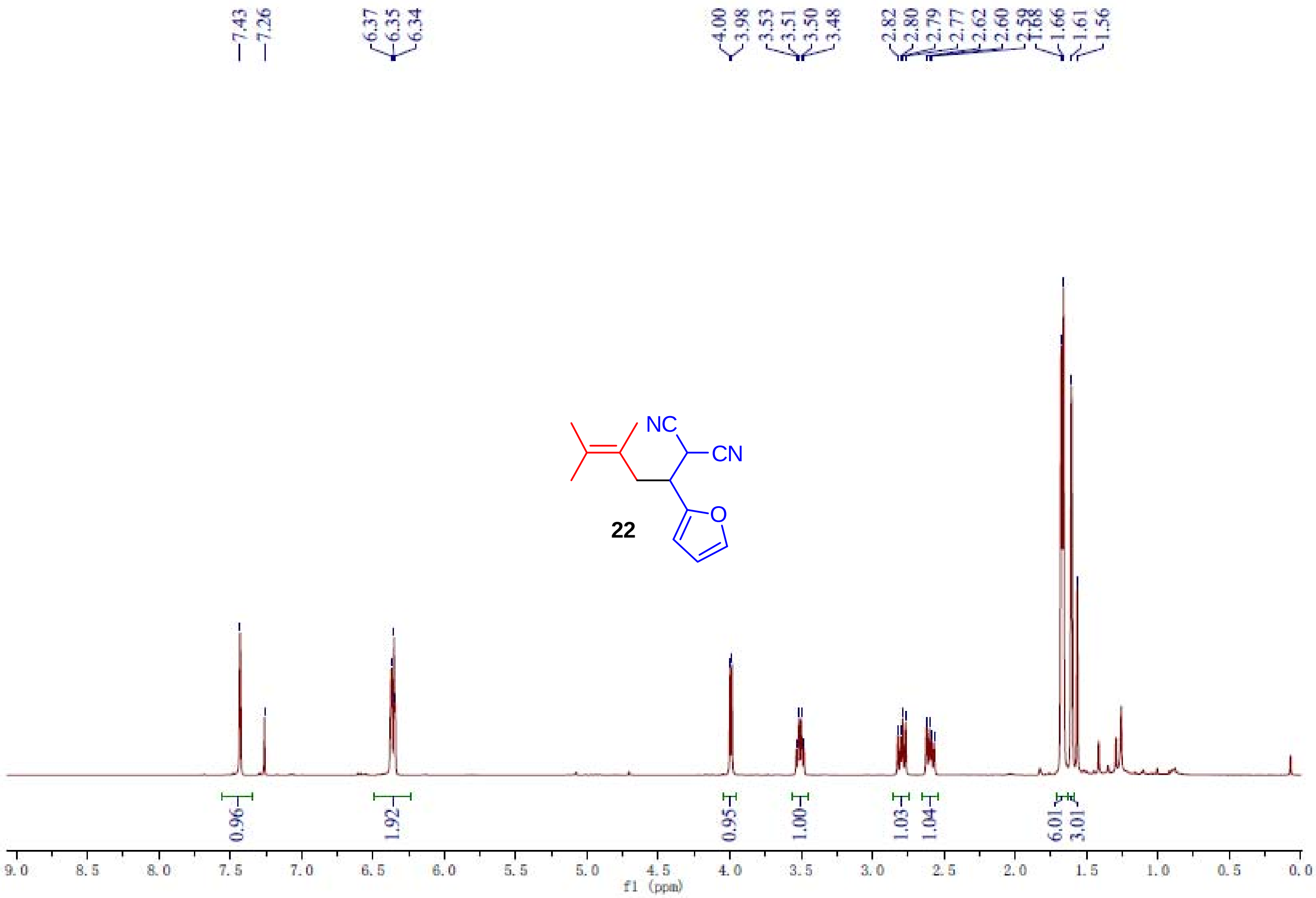
7.43
7.26
6.37
6.35
6.34
4.00
3.98
3.53
3.51
3.50
3.48
2.82
2.80
2.79
2.77
2.62
2.60
2.59
1.68
1.66
1.61
1.56
NC
CN
O
22
0.96
1.92
0.95
1.00
1.03
1.04
6.01
3.01
9.0
8.5
8.0
7.5
7.0
6.5
6.0
5.5
5.0
4.5
4.0
3.5
3.0
2.5
2.0
1.5
1.0
0.5
0.0
f1 (ppm)

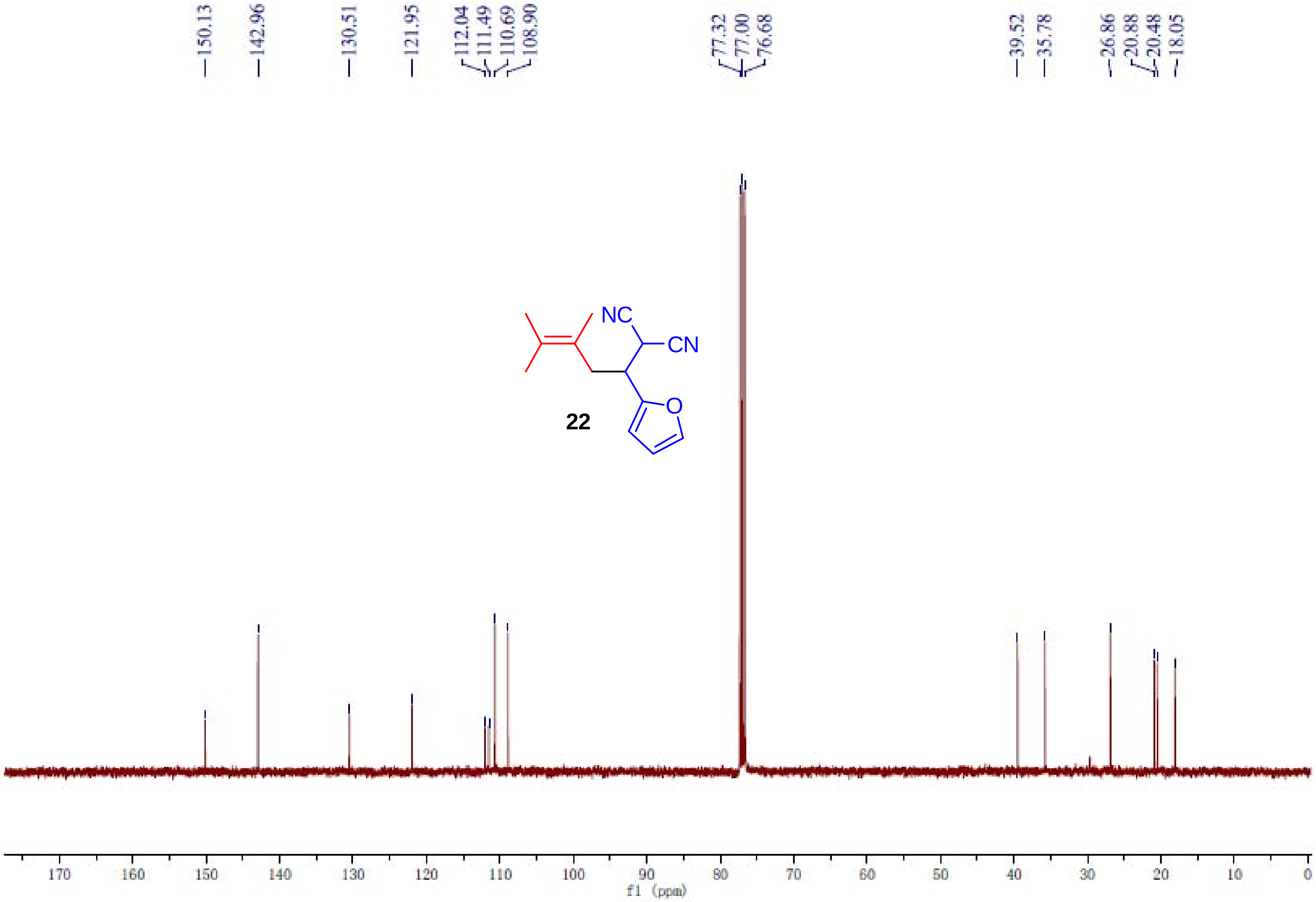
150.13
142.96
130.51
121.95
112.04
111.49
110.69
108.90
77.32
77.00
76.68
39.52
35.78
26.86
20.88
20.48
18.05
NC
CN
O
22
170 160 150 140 130 120 110 100 90 80 70 60 50 40 30 20 10 0
f1 (ppm)

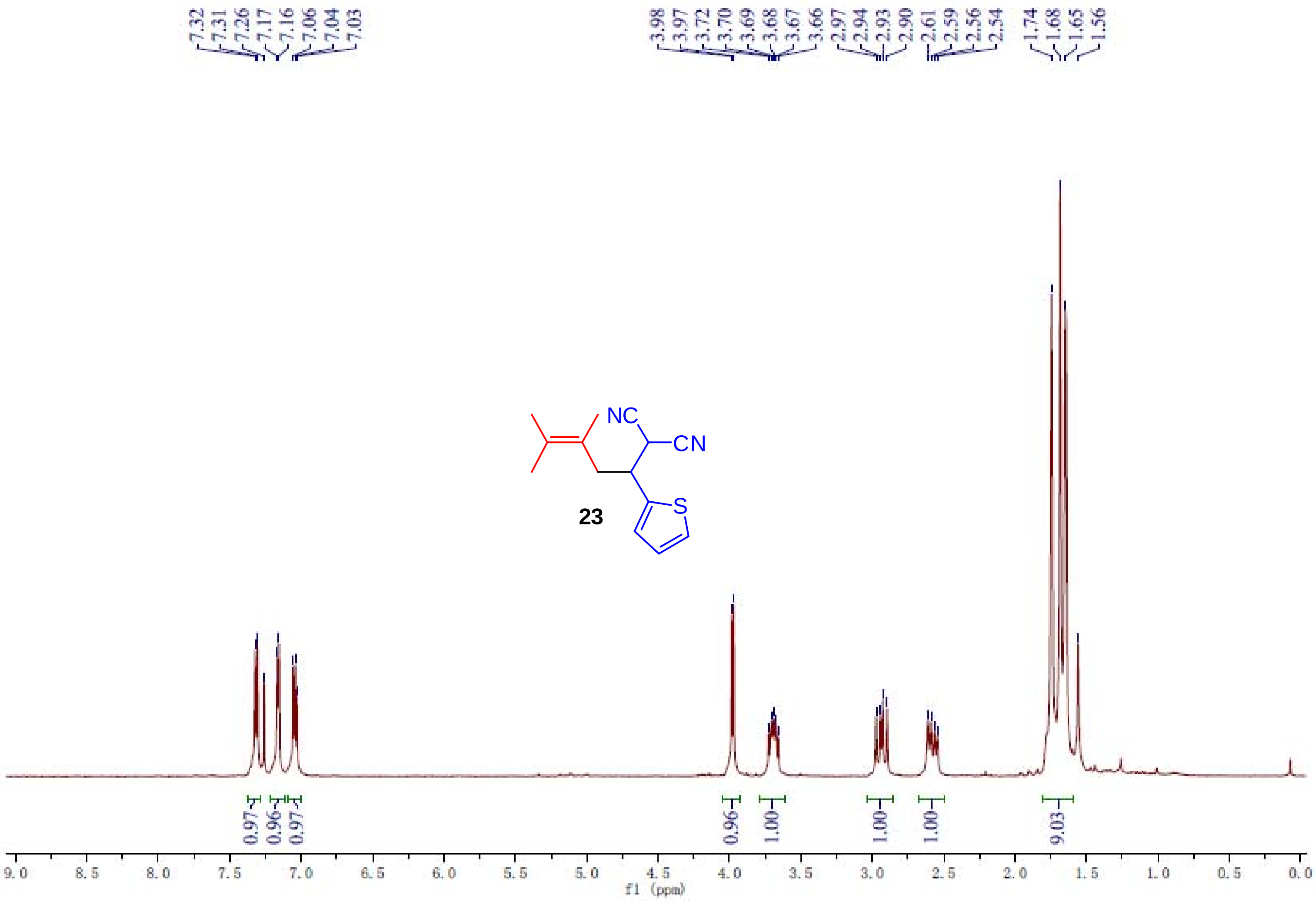
7.32
7.31
7.26
7.17
7.16
7.06
7.04
7.03
3.98
3.97
3.72
3.70
3.69
3.68
3.67
3.66
2.97
2.94
2.93
2.90
2.61
2.59
2.56
2.54
1.74
1.68
1.65
1.56
NC
CN
S
23
0.97
0.96
0.97
0.96
1.00
1.00
1.00
9.03
f1 (ppm)

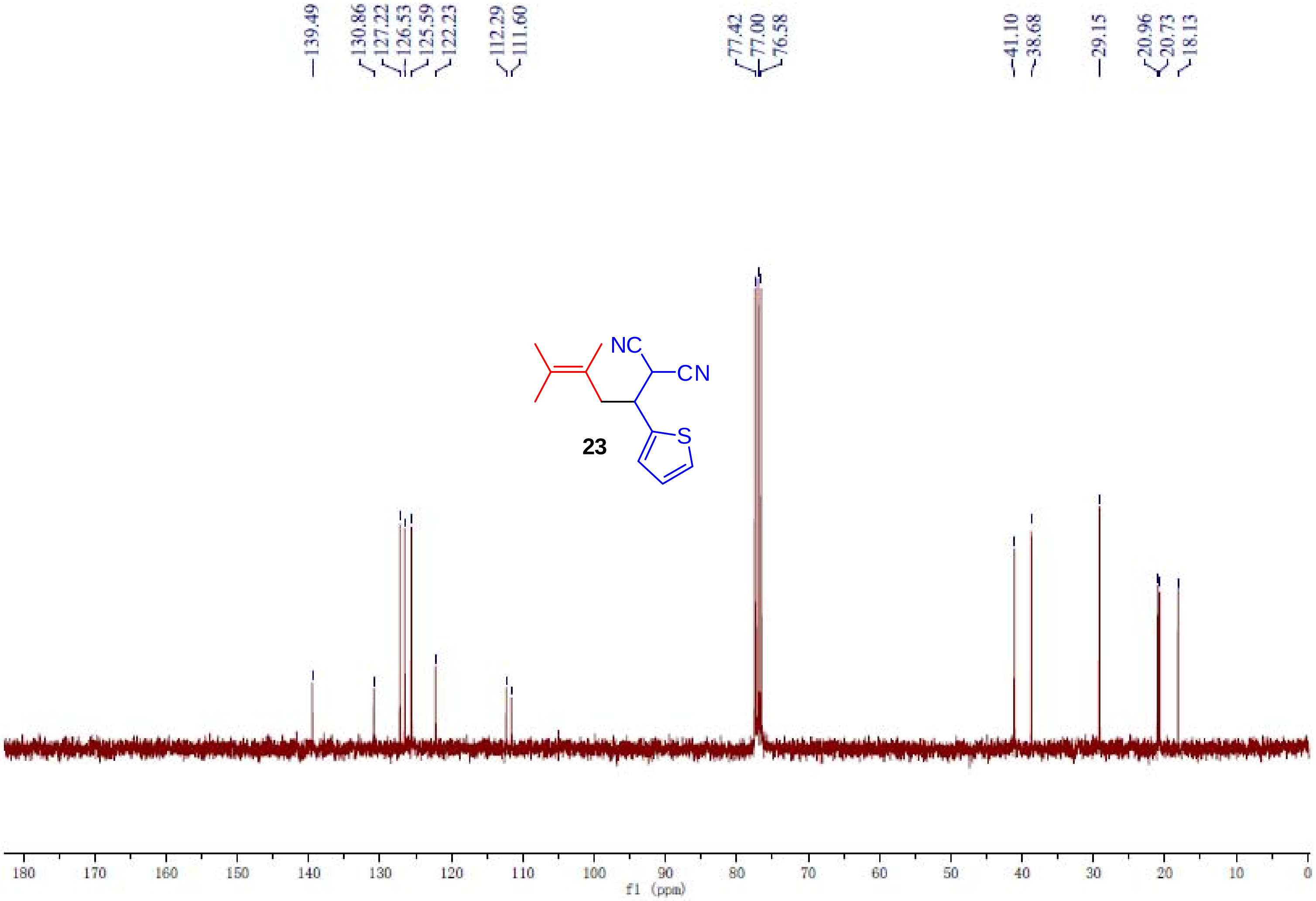
139.49
130.86
127.22
126.53
125.59
122.23
112.29
111.60
77.42
77.00
76.58
41.10
38.68
29.15
20.96
20.73
18.13
NC
CN
S
23
180 170 160 150 140 130 120 110 100 90 80 70 60 50 40 30 20 10 0
f1 (ppm)

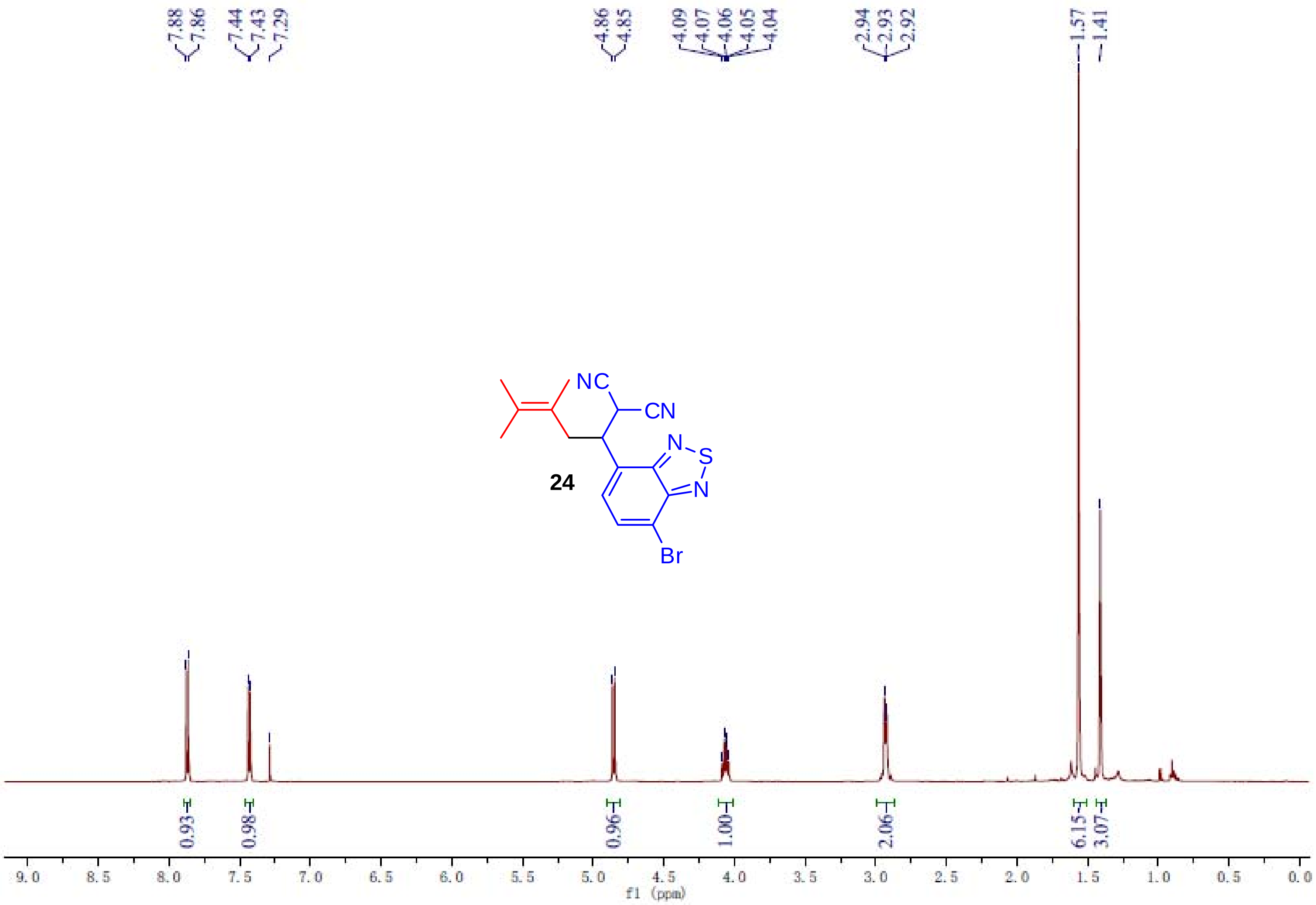
7.88
7.86
7.44
7.43
7.29
4.86
4.85
4.09
4.07
4.06
4.05
4.04
2.94
2.93
2.92
1.57
1.41
NC
CN
N
S
N
Br
0.93
0.98
0.96
1.00
2.06
6.15
3.07
9.0 8.5 8.0 7.5 7.0 6.5 6.0 5.5 5.0 4.5 4.0 3.5 3.0 2.5 2.0 1.5 1.0 0.5 0.0
f1 (ppm)

24

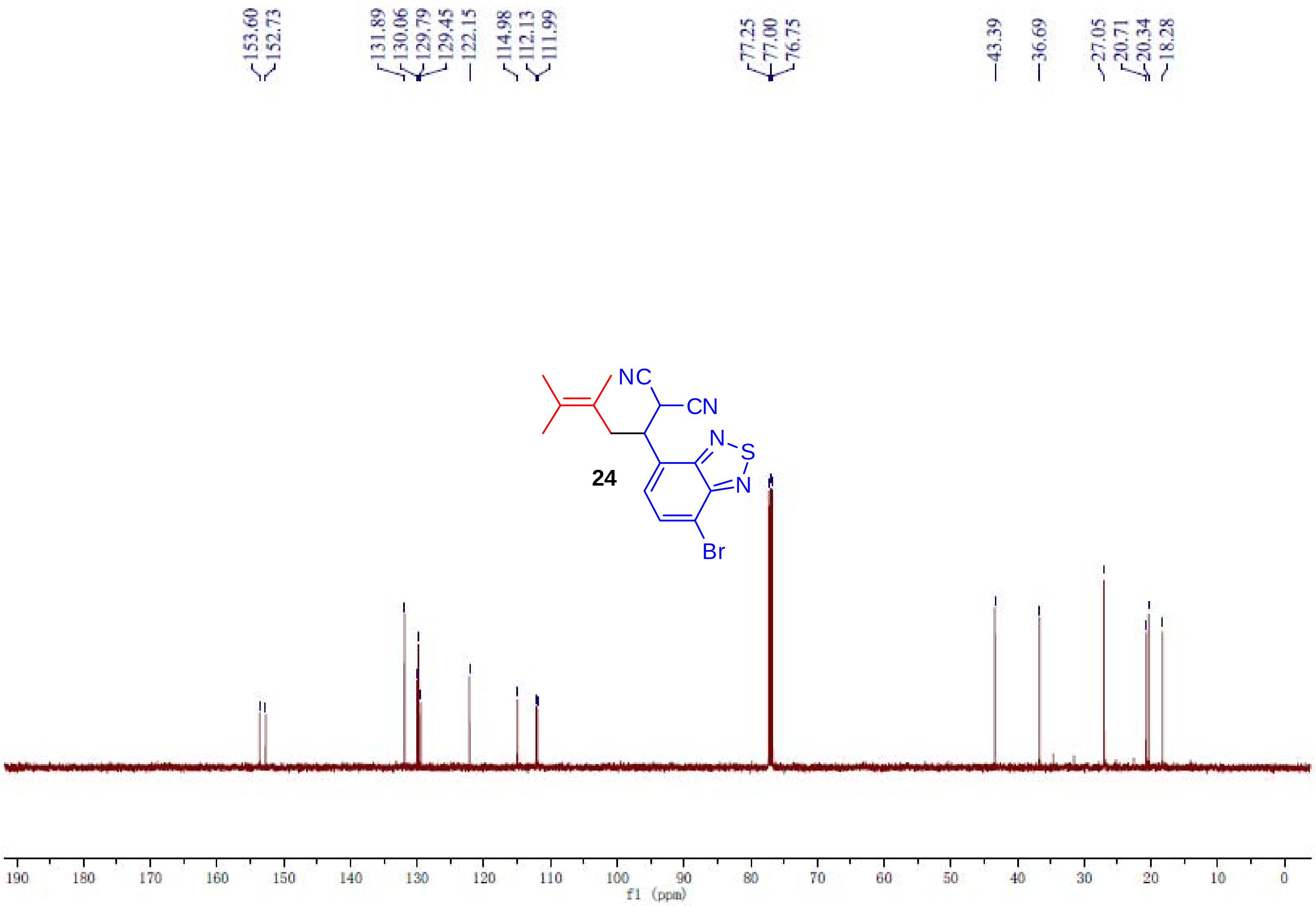

153.60
152.73
131.89
130.06
129.79
129.45
122.15
114.98
112.13
111.99
77.25
77.00
76.75
43.39
36.69
27.05
20.71
20.34
18.28
NC
CN
N
S
N
Br
24
190 180 170 160 150 140 130 120 110 100 90 80 70 60 50 40 30 20 10 0
f1 (ppm)

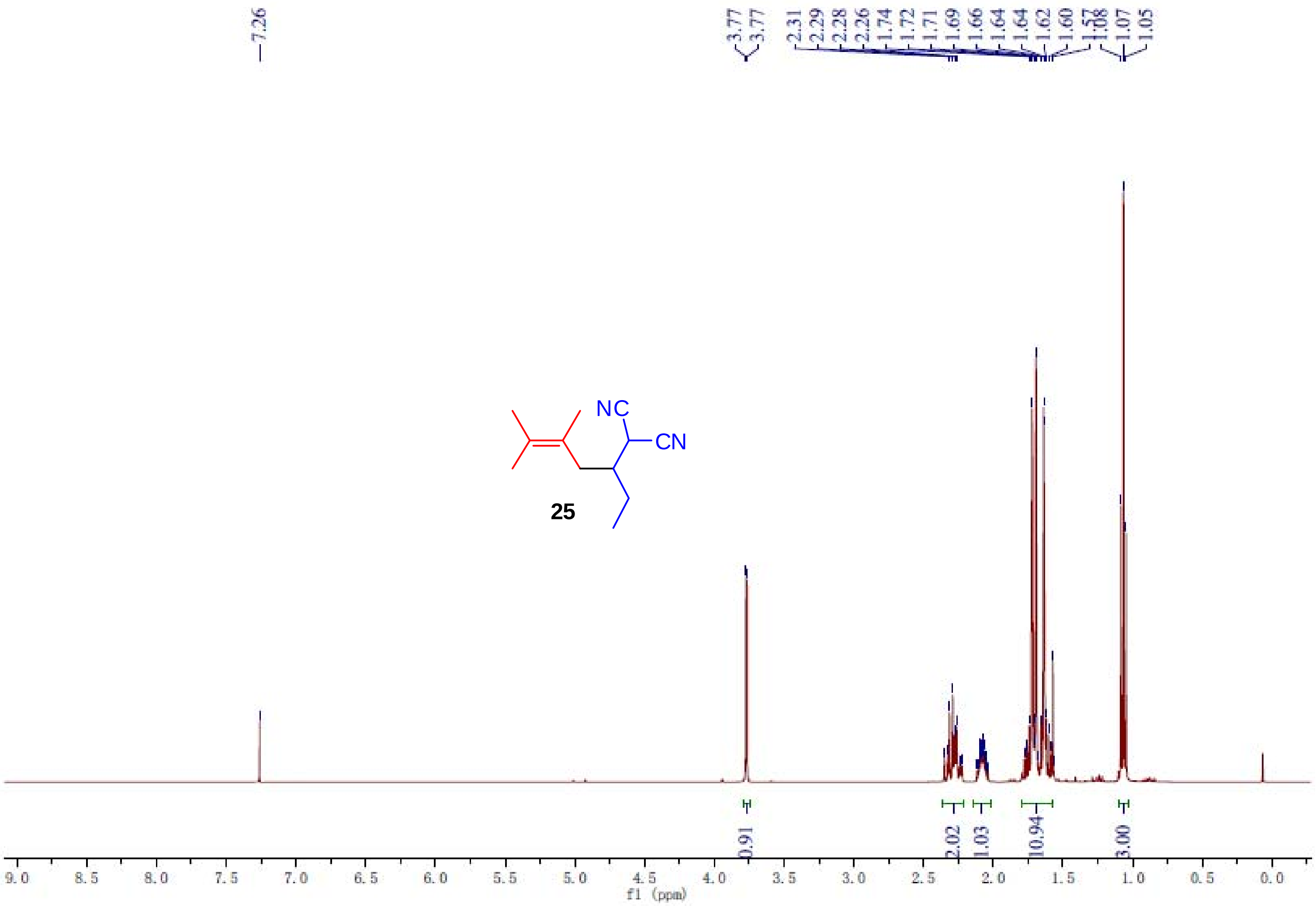

7.26
3.77
3.77
2.31
2.29
2.28
2.26
1.74
1.72
1.71
1.69
1.66
1.64
1.64
1.62
1.60
1.57
1.08
1.07
1.05
NC
CN
25
0.91
2.02
1.03
10.94
3.00
9.0
8.5
8.0
7.5
7.0
6.5
6.0
5.5
5.0
4.5
4.0
3.5
3.0
2.5
2.0
1.5
1.0
0.5
0.0
f1 (ppm)

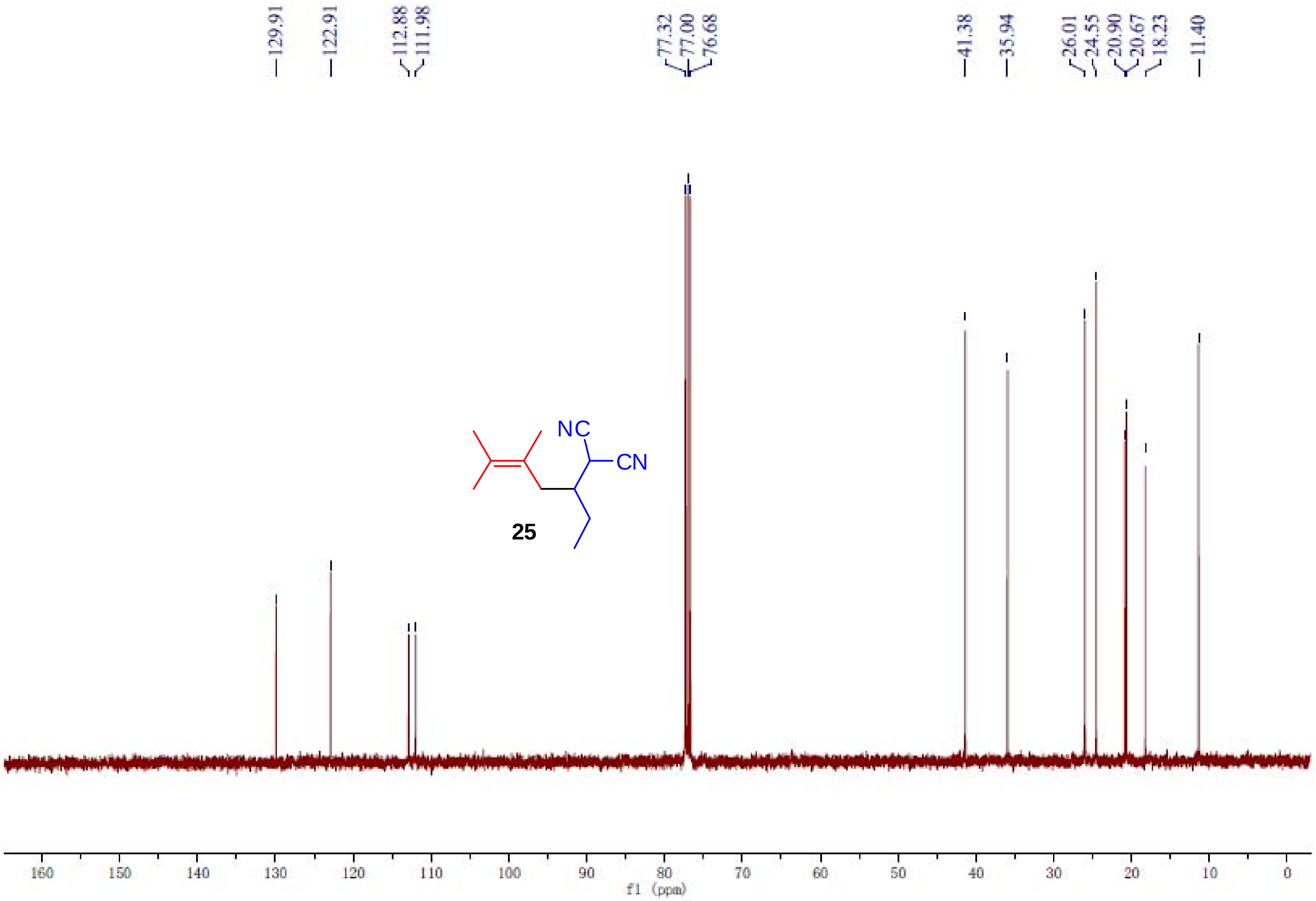

129.91
122.91
112.88
111.98
77.32
77.00
76.68
41.38
35.94
26.01
24.55
20.90
20.67
18.23
11.40
NC
CN
25
160 150 140 130 120 110 100 90 80 70 60 50 40 30 20 10 0
f1 (ppm)

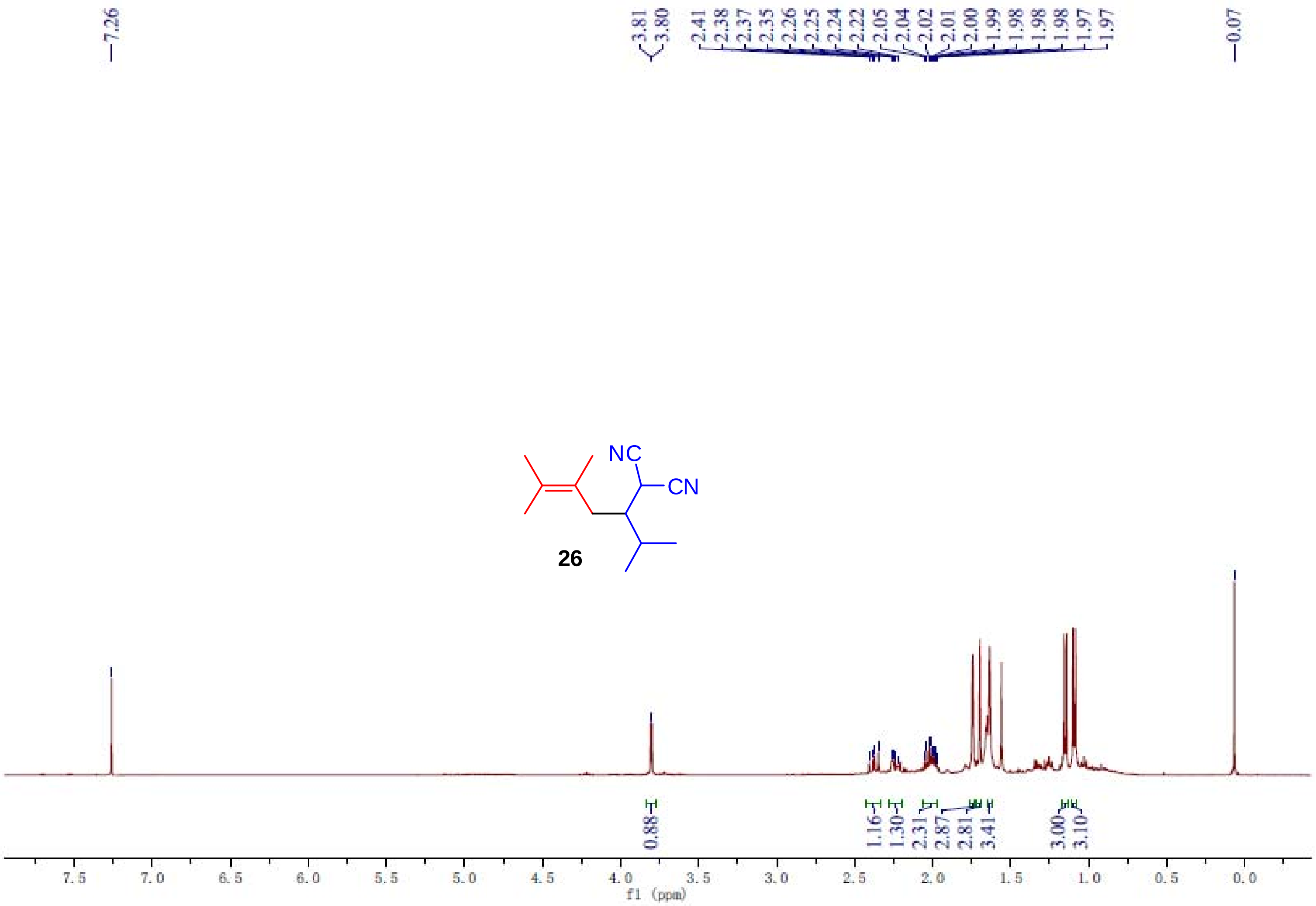
7.26
3.81
3.80
2.41
2.38
2.37
2.35
2.26
2.25
2.24
2.22
2.05
2.04
2.02
2.01
2.00
1.99
1.98
1.98
1.98
1.97
1.97
0.07
NC
CN
26
0.88
1.16
1.30
2.31
2.87
2.81
3.41
3.00
3.10
7.5
7.0
6.5
6.0
5.5
5.0
4.5
4.0
3.5
3.0
2.5
2.0
1.5
1.0
0.5
0.0
f1 (ppm)

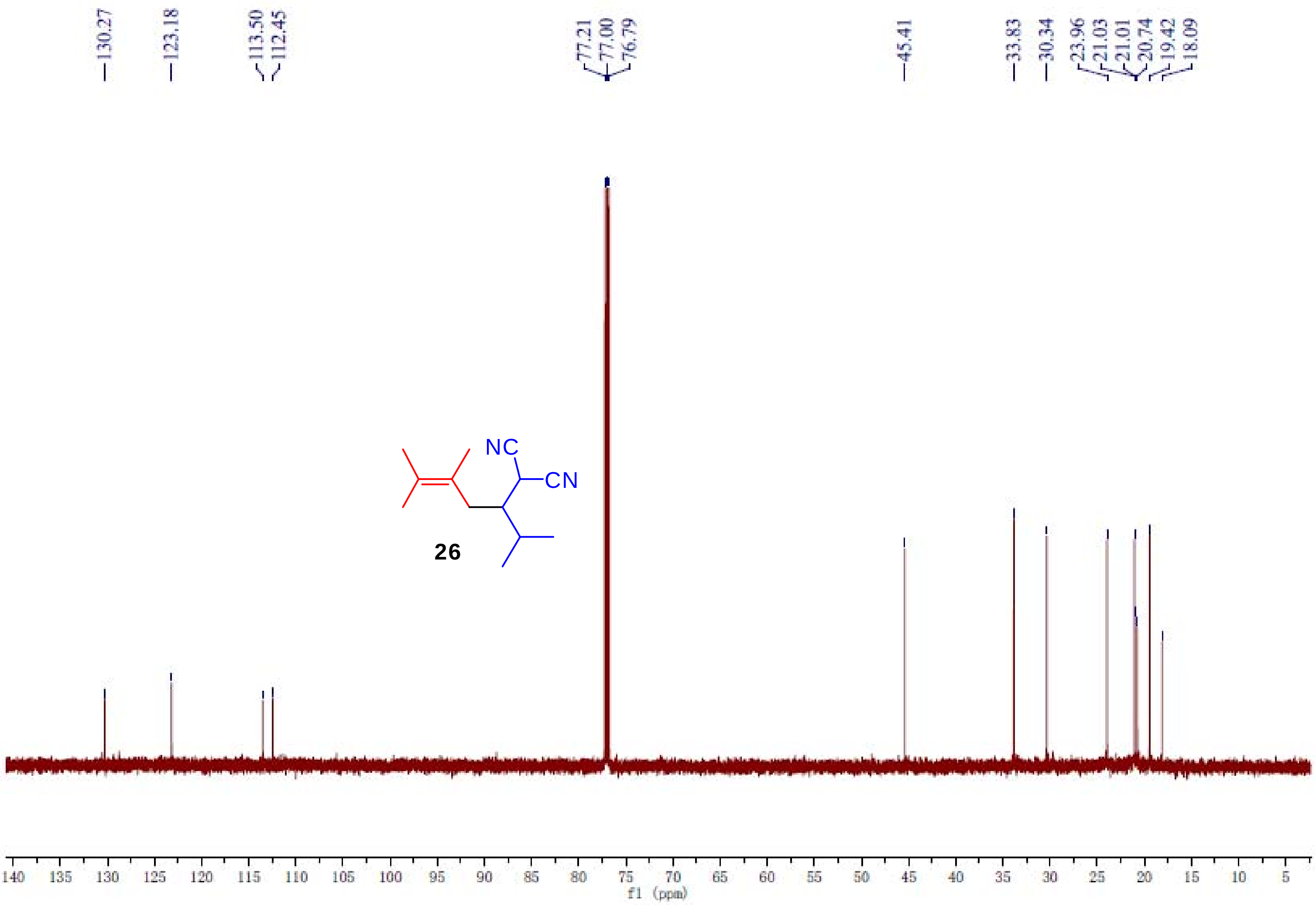

130.27
123.18
113.50
112.45
77.21
77.00
76.79
45.41
33.83
30.34
23.96
21.03
21.01
20.74
19.42
18.09
NC
CN
26
140 135 130 125 120 115 110 105 100 95 90 85 80 75 70 65 60 55 50 45 40 35 30 25 20 15 10 5
f1 (ppm)

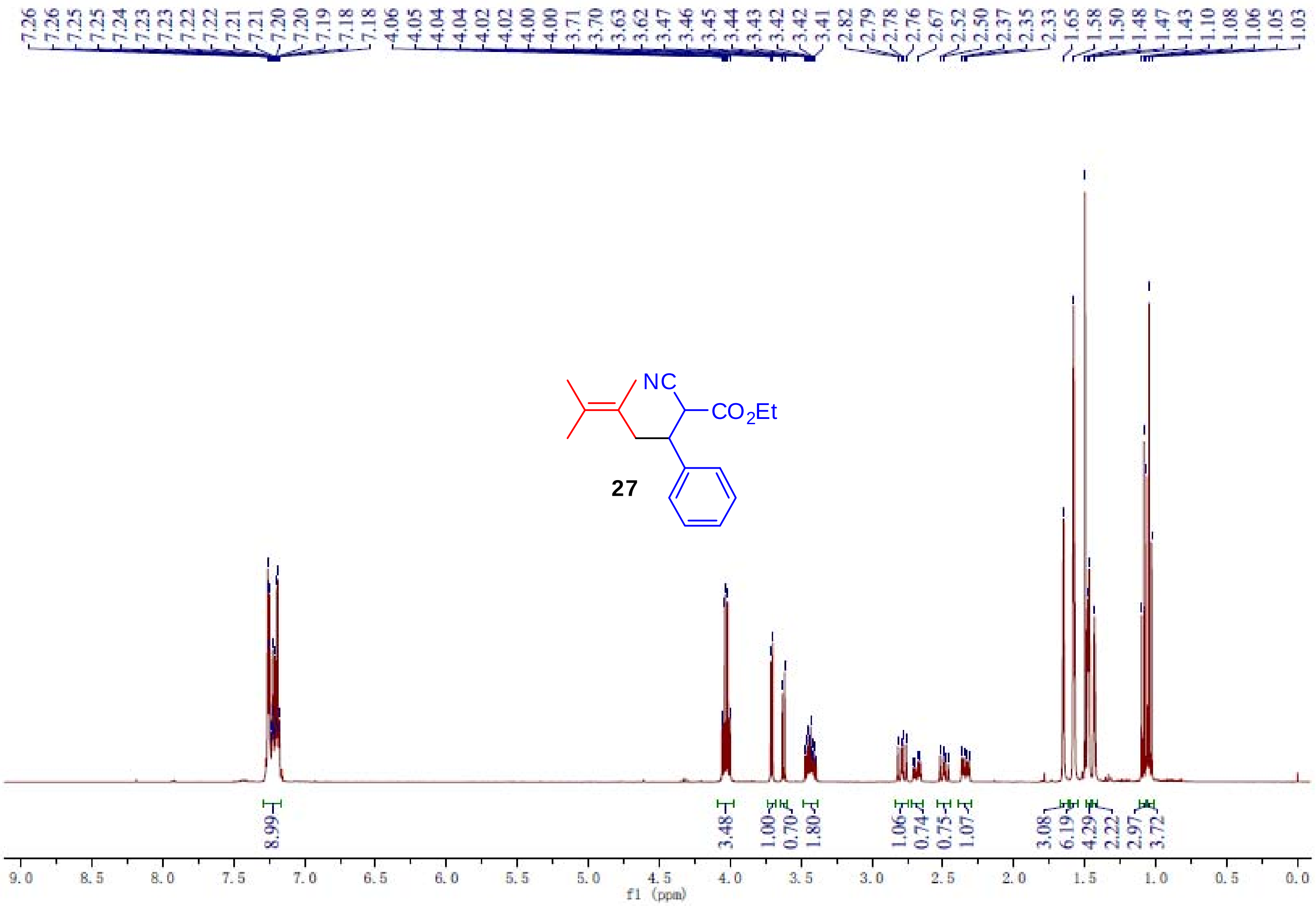
7.26
7.26
7.25
7.25
7.24
7.23
7.23
7.22
7.22
7.21
7.21
7.20
7.20
7.19
7.18
4.06
4.05
4.04
4.04
4.02
4.02
4.00
4.00
3.71
3.70
3.63
3.62
3.47
3.46
3.45
3.44
3.43
3.42
3.42
3.41
2.82
2.79
2.78
2.76
2.67
2.52
2.50
2.37
2.35
2.33
1.65
1.58
1.50
1.48
1.47
1.43
1.10
1.08
1.06
1.05
1.03
NC
CO_2Et
27
8.99
3.48
1.00
0.70
1.80
1.06
0.74
0.75
1.07
3.08
6.19
4.29
2.22
2.97
3.72
9.0
8.5
8.0
7.5
7.0
6.5
6.0
5.5
5.0
4.5
4.0
3.5
3.0
2.5
2.0
1.5
1.0
0.5
0.0
f1 (ppm)

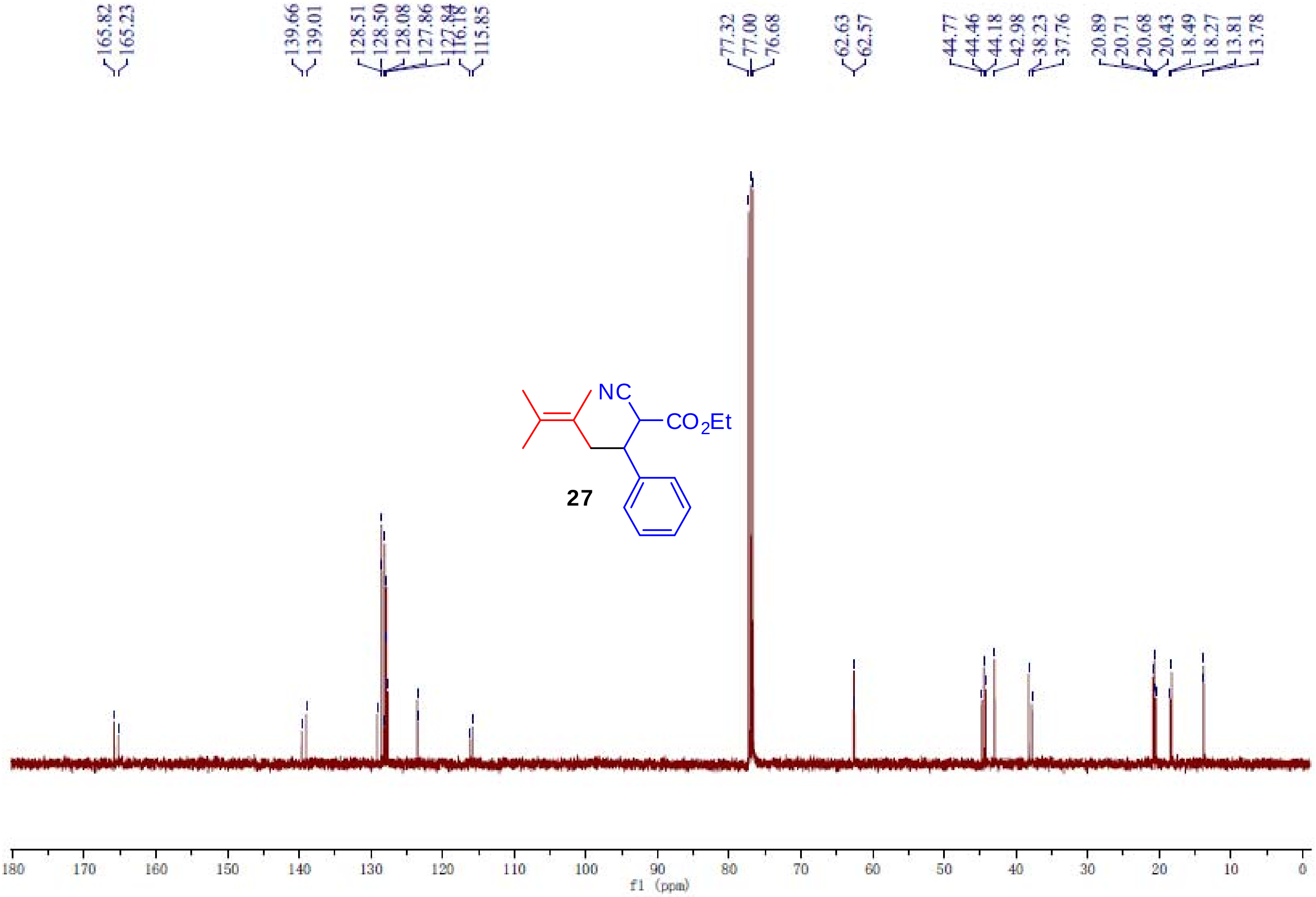
165.82
165.23
139.66
139.01
128.51
128.50
128.08
127.86
127.84
116.18
115.85
77.32
77.00
76.68
62.63
62.57
44.77
44.46
44.18
42.98
38.23
37.76
20.89
20.71
20.68
20.43
18.49
18.27
13.81
13.78
NC
CO2Et
27
180
170
160
150
140
130
120
110
100
90
80
70
60
50
40
30
20
10
0
f1 (ppm)

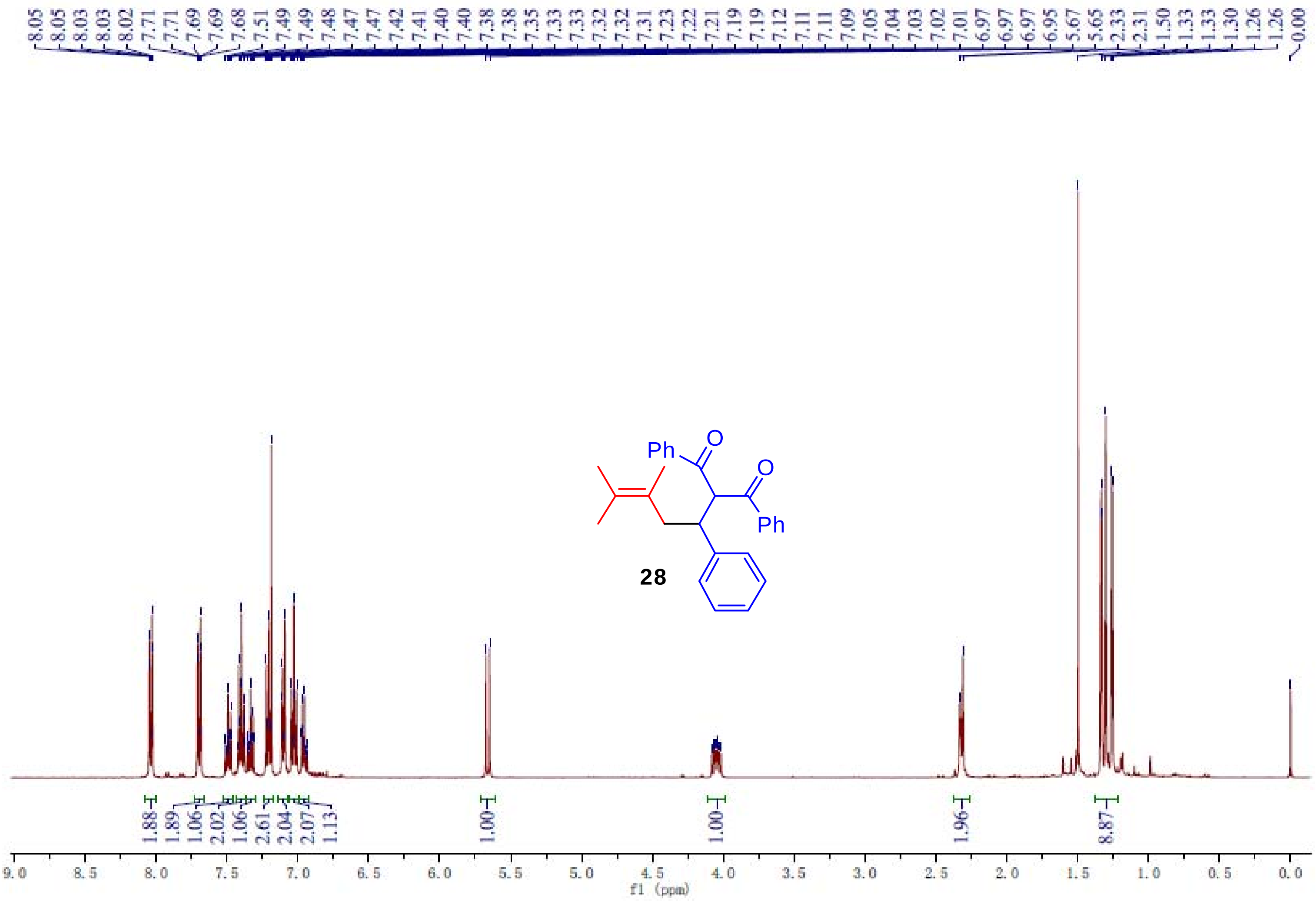
28

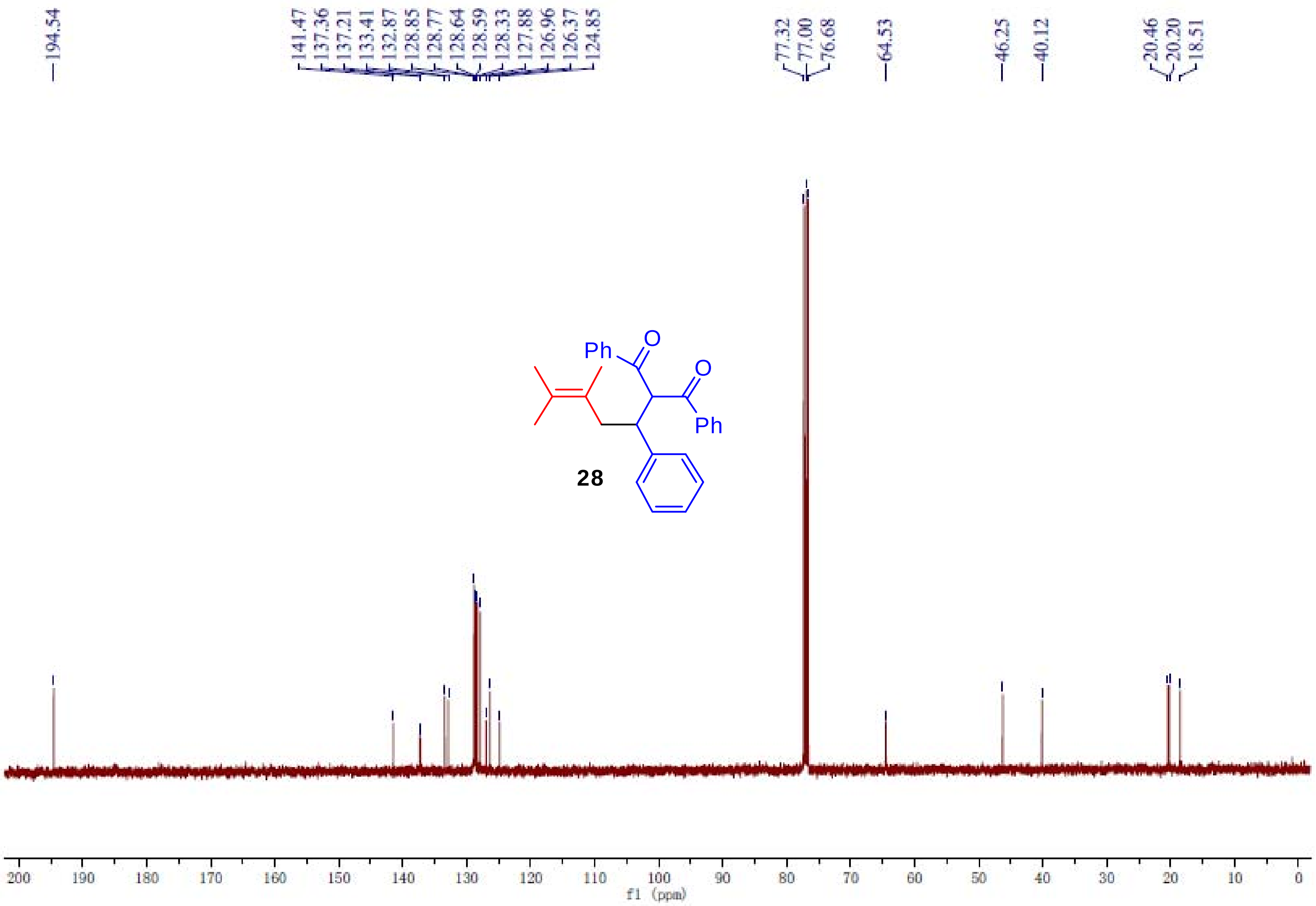
194.54
141.47
137.36
137.21
133.41
132.87
128.85
128.77
128.64
128.59
128.33
127.88
126.96
126.37
124.85
77.32
77.00
76.68
64.53
46.25
40.12
20.46
20.20
18.51
Ph
O
O
Ph
28
200 190 180 170 160 150 140 130 120 110 100 90 80 70 60 50 40 30 20 10 0
f1 (ppm)

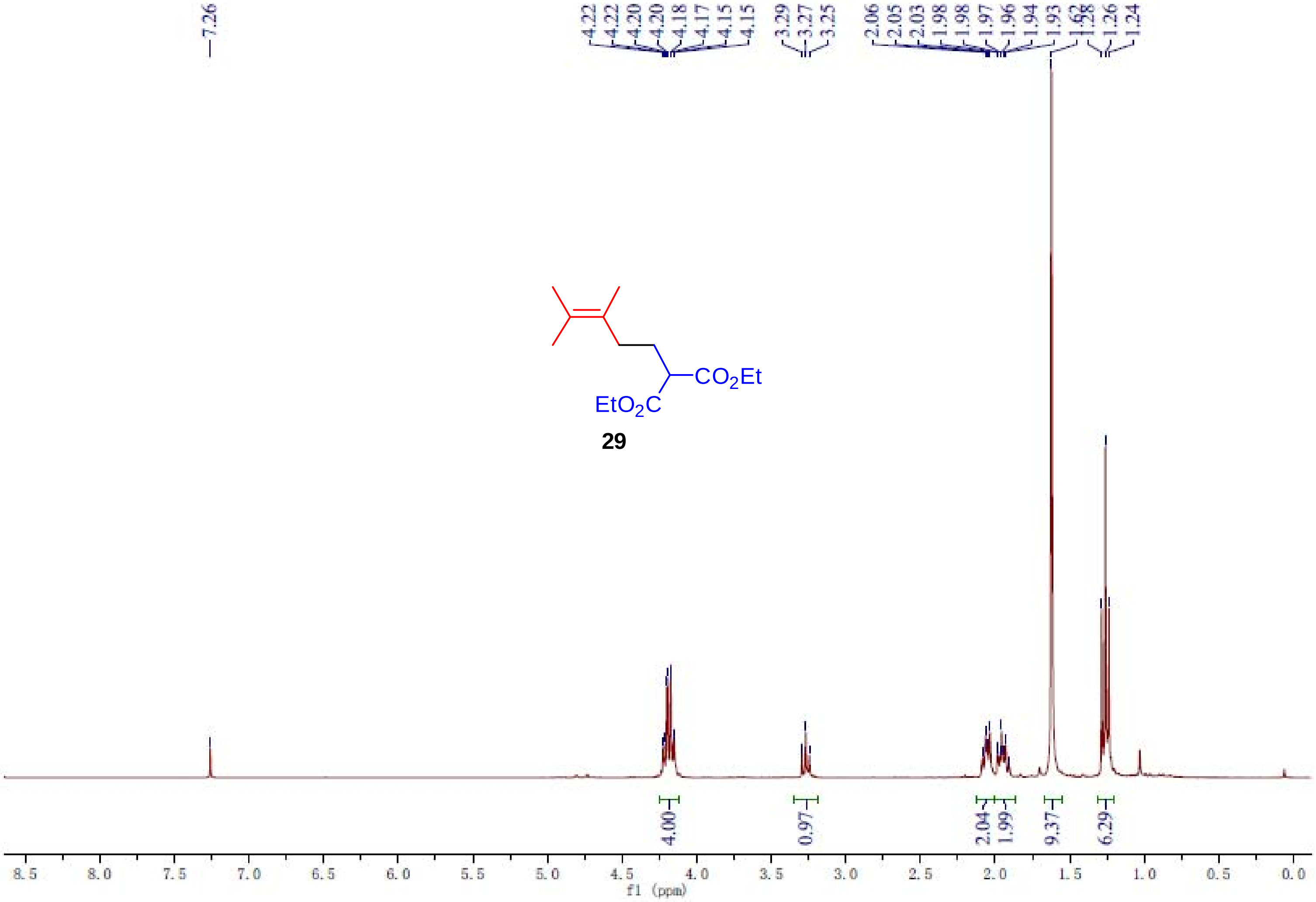
29
f1 (ppm)

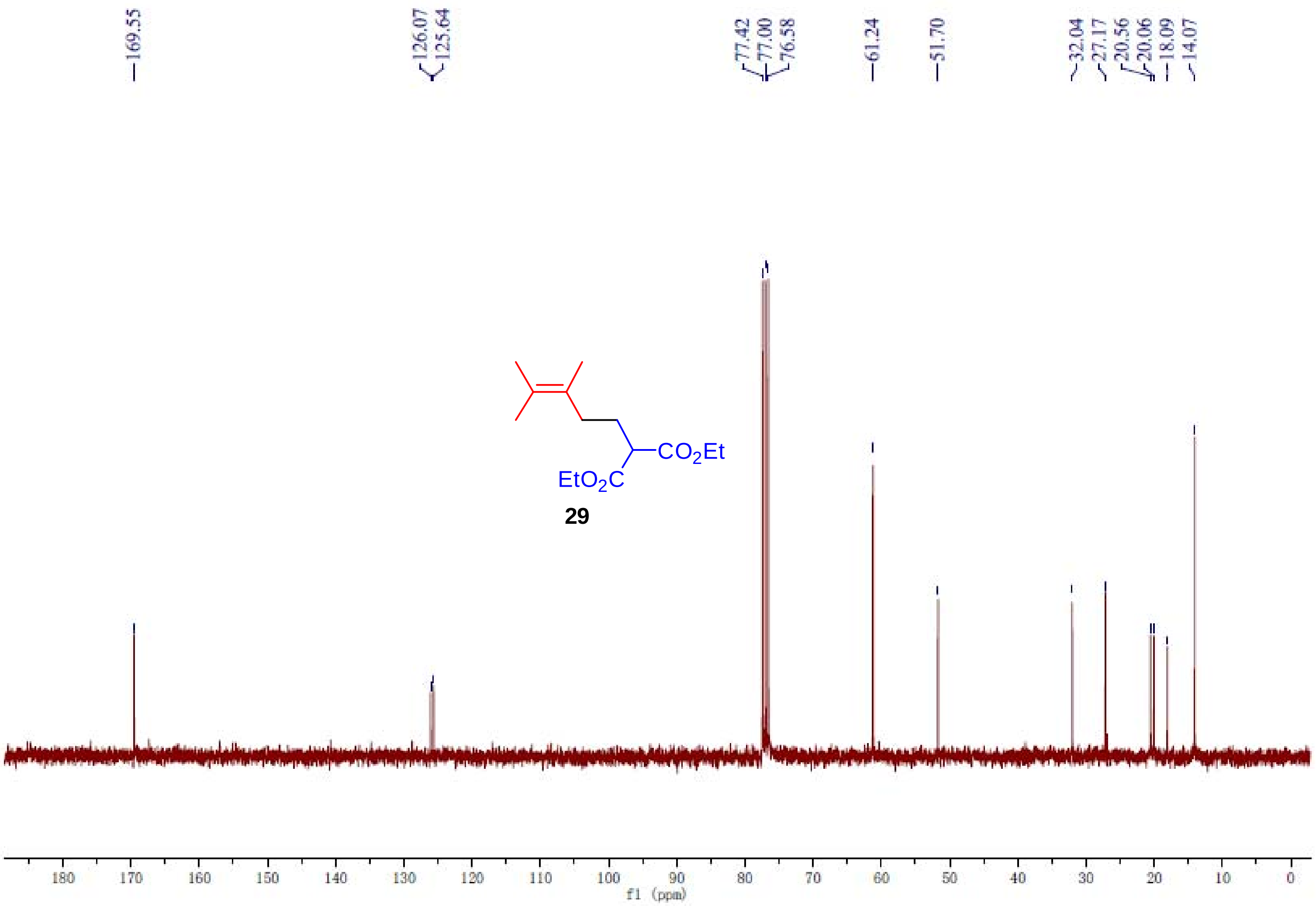
169.55
126.07
125.64
77.42
77.00
76.58
61.24
51.70
32.04
27.17
20.56
20.06
18.09
14.07
CO2Et
EtO2C
180
170
160
150
140
130
120
110
100
90
80
70
60
50
40
30
20
10
0
f1 (ppm)

29

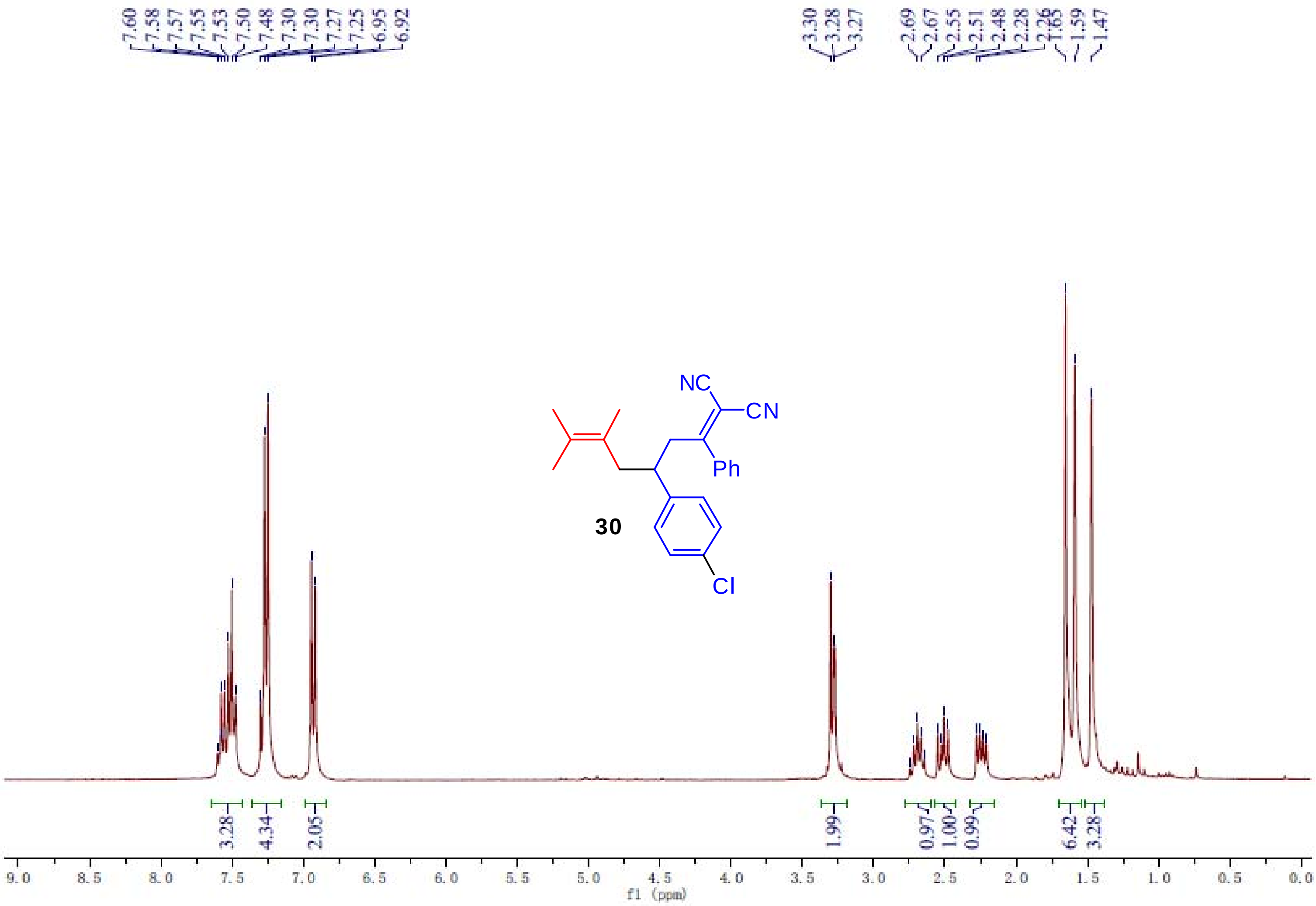
7.60
7.58
7.57
7.55
7.53
7.50
7.48
7.30
7.30
7.27
7.25
6.95
6.92
3.30
3.28
3.27
2.69
2.67
2.55
2.51
2.48
2.28
2.26
1.65
1.59
1.47
NC
CN
Ph
Cl
30
3.28
4.34
2.05
1.99
0.97
1.00
0.99
6.42
3.28
f1 (ppm)

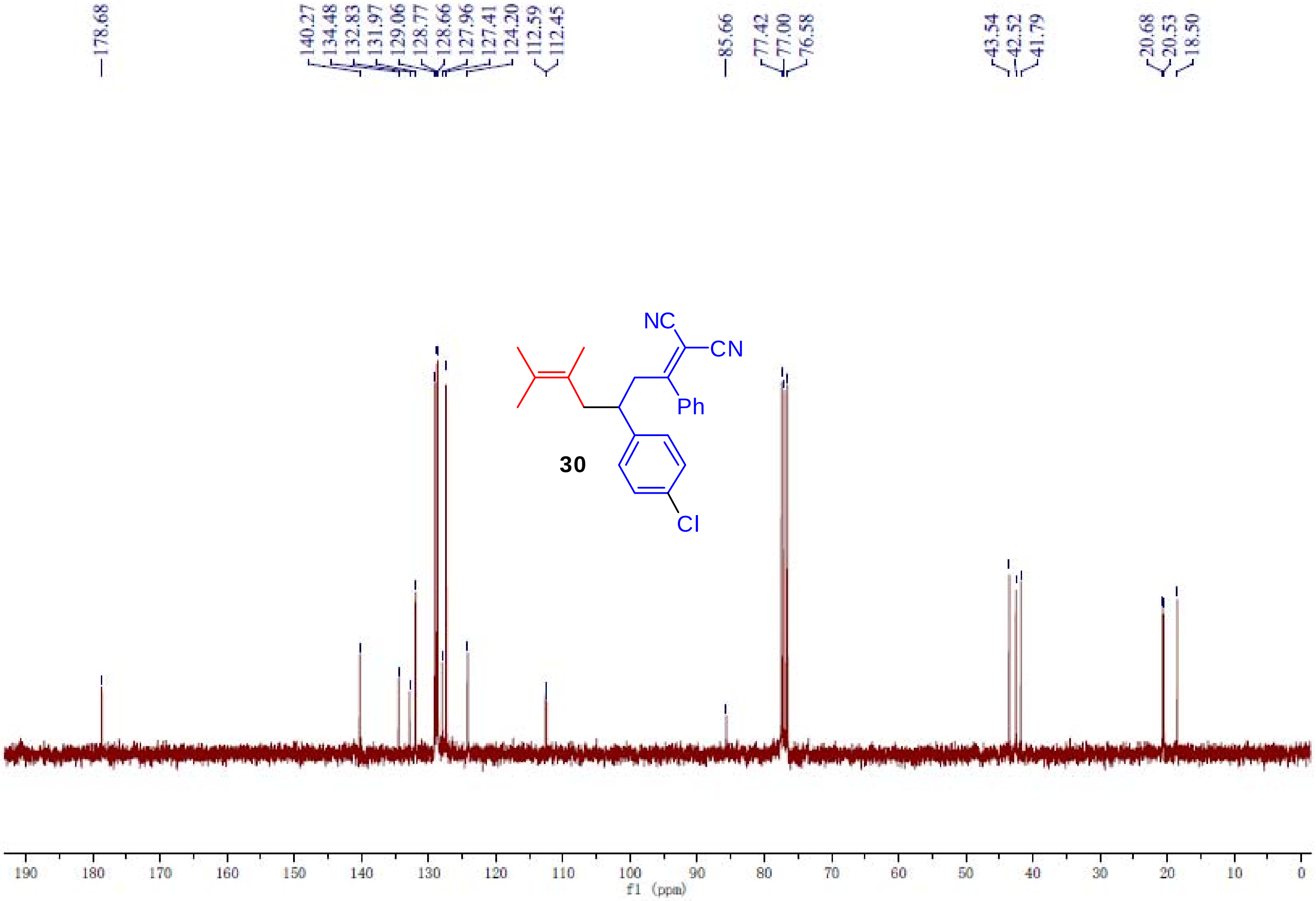

178.68
140.27
134.48
132.83
131.97
129.06
128.77
128.66
127.96
127.41
124.20
112.59
112.45
85.66
77.42
77.00
76.58
43.54
42.52
41.79
20.68
20.53
18.50
NC
CN
Ph
Cl
30
190 180 170 160 150 140 130 120 110 100 90 80 70 60 50 40 30 20 10 0
f1 (ppm)

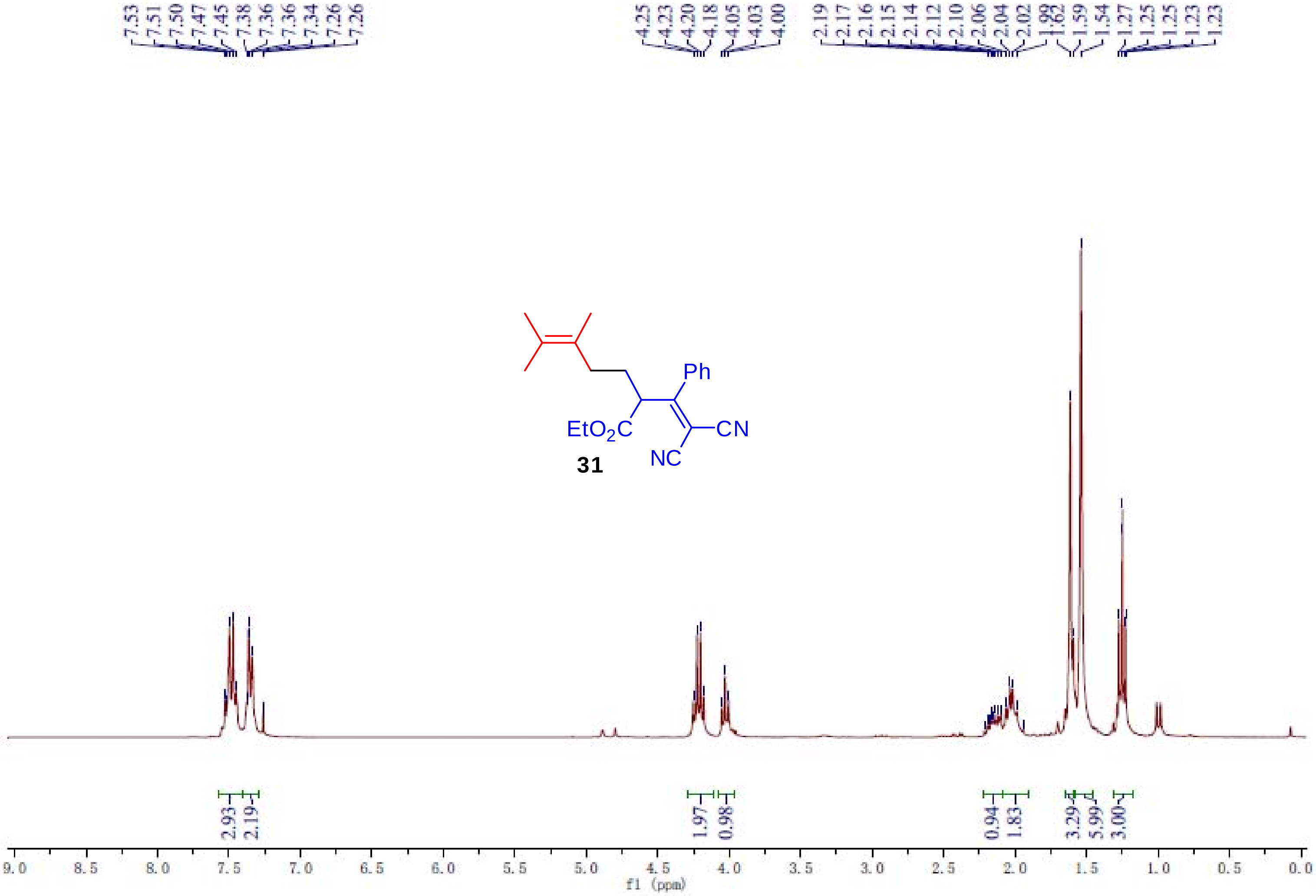

7.53
7.51
7.50
7.47
7.45
7.38
7.36
7.36
7.34
7.26
7.26
4.25
4.23
4.20
4.18
4.05
4.03
4.00
2.19
2.17
2.16
2.15
2.14
2.12
2.10
2.06
2.04
2.02
1.98
1.62
1.59
1.54
1.27
1.25
1.25
1.23
1.23
Ph
EtO2C
CN
NC
31
2.93
2.19
1.97
0.98
0.94
1.83
3.29
5.99
3.00
9.0 8.5 8.0 7.5 7.0 6.5 6.0 5.5 5.0 4.5 4.0 3.5 3.0 2.5 2.0 1.5 1.0 0.5 0.0
f1 (ppm)

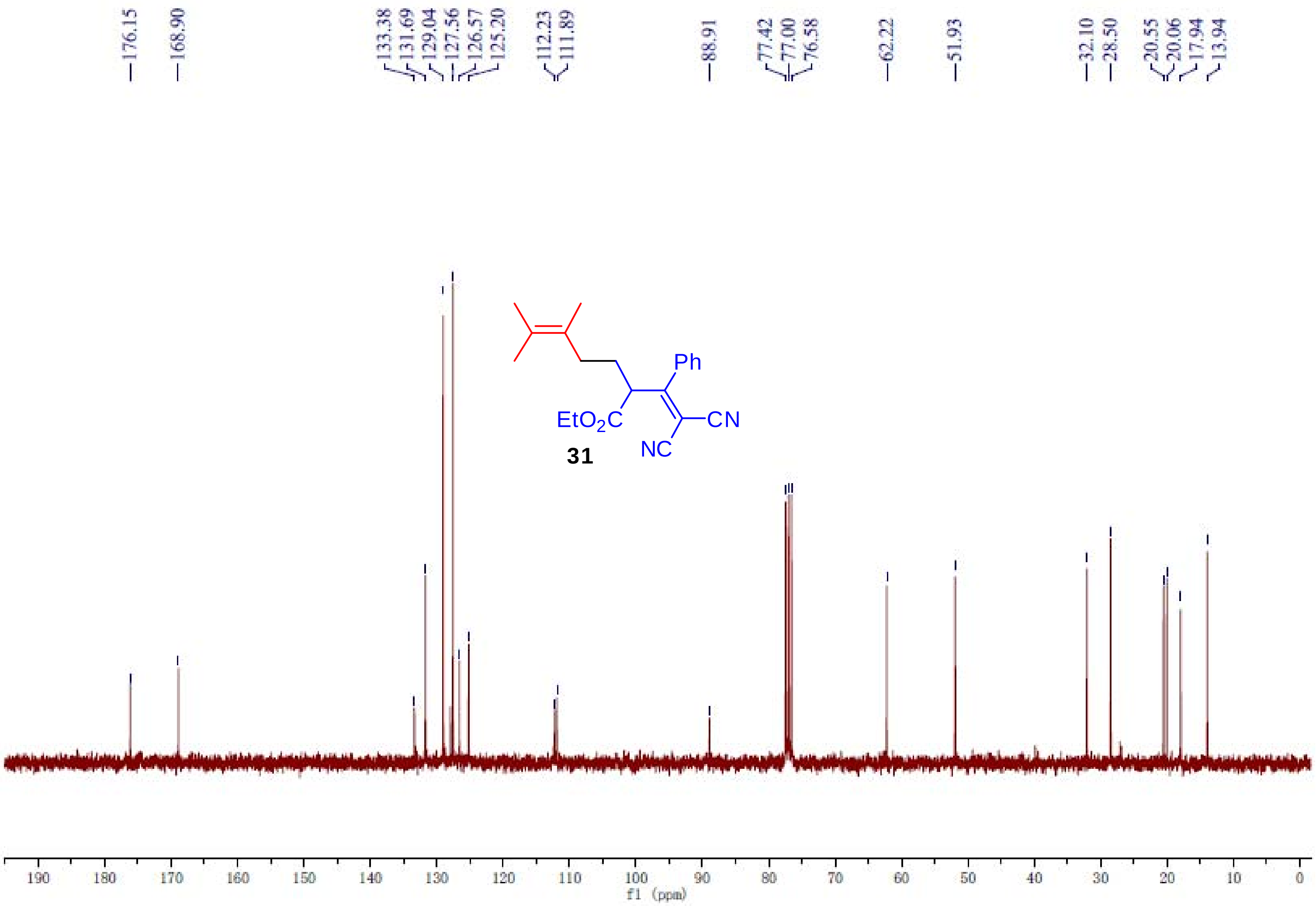
176.15
168.90
133.38
131.69
129.04
127.56
126.57
125.20
112.23
111.89
88.91
77.42
77.00
76.58
62.22
51.93
32.10
28.50
20.55
20.06
17.94
13.94
Ph
CN
NC
EtO2C
31
190 180 170 160 150 140 130 120 110 100 90 80 70 60 50 40 30 20 10 0
f1 (ppm)

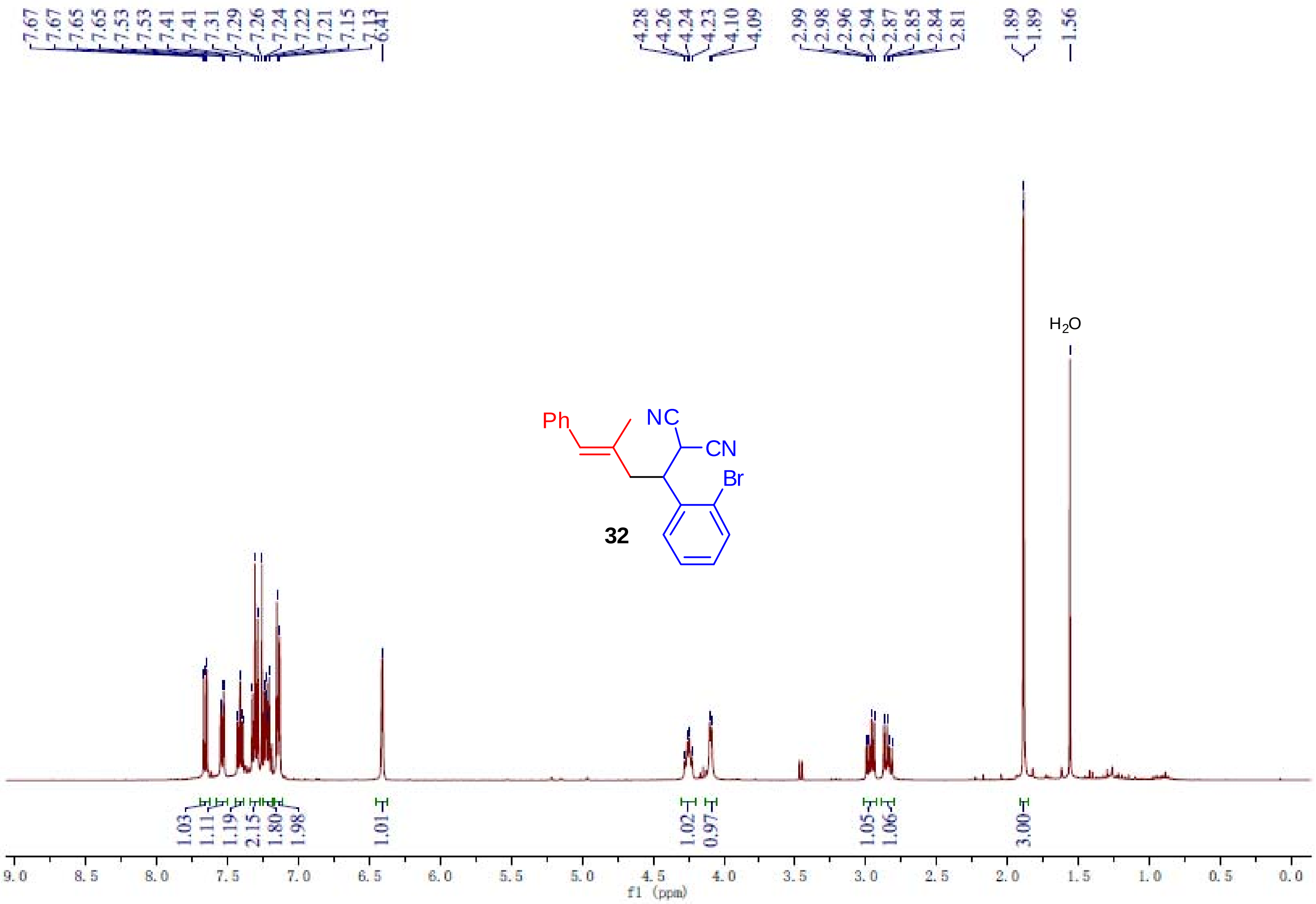
7.67
7.67
7.65
7.65
7.53
7.53
7.41
7.41
7.31
7.29
7.26
7.24
7.22
7.21
7.15
7.13
6.41
4.28
4.26
4.24
4.23
4.10
4.09
2.99
2.98
2.96
2.94
2.87
2.85
2.84
2.81
1.89
1.89
1.56
H2O
Ph
NC
CN
Br
32
1.03
1.11
1.19
2.15
1.80
1.98
1.01
1.02
0.97
1.05
1.06
3.00
9.0 8.5 8.0 7.5 7.0 6.5 6.0 5.5 5.0 4.5 4.0 3.5 3.0 2.5 2.0 1.5 1.0 0.5 0.0
f1 (ppm)

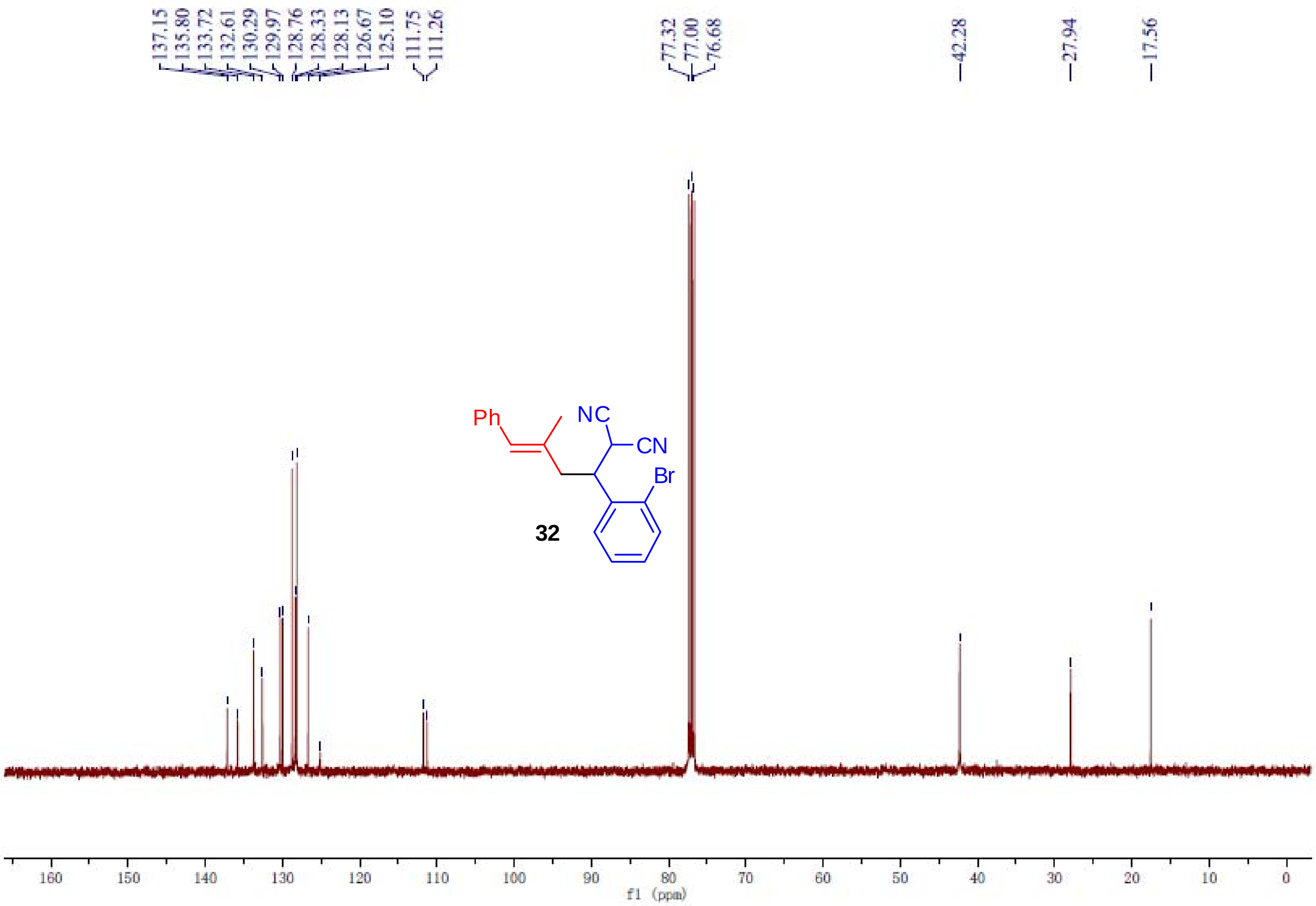
137.15
135.80
133.72
132.61
130.29
129.97
128.76
128.33
128.13
126.67
125.10
111.75
111.26
77.32
77.00
76.68
42.28
27.94
17.56
Ph
NC
CN
Br
32
160
150
140
130
120
110
100
90
80
70
60
50
40
30
20
10
0
f1 (ppm)

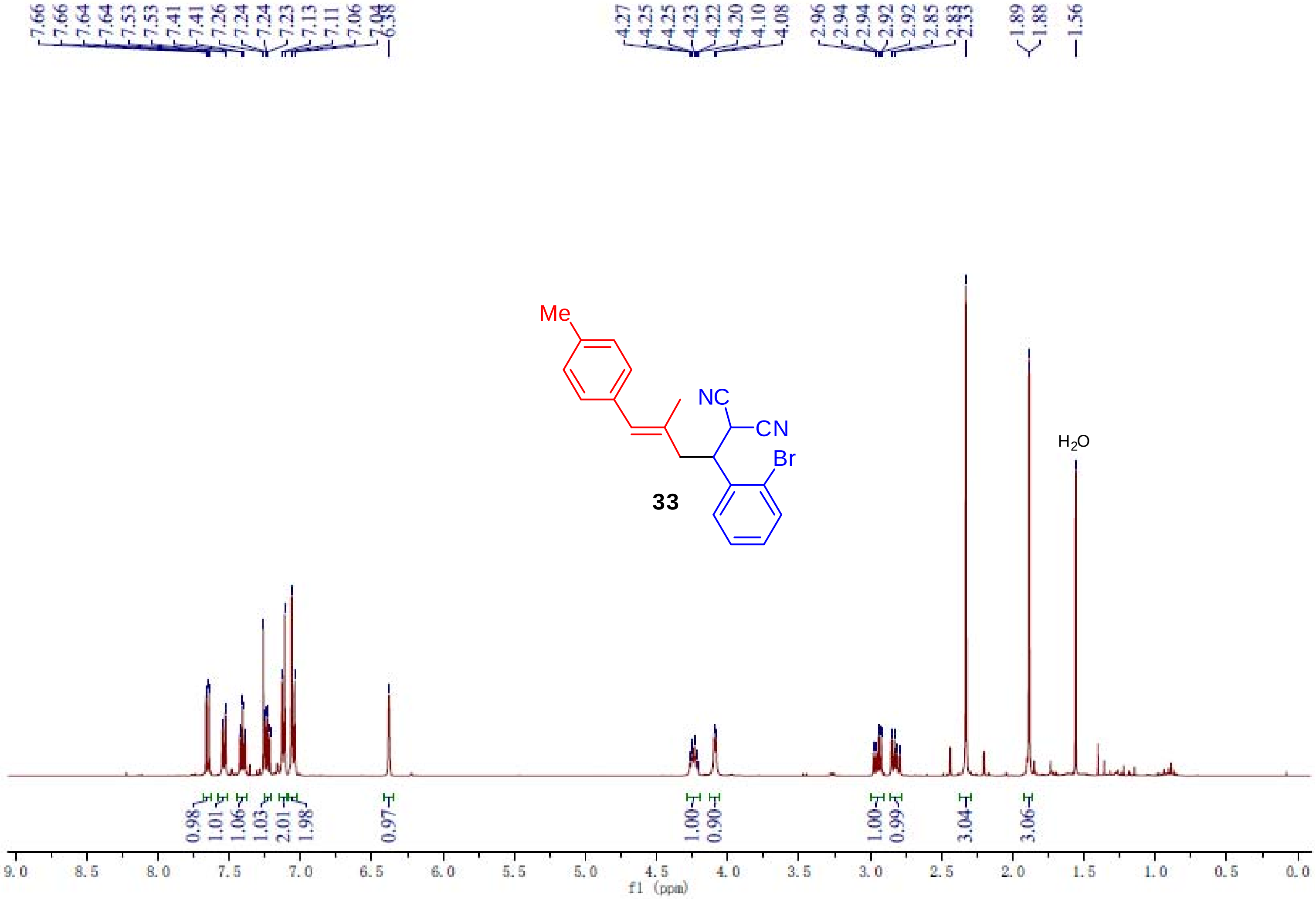
7.66
7.66
7.64
7.64
7.53
7.53
7.41
7.41
7.26
7.24
7.24
7.23
7.13
7.11
7.06
7.04
6.38
4.27
4.25
4.25
4.23
4.22
4.20
4.10
4.08
2.96
2.94
2.94
2.92
2.92
2.85
2.83
2.33
1.89
1.88
1.56
Me
NC
CN
Br
33
H2O
0.98
1.01
1.06
1.03
2.01
1.98
0.97
1.00
0.90
1.00
0.99
3.04
3.06
9.0 8.5 8.0 7.5 7.0 6.5 6.0 5.5 5.0 4.5 4.0 3.5 3.0 2.5 2.0 1.5 1.0 0.5 0.0
f1 (ppm)

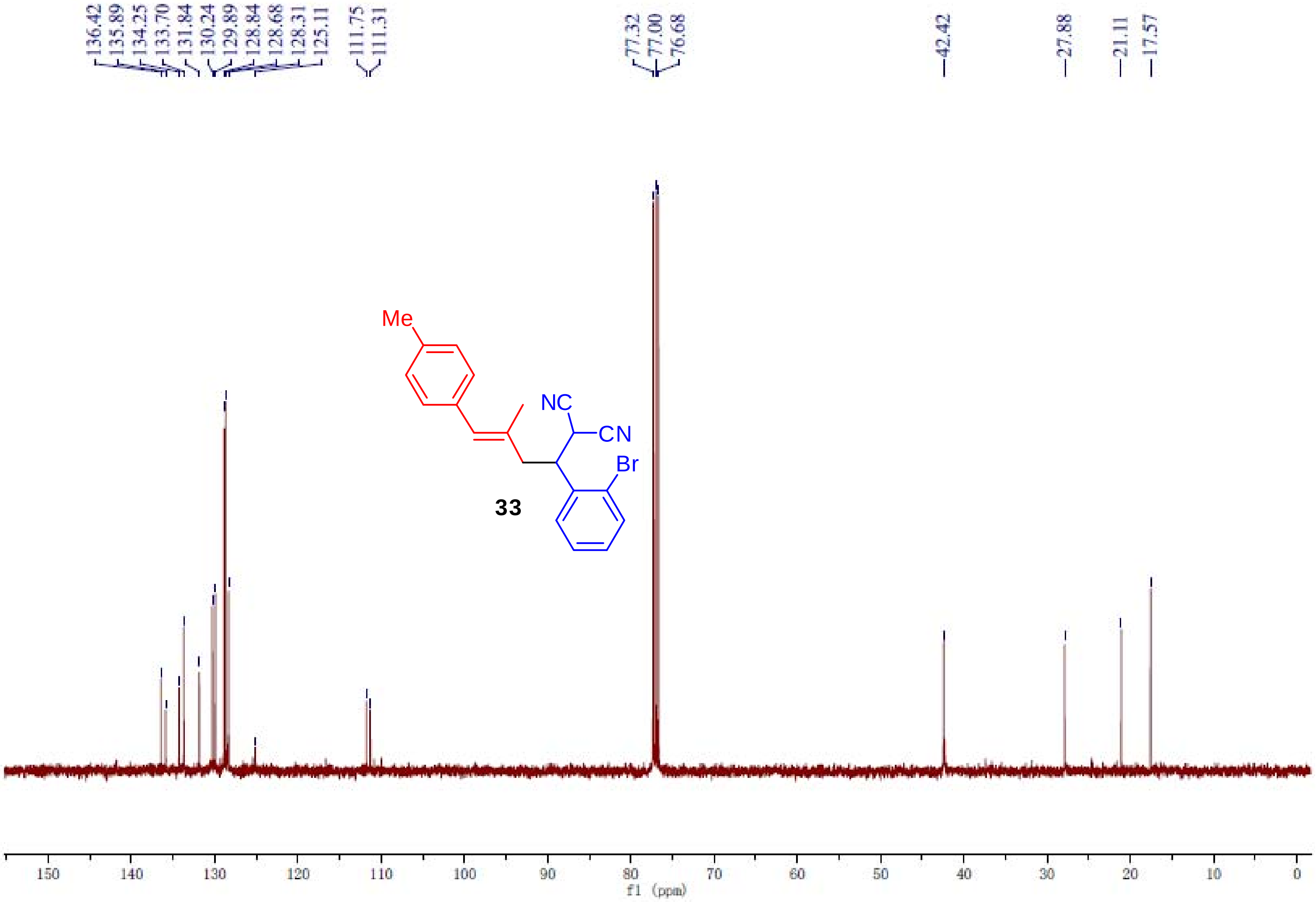

136.42
135.89
134.25
133.70
131.84
130.24
129.89
128.84
128.68
128.31
125.11
111.75
111.31
77.32
77.00
76.68
42.42
27.88
21.11
17.57
Me
NC
CN
Br
33
150 140 130 120 110 100 90 80 70 60 50 40 30 20 10 0
f1 (ppm)

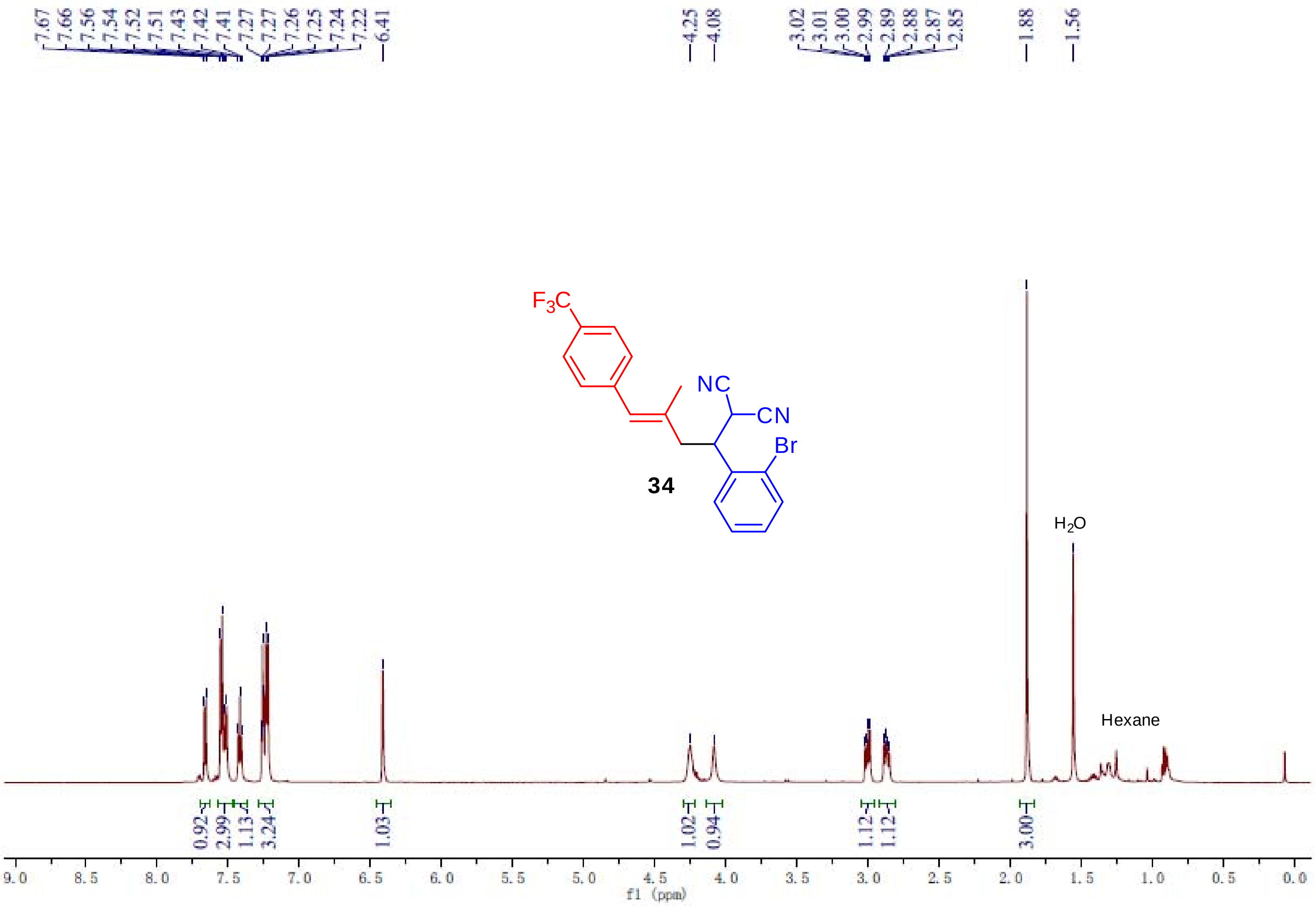
7.67
7.66
7.56
7.54
7.52
7.51
7.43
7.42
7.41
7.27
7.27
7.26
7.25
7.24
7.22
6.41
4.25
4.08
3.02
3.01
3.00
2.99
2.89
2.88
2.87
2.85
1.88
1.56
F3C
NC
CN
Br
34
H2O
Hexane
0.92
2.99
1.13
3.24
1.03
1.02
0.94
1.12
1.12
3.00
9.0
8.5
8.0
7.5
7.0
6.5
6.0
5.5
5.0
4.5
4.0
3.5
3.0
2.5
2.0
1.5
1.0
0.5
0.0
f1 (ppm)

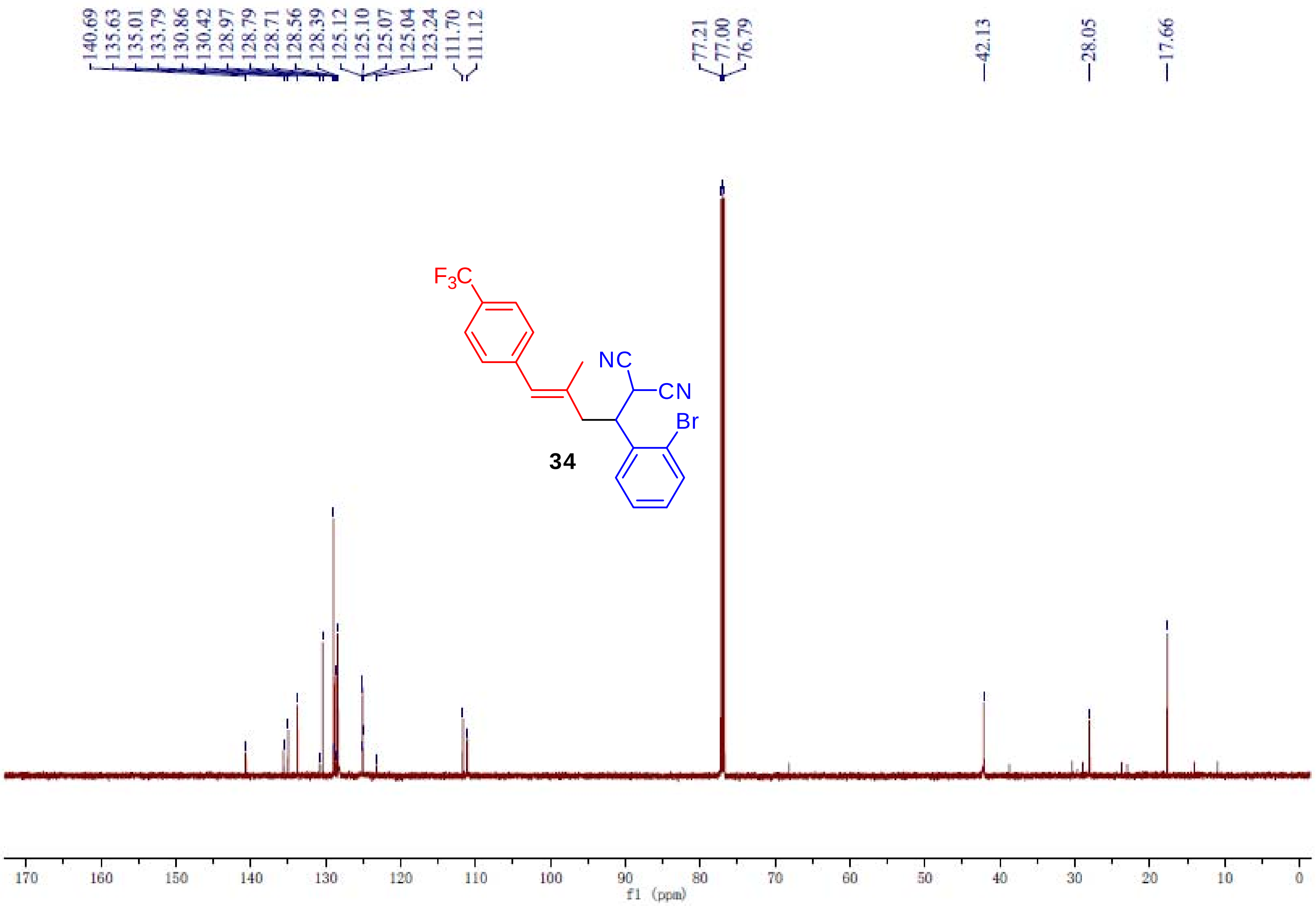
140.69
135.63
135.01
133.79
130.86
130.42
128.97
128.79
128.71
128.56
128.39
125.12
125.10
125.07
125.04
123.24
111.70
111.12
77.21
77.00
76.79
42.13
28.05
17.66
F_3C
NC
CN
Br
34
f1 (ppm)

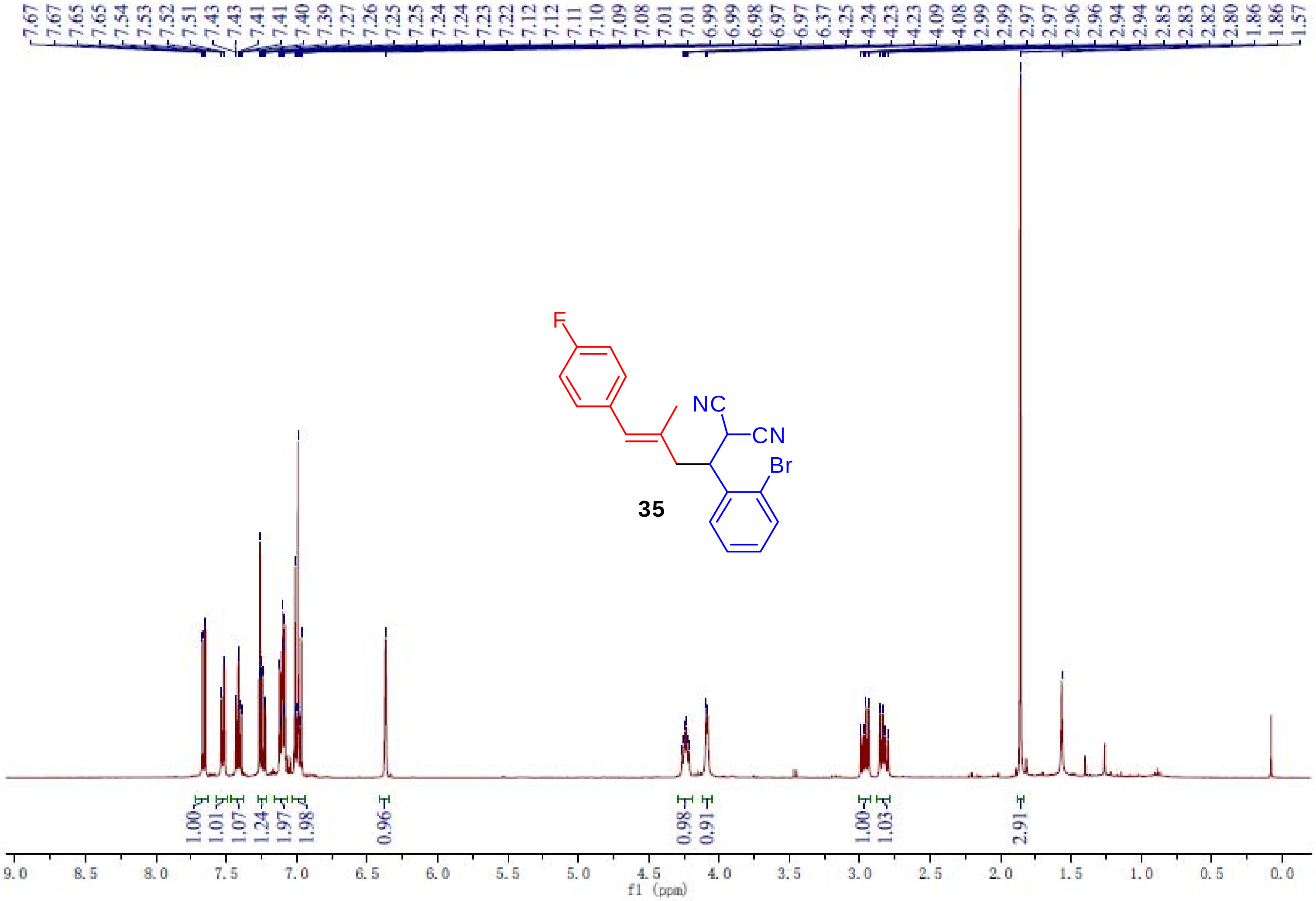

7.67
7.67
7.65
7.65
7.54
7.53
7.52
7.51
7.43
7.43
7.41
7.41
7.40
7.39
7.27
7.26
7.25
7.25
7.24
7.24
7.23
7.22
7.12
7.12
7.11
7.10
7.09
7.08
7.01
7.01
6.99
6.99
6.98
6.97
6.97
6.37
4.25
4.24
4.23
4.23
4.09
4.08
2.99
2.99
2.97
2.97
2.96
2.96
2.94
2.94
2.85
2.83
2.82
2.80
1.86
1.86
1.57
F
NC
CN
Br
1.00
1.01
1.07
1.24
1.97
1.98
0.96
0.98
0.91
1.00
1.03
2.91
9.0
8.5
8.0
7.5
7.0
6.5
6.0
5.5
5.0
4.5
4.0
3.5
3.0
2.5
2.0
1.5
1.0
0.5
0.0
f1 (ppm)

35

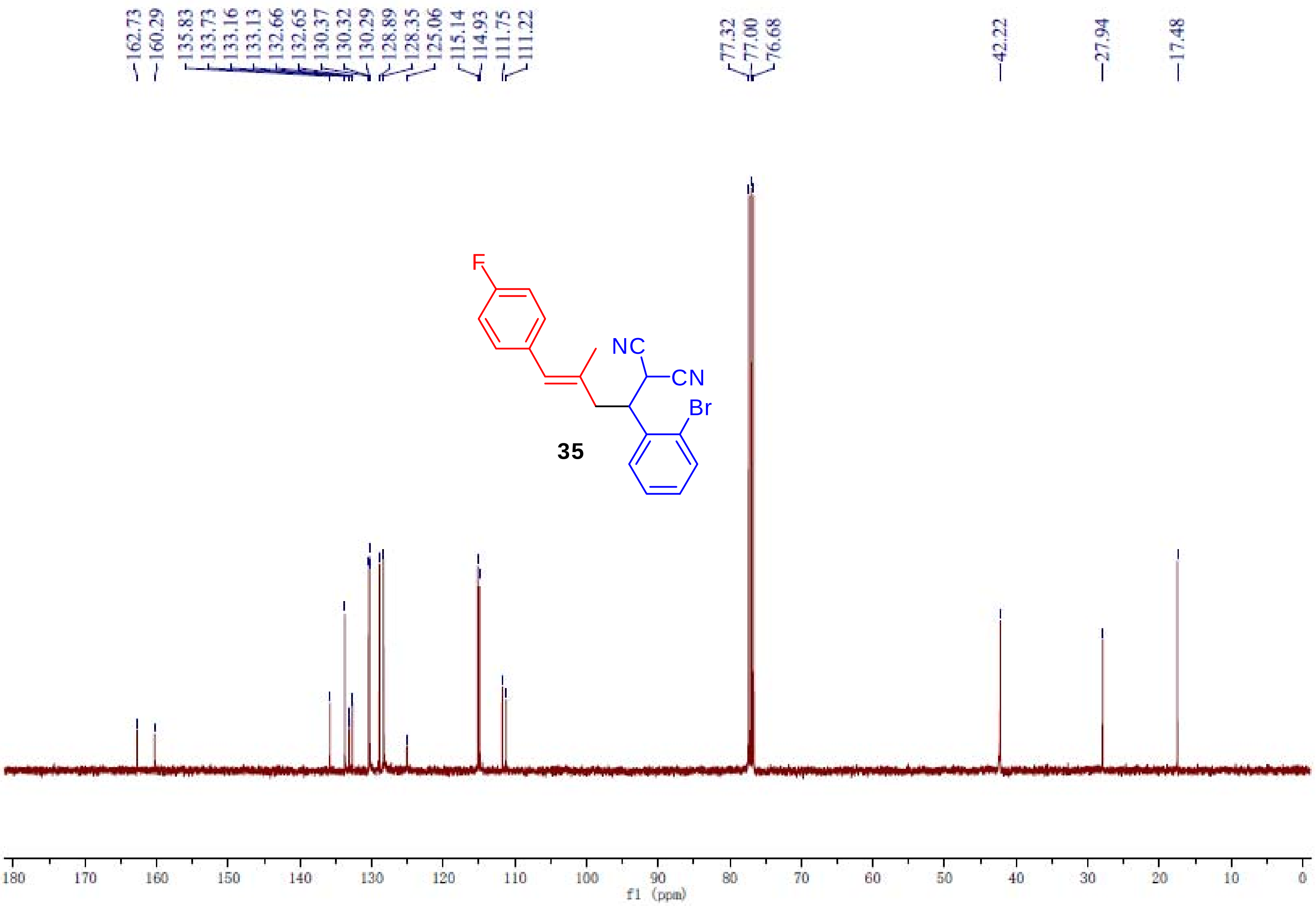

162.73
160.29
135.83
133.73
133.16
133.13
132.66
132.65
130.37
130.32
130.29
128.89
128.35
125.06
115.14
114.93
111.75
111.22
77.32
77.00
76.68
42.22
27.94
17.48
F
NC
CN
Br
35
180
170
160
150
140
130
120
110
100
90
80
70
60
50
40
30
20
10
0
f1 (ppm)

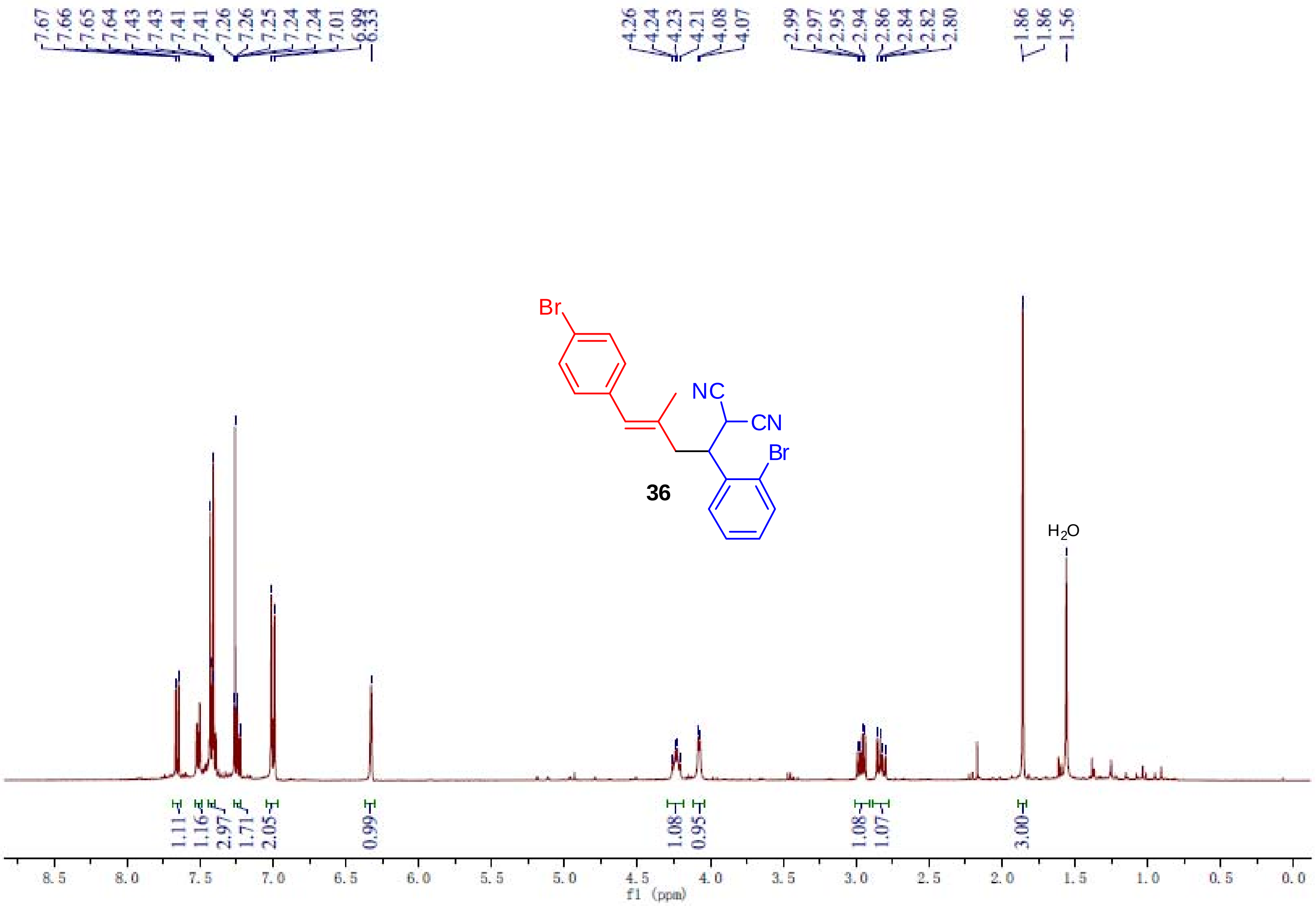
7.67
7.66
7.65
7.64
7.43
7.43
7.41
7.41
7.26
7.26
7.25
7.24
7.24
7.01
6.99
6.33
4.26
4.24
4.23
4.21
4.08
4.07
2.99
2.97
2.95
2.94
2.86
2.84
2.82
2.80
1.86
1.86
1.56
Br
NC
CN
Br
36
H2O
1.11
1.16
2.97
1.71
2.05
0.99
1.08
0.95
1.08
1.07
3.00
8.5
8.0
7.5
7.0
6.5
6.0
5.5
5.0
4.5
4.0
3.5
3.0
2.5
2.0
1.5
1.0
0.5
0.0
f1 (ppm)

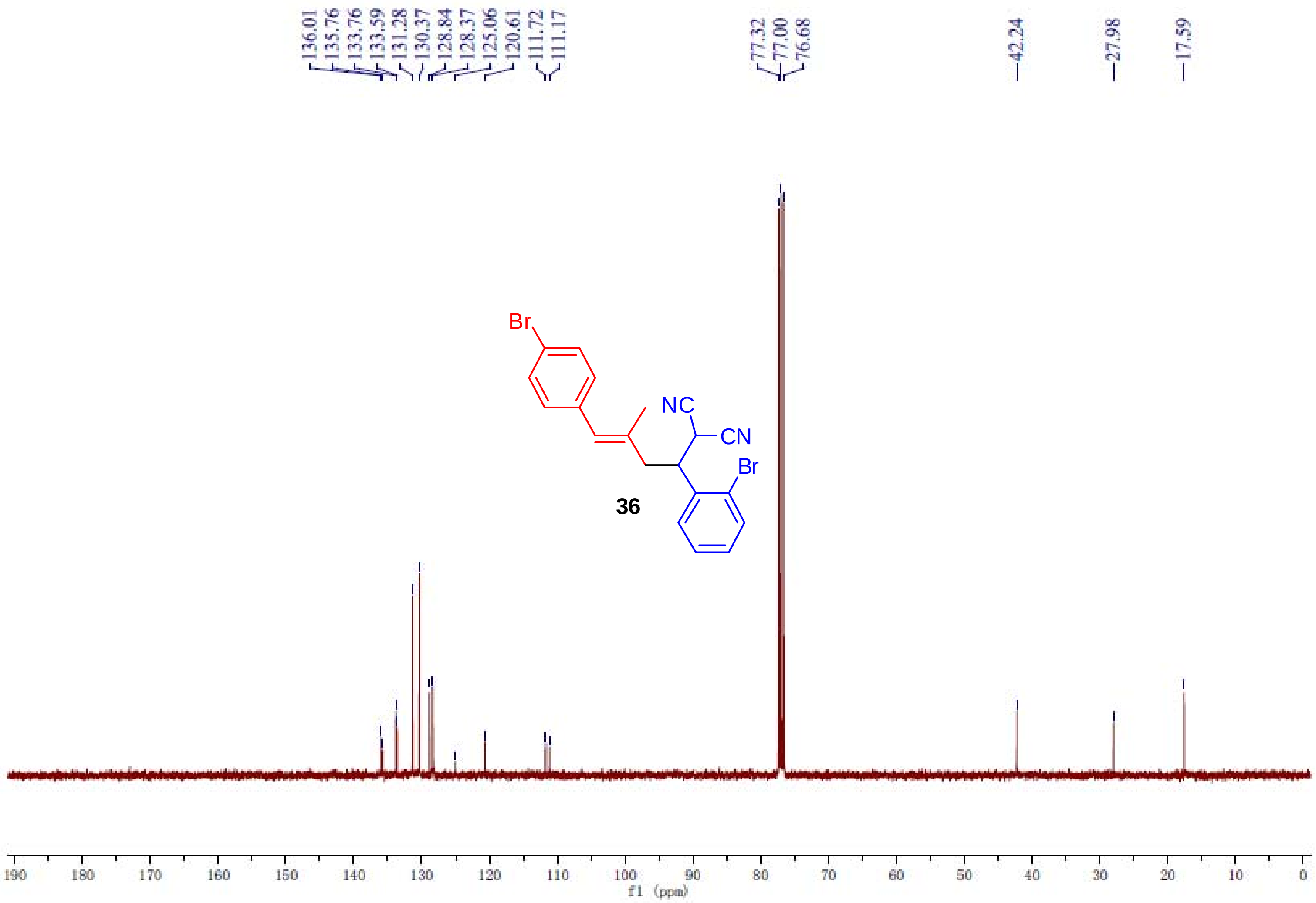
136.01
135.76
133.76
133.59
131.28
130.37
128.84
128.37
125.06
120.61
111.72
111.17
77.32
77.00
76.68
42.24
27.98
17.59
Br
NC
CN
Br
36
190 180 170 160 150 140 130 120 110 100 90 80 70 60 50 40 30 20 10 0
f1 (ppm)

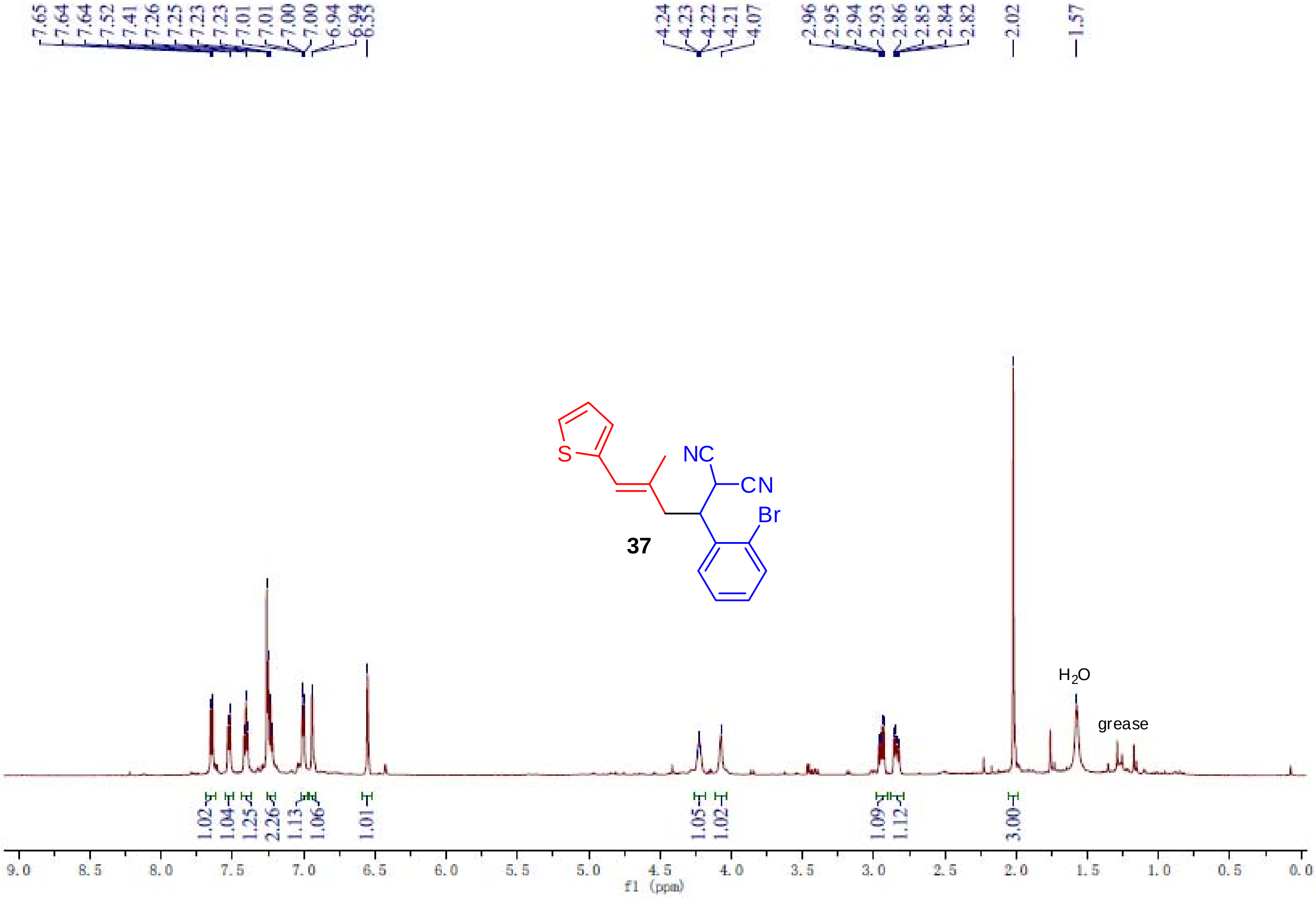
7.65
7.64
7.64
7.52
7.41
7.26
7.25
7.23
7.23
7.01
7.01
7.00
7.00
6.94
6.94
6.55
4.24
4.23
4.22
4.21
4.07
2.96
2.95
2.94
2.93
2.86
2.85
2.84
2.82
2.02
1.57
NC
CN
Br
S
37
H2O
grease
1.02
1.04
1.25
2.26
1.13
1.06
1.01
1.05
1.02
1.09
1.12
3.00
9.0
8.5
8.0
7.5
7.0
6.5
6.0
5.5
5.0
4.5
4.0
3.5
3.0
2.5
2.0
1.5
1.0
0.5
0.0
f1 (ppm)

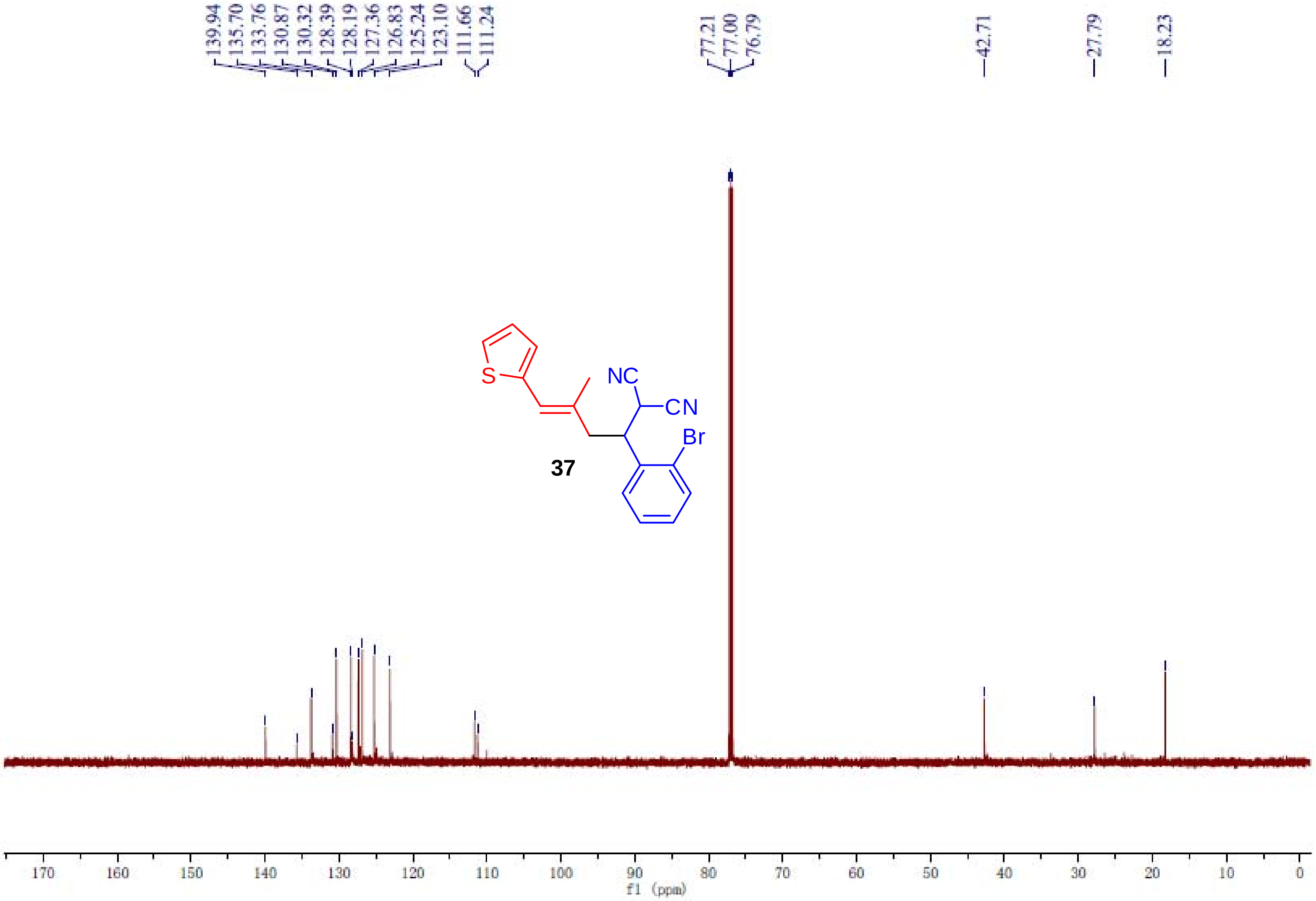
139.94
135.70
133.76
130.87
130.32
128.39
128.19
127.36
126.83
125.24
123.10
111.66
111.24
77.21
77.00
76.79
42.71
27.79
18.23
S
NC
CN
Br
37
170 160 150 140 130 120 110 100 90 80 70 60 50 40 30 20 10 0
f1 (ppm)

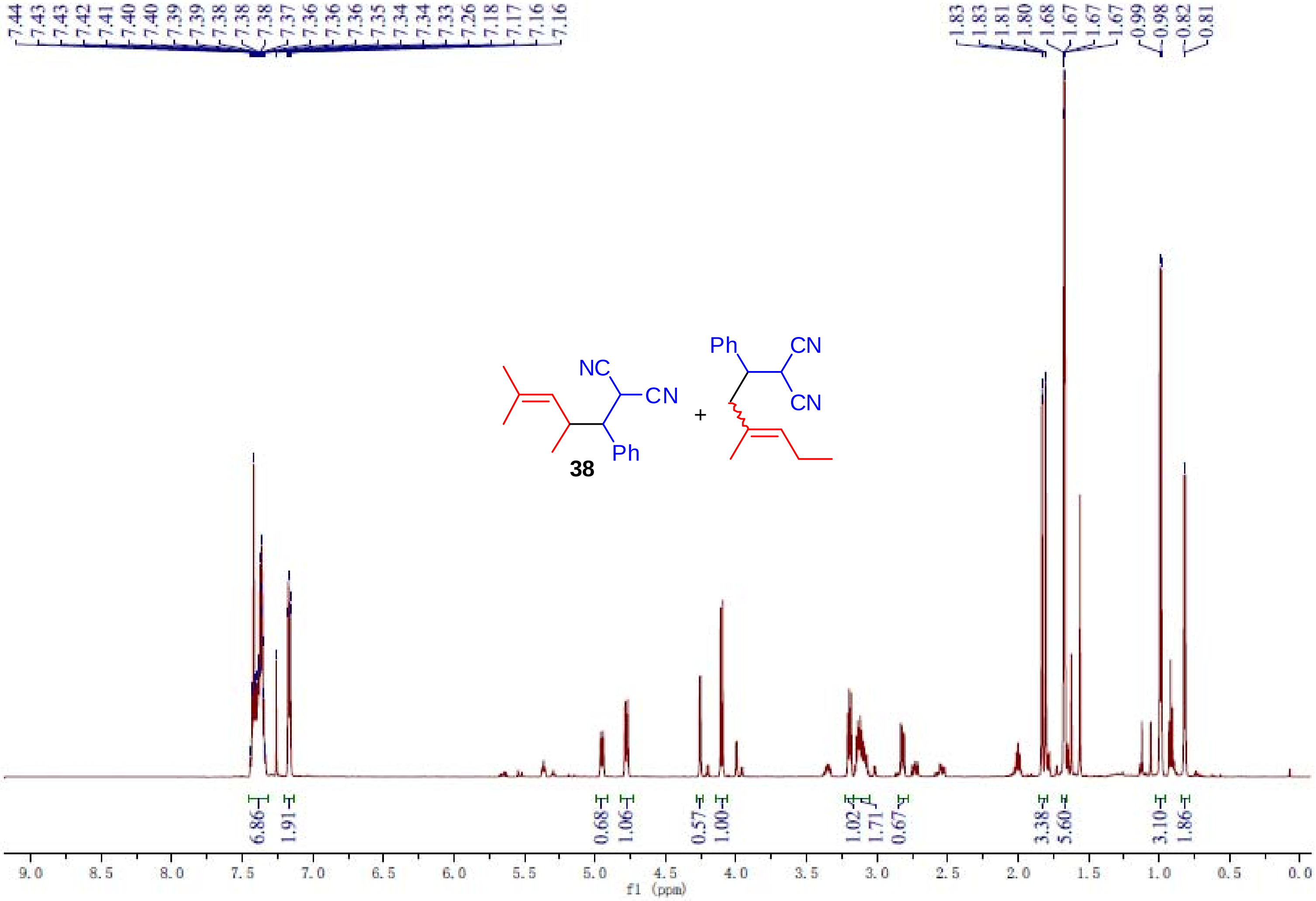
7.44 7.43 7.43 7.42 7.41 7.40 7.40 7.39 7.39 7.38 7.38 7.38 7.37 7.36 7.36 7.36 7.35 7.34 7.34 7.33 7.26 7.18 7.17 7.16 7.16
1.83 1.83 1.81 1.80 1.68 1.67 1.67 1.67 0.99 0.98 0.82 0.81
NC
CN
Ph
38
+
Ph
CN
CN
6.86 1.91 0.68 1.06 0.57 1.00 1.02 1.71 0.67 3.38 5.60 3.10 1.86
9.0 8.5 8.0 7.5 7.0 6.5 6.0 5.5 5.0 4.5 4.0 3.5 3.0 2.5 2.0 1.5 1.0 0.5 0.0
f1 (ppm)

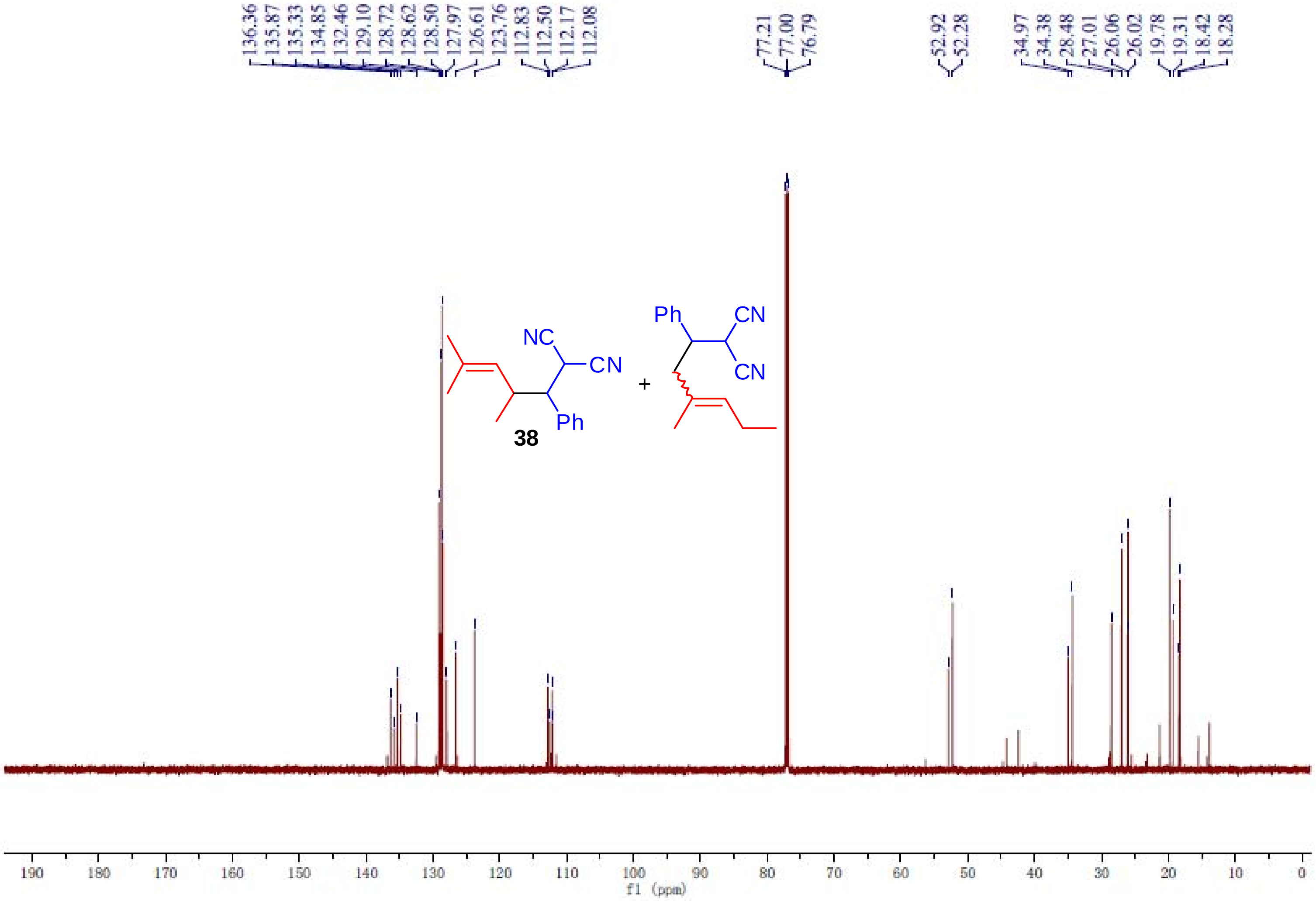
136.36
135.87
135.33
134.85
132.46
129.10
128.72
128.62
128.50
127.97
126.61
123.76
112.83
112.50
112.17
112.08
77.21
77.00
76.79
52.92
52.28
34.97
34.38
28.48
27.01
26.06
26.02
19.78
19.31
18.42
18.28
NC
CN
Ph
38
+
Ph
CN
CN
f1 (ppm)

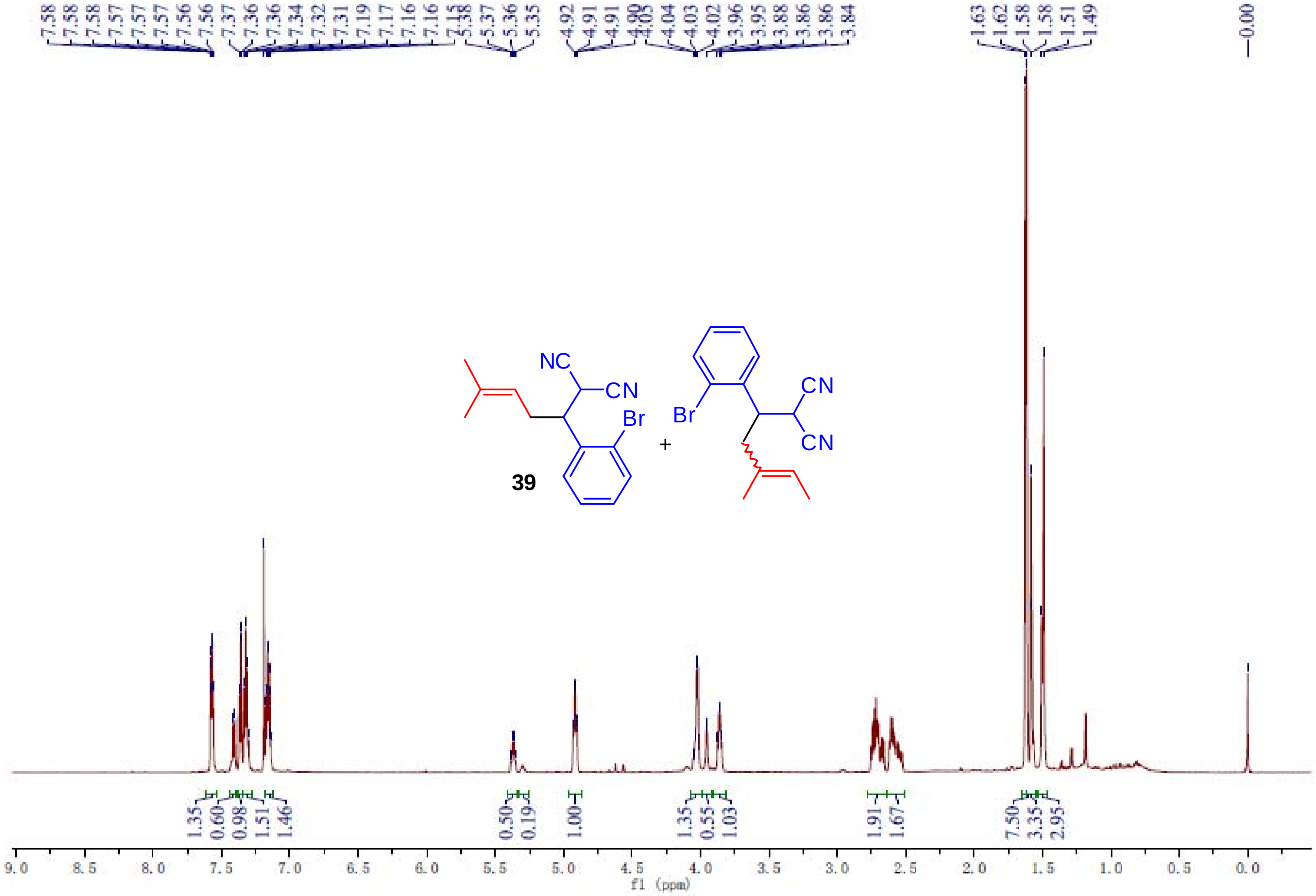

7.58
7.58
7.58
7.57
7.57
7.57
7.56
7.56
7.37
7.36
7.36
7.34
7.32
7.31
7.19
7.17
7.16
7.16
7.15
5.38
5.37
5.36
5.35
4.92
4.91
4.91
4.90
4.05
4.04
4.03
4.02
3.96
3.95
3.88
3.86
3.86
3.84
1.63
1.62
1.58
1.58
1.51
1.49
0.00
NC
CN
Br
39
+
Br
CN
CN
1.35
0.60
0.98
1.51
1.46
0.50
0.19
1.00
1.35
0.55
1.03
1.91
1.67
7.50
3.35
2.95
9.0
8.5
8.0
7.5
7.0
6.5
6.0
5.5
5.0
4.5
4.0
3.5
3.0
2.5
2.0
1.5
1.0
0.5
0.0
f1 (ppm)

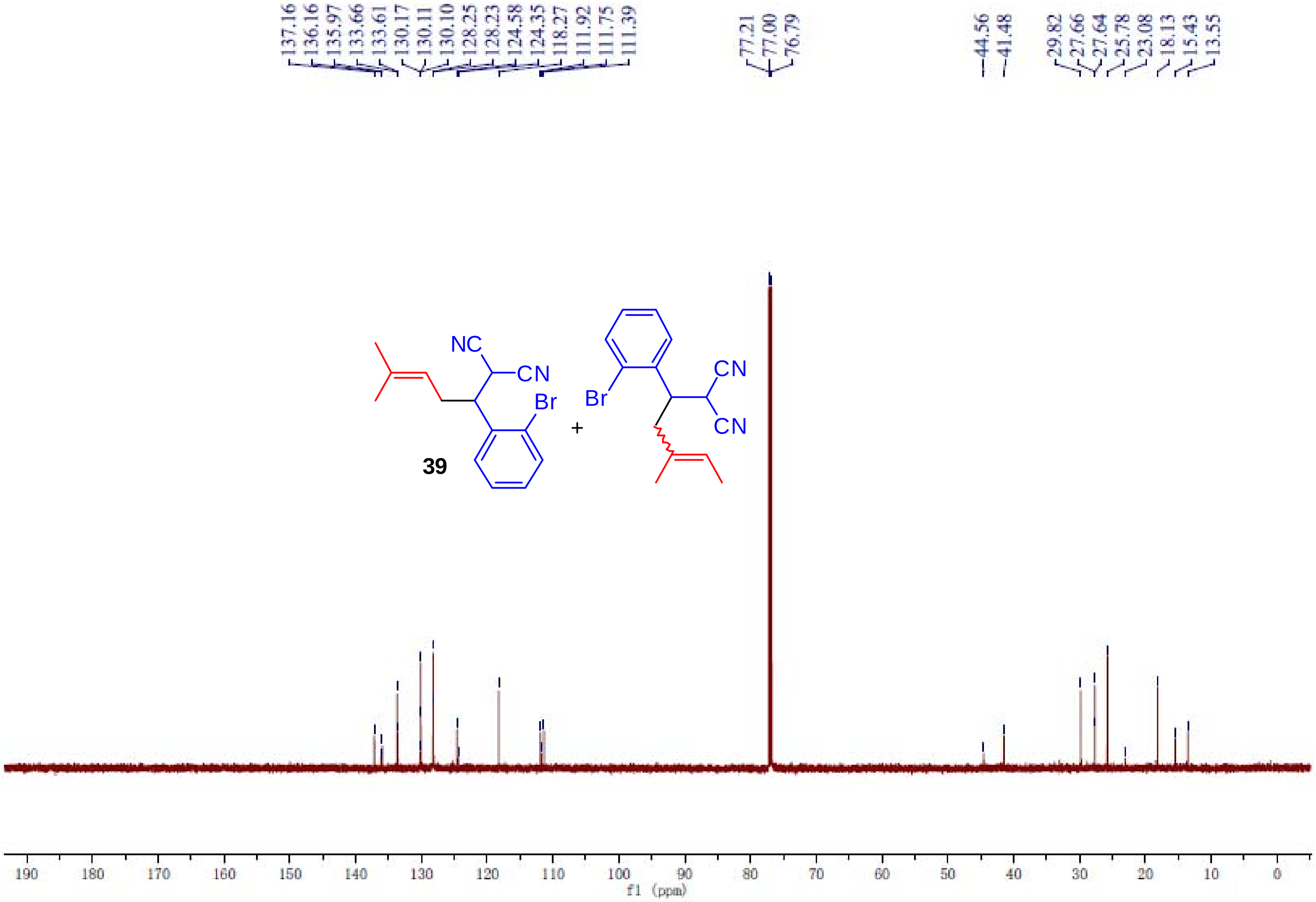

137.16
136.16
135.97
133.66
133.61
130.17
130.11
130.10
128.25
128.23
124.58
124.35
118.27
111.92
111.75
111.39
77.21
77.00
76.79
44.56
41.48
29.82
27.66
27.64
25.78
23.08
18.13
15.43
13.55
NC
CN
Br
39
+
Br
CN
CN
190
180
170
160
150
140
130
120
110
100
90
80
70
60
50
40
30
20
10
0
f1 (ppm)

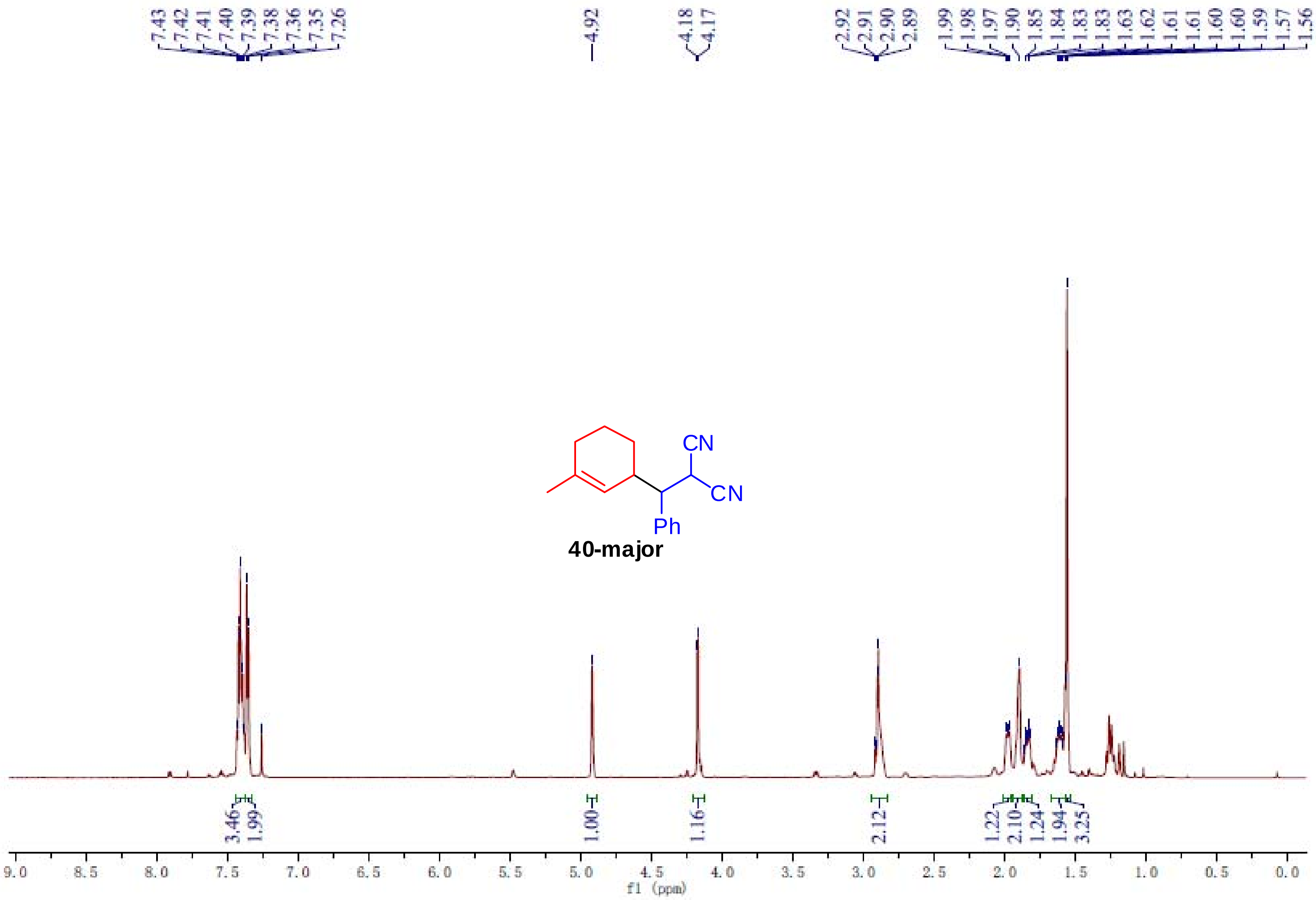
7.43
7.42
7.41
7.40
7.39
7.38
7.36
7.35
7.26
4.92
4.18
4.17
2.92
2.91
2.90
2.89
1.99
1.98
1.97
1.90
1.85
1.84
1.83
1.83
1.63
1.62
1.61
1.61
1.60
1.60
1.59
1.57
1.56
CN
CN
Ph
40-major
3.46
1.99
1.00
1.16
2.12
1.22
2.10
1.24
1.94
3.25
9.0
8.5
8.0
7.5
7.0
6.5
6.0
5.5
5.0
4.5
4.0
3.5
3.0
2.5
2.0
1.5
1.0
0.5
0.0
f1 (ppm)

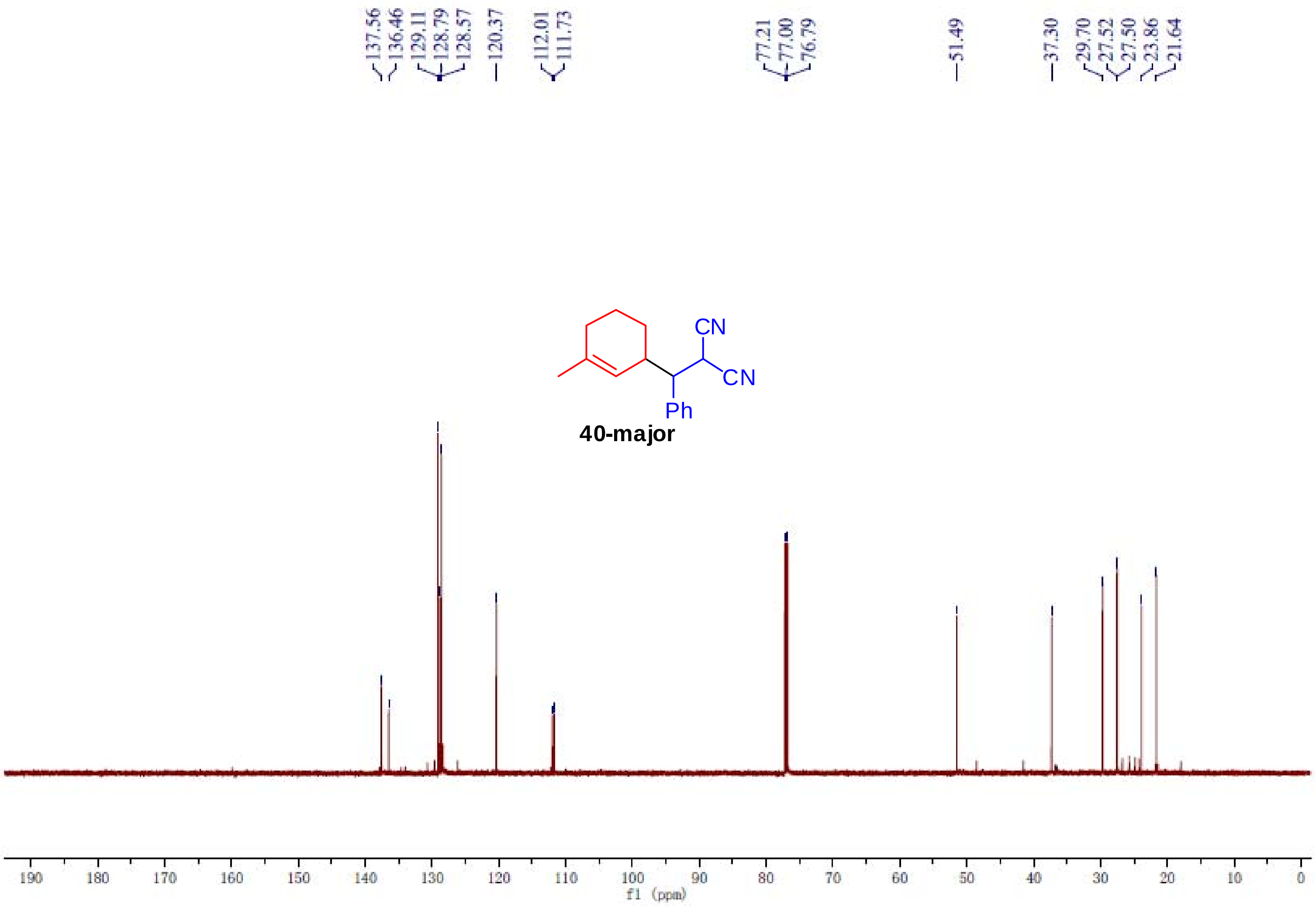
137.56
136.46
129.11
128.79
128.57
120.37
112.01
111.73
77.21
77.00
76.79
51.49
37.30
29.70
27.52
27.50
23.86
21.64
CN
CN
Ph
40-major
190
180
170
160
150
140
130
120
110
100
90
80
70
60
50
40
30
20
10
0
f1 (ppm)

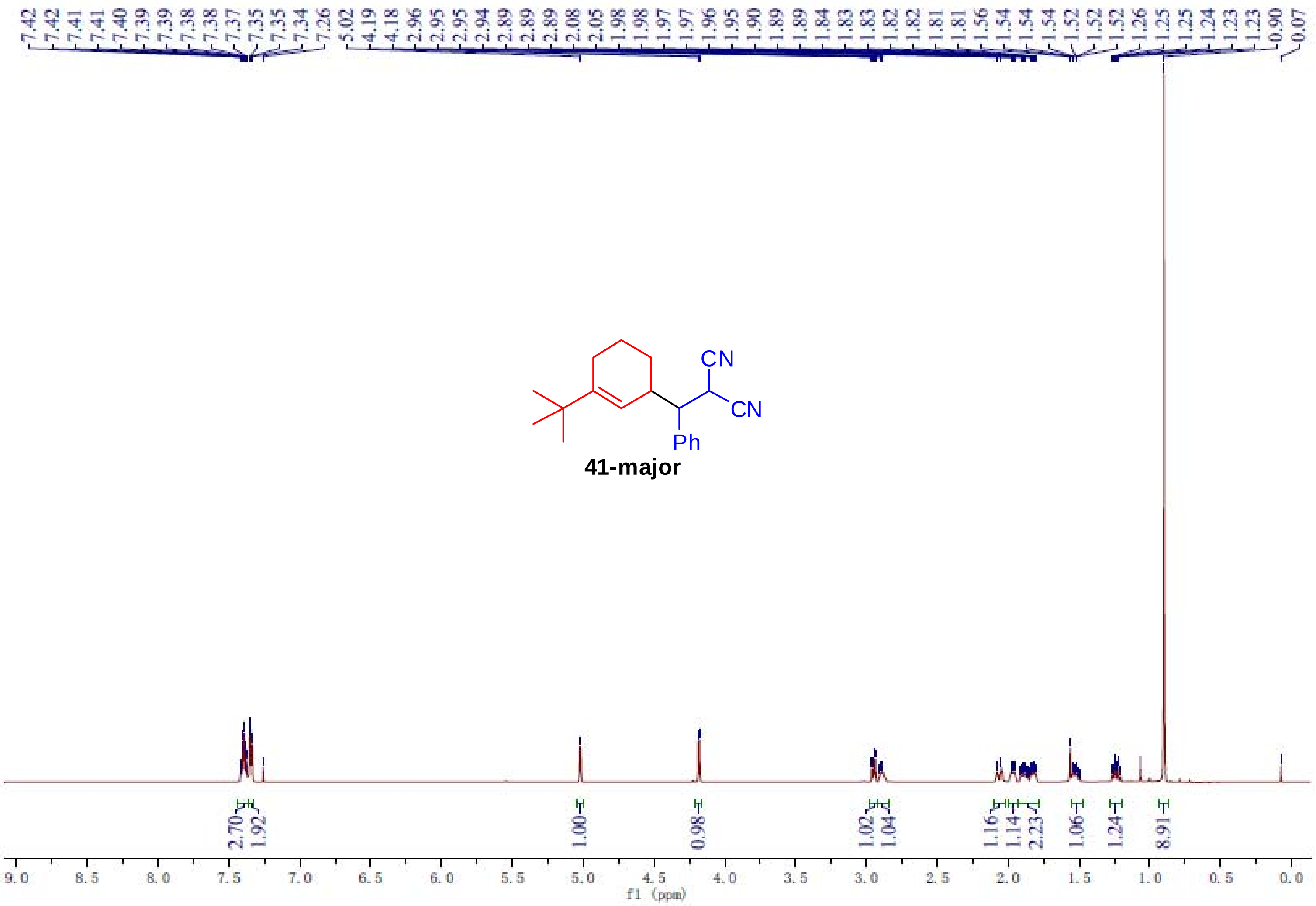

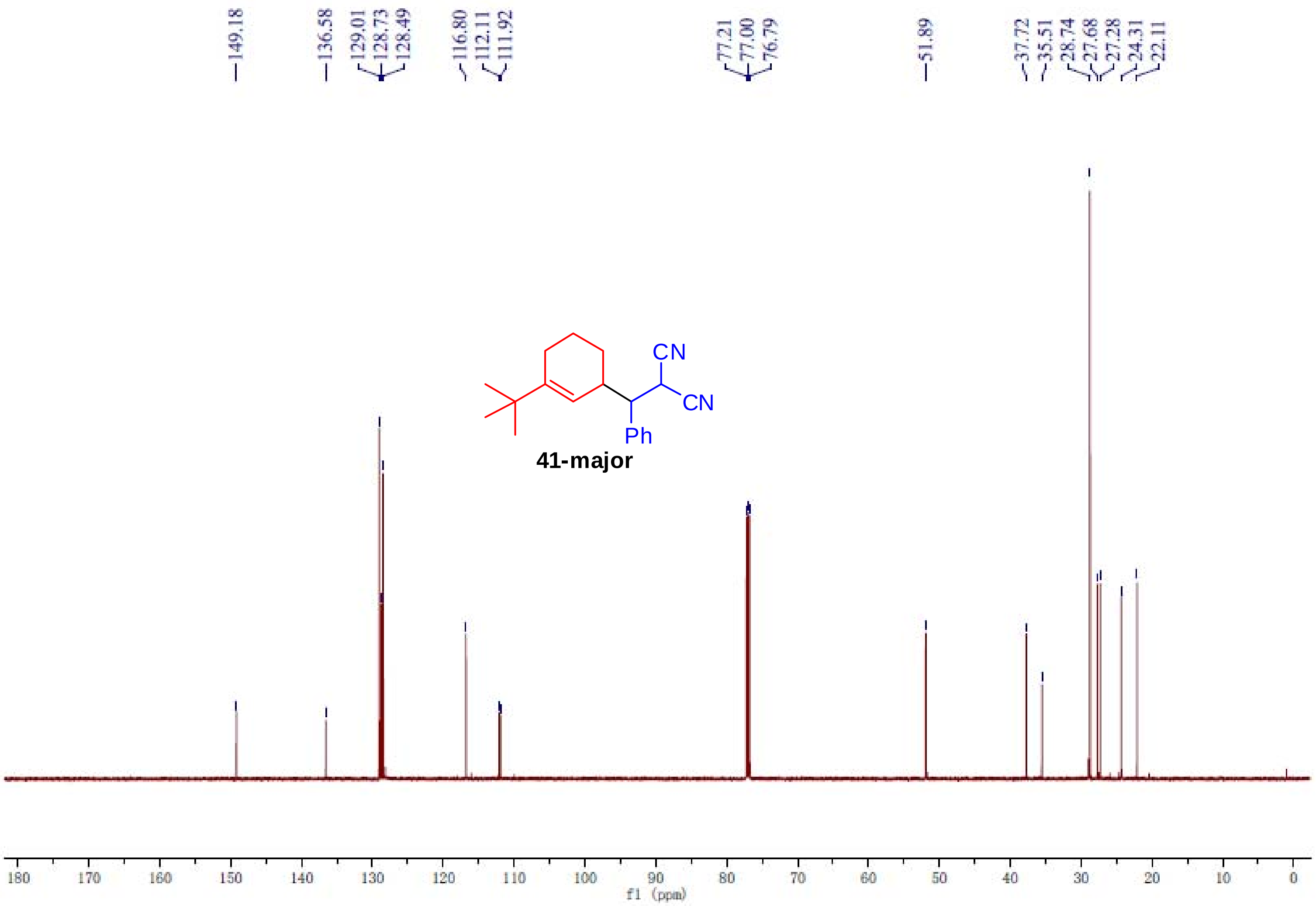
149.18
136.58
129.01
128.73
128.49
116.80
112.11
111.92
77.21
77.00
76.79
51.89
37.72
35.51
28.74
27.68
27.28
24.31
22.11
CN
CN
Ph
41-major
180 170 160 150 140 130 120 110 100 90 80 70 60 50 40 30 20 10 0
f1 (ppm)

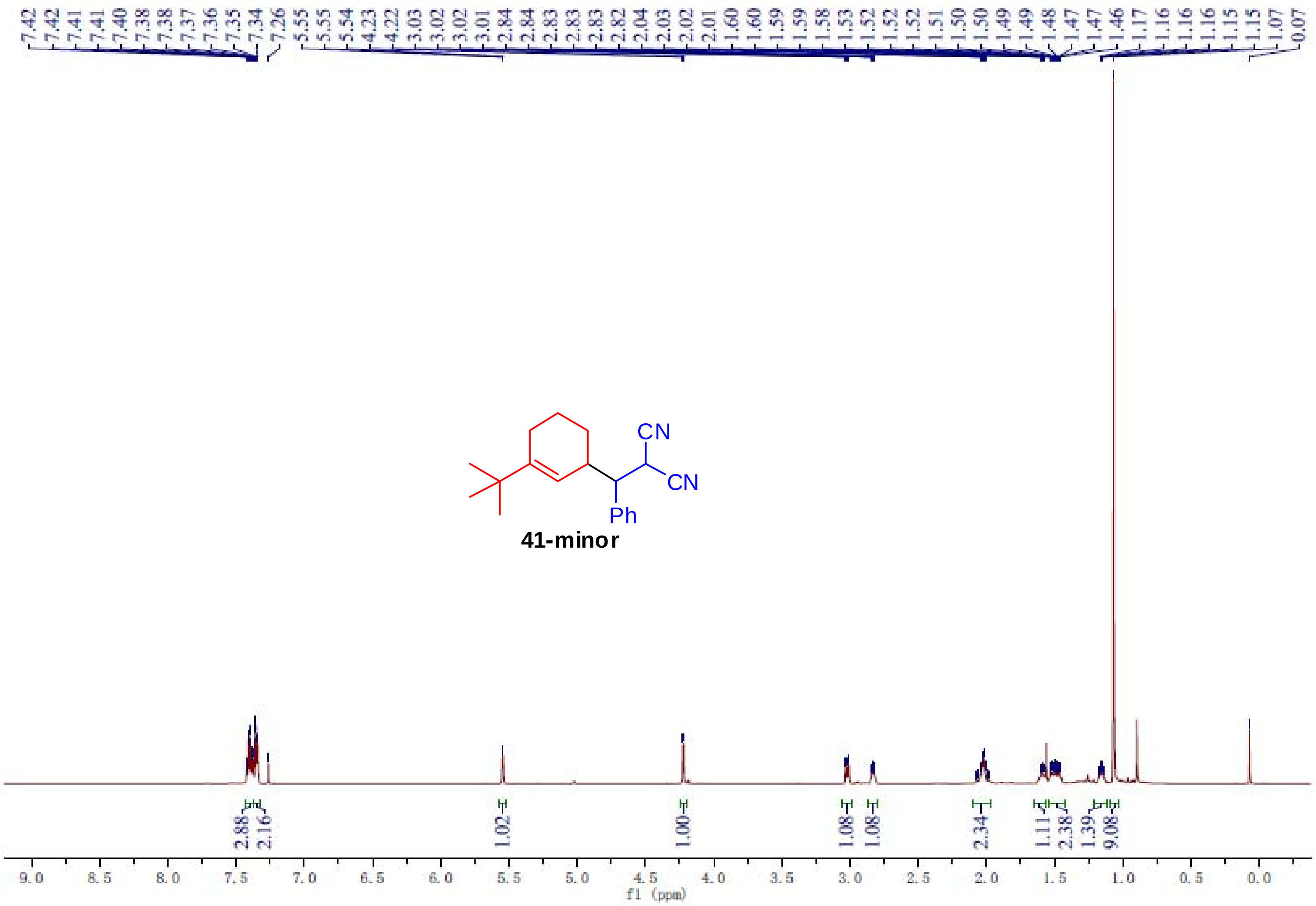

41-minor

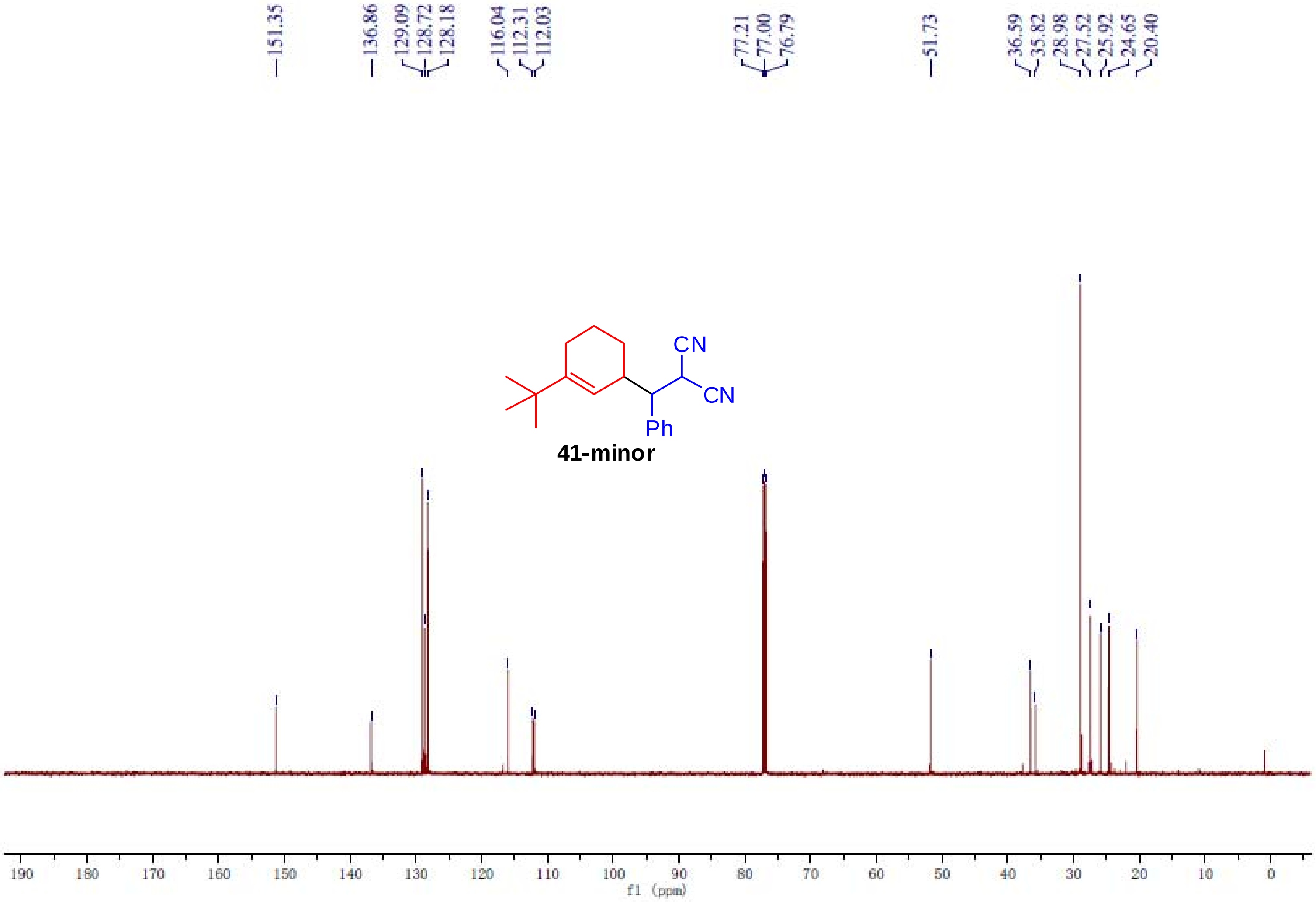
151.35
136.86
129.09
128.72
128.18
116.04
112.31
112.03
77.21
77.00
76.79
51.73
36.59
35.82
28.98
27.52
25.92
24.65
20.40
CN
CN
Ph
41-minor
190 180 170 160 150 140 130 120 110 100 90 80 70 60 50 40 30 20 10 0
f1 (ppm)

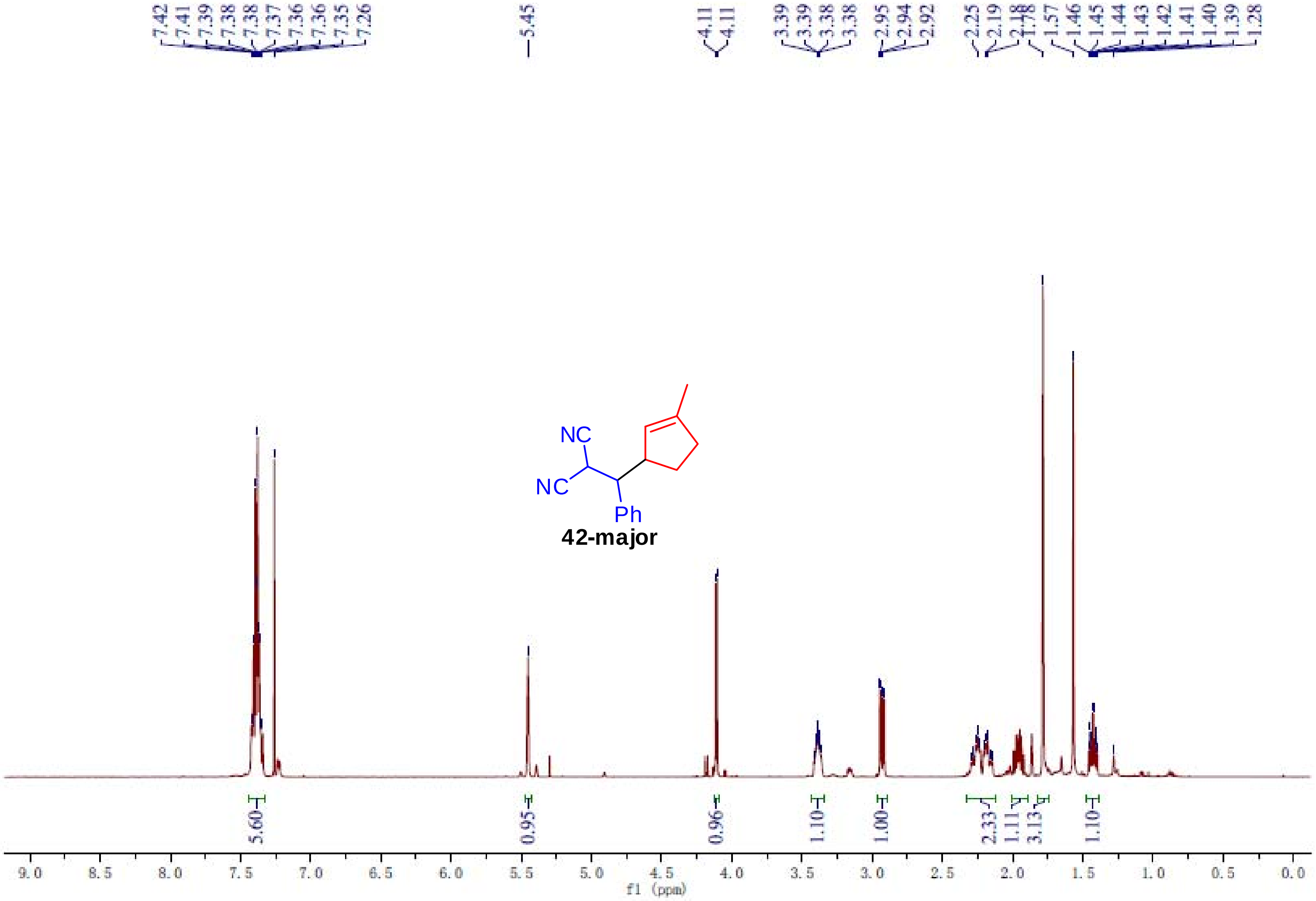
7.42 7.41 7.39 7.38 7.38 7.37 7.36 7.36 7.35 7.26
5.45
4.11 4.11
3.39 3.39 3.38 3.38
2.95 2.94 2.92
2.25 2.19 2.18 1.78 1.57 1.46 1.45 1.44 1.43 1.42 1.41 1.40 1.39 1.28
NC
NC
Ph
42-major
5.60
0.95
0.96
1.10
1.00
2.33
1.11
3.13
1.10
9.0 8.5 8.0 7.5 7.0 6.5 6.0 5.5 5.0 4.5 4.0 3.5 3.0 2.5 2.0 1.5 1.0 0.5 0.0
f1 (ppm)

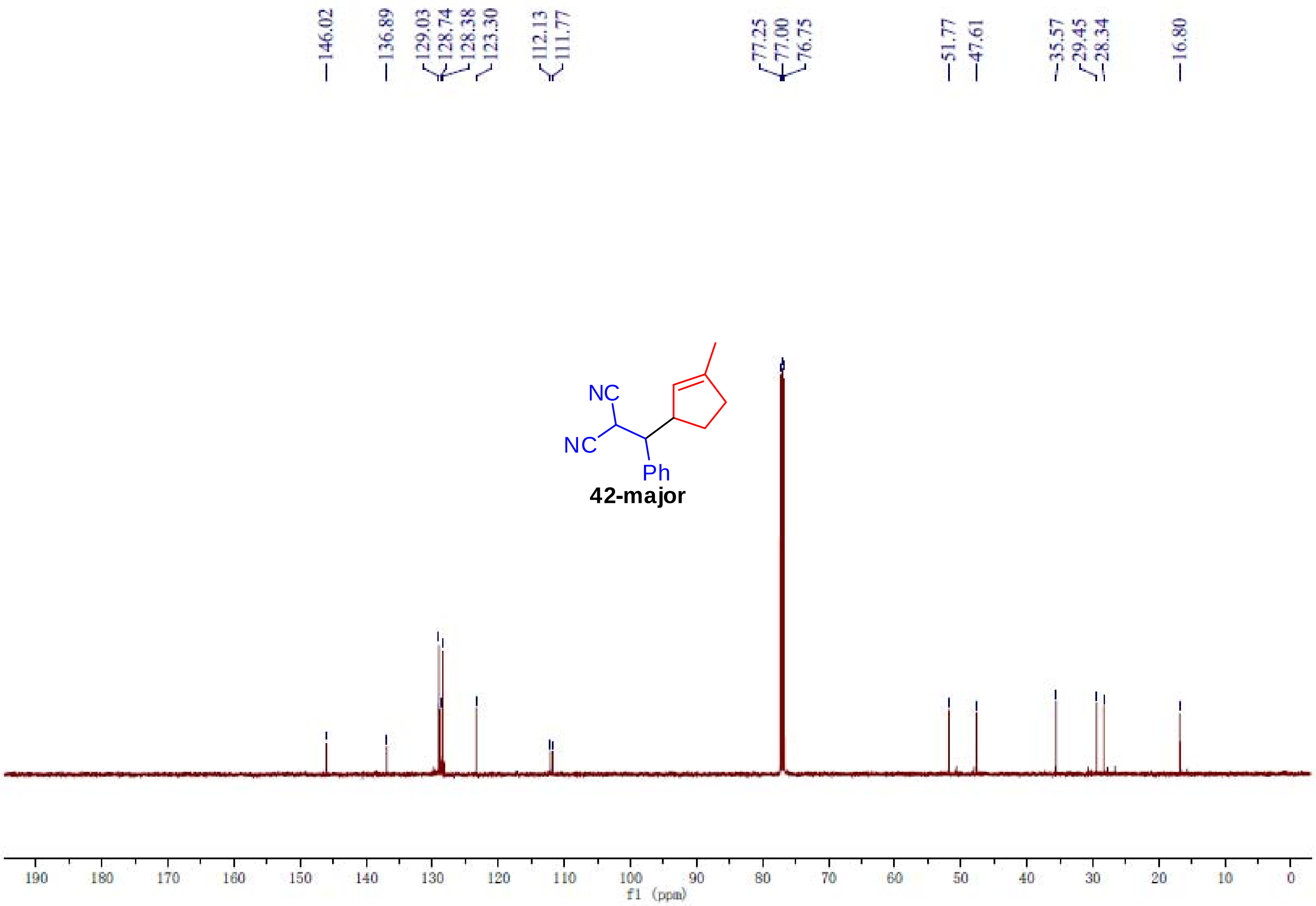
146.02
136.89
129.03
128.74
128.38
123.30
112.13
111.77
77.25
77.00
76.75
51.77
47.61
35.57
29.45
28.34
16.80
NC
NC
Ph
42-major
f1 (ppm)

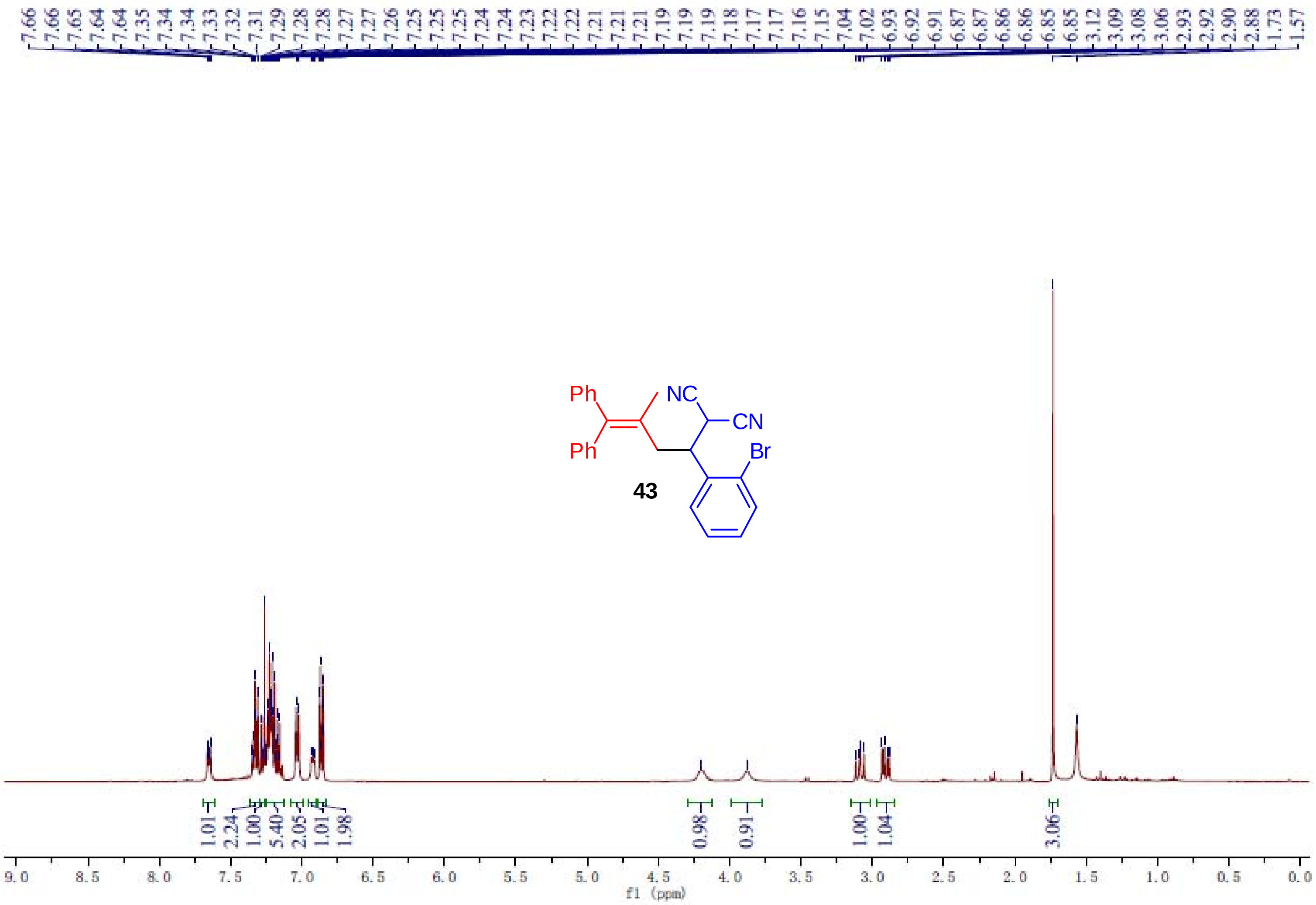

7.66 7.66 7.65 7.64 7.64 7.35 7.34 7.34 7.33 7.32 7.31 7.29 7.28 7.28 7.27 7.27 7.26 7.25 7.25 7.25 7.24 7.24 7.23 7.22 7.22 7.21 7.21 7.21 7.19 7.19 7.19 7.18 7.17 7.17 7.16 7.15 7.04 7.02 6.93 6.92 6.91 6.87 6.87 6.86 6.86 6.85 6.85 3.12 3.09 3.08 3.06 2.93 2.92 2.90 2.88 1.73 1.57
Ph
Ph
NC
CN
Br
43
1.01 2.24 1.00 5.40 2.05 1.01 1.98 0.98 0.91 1.00 1.04 3.06
9.0 8.5 8.0 7.5 7.0 6.5 6.0 5.5 5.0 4.5 4.0 3.5 3.0 2.5 2.0 1.5 1.0 0.5 0.0
f1 (ppm)

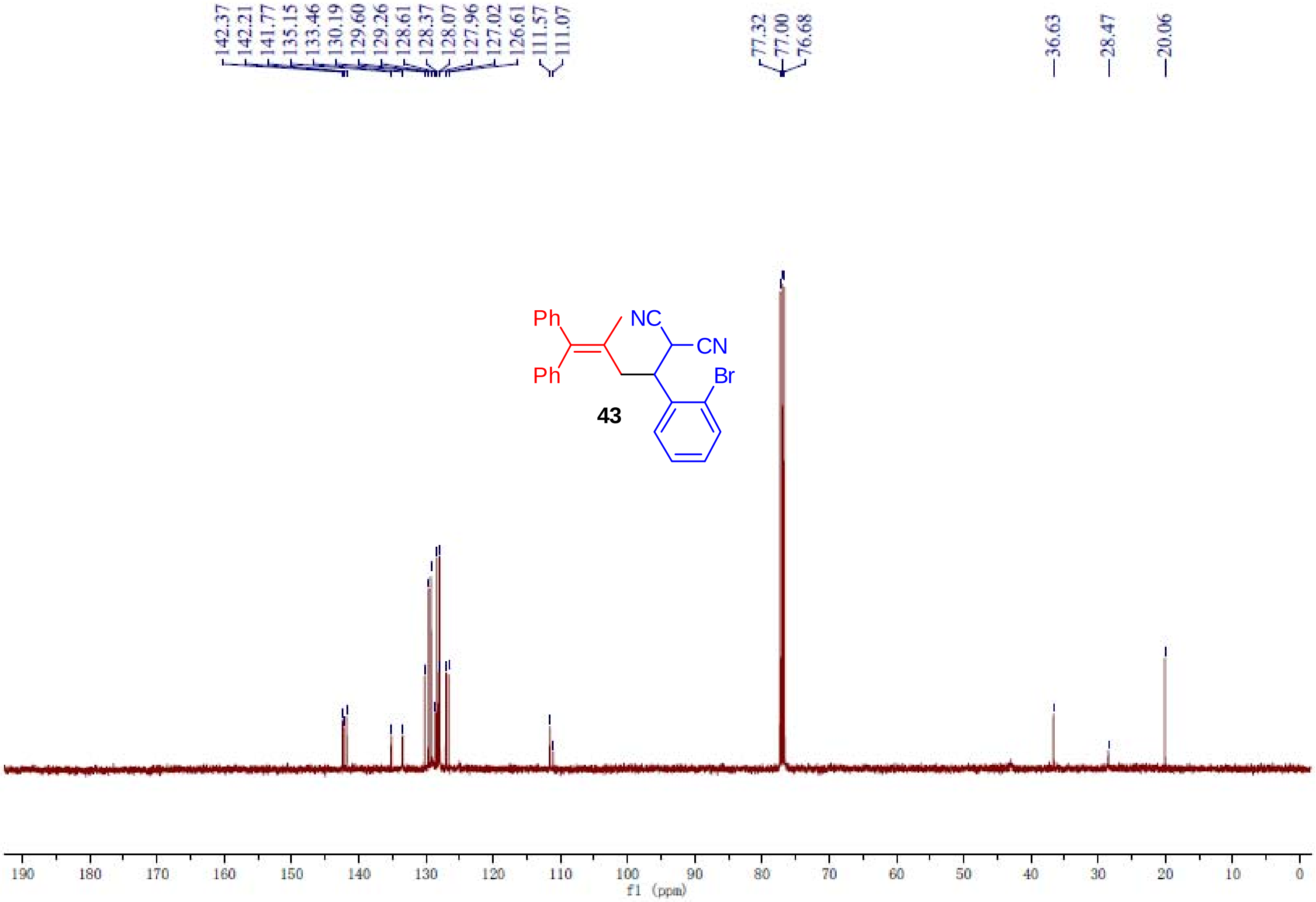

142.37
142.21
141.77
135.15
133.46
130.19
129.60
129.26
128.61
128.37
128.07
127.96
127.02
126.61
111.57
111.07
77.32
77.00
76.68
36.63
28.47
20.06
Ph
Ph
NC
CN
Br
43
190 180 170 160 150 140 130 120 110 100 90 80 70 60 50 40 30 20 10 0
f1 (ppm)

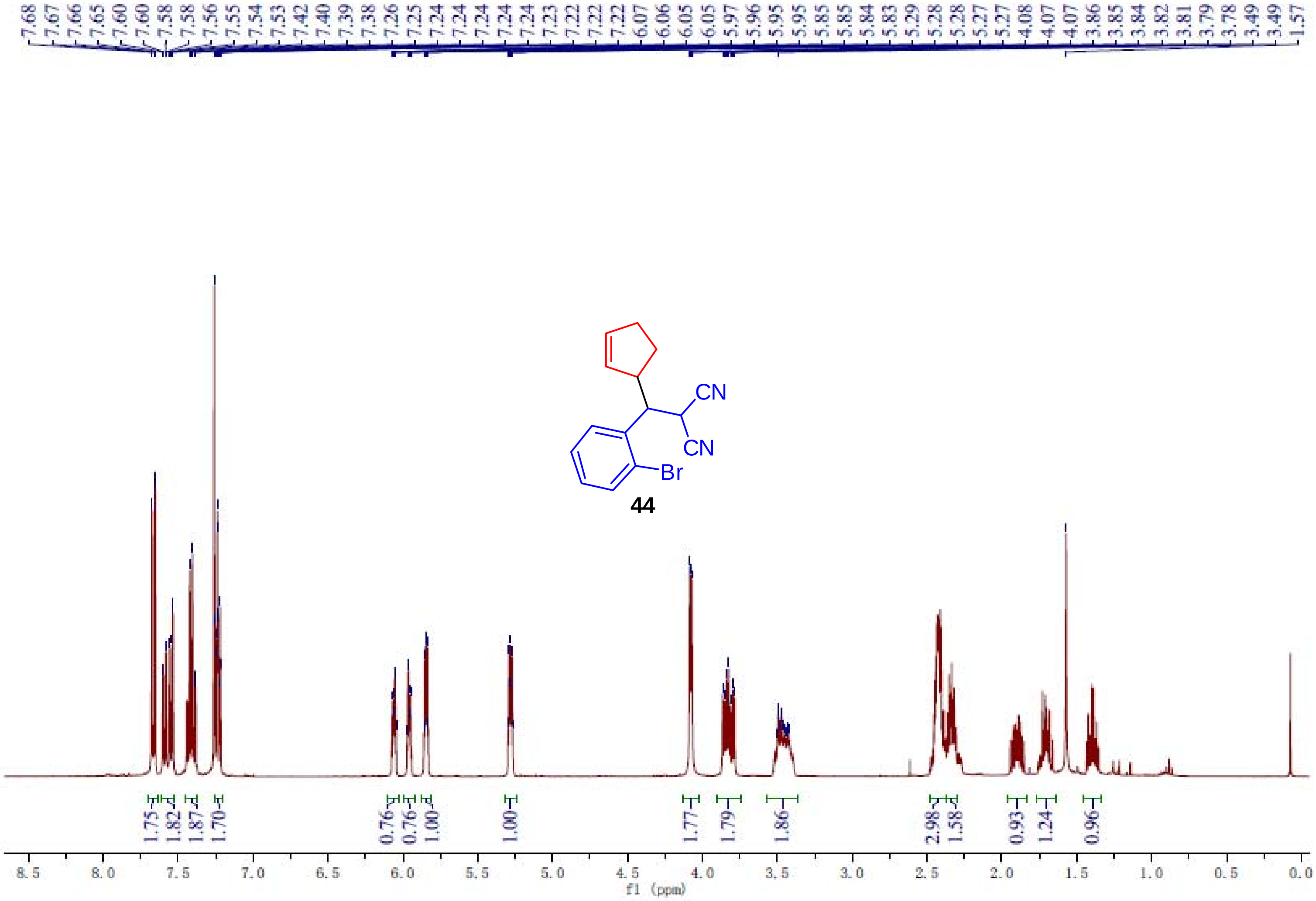
7.68 7.67 7.66 7.65 7.60 7.60 7.58 7.58 7.56 7.55 7.54 7.53 7.42 7.40 7.39 7.38 7.26 7.25 7.24 7.24 7.24 7.24 7.24 7.24 7.23 7.22 7.22 7.22 7.22 6.07 6.06 6.05 6.05 5.97 5.96 5.95 5.95 5.85 5.85 5.84 5.83 5.29 5.28 5.28 5.27 5.27 4.08 4.07 4.07 3.86 3.85 3.84 3.82 3.81 3.79 3.78 3.49 3.49 1.57
CN
CN
Br
44
1.75 1.82 1.87 1.70 0.76 0.76 1.00 1.00 1.77 1.79 1.86 2.98 1.58 0.93 1.24 0.96
8.5 8.0 7.5 7.0 6.5 6.0 5.5 5.0 4.5 4.0 3.5 3.0 2.5 2.0 1.5 1.0 0.5 0.0
f1 (ppm)

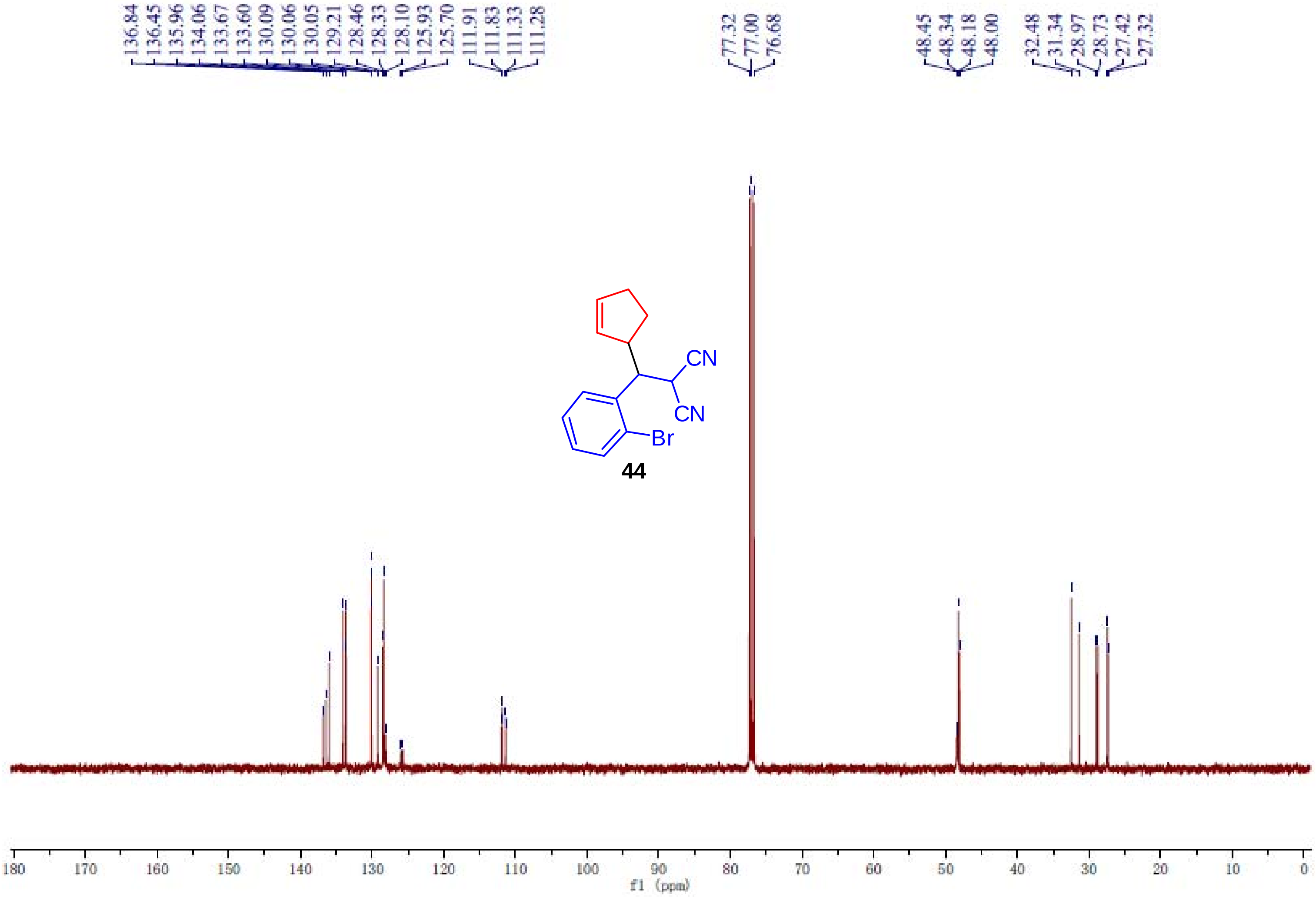
136.84
136.45
135.96
134.06
133.67
133.60
130.09
130.06
130.05
129.21
128.46
128.33
128.10
125.93
125.70
111.91
111.83
111.33
111.28
77.32
77.00
76.68
48.45
48.34
48.18
48.00
32.48
31.34
28.97
28.73
27.42
27.32
CN
CN
Br
44
f1 (ppm)

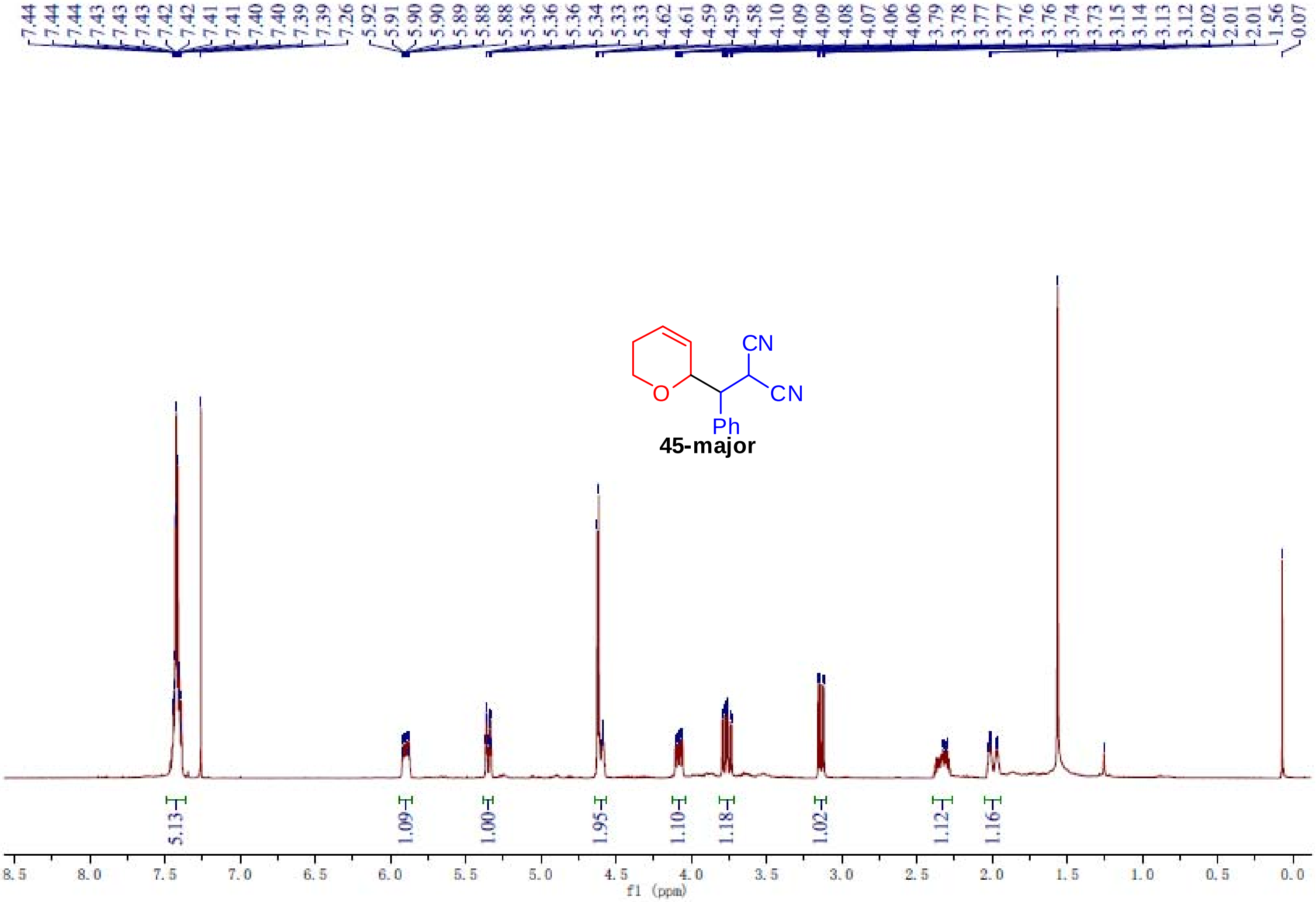
7.44 7.44 7.44 7.43 7.43 7.43 7.42 7.42 7.41 7.41 7.40 7.40 7.39 7.39 7.26 5.92 5.91 5.90 5.90 5.89 5.88 5.88 5.36 5.36 5.36 5.34 5.33 5.33 4.62 4.61 4.59 4.59 4.58 4.10 4.09 4.08 4.07 4.06 4.06 3.79 3.78 3.77 3.77 3.76 3.76 3.74 3.73 3.15 3.14 3.13 3.12 2.02 2.01 2.01 1.56 0.07
CN
CN
O
Ph
45-major
5.13
1.09
1.00
1.95
1.10
1.18
1.02
1.12
1.16
8.5 8.0 7.5 7.0 6.5 6.0 5.5 5.0 4.5 4.0 3.5 3.0 2.5 2.0 1.5 1.0 0.5 0.0
f1 (ppm)

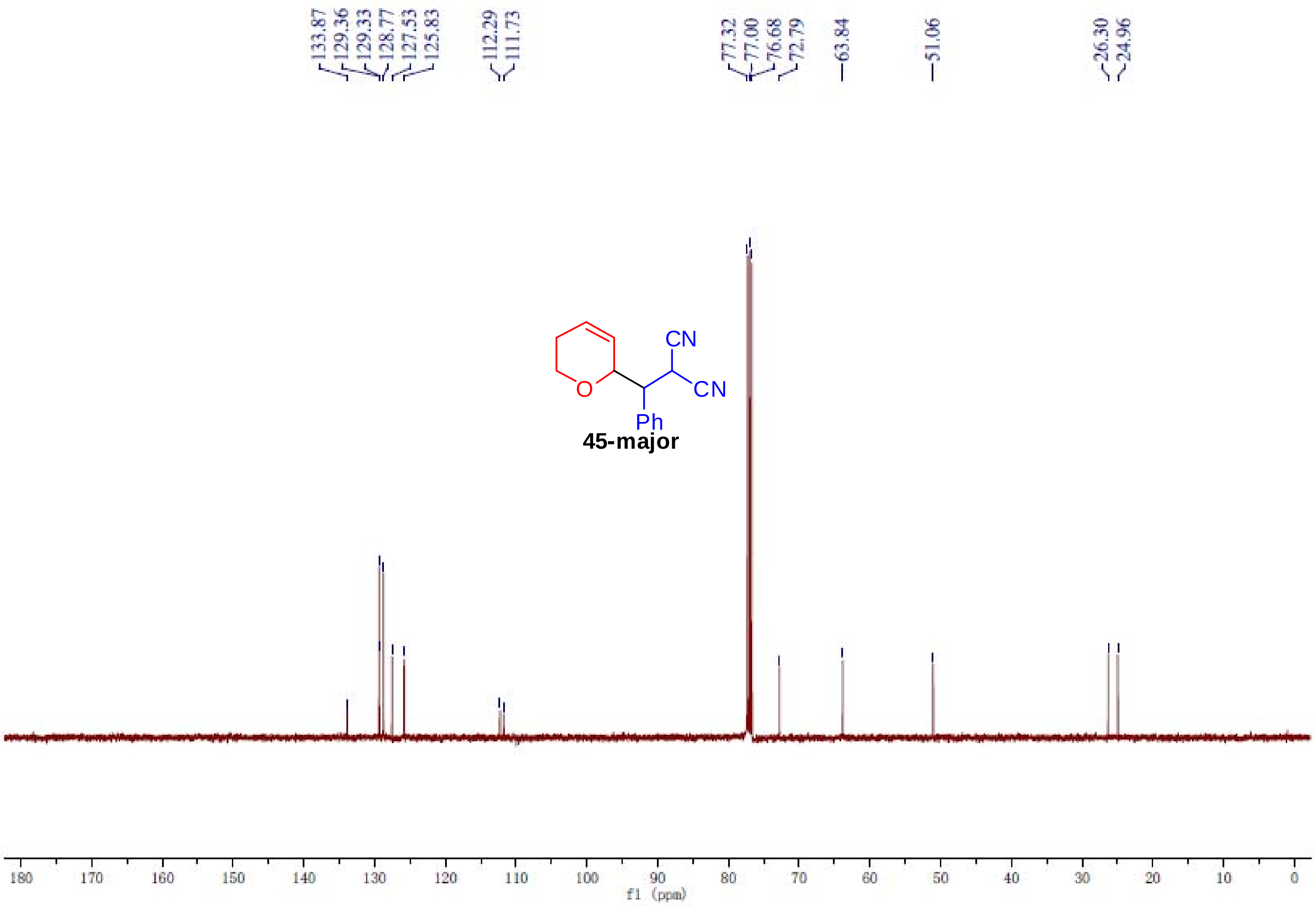
133.87
129.36
129.33
128.77
127.53
125.83
112.29
111.73
77.32
77.00
76.68
72.79
63.84
51.06
26.30
24.96
CN
CN
O
Ph
45-major
180 170 160 150 140 130 120 110 100 90 80 70 60 50 40 30 20 10 0
f1 (ppm)

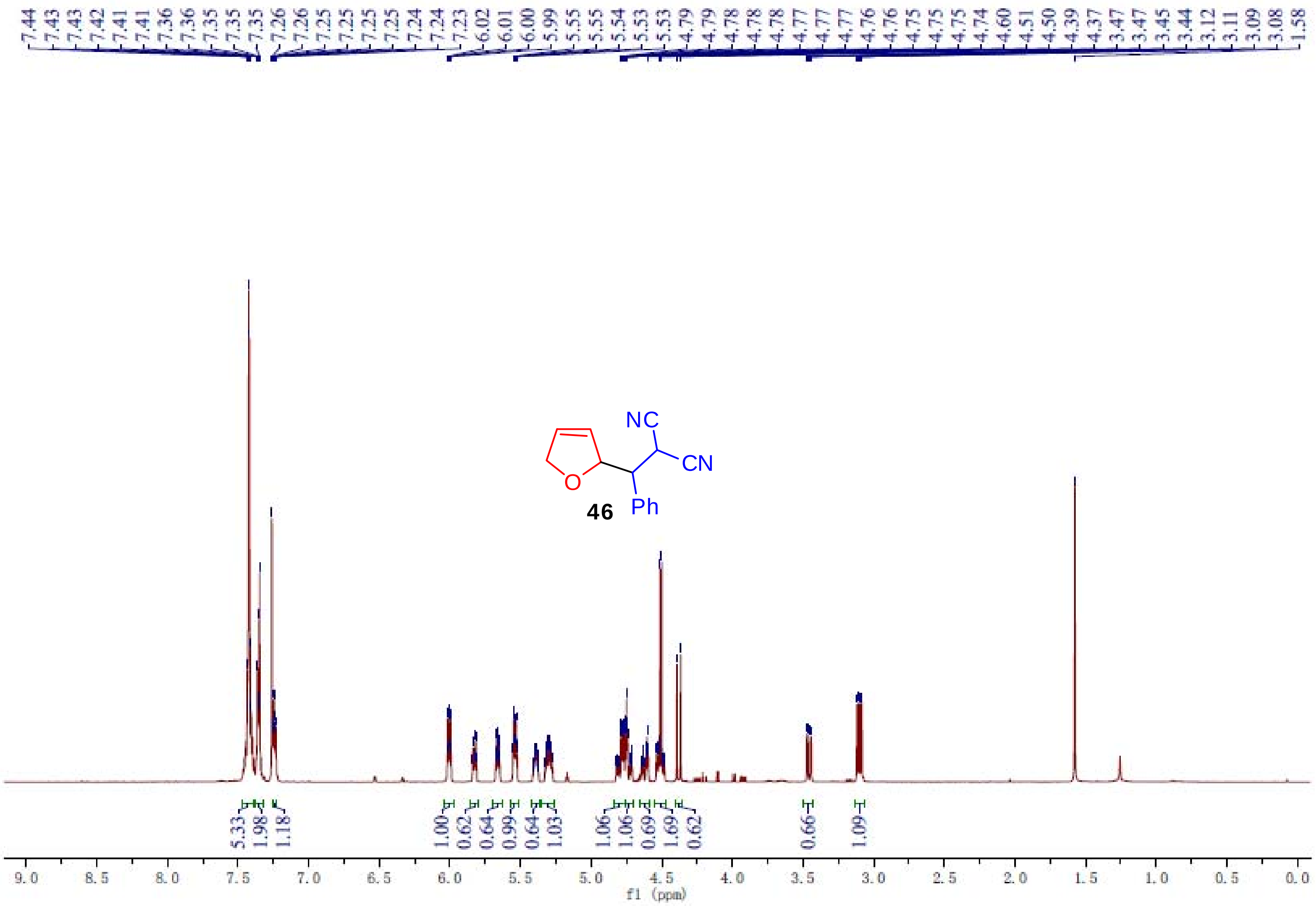
7.44 7.43 7.43 7.42 7.41 7.41 7.36 7.36 7.35 7.35 7.35 7.26 7.26 7.25 7.25 7.25 7.25 7.24 7.24 7.23 6.02 6.01 6.00 5.99 5.55 5.55 5.54 5.53 5.53 4.79 4.79 4.78 4.78 4.78 4.77 4.77 4.77 4.76 4.76 4.75 4.75 4.75 4.74 4.60 4.51 4.50 4.39 4.37 3.47 3.47 3.45 3.44 3.12 3.11 3.09 3.08 1.58
NC
CN
O
Ph
46
5.33 1.98 1.18 1.00 0.62 0.64 0.99 0.64 1.03 1.06 1.06 0.69 1.69 0.62 0.66 1.09
9.0 8.5 8.0 7.5 7.0 6.5 6.0 5.5 5.0 4.5 4.0 3.5 3.0 2.5 2.0 1.5 1.0 0.5 0.0
f1 (ppm)

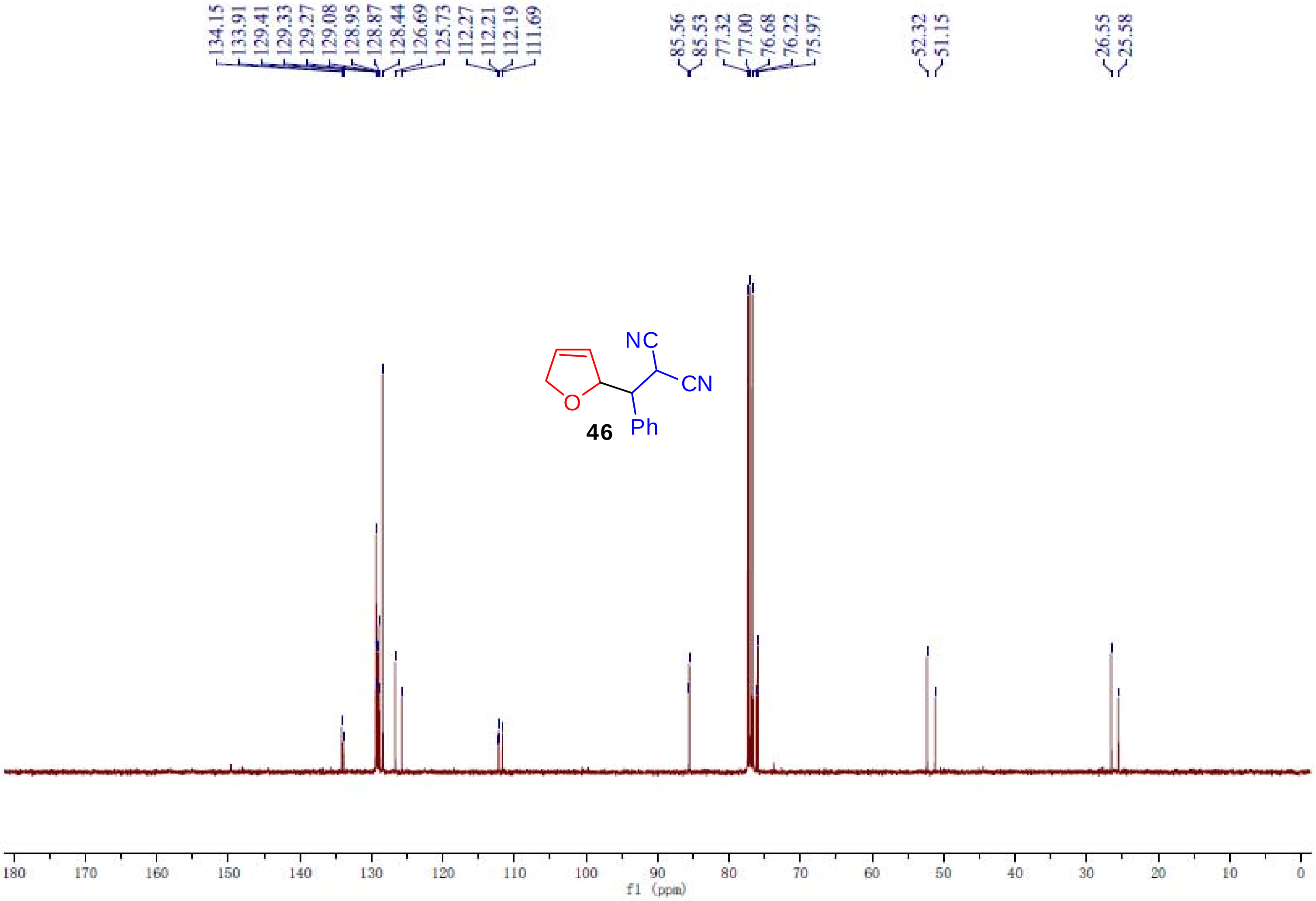
134.15
133.91
129.41
129.33
129.27
129.08
128.95
128.87
128.44
126.69
125.73
112.27
112.21
112.19
111.69
85.56
85.53
77.32
77.00
76.68
76.22
75.97
52.32
51.15
26.55
25.58
NC
CN
O
46
Ph
f1 (ppm)

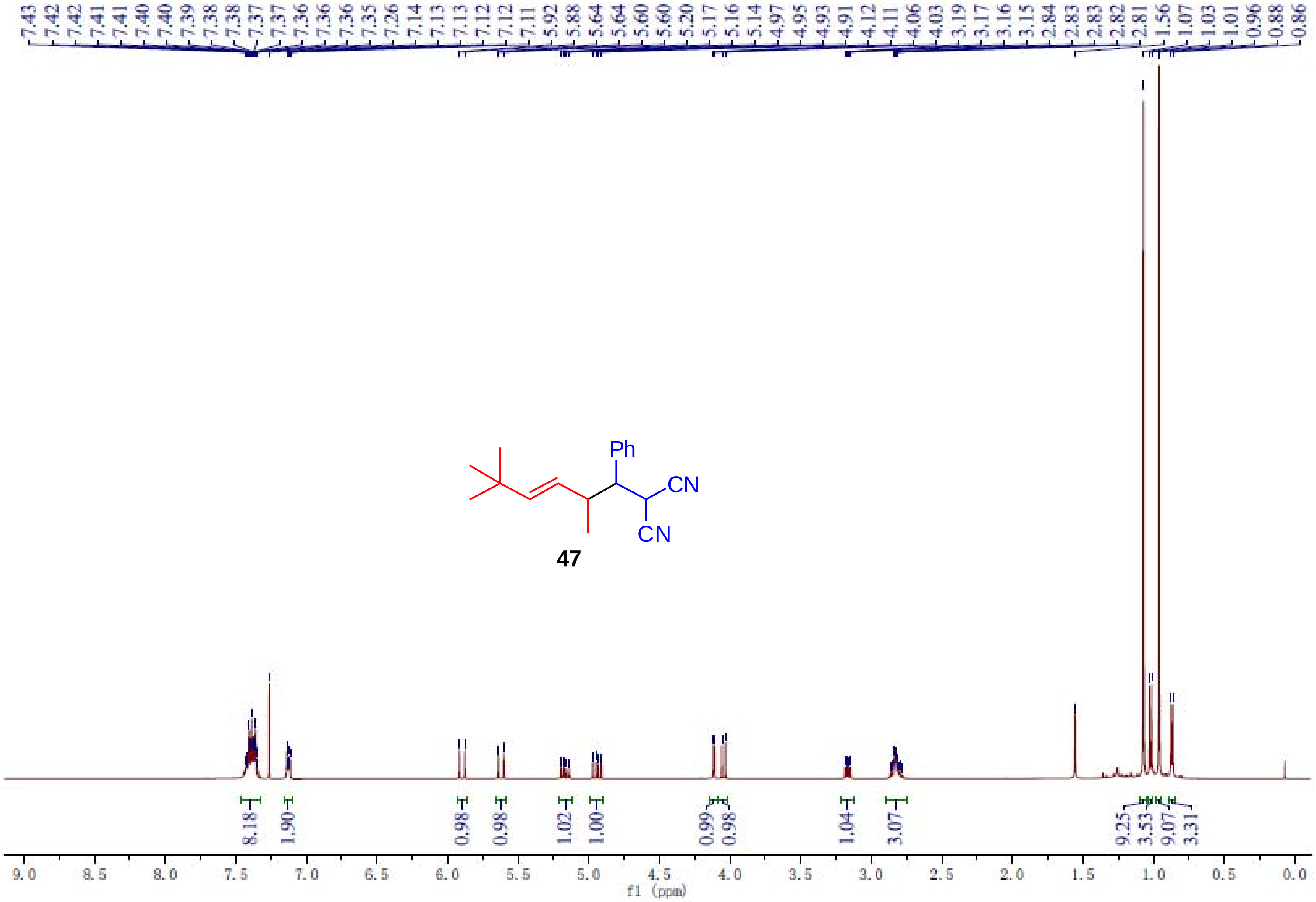

47

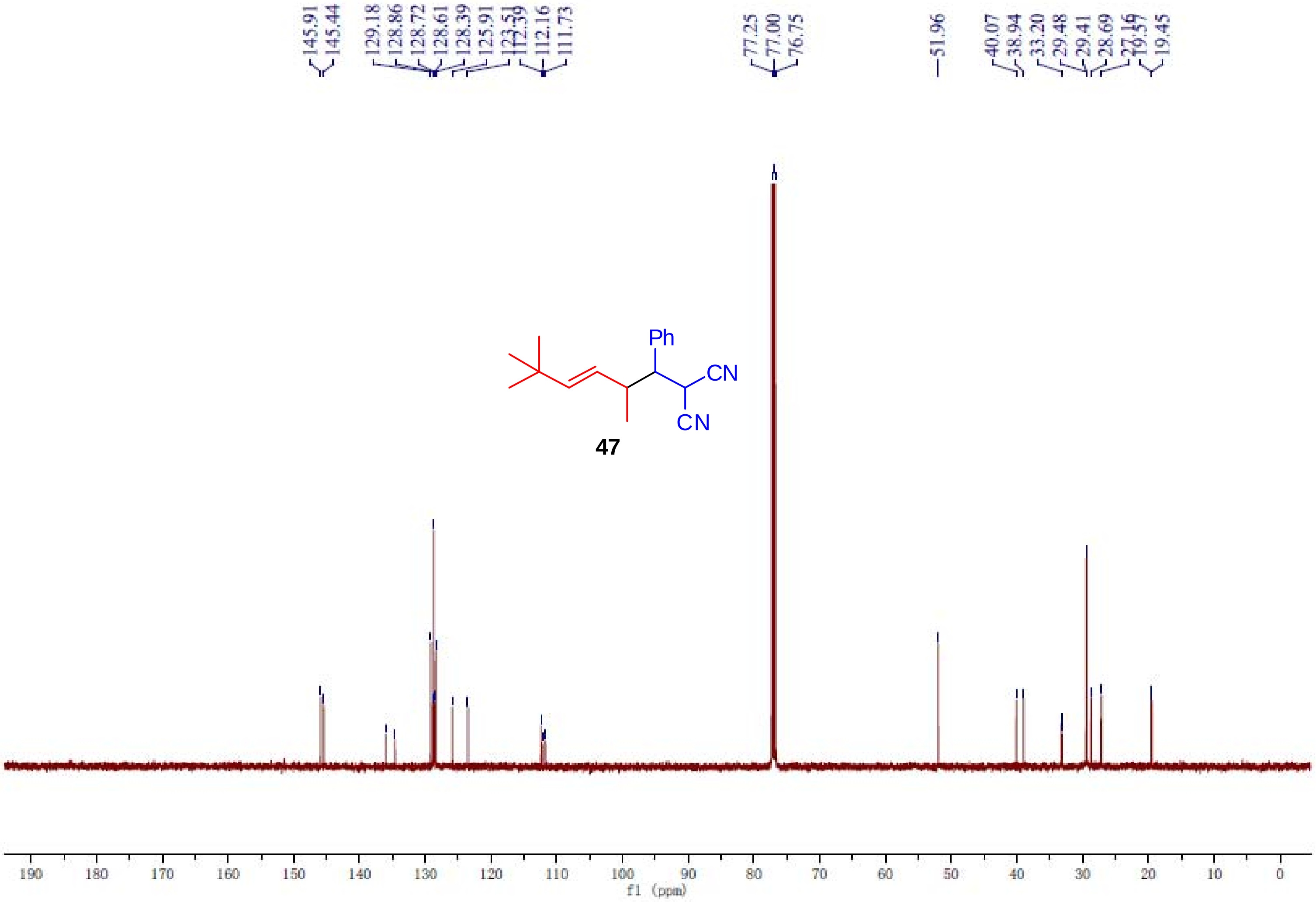
145.91
145.44
129.18
128.86
128.72
128.61
128.39
125.91
123.51
112.16
111.73
77.25
77.00
76.75
51.96
40.07
38.94
33.20
29.48
29.41
28.69
27.16
19.57
19.45
Ph
CN
CN
47
190 180 170 160 150 140 130 120 110 100 90 80 70 60 50 40 30 20 10 0
f1 (ppm)

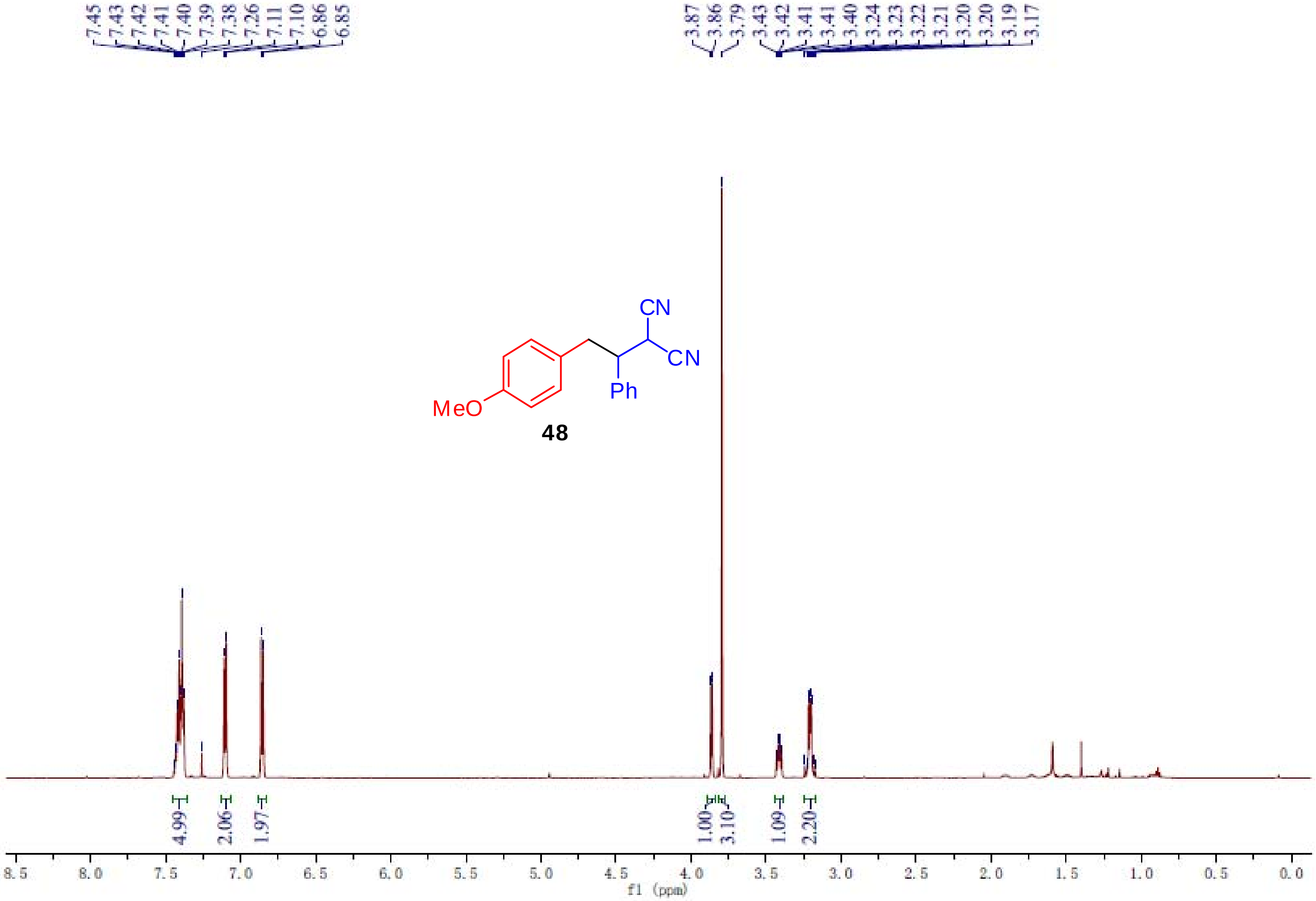
7.45
7.43
7.42
7.41
7.40
7.39
7.38
7.26
7.11
7.10
6.86
6.85
3.87
3.86
3.79
3.43
3.42
3.41
3.41
3.40
3.24
3.23
3.22
3.21
3.20
3.20
3.19
3.17
CN
CN
Ph
MeO
48
4.99
2.06
1.97
1.00
3.10
1.09
2.20
8.5
8.0
7.5
7.0
6.5
6.0
5.5
5.0
4.5
4.0
3.5
3.0
2.5
2.0
1.5
1.0
0.5
0.0
f1 (ppm)

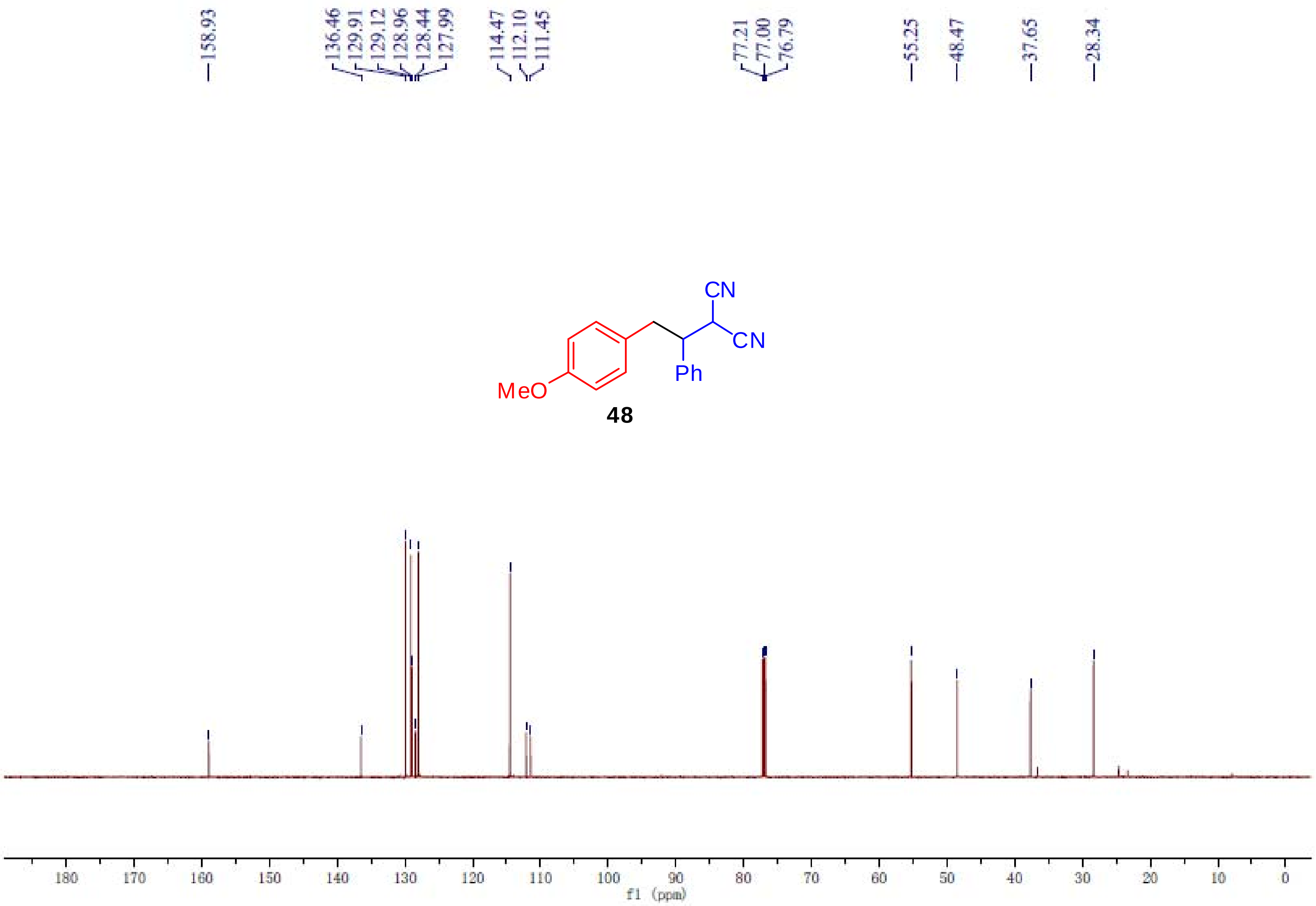
158.93
136.46
129.91
129.12
128.96
128.44
127.99
114.47
112.10
111.45
77.21
77.00
76.79
55.25
48.47
37.65
28.34
CN
CN
Ph
MeO
48
180
170
160
150
140
130
120
110
100
90
80
70
60
50
40
30
20
10
0
f1 (ppm)

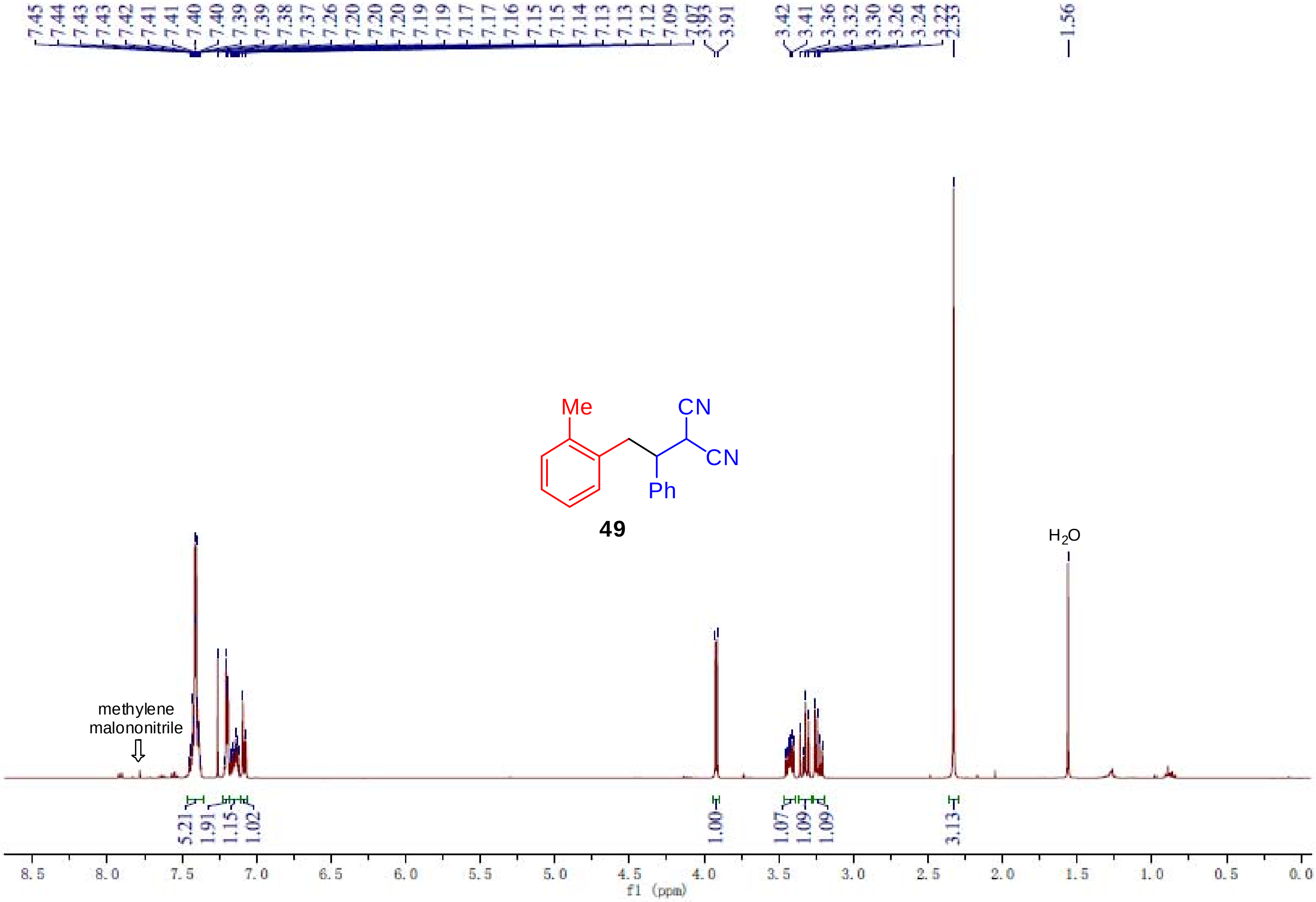
7.45
7.44
7.43
7.43
7.42
7.41
7.41
7.40
7.40
7.39
7.39
7.38
7.37
7.26
7.20
7.20
7.20
7.19
7.19
7.17
7.17
7.16
7.15
7.15
7.14
7.13
7.13
7.12
7.09
7.07
3.93
3.91
3.42
3.41
3.36
3.32
3.30
3.26
3.24
3.22
2.33
1.56
Me
CN
CN
Ph
49
H_2O
methylene
malononitrile
5.21
1.91
1.15
1.02
1.00
1.07
1.09
1.09
3.13
8.5
8.0
7.5
7.0
6.5
6.0
5.5
5.0
4.5
4.0
3.5
3.0
2.5
2.0
1.5
1.0
0.5
0.0
f1 (ppm)

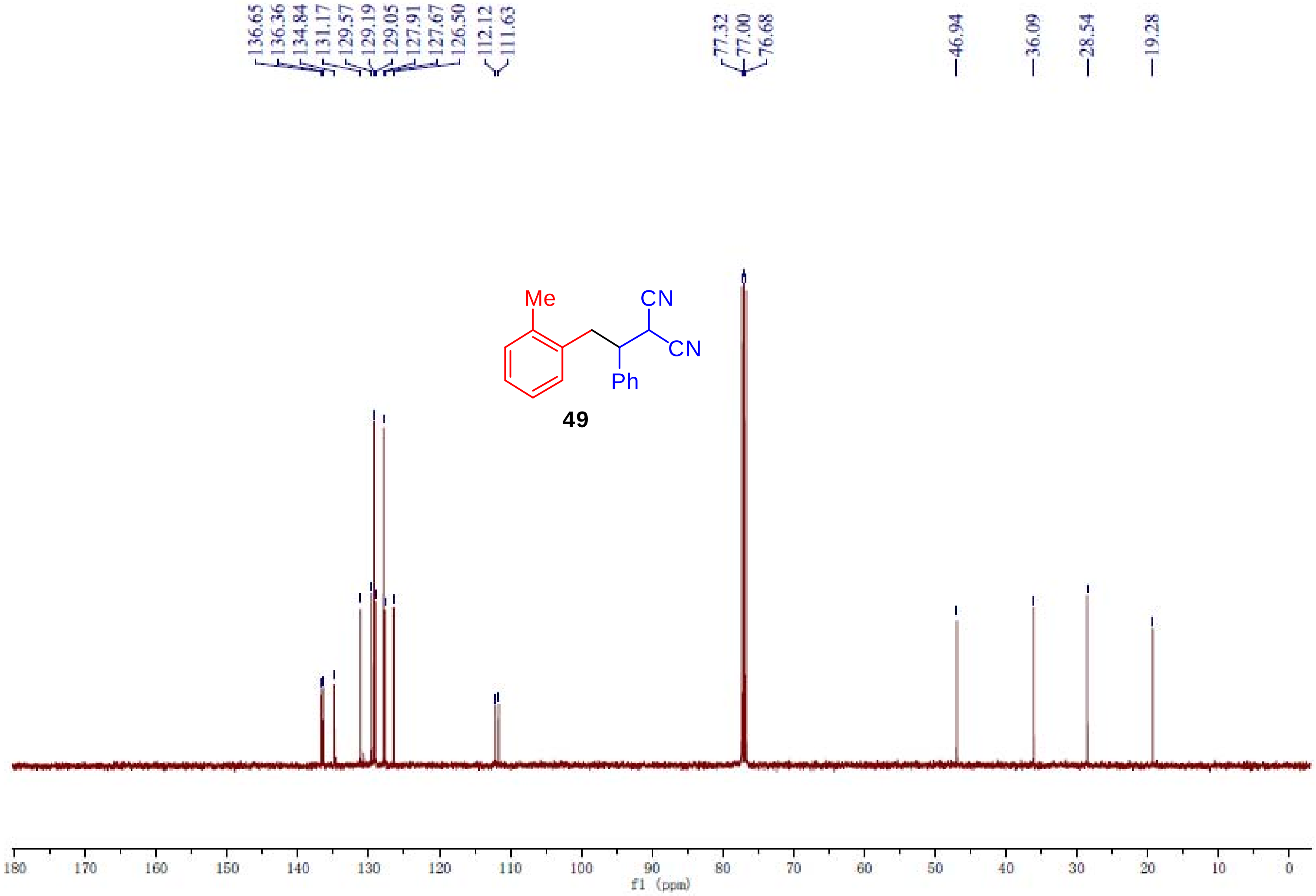
136.65
136.36
134.84
131.17
129.57
129.19
129.05
127.91
127.67
126.50
112.12
111.63
77.32
77.00
76.68
46.94
36.09
28.54
19.28
Me
CN
CN
Ph
49
180 170 160 150 140 130 120 110 100 90 80 70 60 50 40 30 20 10 0
f1 (ppm)

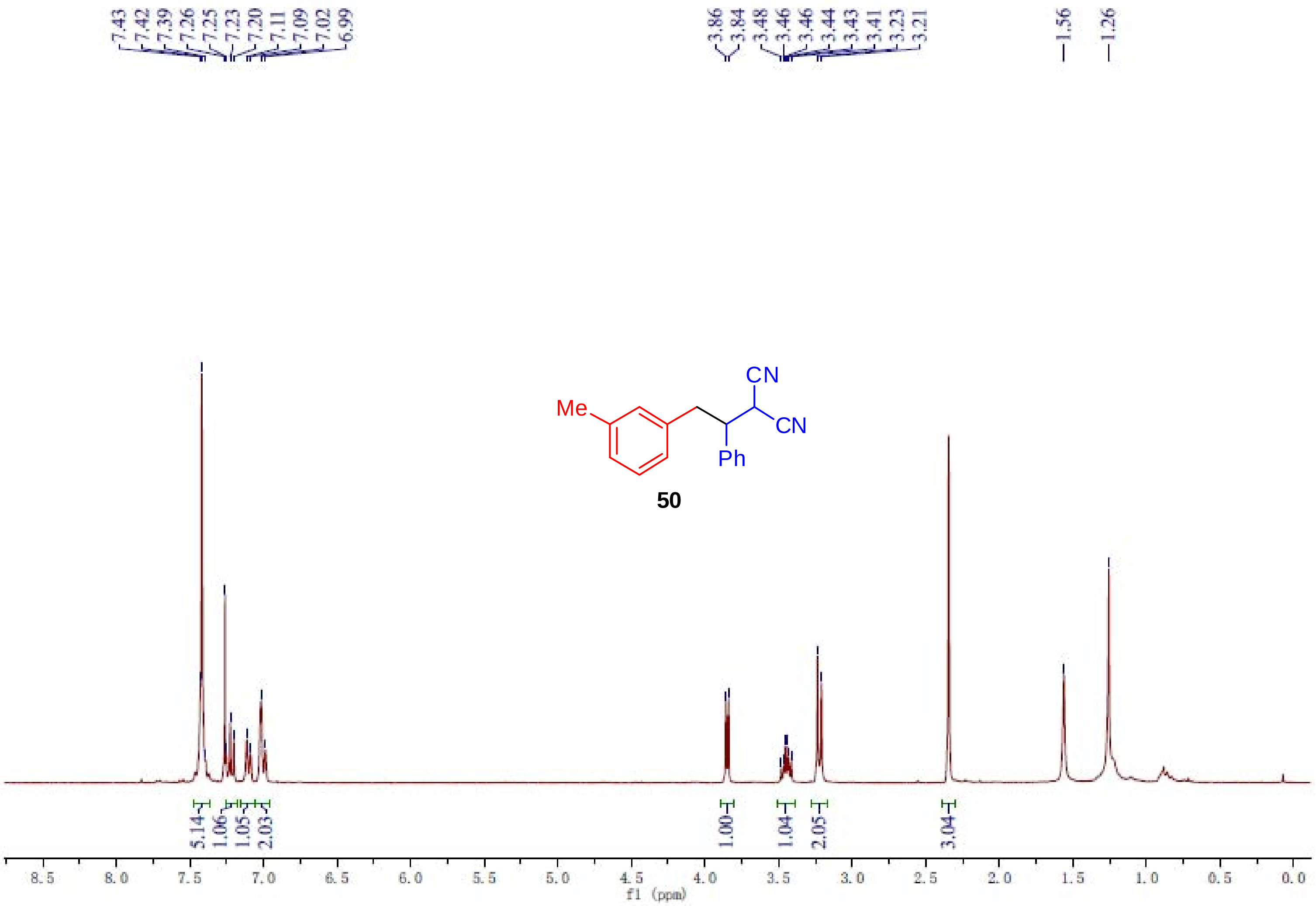
7.43
7.42
7.39
7.26
7.25
7.23
7.20
7.11
7.09
7.02
6.99
3.86
3.84
3.48
3.46
3.46
3.44
3.43
3.41
3.23
3.21
1.56
1.26
Me
CN
CN
Ph
50
5.14
1.06
1.05
2.03
1.00
1.04
2.05
3.04
8.5
8.0
7.5
7.0
6.5
6.0
5.5
5.0
4.5
4.0
3.5
3.0
2.5
2.0
1.5
1.0
0.5
0.0
f1 (ppm)

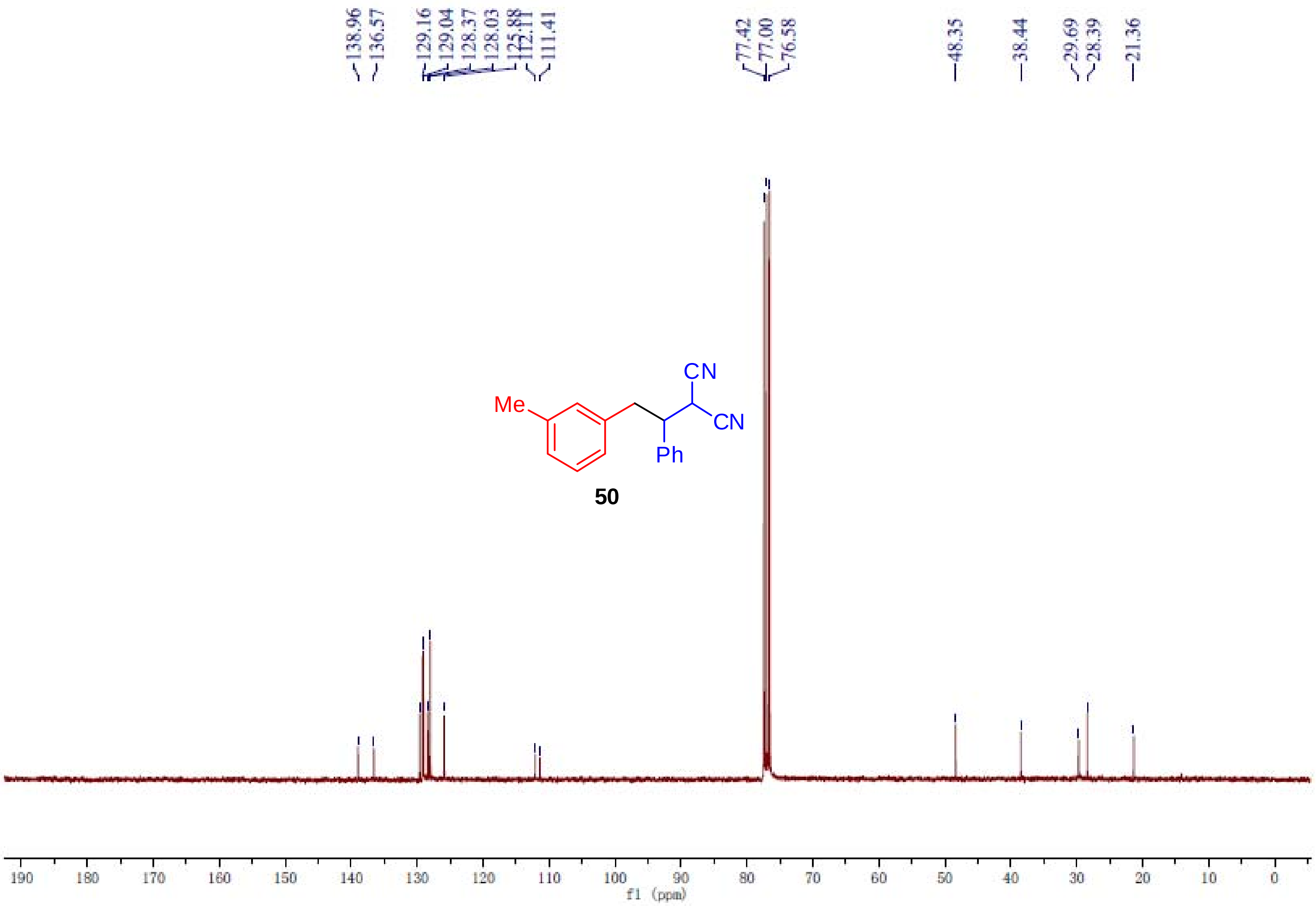
138.96
136.57
129.16
129.04
128.37
128.03
125.88
112.11
111.41
77.42
77.00
76.58
48.35
38.44
29.69
28.39
21.36
Me
CN
CN
Ph
50
190 180 170 160 150 140 130 120 110 100 90 80 70 60 50 40 30 20 10 0
f1 (ppm)

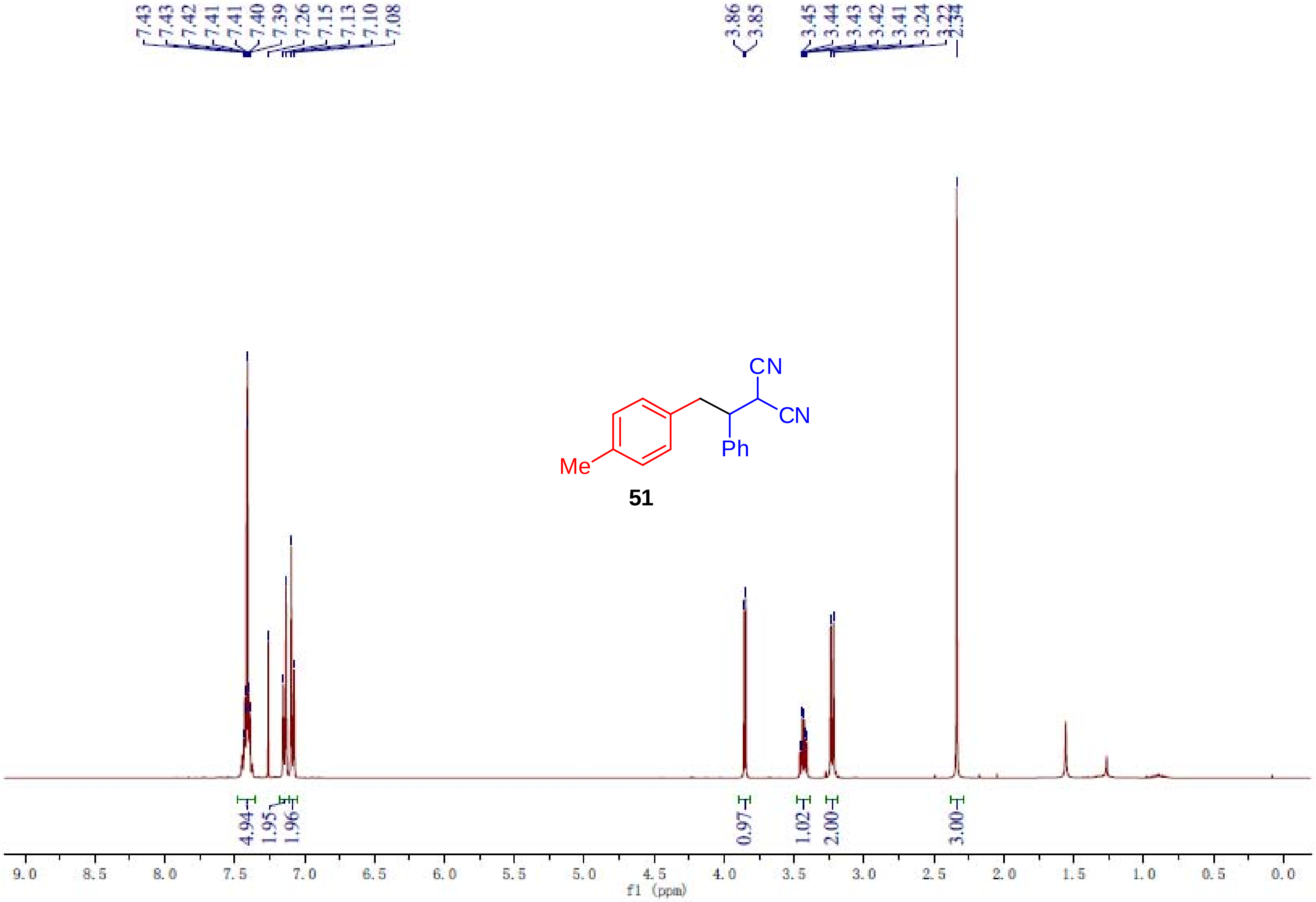
7.43
7.43
7.42
7.41
7.41
7.40
7.39
7.26
7.15
7.13
7.10
7.08
3.86
3.85
3.45
3.44
3.43
3.42
3.41
3.24
3.22
2.34
CN
CN
Ph
Me
51
4.94
1.95
1.96
0.97
1.02
2.00
3.00
9.0
8.5
8.0
7.5
7.0
6.5
6.0
5.5
5.0
4.5
4.0
3.5
3.0
2.5
2.0
1.5
1.0
0.5
0.0
f1 (ppm)

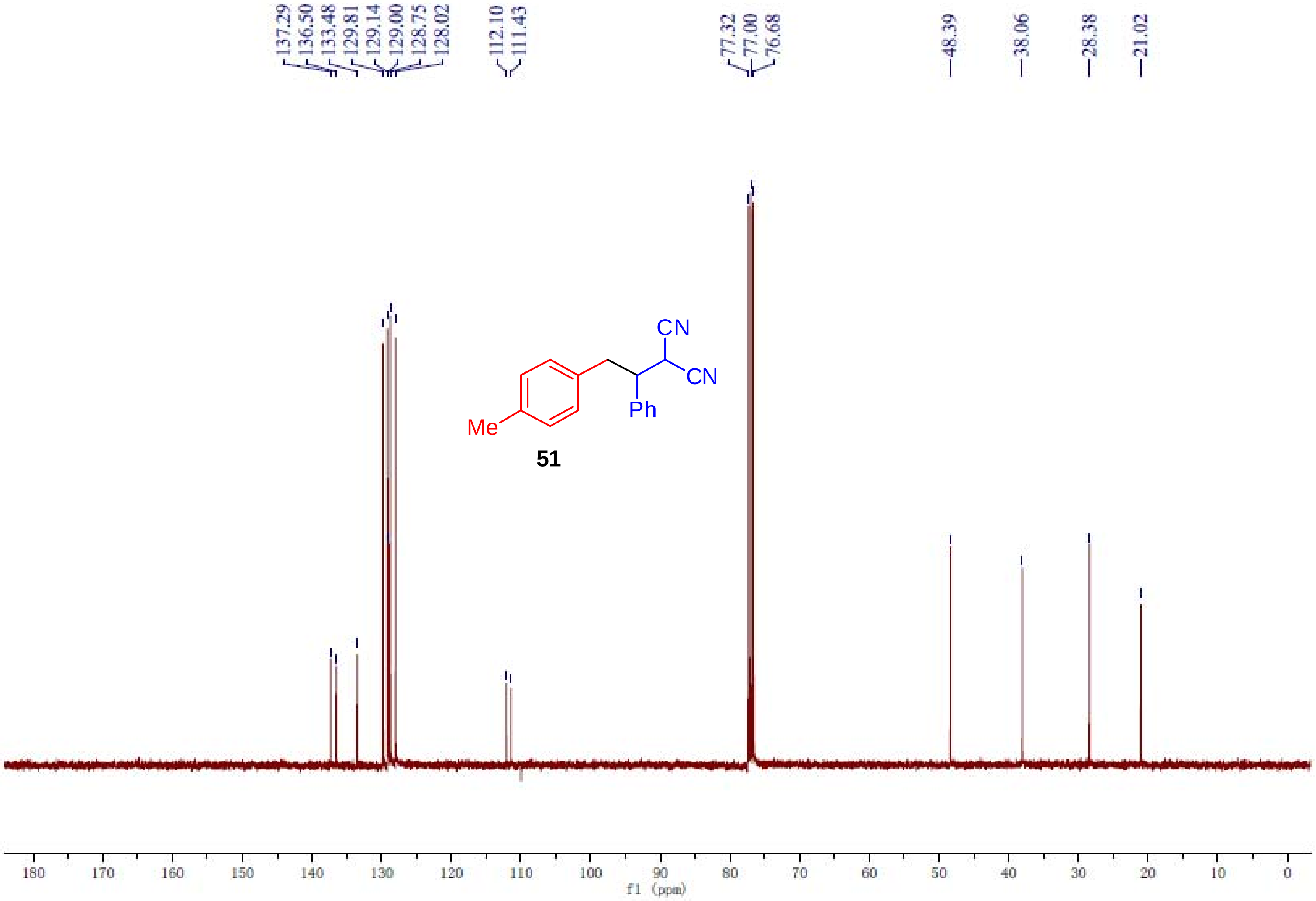

137.29
136.50
133.48
129.81
129.14
129.00
128.75
128.02
112.10
111.43
77.32
77.00
76.68
48.39
38.06
28.38
21.02
CN
CN
Me
Ph
51
180 170 160 150 140 130 120 110 100 90 80 70 60 50 40 30 20 10 0
f1 (ppm)

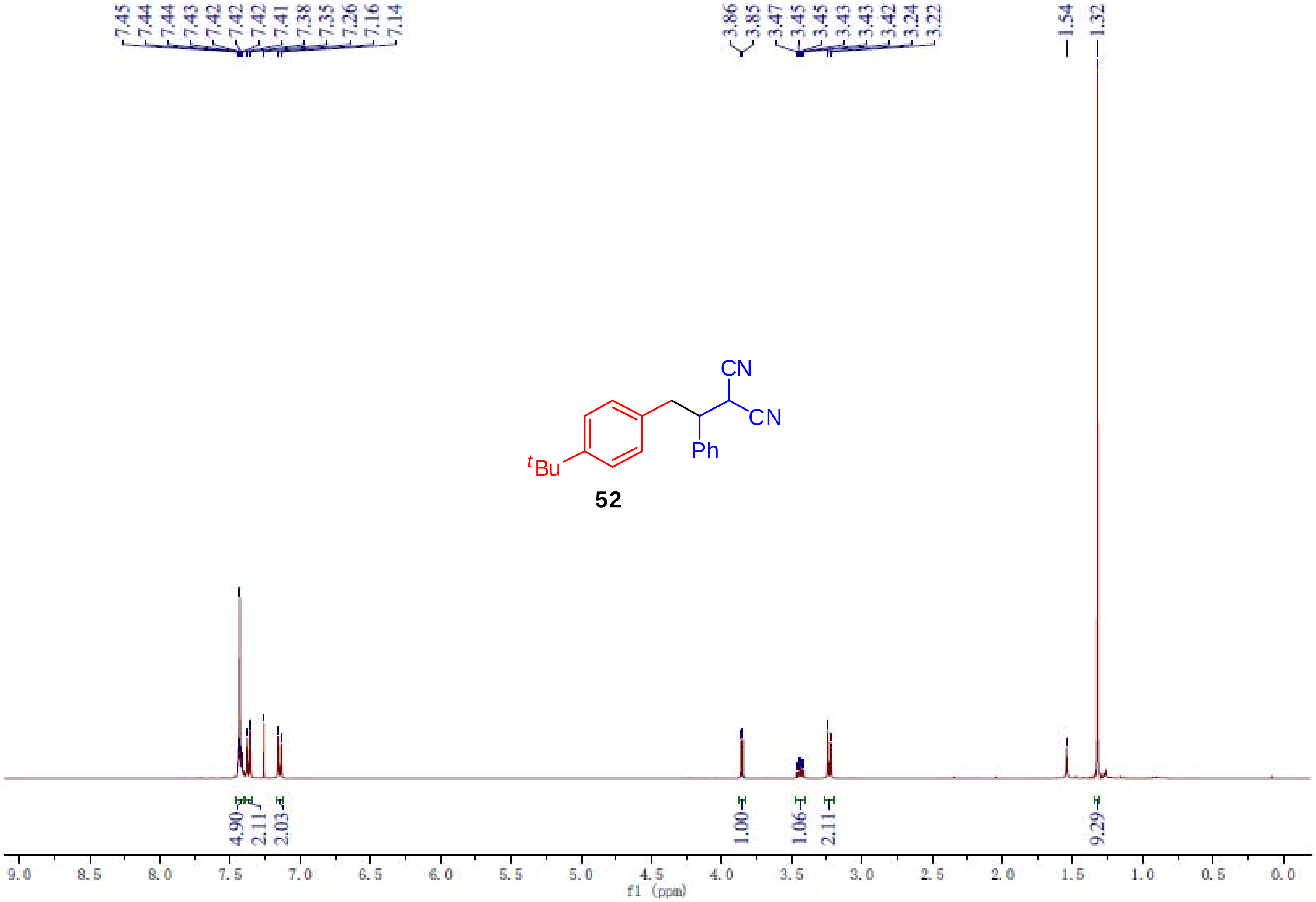
7.45 7.44 7.44 7.43 7.42 7.42 7.42 7.41 7.38 7.35 7.26 7.16 7.14
3.86 3.85 3.47 3.45 3.45 3.43 3.43 3.42 3.24 3.22
1.54
1.32
CN
CN
Ph
^{t}Bu
52
4.90
2.11
2.03
1.00
1.06
2.11
9.29
9.0 8.5 8.0 7.5 7.0 6.5 6.0 5.5 5.0 4.5 4.0 3.5 3.0 2.5 2.0 1.5 1.0 0.5 0.0
f1 (ppm)

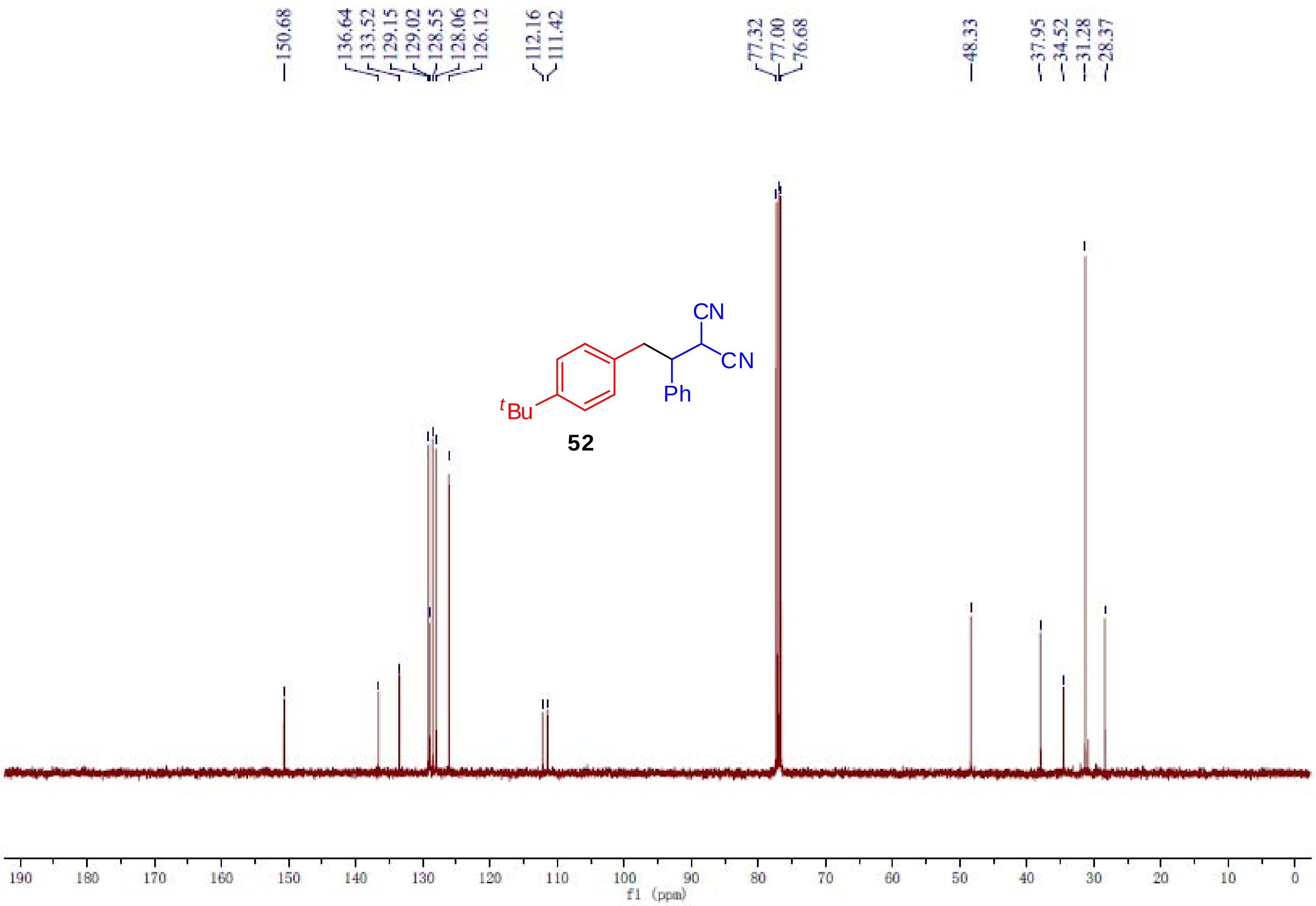
150.68
136.64
133.52
129.15
129.02
128.55
128.06
126.12
112.16
111.42
77.32
77.00
76.68
48.33
37.95
34.52
31.28
28.37
CN
CN
Ph
tBu
52
190 180 170 160 150 140 130 120 110 100 90 80 70 60 50 40 30 20 10 0
f1 (ppm)

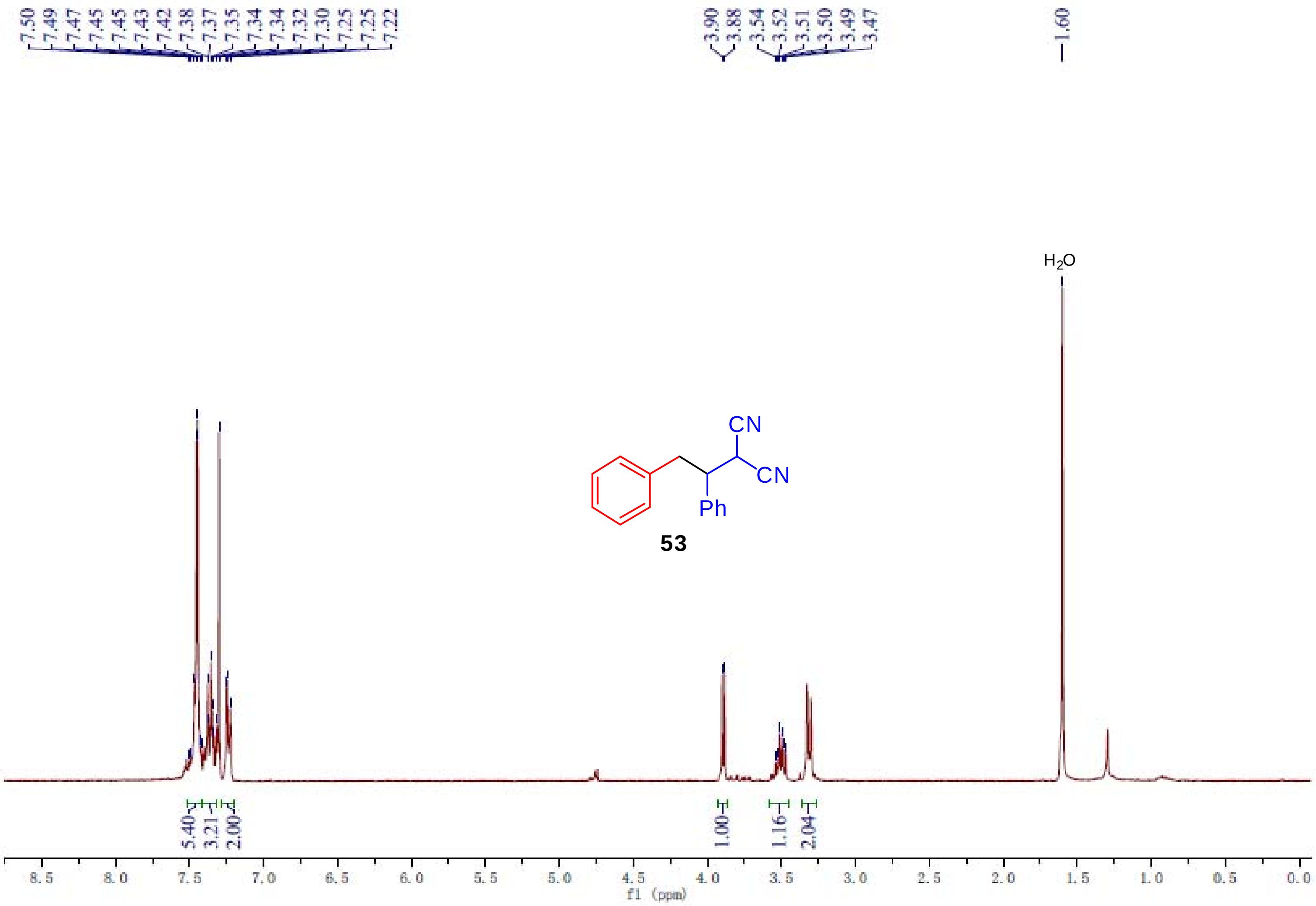
7.50
7.49
7.47
7.45
7.45
7.43
7.42
7.38
7.37
7.35
7.34
7.34
7.32
7.30
7.25
7.25
7.22
3.90
3.88
3.54
3.52
3.51
3.50
3.49
3.47
1.60
H2O
CN
CN
Ph
53
5.40
3.21
2.00
1.00
1.16
2.04
8.5
8.0
7.5
7.0
6.5
6.0
5.5
5.0
4.5
4.0
3.5
3.0
2.5
2.0
1.5
1.0
0.5
0.0
f1 (ppm)

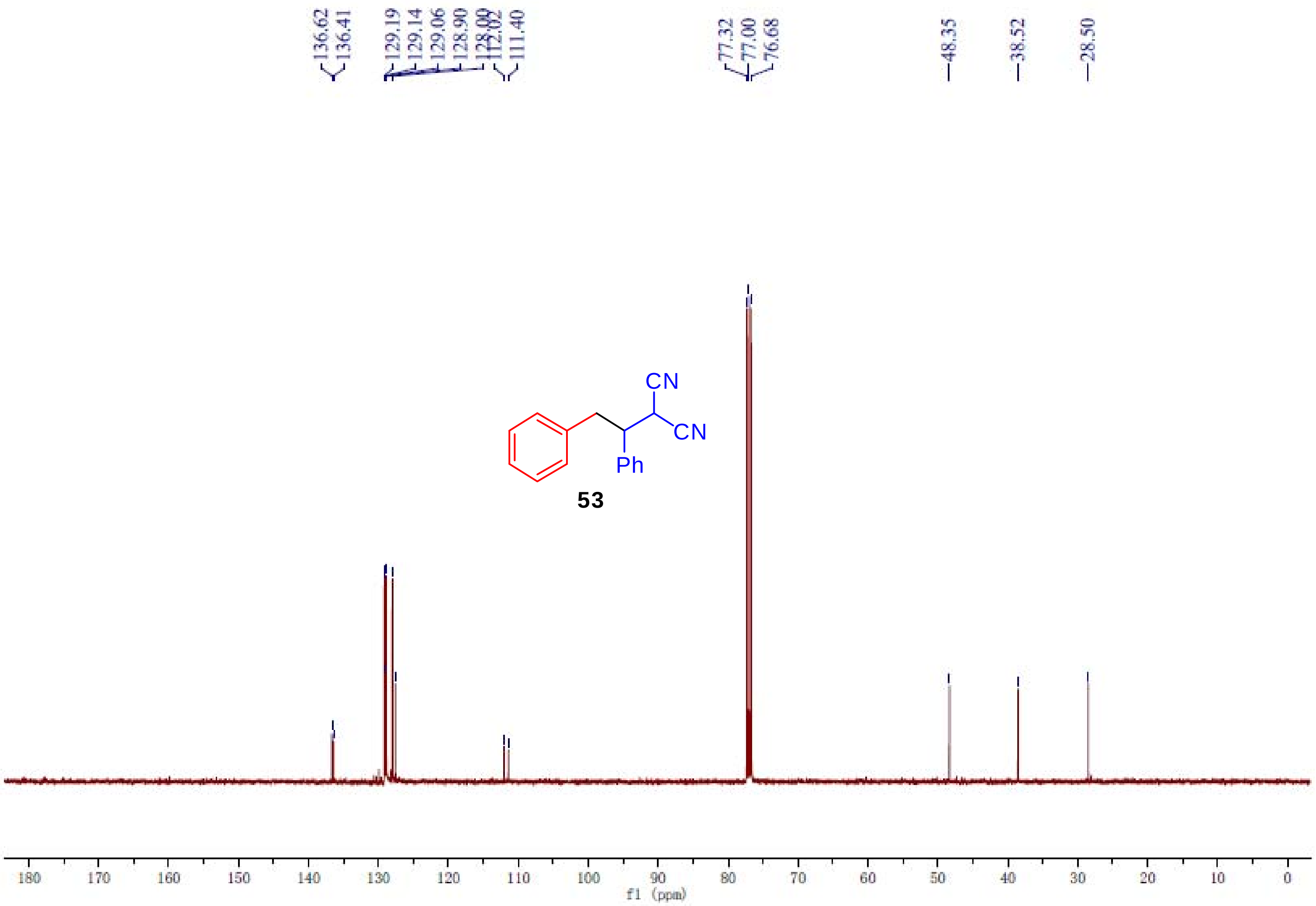
136.62
136.41
129.19
129.14
129.06
128.90
128.00
112.02
111.40
77.32
77.00
76.68
48.35
38.52
28.50
CN
CN
Ph
53
180 170 160 150 140 130 120 110 100 90 80 70 60 50 40 30 20 10 0
f1 (ppm)

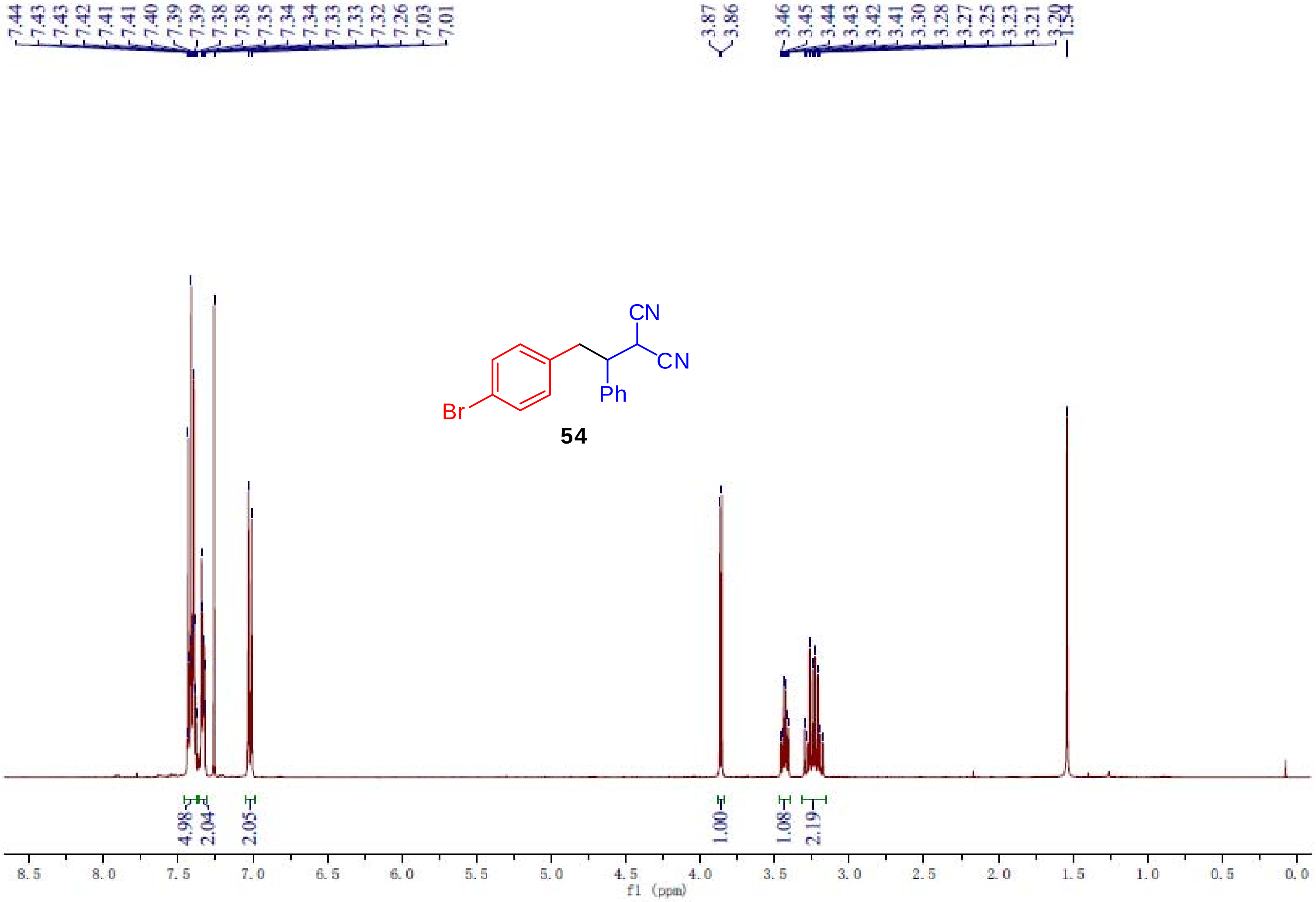
7.44
7.43
7.43
7.42
7.41
7.41
7.40
7.39
7.39
7.38
7.38
7.35
7.34
7.34
7.33
7.33
7.32
7.26
7.03
7.01
3.87
3.86
3.46
3.45
3.44
3.43
3.42
3.41
3.30
3.28
3.27
3.25
3.23
3.21
3.20
1.54
CN
CN
Ph
Br
54
4.98
2.04
2.05
1.00
1.08
2.19
8.5
8.0
7.5
7.0
6.5
6.0
5.5
5.0
4.5
4.0
3.5
3.0
2.5
2.0
1.5
1.0
0.5
0.0
f1 (ppm)

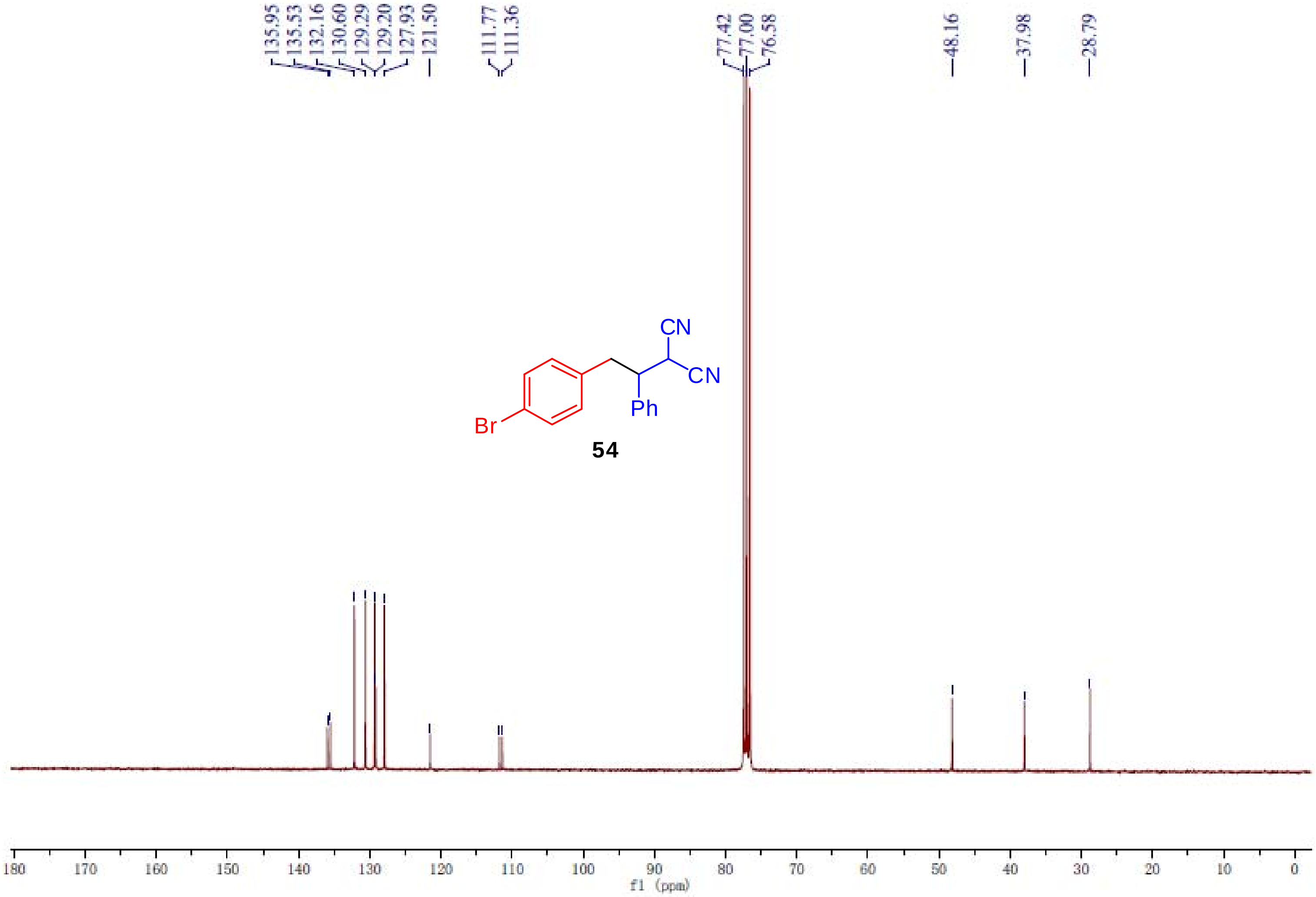
135.95
135.53
132.16
130.60
129.29
129.20
127.93
121.50
111.77
111.36
77.42
77.00
76.58
48.16
37.98
28.79
CN
CN
Br
Ph
54
180 170 160 150 140 130 120 110 100 90 80 70 60 50 40 30 20 10 0
f1 (ppm)

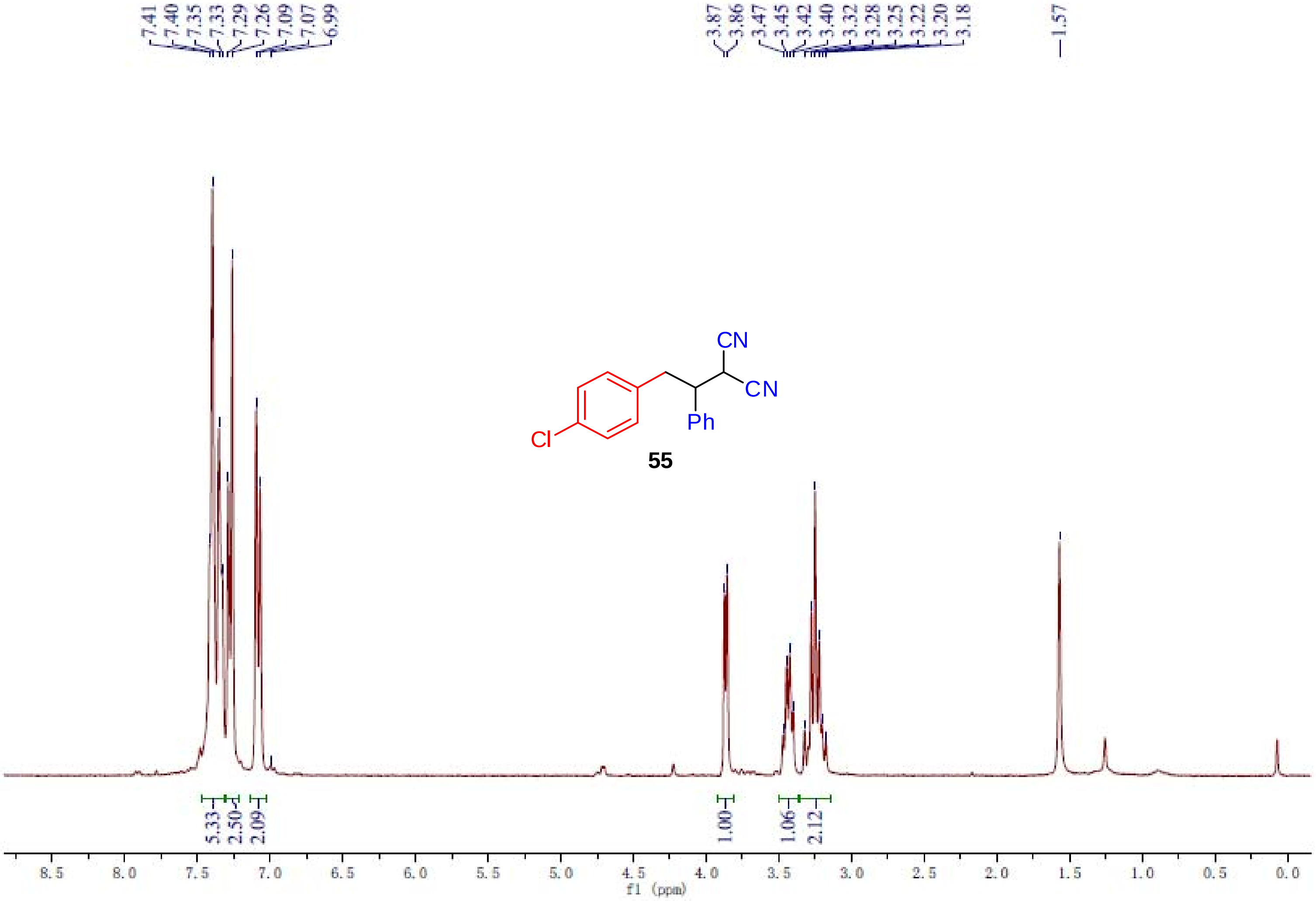
7.41
7.40
7.35
7.33
7.29
7.26
7.09
7.07
6.99
3.87
3.86
3.47
3.45
3.42
3.40
3.32
3.28
3.25
3.22
3.20
3.18
1.57
CN
CN
Cl
Ph
55
5.33
2.50
2.09
1.00
1.06
2.12
8.5
8.0
7.5
7.0
6.5
6.0
5.5
5.0
4.5
4.0
3.5
3.0
2.5
2.0
1.5
1.0
0.5
0.0
f1 (ppm)

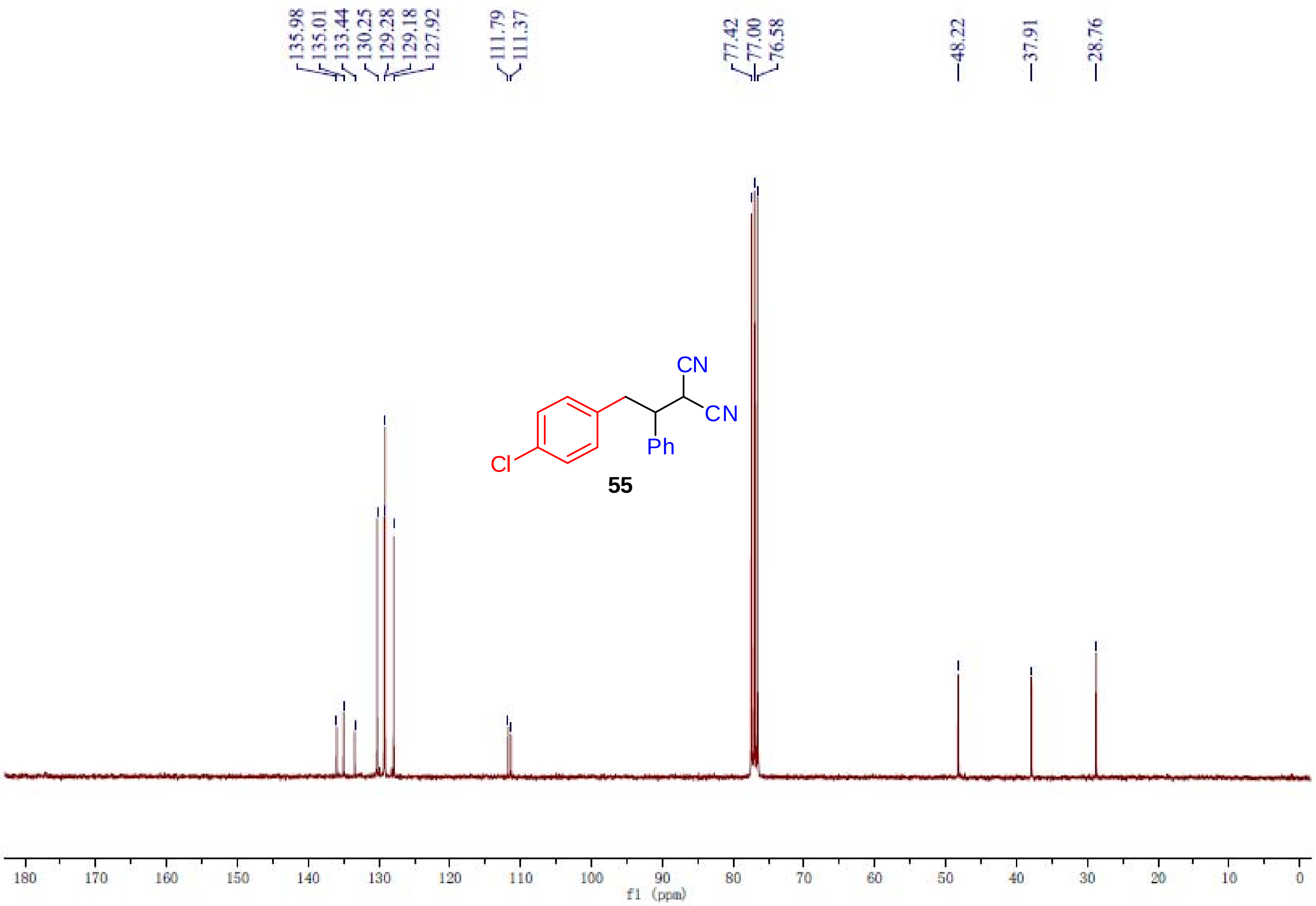
135.98
135.01
133.44
130.25
129.28
129.18
127.92
111.79
111.37
77.42
77.00
76.58
48.22
37.91
28.76
CN
CN
Cl
Ph
55
180
170
160
150
140
130
120
110
100
90
80
70
60
50
40
30
20
10
0
f1 (ppm)

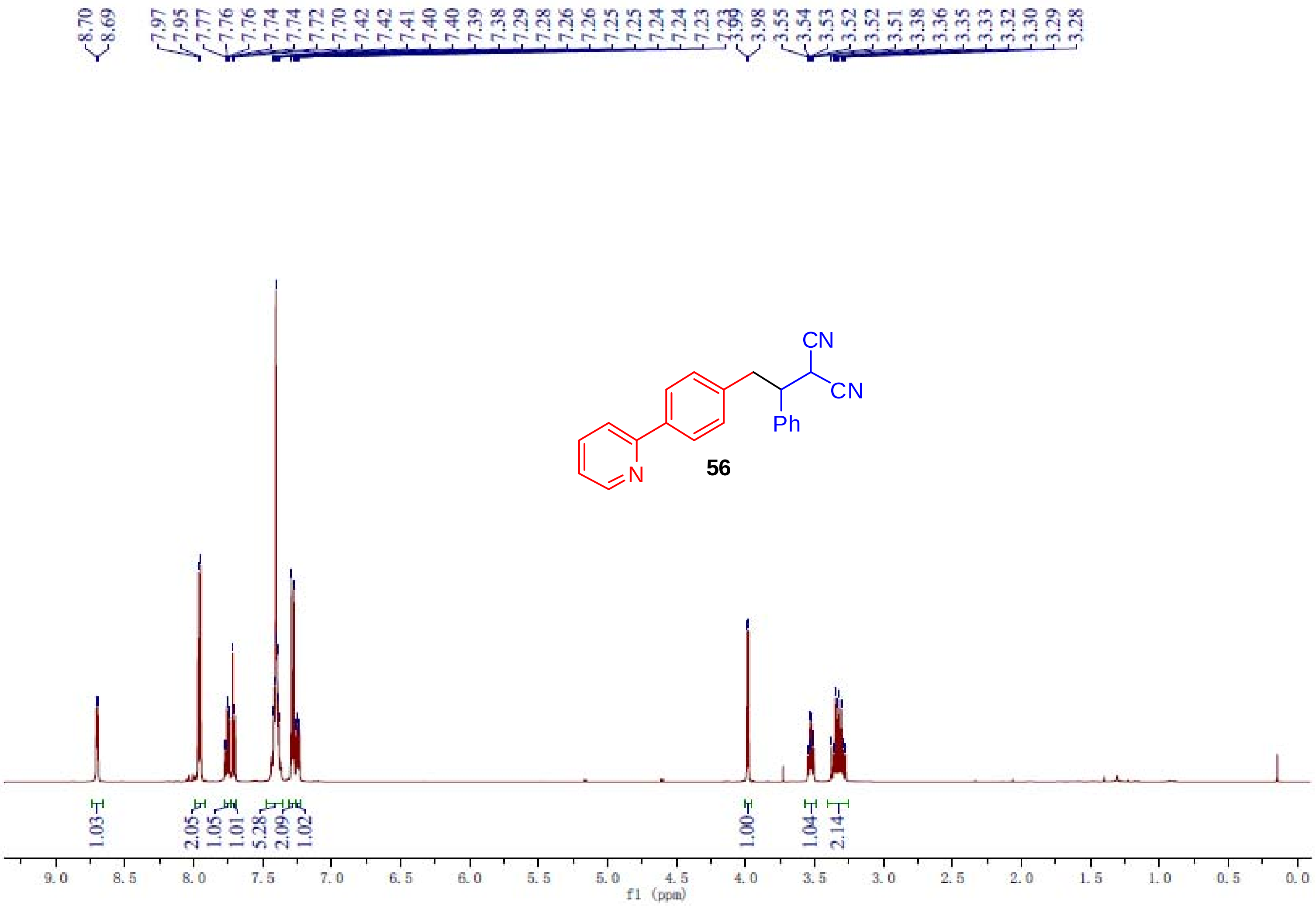

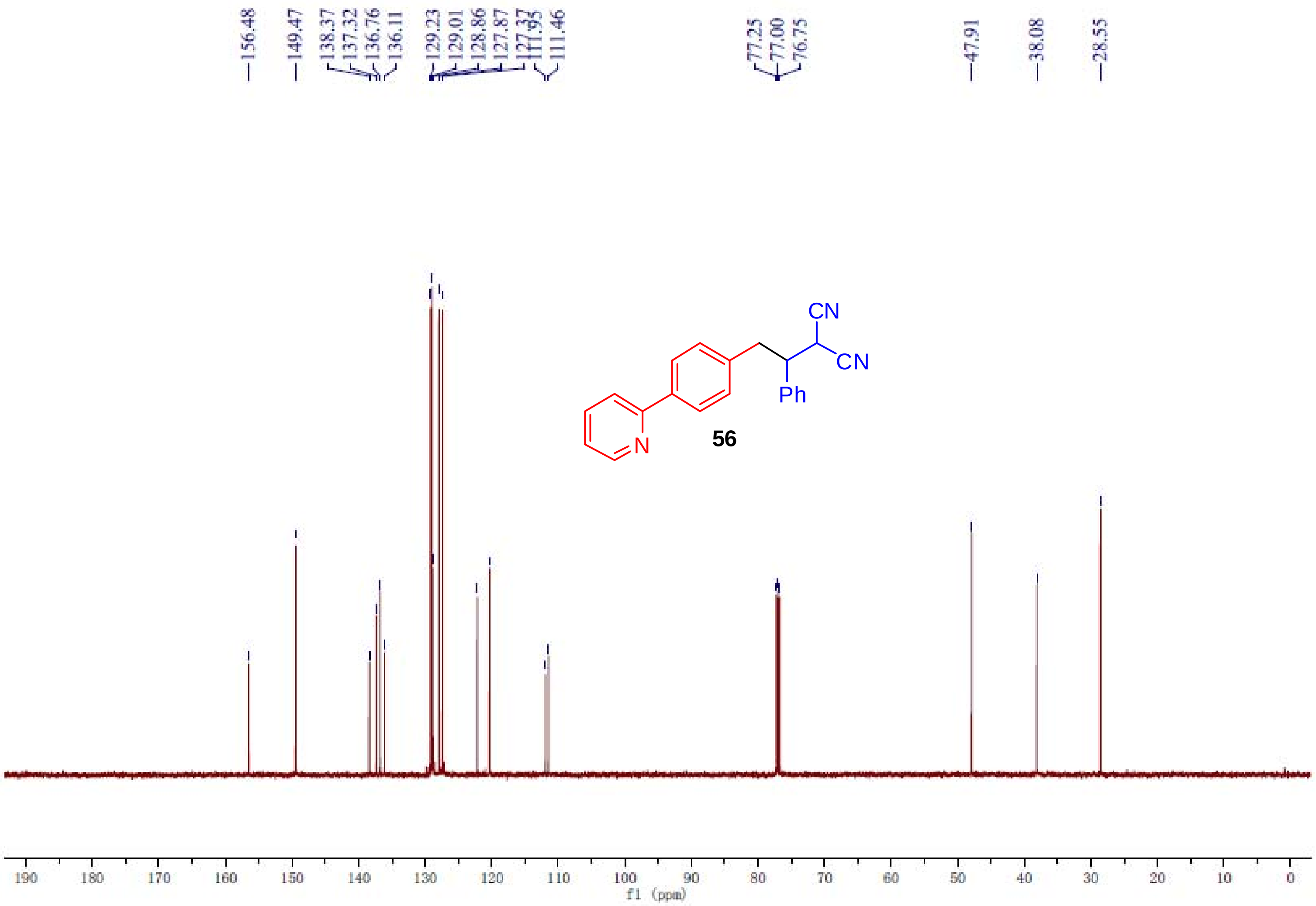

156.48
149.47
138.37
137.32
136.76
136.11
129.23
129.01
128.86
127.87
127.37
111.95
111.46
77.25
77.00
76.75
47.91
38.08
28.55
CN
CN
Ph
N
56
190
180
170
160
150
140
130
120
110
100
90
80
70
60
50
40
30
20
10
0
f1 (ppm)

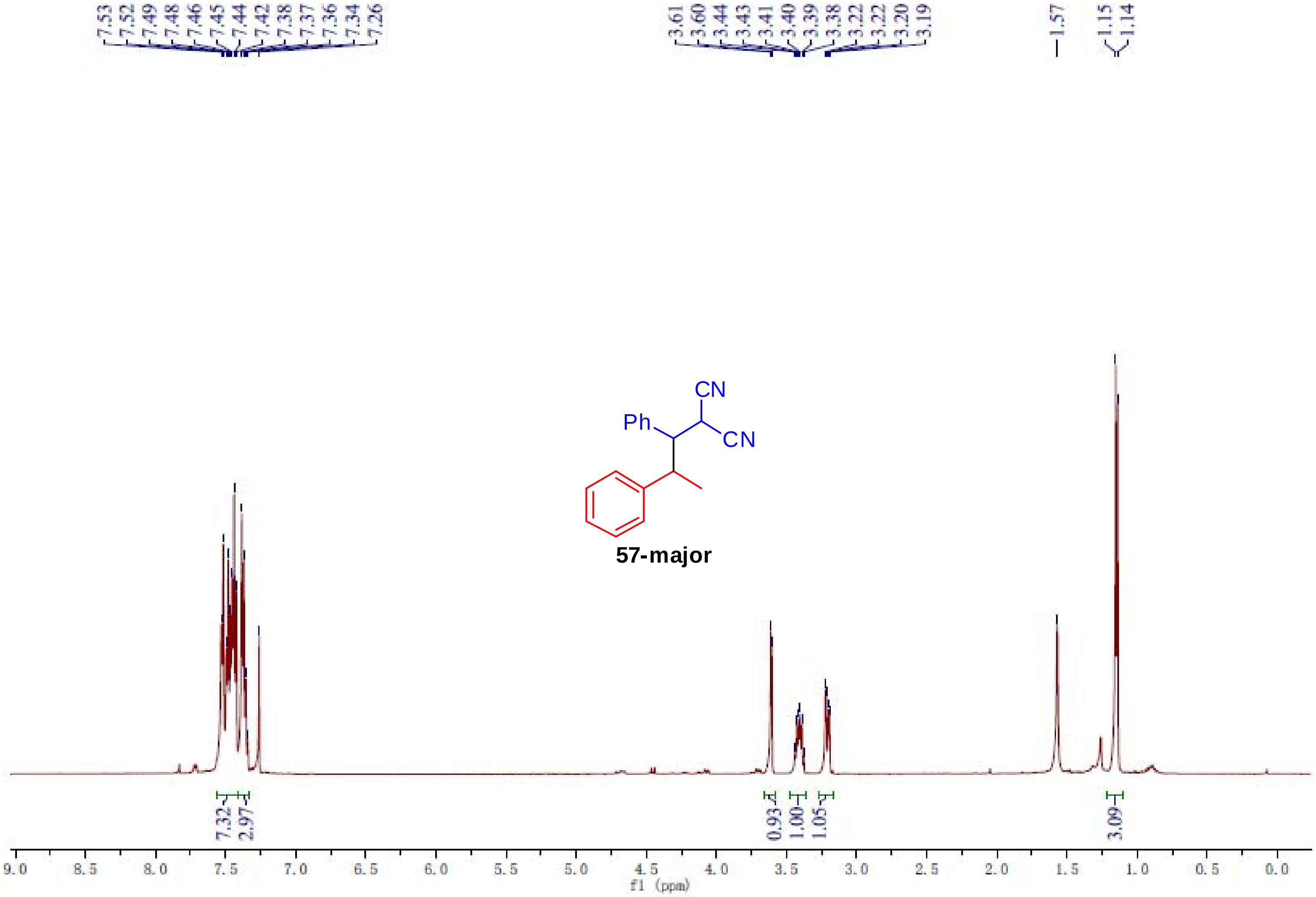
7.53
7.52
7.49
7.48
7.46
7.45
7.44
7.42
7.38
7.37
7.36
7.34
7.26
3.61
3.60
3.44
3.43
3.41
3.40
3.39
3.38
3.22
3.22
3.20
3.19
1.57
1.15
1.14
Ph
CN
CN
57-major
7.32
2.97
0.93
1.00
1.05
3.09
9.0
8.5
8.0
7.5
7.0
6.5
6.0
5.5
5.0
4.5
4.0
3.5
3.0
2.5
2.0
1.5
1.0
0.5
0.0
f1 (ppm)

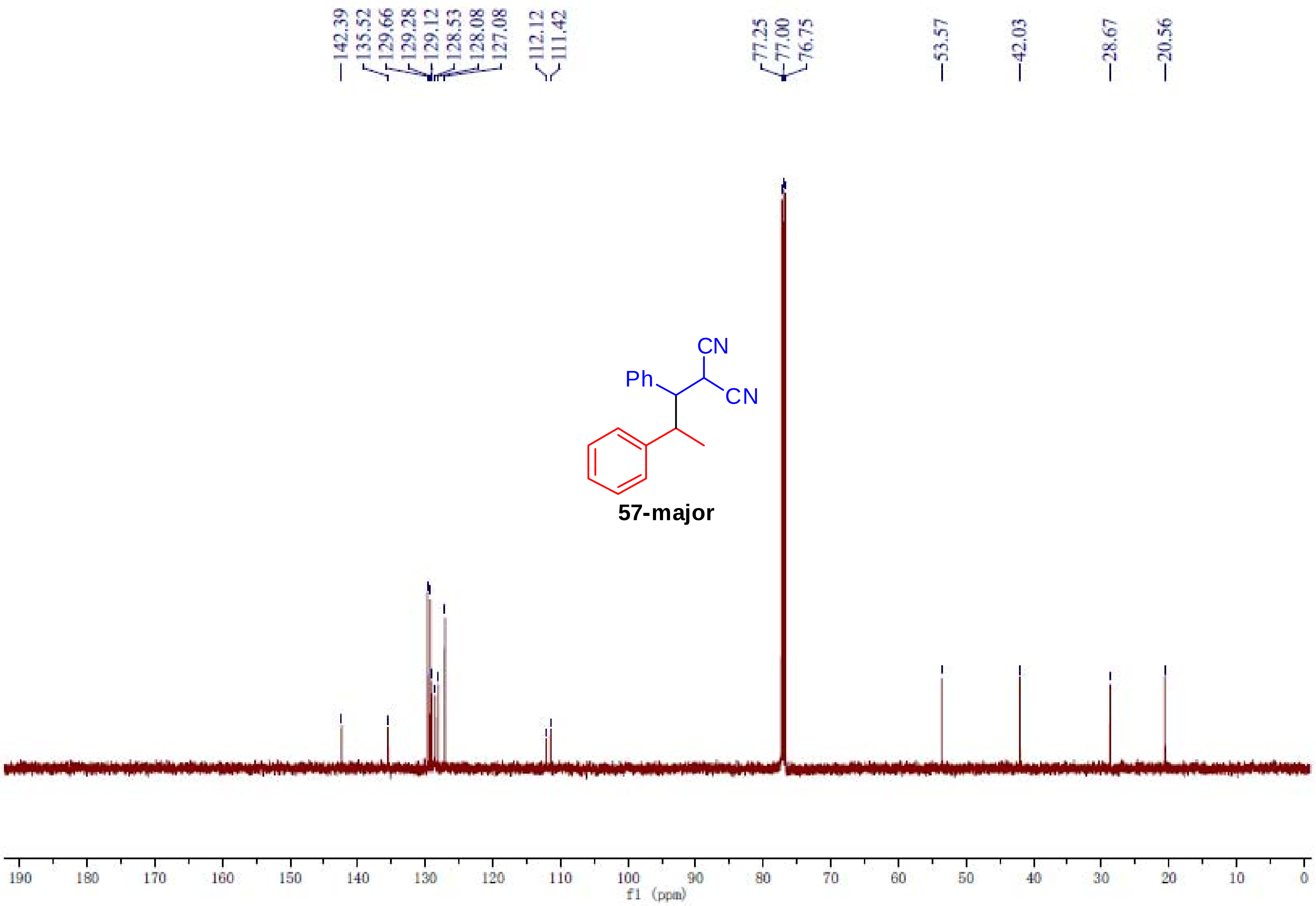

142.39
135.52
129.66
129.28
129.12
128.53
128.08
127.08
112.12
111.42
77.25
77.00
76.75
53.57
42.03
28.67
20.56
CN
Ph
CN
57-major
190
180
170
160
150
140
130
120
110
100
90
80
70
60
50
40
30
20
10
0
f1 (ppm)

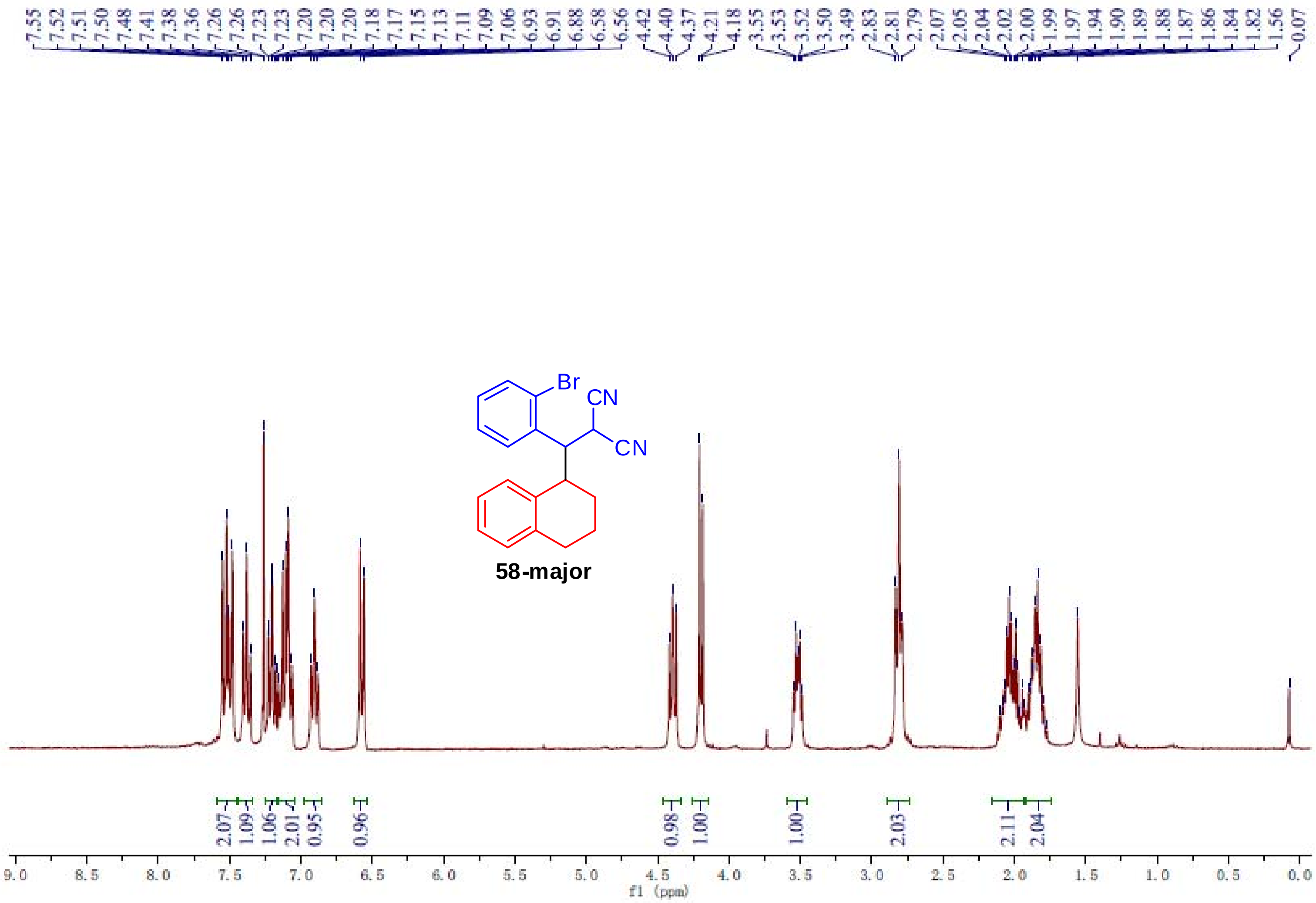
7.55 7.52 7.51 7.50 7.48 7.41 7.38 7.36 7.26 7.26 7.23 7.23 7.20 7.20 7.20 7.18 7.17 7.15 7.13 7.11 7.09 7.06 6.93 6.91 6.88 6.58 6.56 4.42 4.40 4.37 4.21 4.18 3.55 3.53 3.52 3.50 3.49 2.83 2.81 2.79 2.07 2.05 2.04 2.02 2.00 1.99 1.97 1.94 1.90 1.89 1.88 1.87 1.86 1.84 1.82 1.56 0.07
Br
CN
CN
58-major
2.07 1.09 1.06 2.01 0.95 0.96 0.98 1.00 1.00 2.03 2.11 2.04
f1 (ppm)

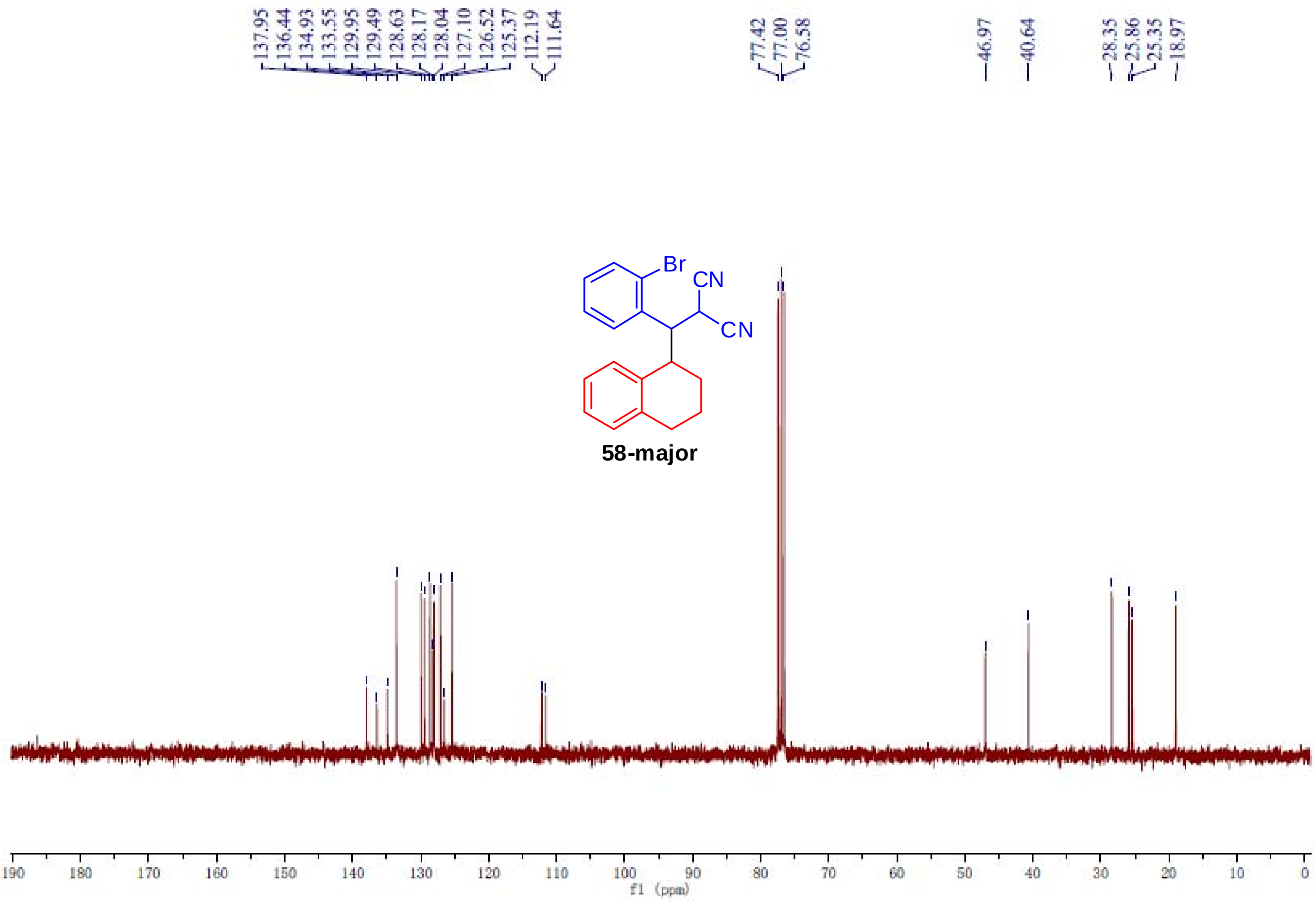
137.95
136.44
134.93
133.55
129.95
129.49
128.63
128.17
128.04
127.10
126.52
125.37
112.19
111.64
77.42
77.00
76.58
46.97
40.64
28.35
25.86
25.35
18.97
Br
CN
CN
58-major
190
180
170
160
150
140
130
120
110
100
90
80
70
60
50
40
30
20
10
0
f1 (ppm)

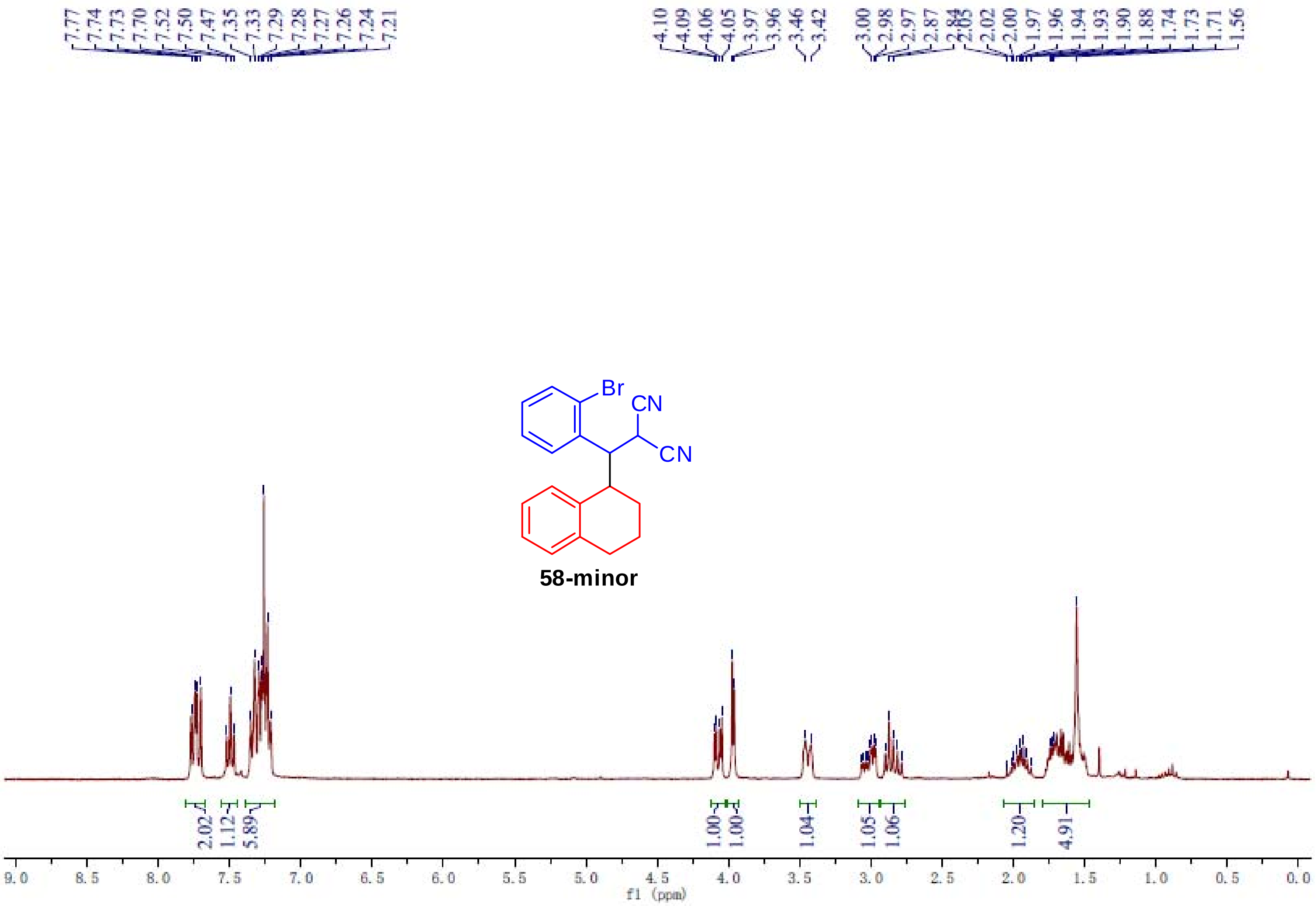

7.77
7.74
7.73
7.70
7.52
7.50
7.47
7.35
7.33
7.29
7.28
7.27
7.26
7.24
7.21
4.10
4.09
4.06
4.05
3.97
3.96
3.46
3.42
3.00
2.98
2.97
2.87
2.84
2.05
2.02
2.00
1.97
1.96
1.94
1.93
1.90
1.88
1.74
1.73
1.71
1.56
Br
CN
CN
58-minor
2.02
1.12
5.89
1.00
1.00
1.04
1.05
1.06
1.20
4.91
f1 (ppm)

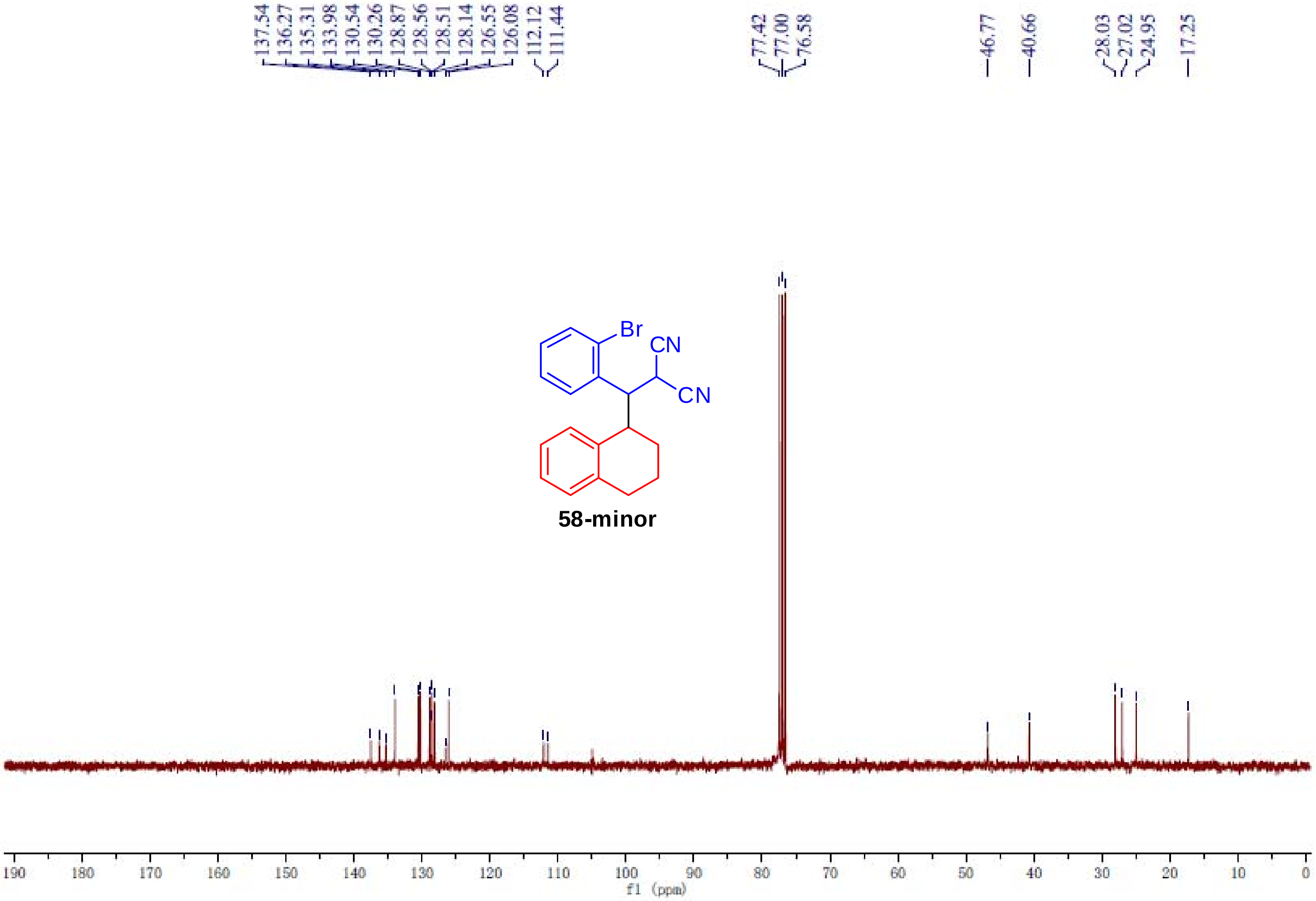
137.54
136.27
135.31
133.98
130.54
130.26
128.87
128.56
128.51
128.14
126.55
126.08
112.12
111.44
77.42
77.00
76.58
46.77
40.66
28.03
27.02
24.95
17.25
Br
CN
CN
58-minor
190 180 170 160 150 140 130 120 110 100 90 80 70 60 50 40 30 20 10 0
f1 (ppm)

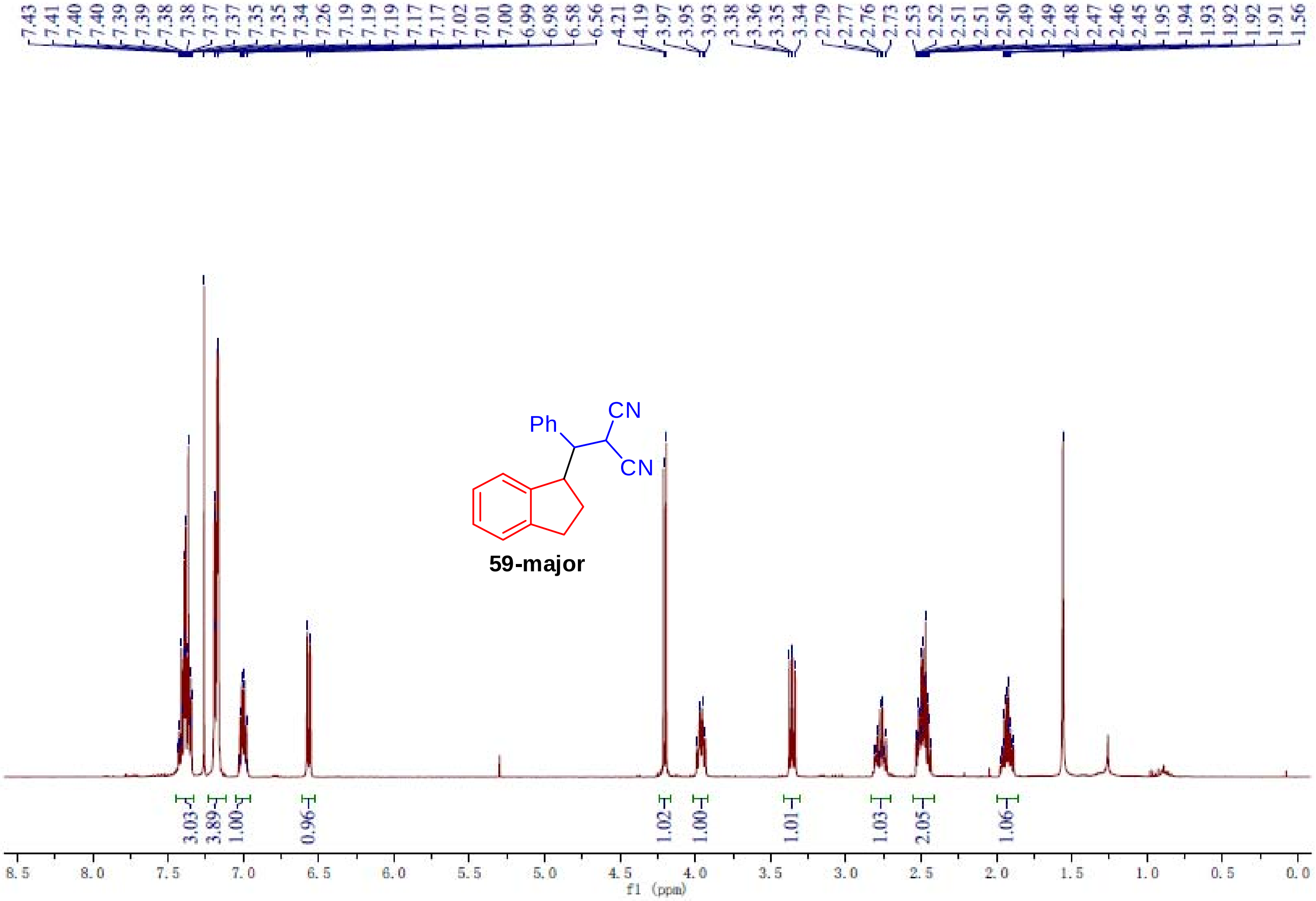
7.43
7.41
7.40
7.40
7.39
7.39
7.38
7.38
7.37
7.37
7.35
7.35
7.34
7.26
7.19
7.19
7.19
7.17
7.17
7.02
7.01
7.00
6.99
6.98
6.58
6.56
4.21
4.19
3.97
3.95
3.93
3.38
3.36
3.35
3.34
2.79
2.77
2.76
2.73
2.53
2.52
2.51
2.51
2.50
2.49
2.49
2.48
2.47
2.46
2.45
1.95
1.94
1.93
1.92
1.92
1.91
1.56
Ph
CN
CN
59-major
3.03
3.89
1.00
0.96
1.02
1.00
1.01
1.03
2.05
1.06
8.5
8.0
7.5
7.0
6.5
6.0
5.5
5.0
4.5
4.0
3.5
3.0
2.5
2.0
1.5
1.0
0.5
0.0
f1 (ppm)

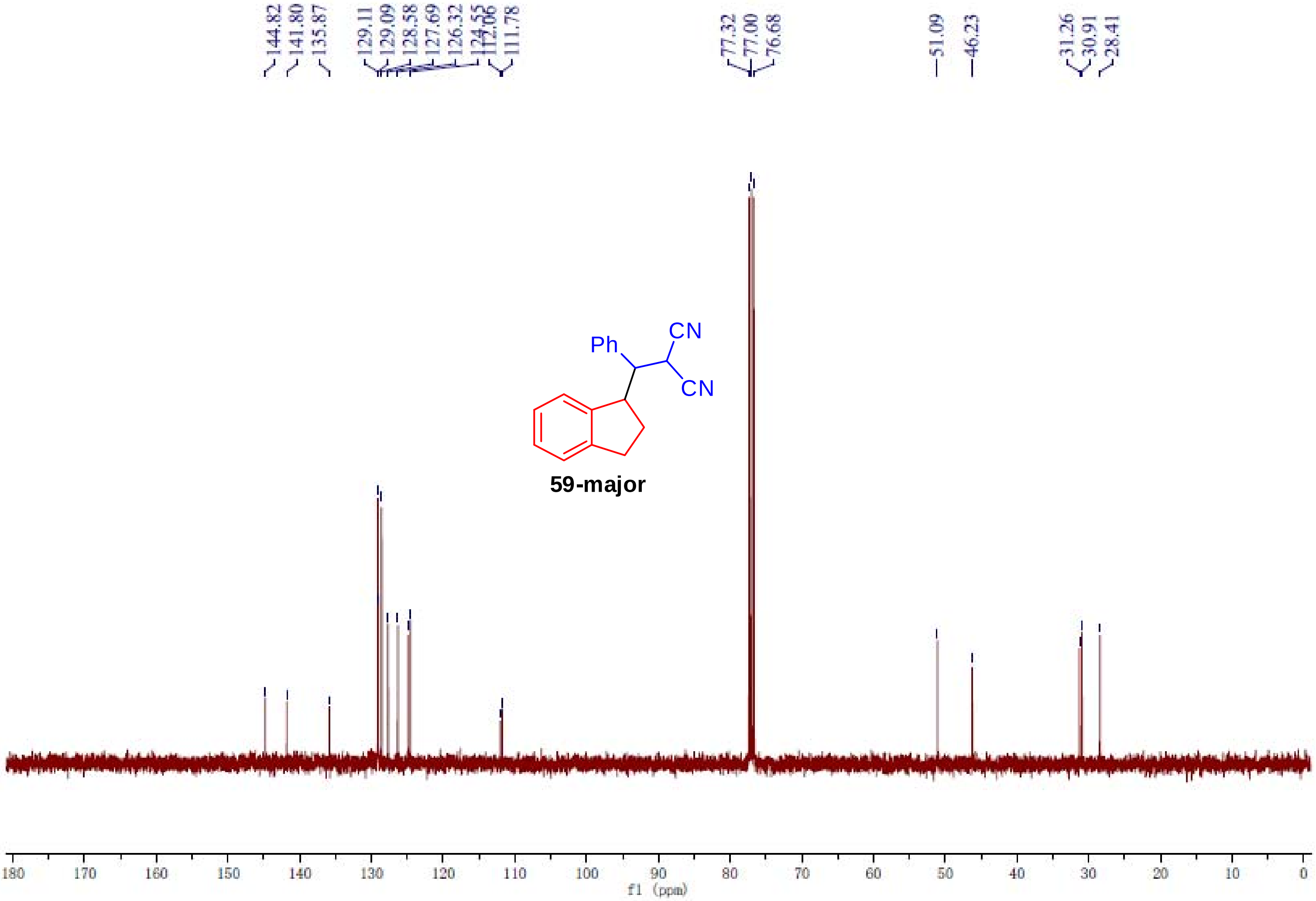
144.82
141.80
135.87
129.11
129.09
128.58
127.69
126.32
124.55
112.06
111.78
77.32
77.00
76.68
51.09
46.23
31.26
30.91
28.41
Ph
CN
CN
59-major
180 170 160 150 140 130 120 110 100 90 80 70 60 50 40 30 20 10 0
f1 (ppm)

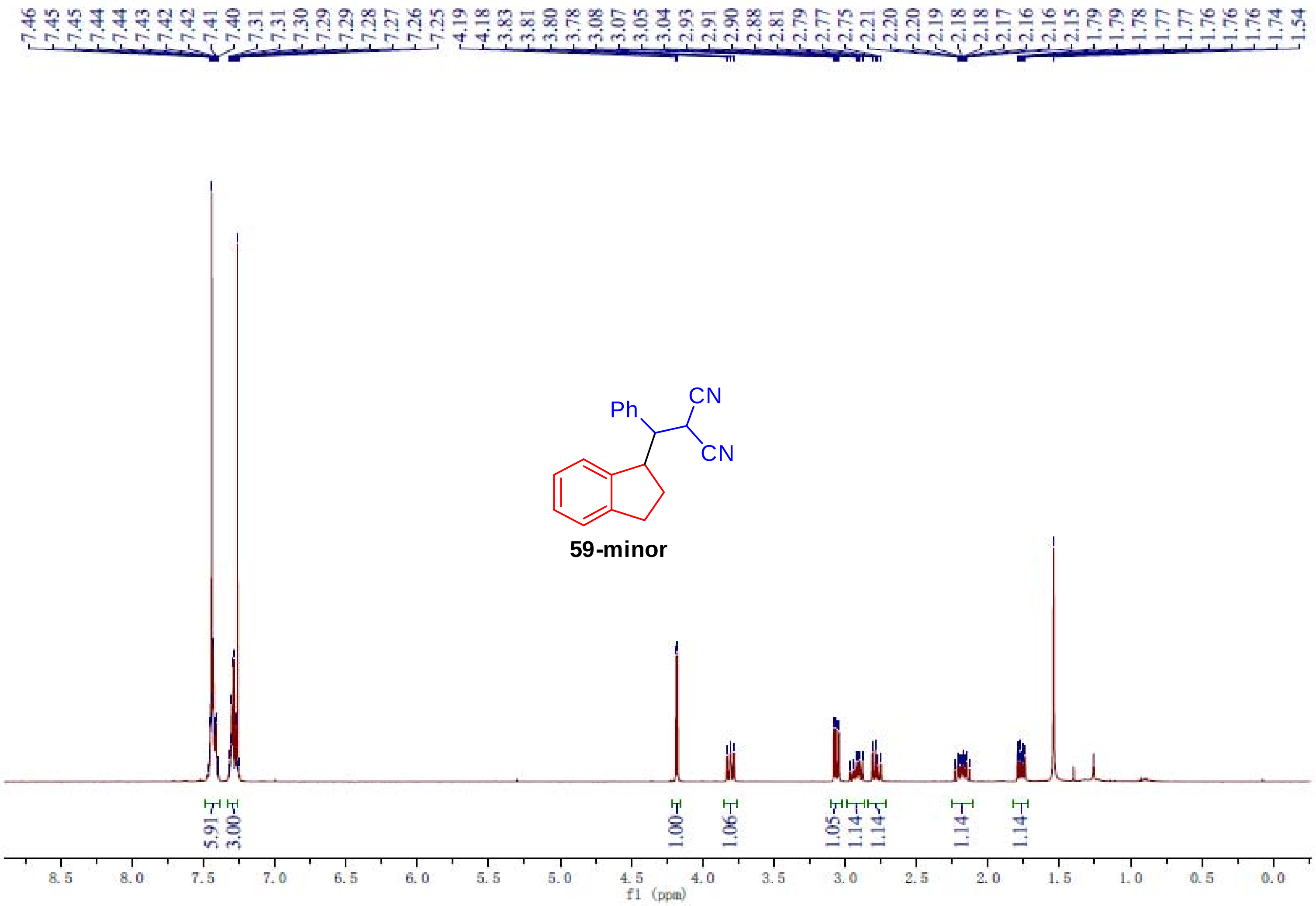

7.46
7.45
7.45
7.44
7.44
7.43
7.42
7.42
7.41
7.40
7.31
7.31
7.30
7.29
7.29
7.28
7.27
7.26
7.25
4.19
4.18
3.83
3.81
3.80
3.78
3.08
3.07
3.05
3.04
2.93
2.91
2.90
2.88
2.81
2.79
2.77
2.75
2.21
2.20
2.20
2.19
2.18
2.18
2.17
2.16
2.16
2.15
1.79
1.79
1.78
1.77
1.77
1.76
1.76
1.76
1.74
1.54
Ph
CN
CN
59-minor
5.91
3.00
1.00
1.06
1.05
1.14
1.14
1.14
1.14
8.5
8.0
7.5
7.0
6.5
6.0
5.5
5.0
4.5
4.0
3.5
3.0
2.5
2.0
1.5
1.0
0.5
0.0
f1 (ppm)

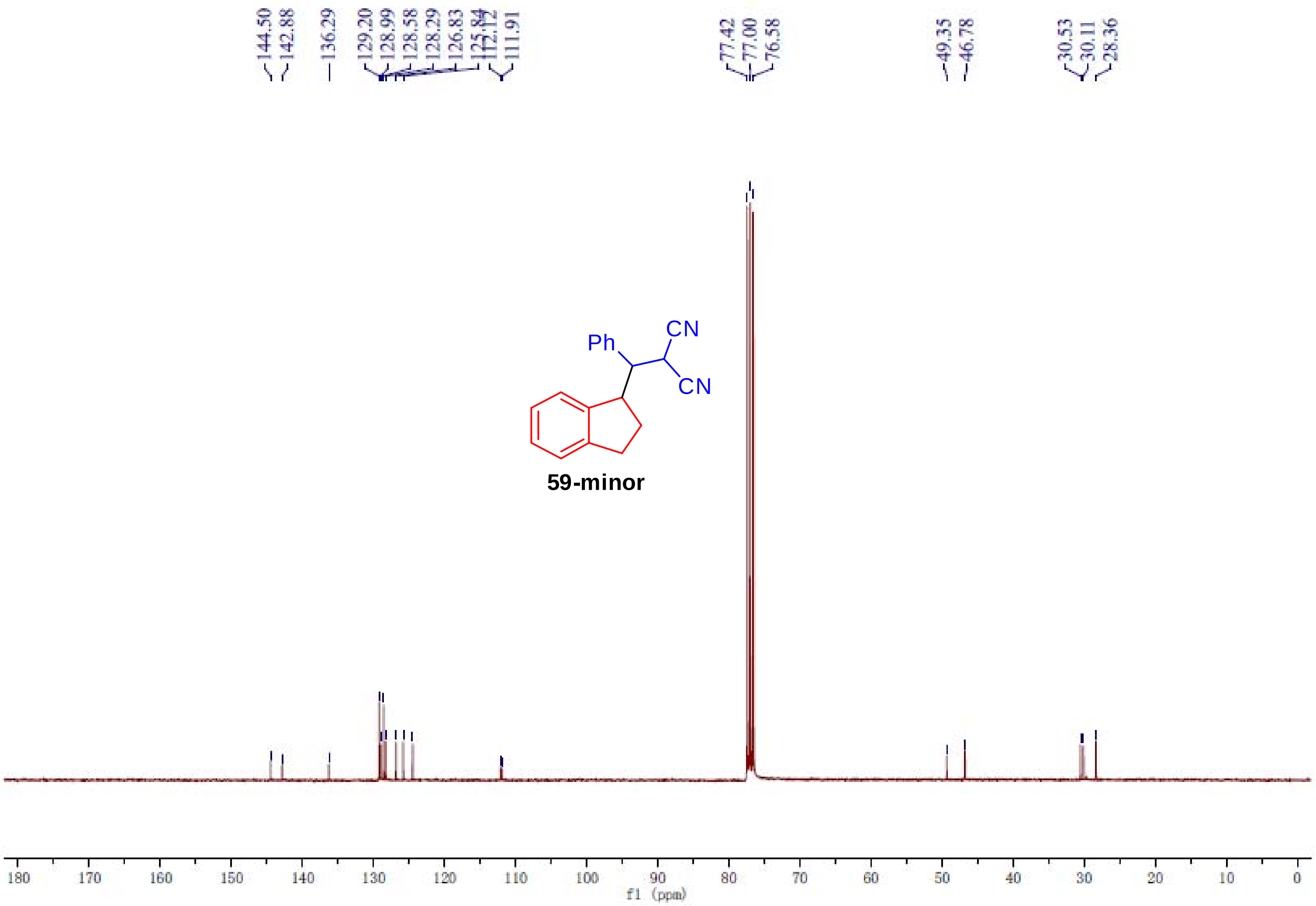
144.50
142.88
136.29
129.20
128.99
128.58
128.29
126.83
125.84
124.12
111.91
77.42
77.00
76.58
49.35
46.78
30.53
30.11
28.36
Ph
CN
CN
59-minor
180 170 160 150 140 130 120 110 100 90 80 70 60 50 40 30 20 10 0
f1 (ppm)

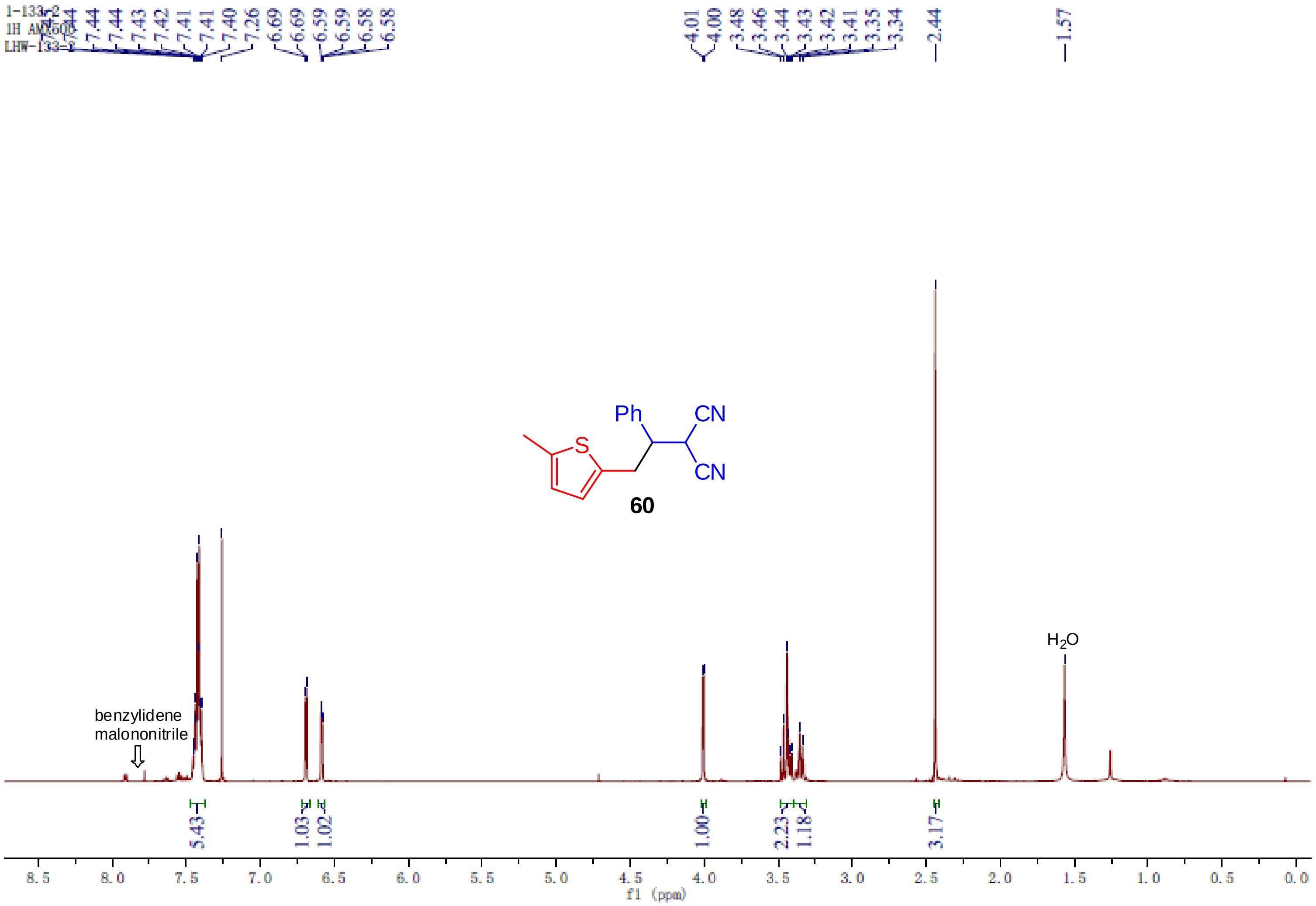
1-133-2
1H AM600
LHW-133-2
7.44
7.44
7.43
7.42
7.41
7.41
7.40
7.26
6.69
6.69
6.59
6.59
6.58
6.58
4.01
4.00
3.48
3.46
3.44
3.43
3.42
3.41
3.35
3.34
2.44
1.57
Ph
CN
S
CN
60
benzylidene
malononitrile
H_2O
5.43
1.03
1.02
1.00
2.23
1.18
3.17
8.5
8.0
7.5
7.0
6.5
6.0
5.5
5.0
4.5
4.0
3.5
3.0
2.5
2.0
1.5
1.0
0.5
0.0
f1 (ppm)

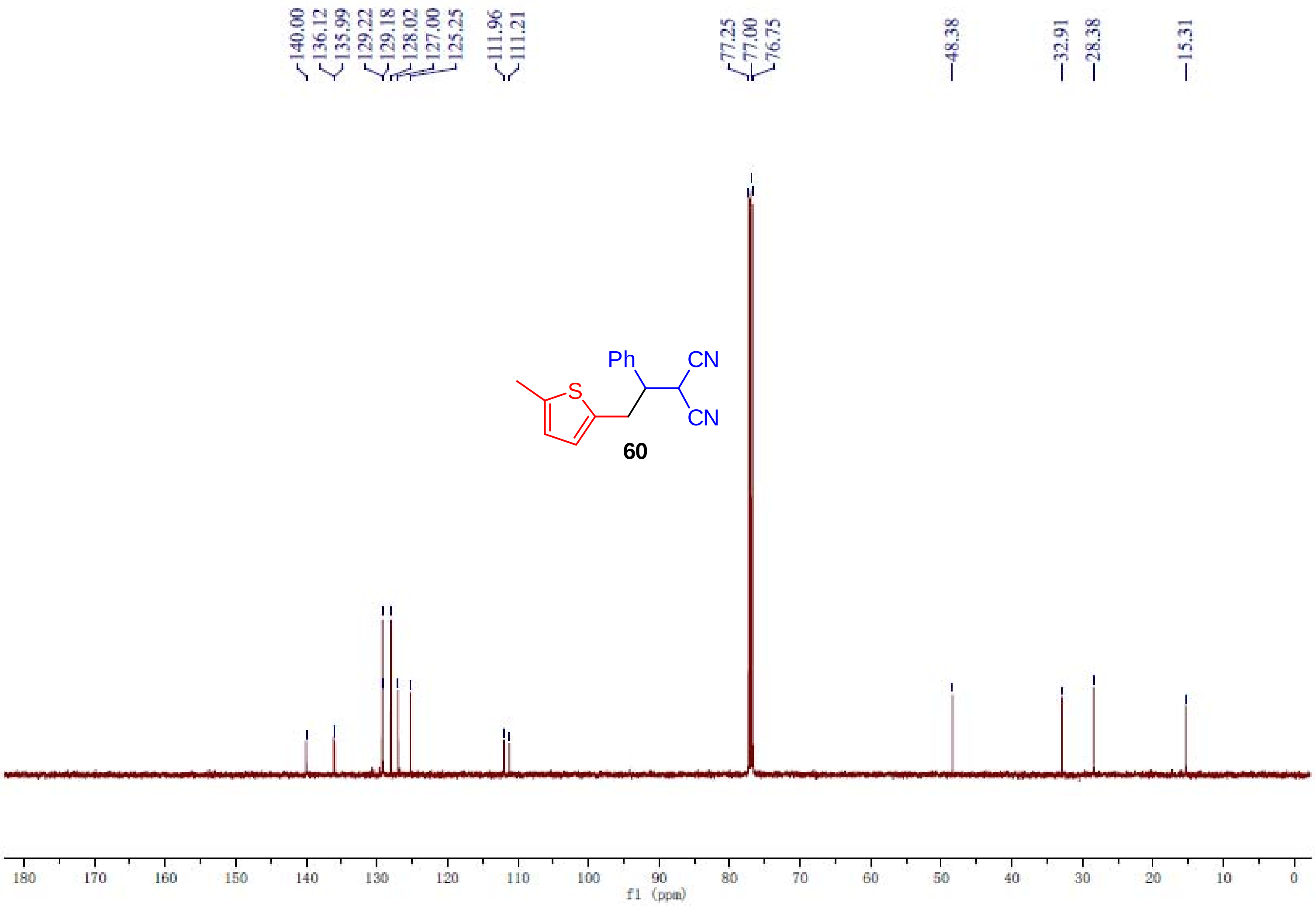
140.00
136.12
135.99
129.22
129.18
128.02
127.00
125.25
111.96
111.21
77.25
77.00
76.75
48.38
32.91
28.38
15.31
Ph
CN
S
CN
60
180
170
160
150
140
130
120
110
100
90
80
70
60
50
40
30
20
10
0
f1 (ppm)

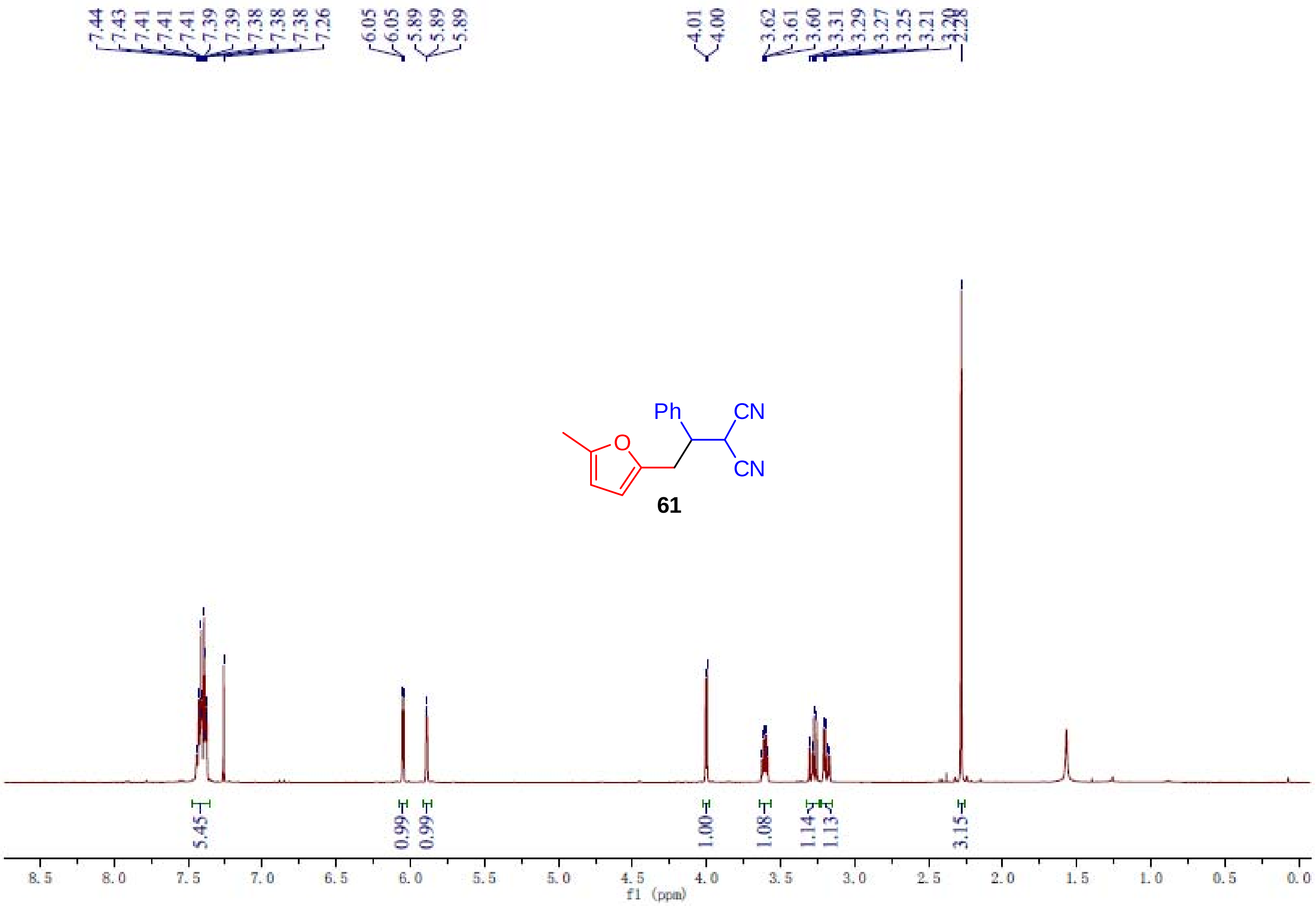
7.44
7.43
7.41
7.41
7.41
7.39
7.39
7.38
7.38
7.38
7.26
6.05
6.05
5.89
5.89
5.89
4.01
4.00
3.62
3.61
3.60
3.31
3.29
3.27
3.25
3.21
3.20
2.28
Ph
CN
O
CN
5.45
0.99
0.99
1.00
1.08
1.14
1.13
3.15
8.5
8.0
7.5
7.0
6.5
6.0
5.5
5.0
4.5
4.0
3.5
3.0
2.5
2.0
1.5
1.0
0.5
0.0
f1 (ppm)

61

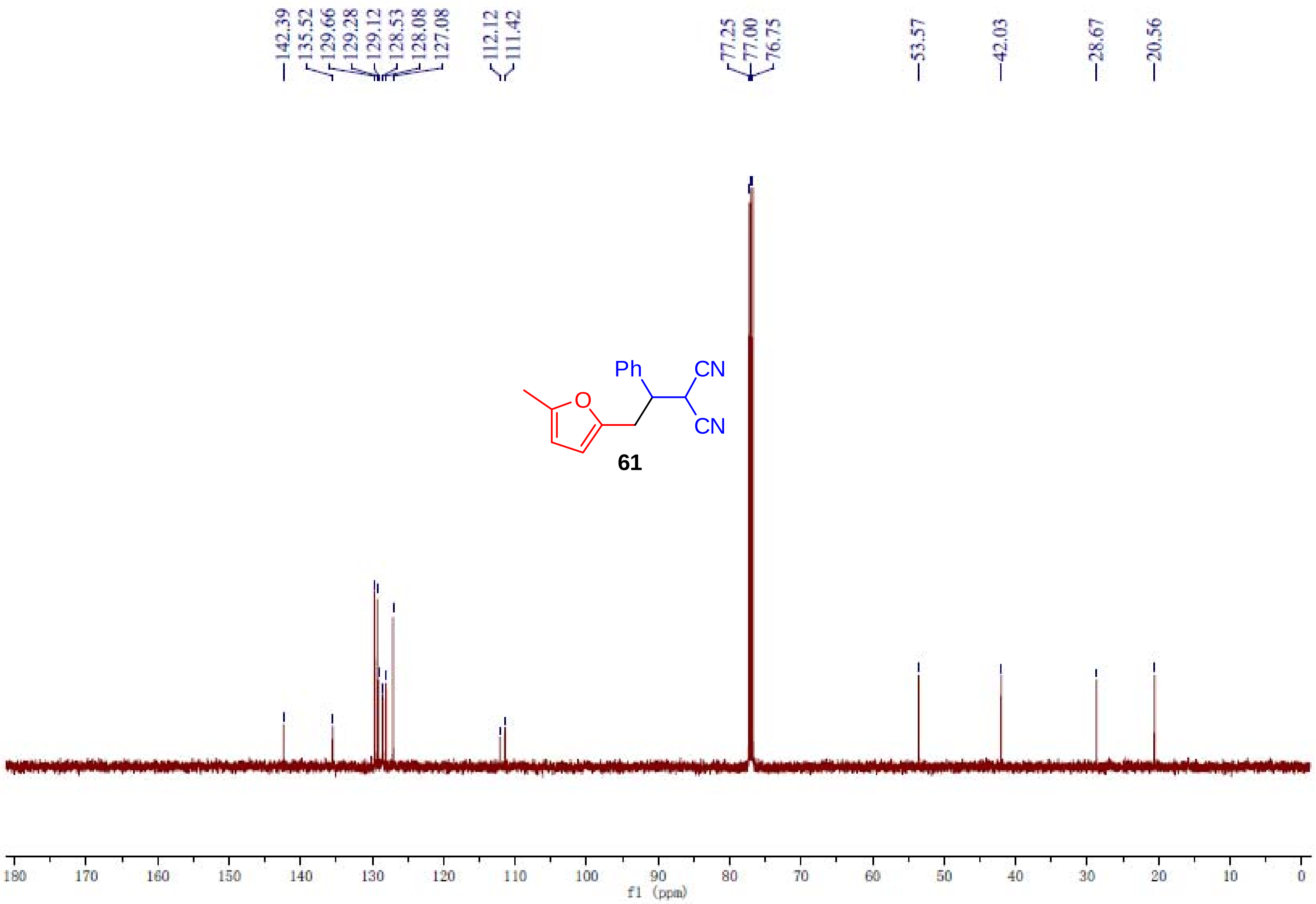

142.39
135.52
129.66
129.28
129.12
128.53
128.08
127.08
112.12
111.42
77.25
77.00
76.75
53.57
42.03
28.67
20.56
Ph
CN
O
CN
61
180
170
160
150
140
130
120
110
100
90
80
70
60
50
40
30
20
10
0
f1 (ppm)

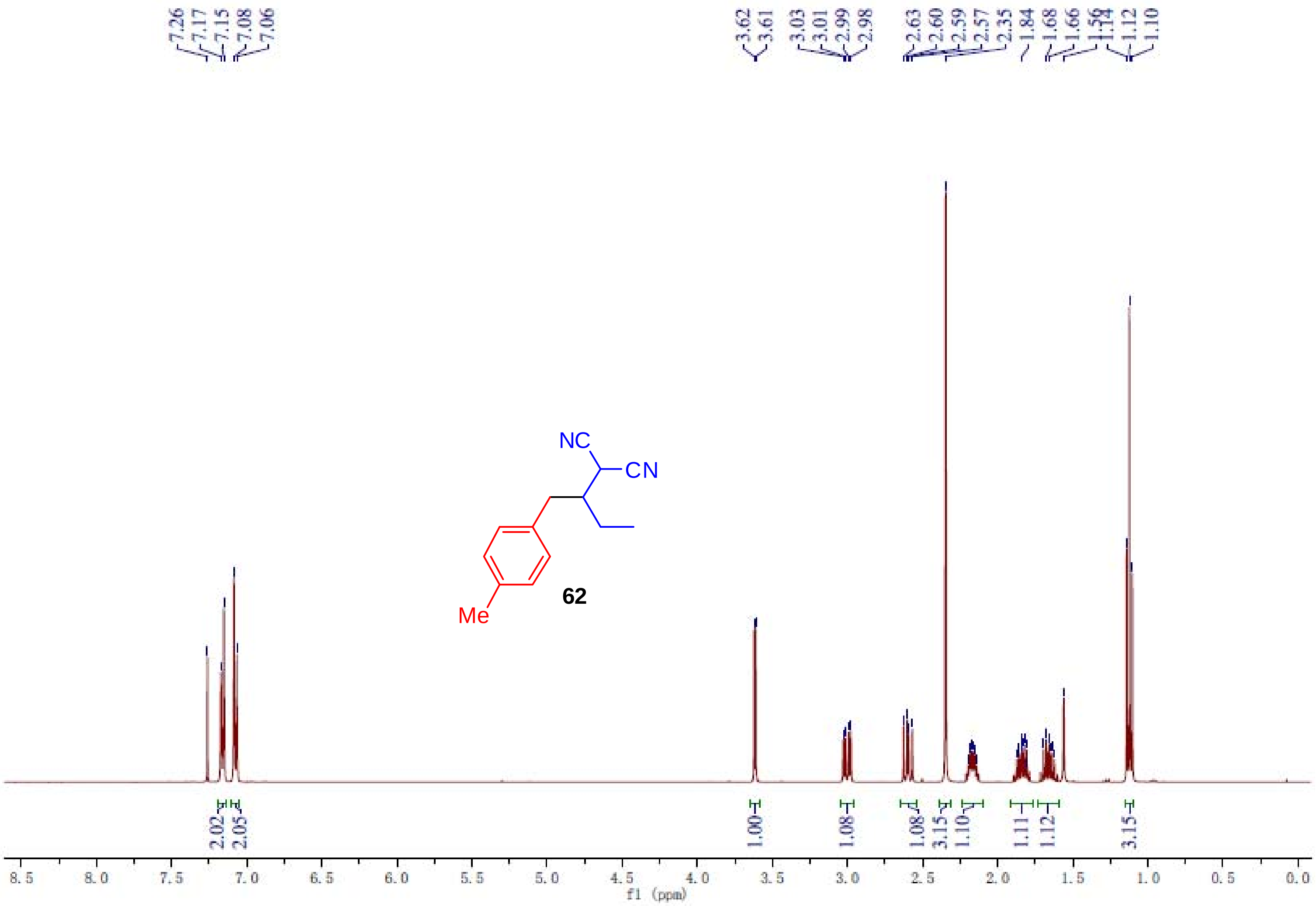
7.26
7.17
7.15
7.08
7.06
3.62
3.61
3.03
3.01
2.99
2.98
2.63
2.60
2.59
2.57
2.35
1.84
1.68
1.66
1.56
1.14
1.12
1.10
NC
CN
Me
62
2.02
2.05
1.00
1.08
1.08
3.15
1.10
1.11
1.12
3.15
8.5
8.0
7.5
7.0
6.5
6.0
5.5
5.0
4.5
4.0
3.5
3.0
2.5
2.0
1.5
1.0
0.5
0.0
f1 (ppm)

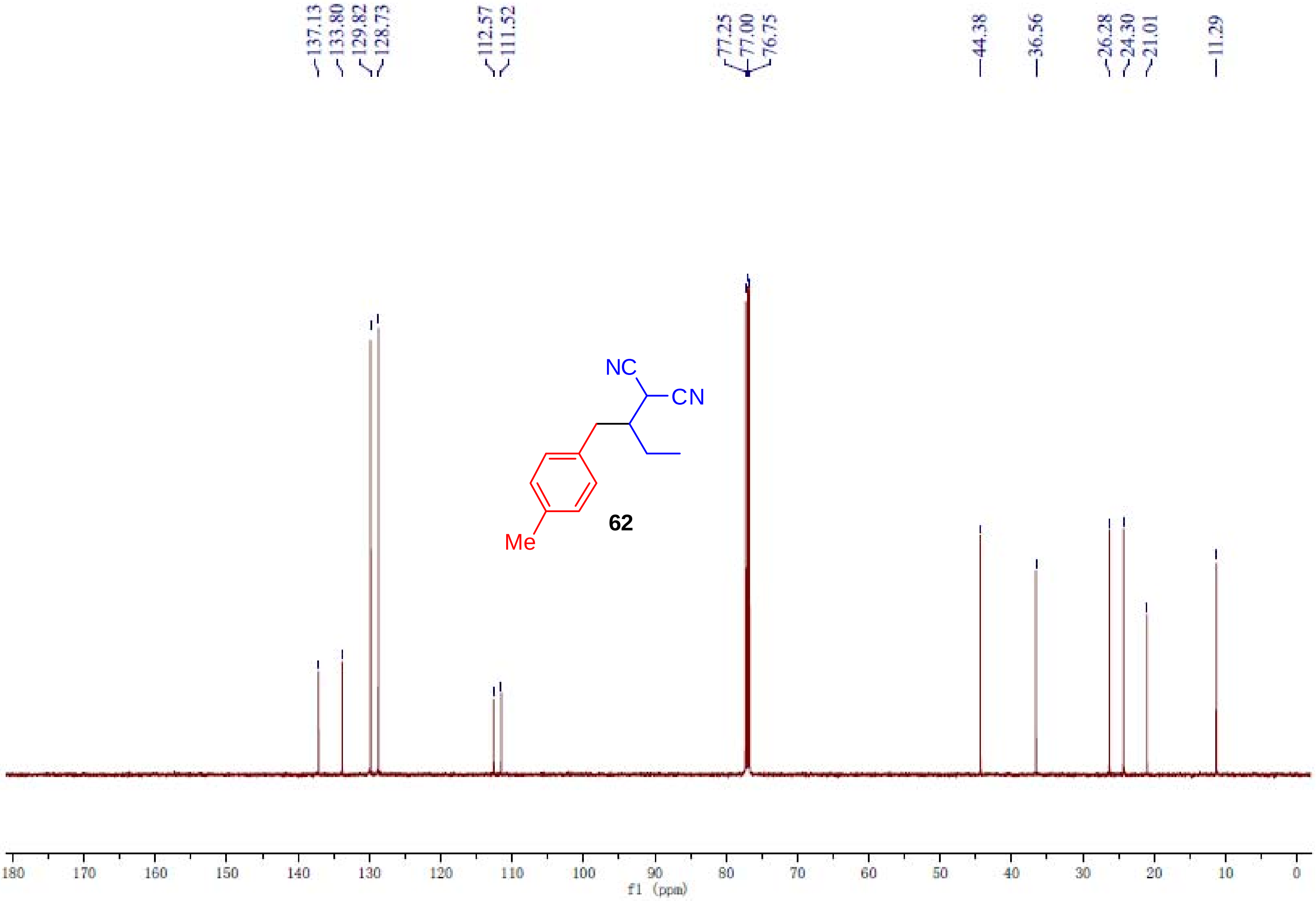

137.13
133.80
129.82
128.73
112.57
111.52
77.25
77.00
76.75
44.38
36.56
26.28
24.30
21.01
11.29
NC
CN
Me
62
180 170 160 150 140 130 120 110 100 90 80 70 60 50 40 30 20 10 0
f1 (ppm)

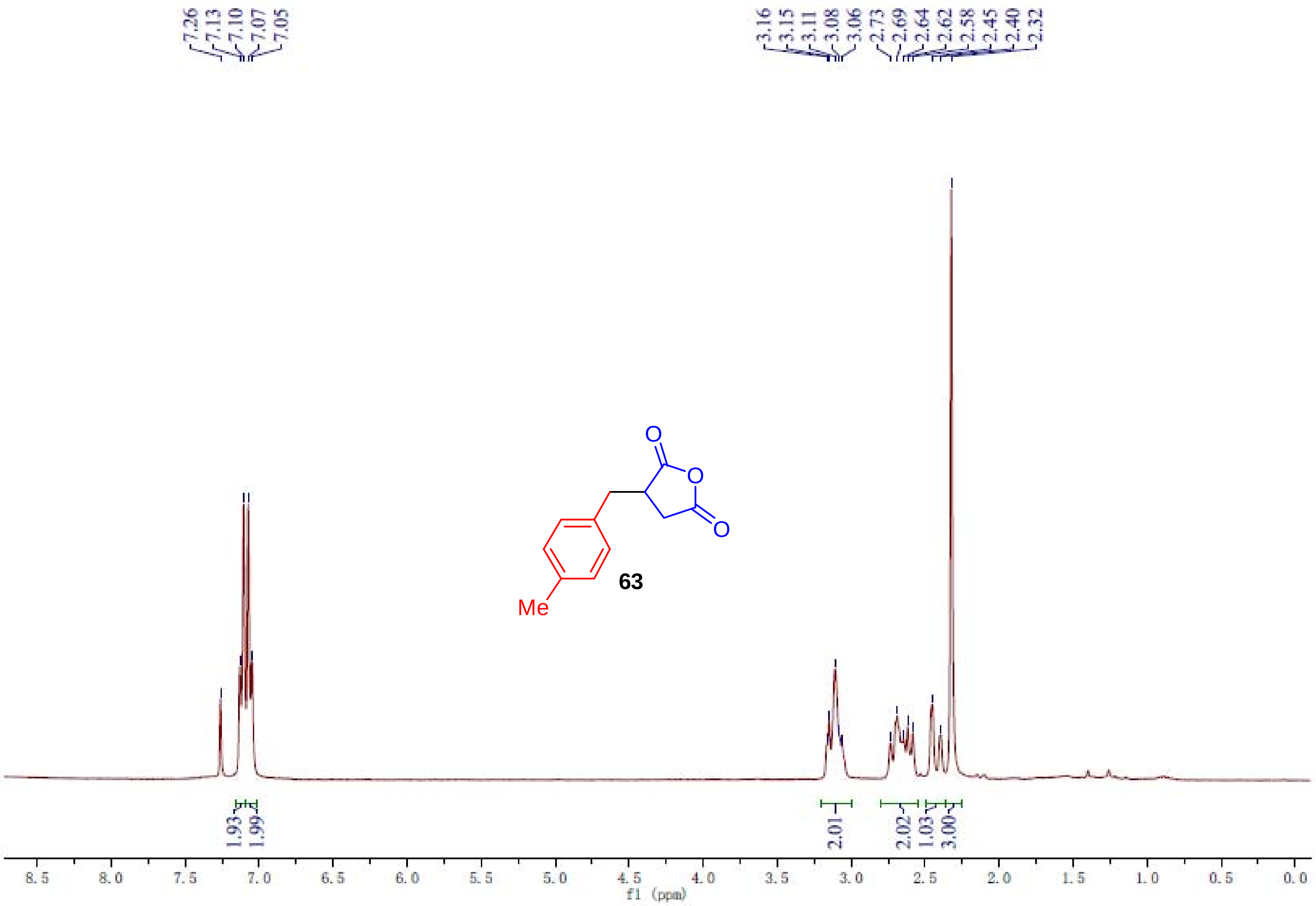
7.26
7.13
7.10
7.07
7.05
3.16
3.15
3.11
3.08
3.06
2.73
2.69
2.64
2.62
2.58
2.45
2.40
2.32
Me
63
1.93
1.99
2.01
2.02
1.03
3.00
8.5 8.0 7.5 7.0 6.5 6.0 5.5 5.0 4.5 4.0 3.5 3.0 2.5 2.0 1.5 1.0 0.5 0.0
f1 (ppm)

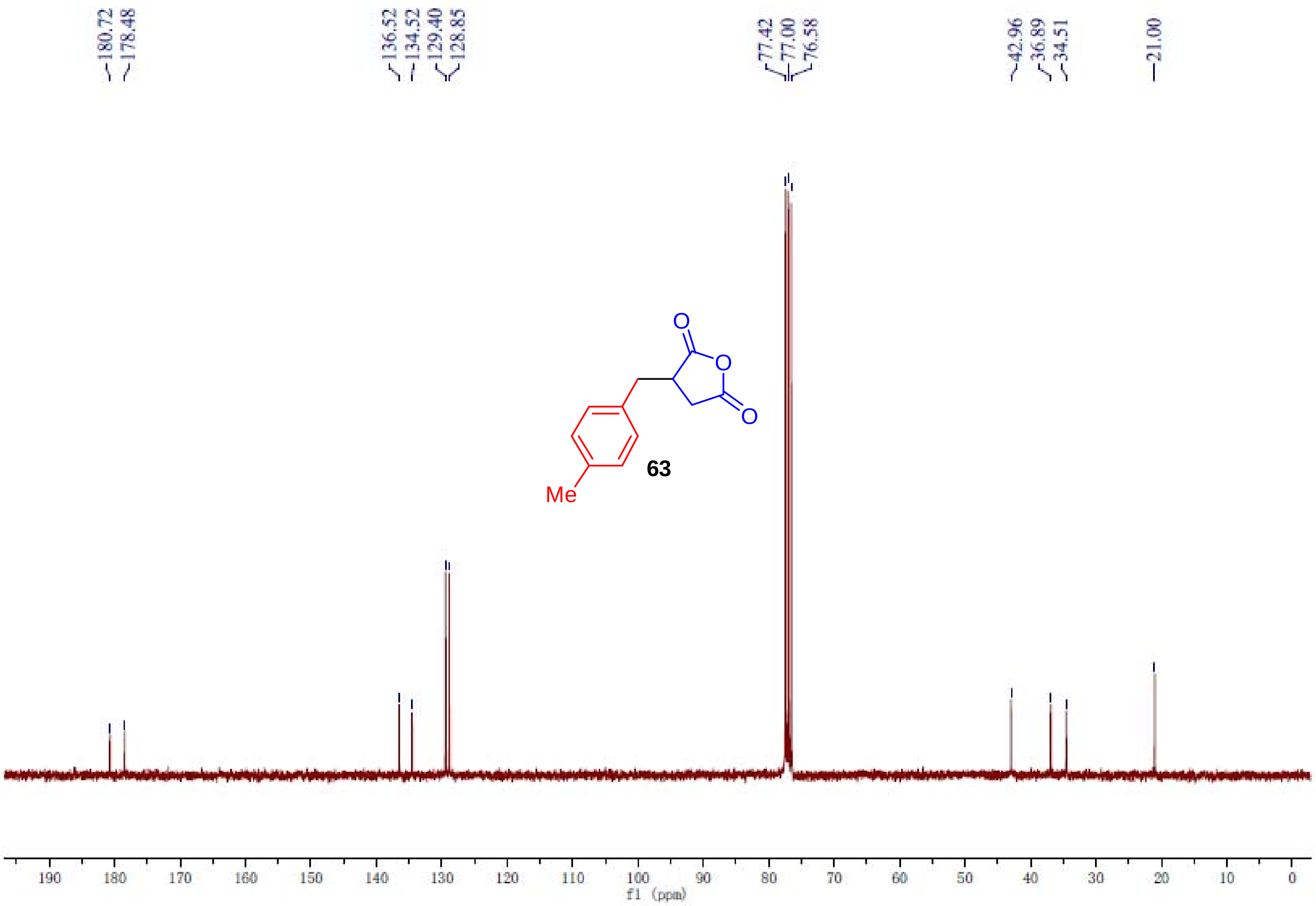

180.72
178.48
136.52
134.52
129.40
128.85
77.42
77.00
76.58
42.96
36.89
34.51
21.00
O
O
O
Me
63
190 180 170 160 150 140 130 120 110 100 90 80 70 60 50 40 30 20 10 0
f1 (ppm)

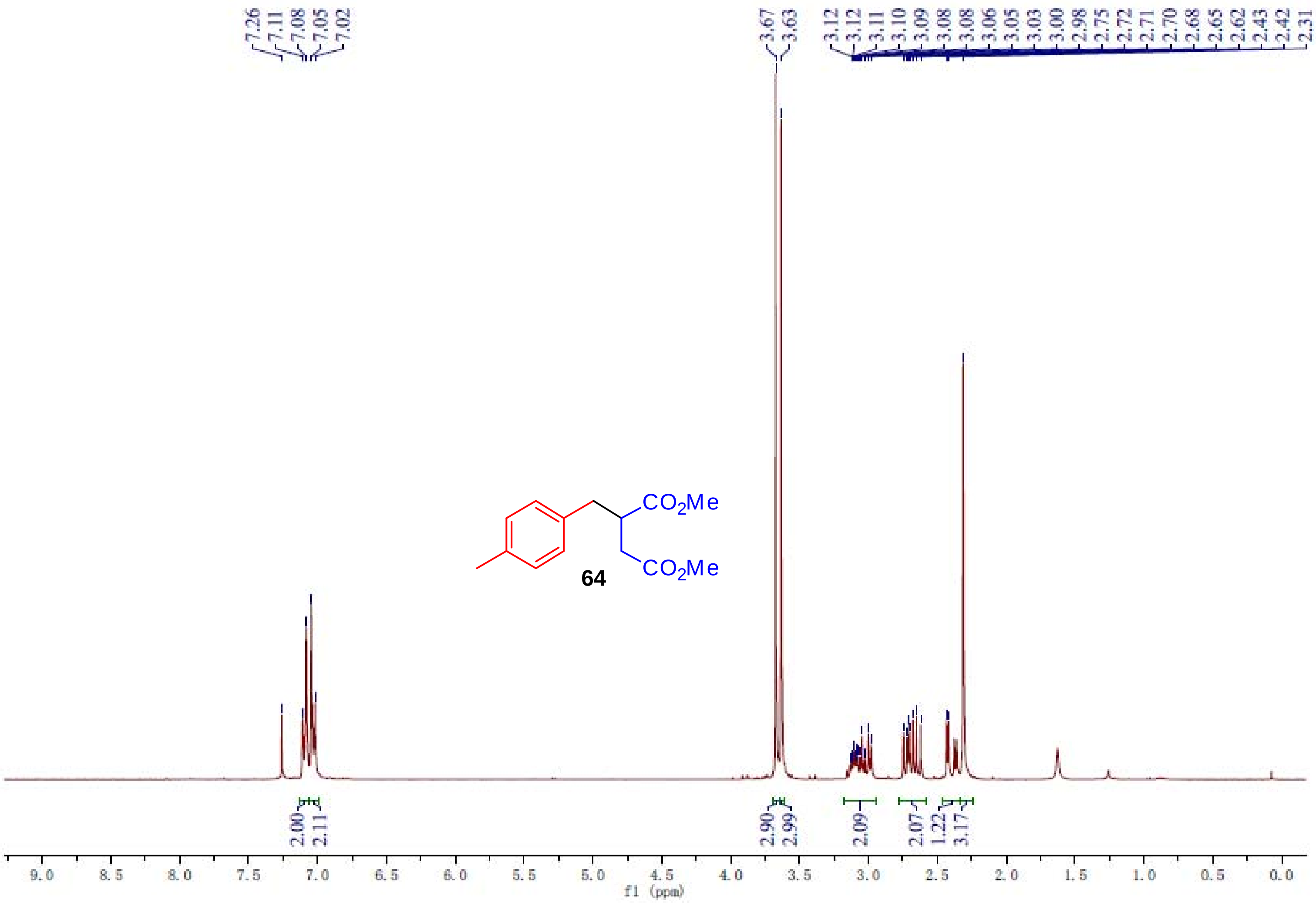
7.26
7.11
7.08
7.05
7.02
3.67
3.63
3.12
3.12
3.11
3.10
3.09
3.08
3.08
3.06
3.05
3.03
3.00
2.98
2.75
2.72
2.71
2.70
2.68
2.65
2.62
2.43
2.42
2.31
CO2Me
CO2Me
64
2.00
2.11
2.90
2.99
2.09
2.07
1.22
3.17
9.0 8.5 8.0 7.5 7.0 6.5 6.0 5.5 5.0 4.5 4.0 3.5 3.0 2.5 2.0 1.5 1.0 0.5 0.0
f1 (ppm)

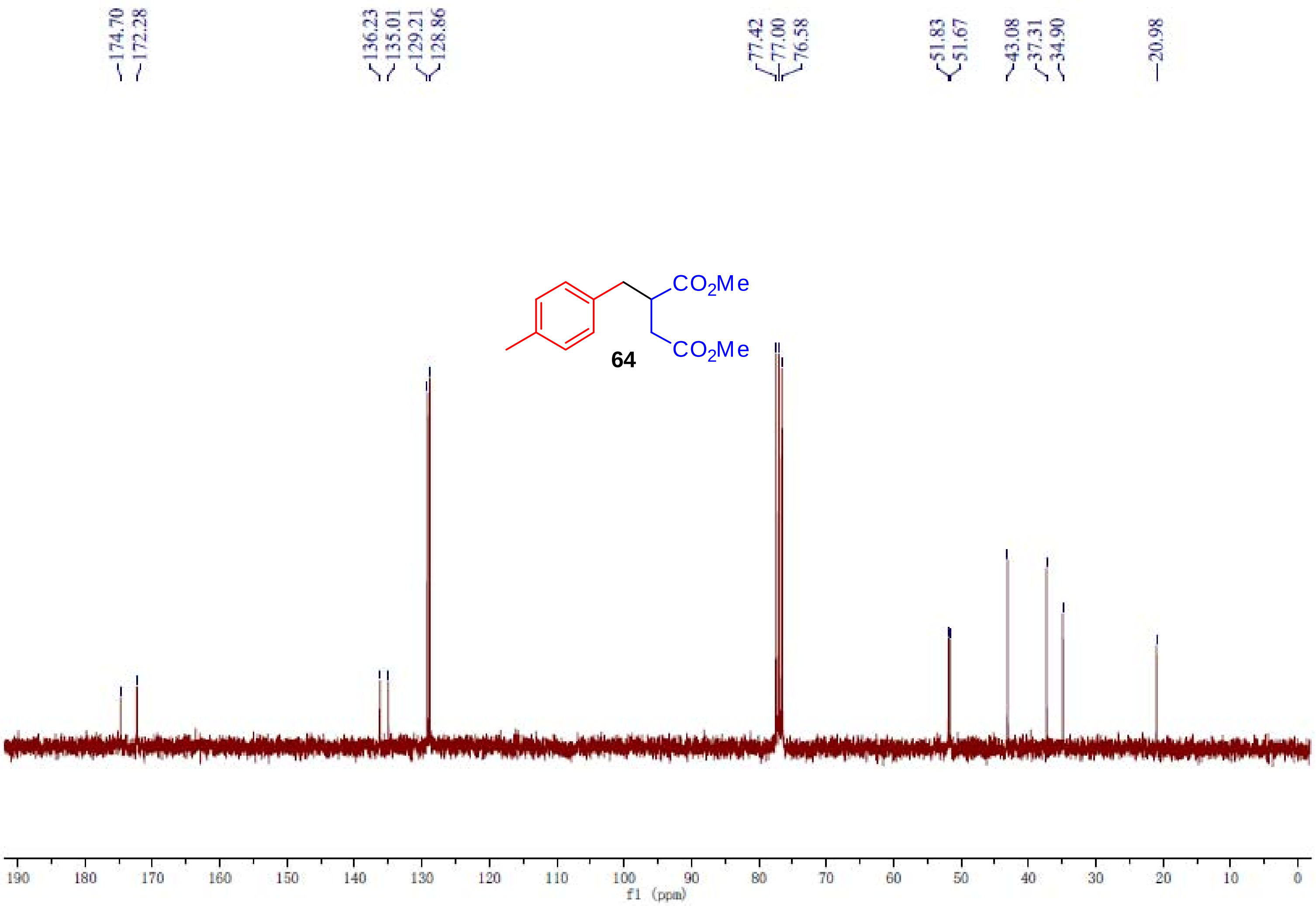

174.70
172.28
136.23
135.01
129.21
128.86
77.42
77.00
76.58
51.83
51.67
43.08
37.31
34.90
20.98
CO_2Me
CO_2Me
64
190 180 170 160 150 140 130 120 110 100 90 80 70 60 50 40 30 20 10 0
f1 (ppm)

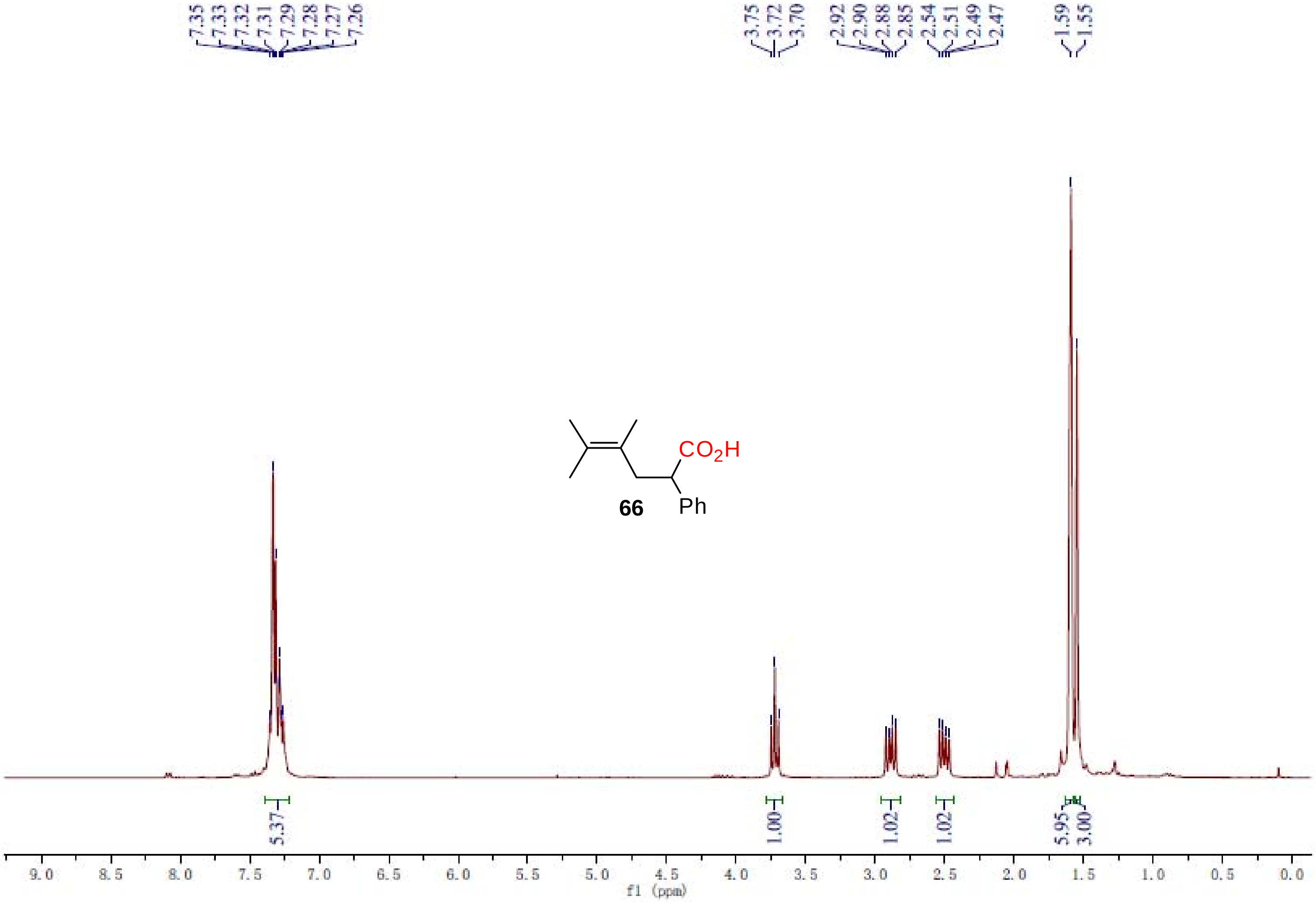
7.35
7.33
7.32
7.31
7.29
7.28
7.27
7.26
3.75
3.72
3.70
2.92
2.90
2.88
2.85
2.54
2.51
2.49
2.47
1.59
1.55
CO_2H
Ph
66
5.37
1.00
1.02
1.02
5.95
3.00
9.0 8.5 8.0 7.5 7.0 6.5 6.0 5.5 5.0 4.5 4.0 3.5 3.0 2.5 2.0 1.5 1.0 0.5 0.0
f1 (ppm)

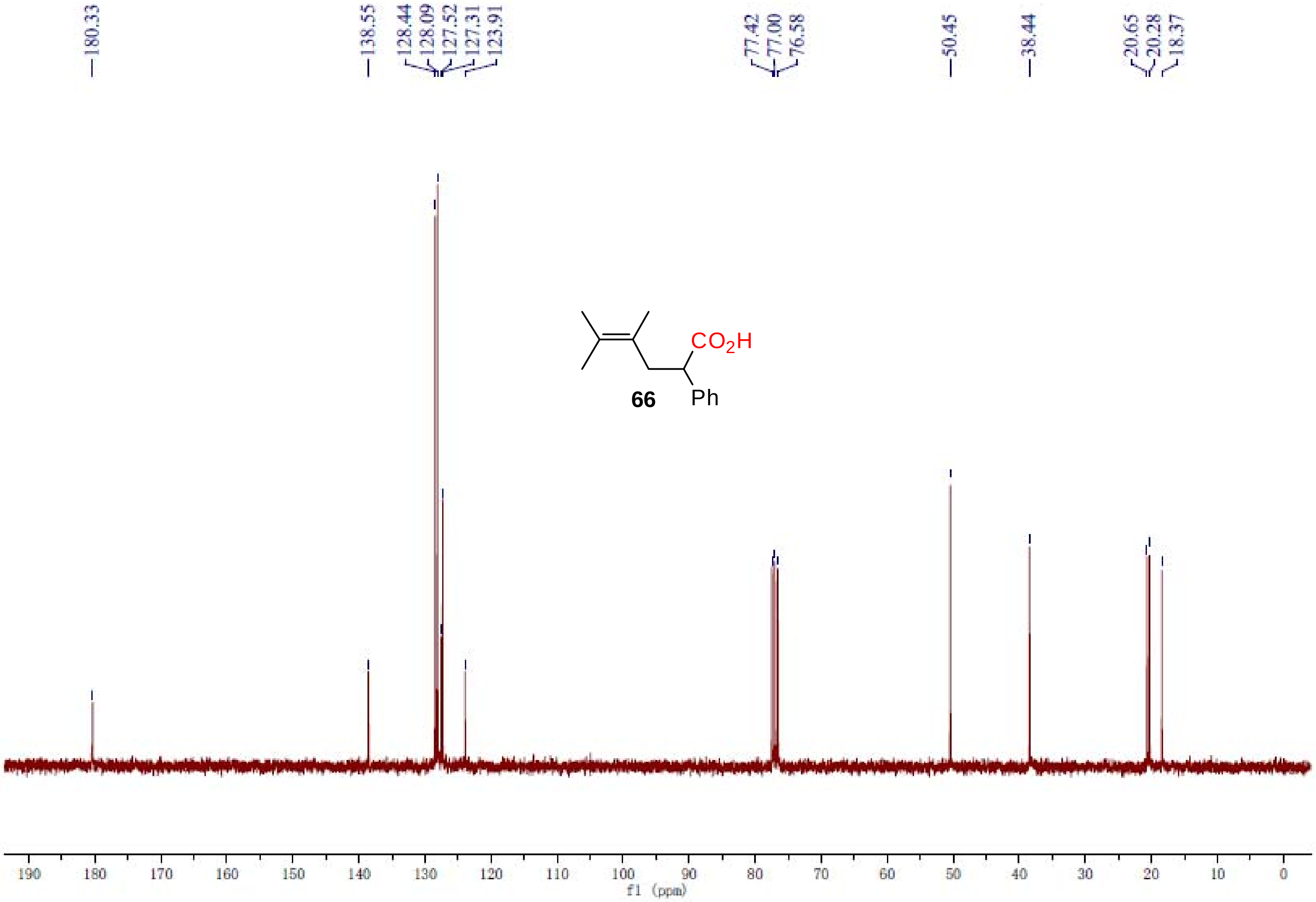
180.33
138.55
128.44
128.09
127.52
127.31
123.91
77.42
77.00
76.58
50.45
38.44
20.65
20.28
18.37
CO2H
Ph
66
190
180
170
160
150
140
130
120
110
100
90
80
70
60
50
40
30
20
10
0
f1 (ppm)

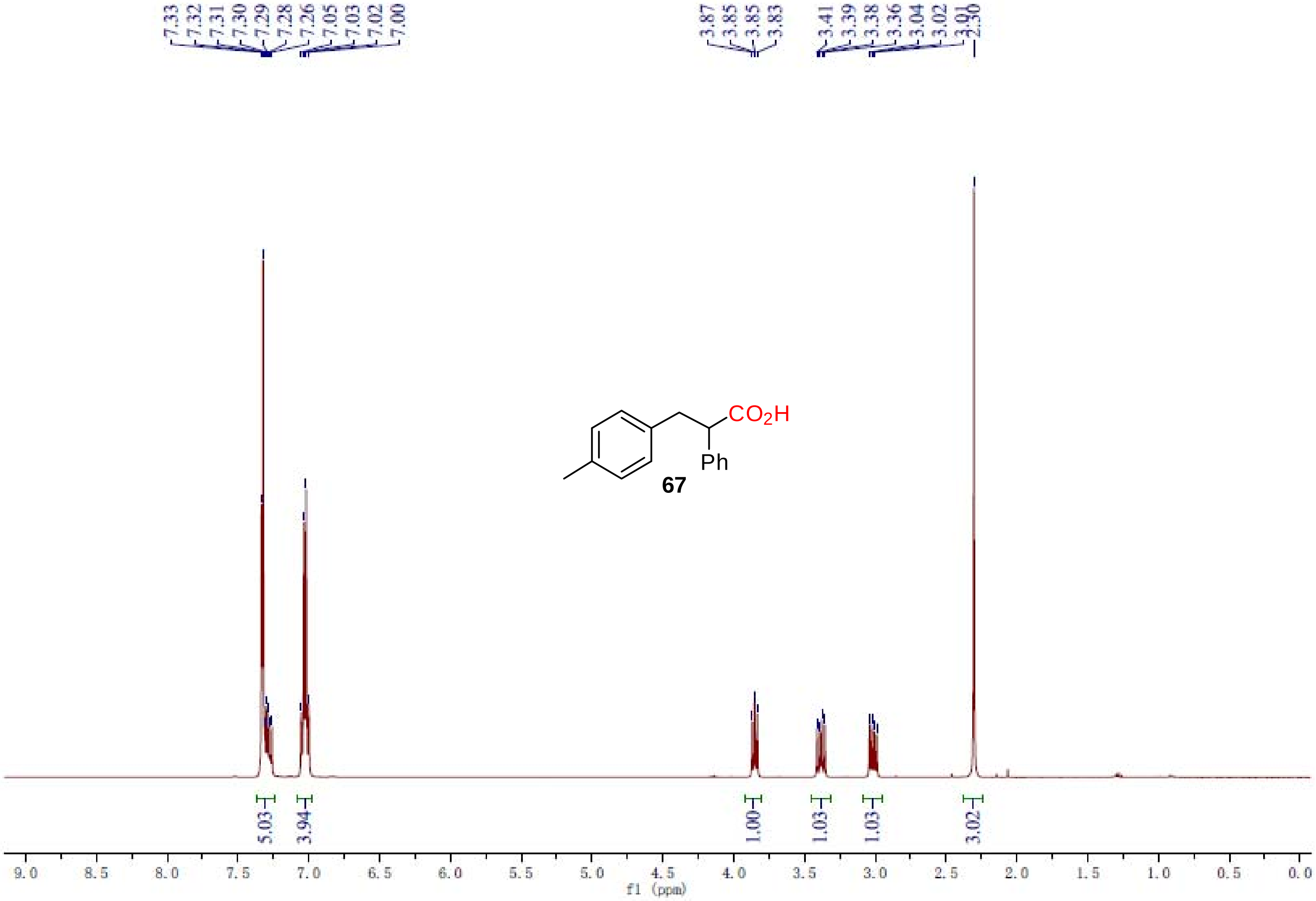
7.33
7.32
7.31
7.30
7.29
7.28
7.26
7.05
7.03
7.02
7.00
3.87
3.85
3.85
3.83
3.41
3.39
3.38
3.36
3.04
3.02
3.01
2.30
CO2H
Ph
67
5.03
3.94
1.00
1.03
1.03
3.02
9.0
8.5
8.0
7.5
7.0
6.5
6.0
5.5
5.0
4.5
4.0
3.5
3.0
2.5
2.0
1.5
1.0
0.5
0.0
f1 (ppm)

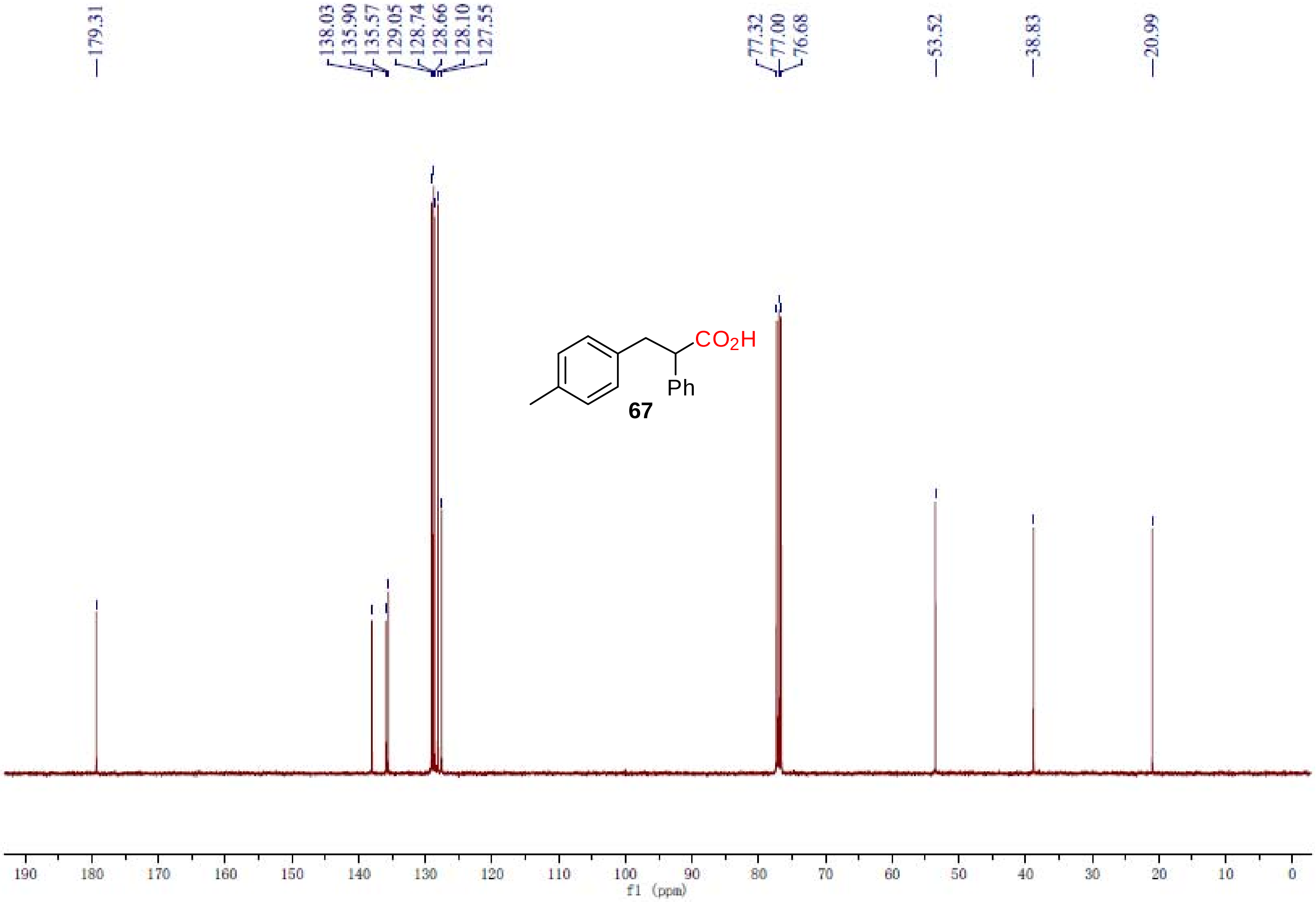
179.31
138.03
135.90
135.57
129.05
128.74
128.66
128.10
127.55
77.32
77.00
76.68
53.52
38.83
20.99
CO2H
Ph
67
190 180 170 160 150 140 130 120 110 100 90 80 70 60 50 40 30 20 10 0
f1 (ppm)

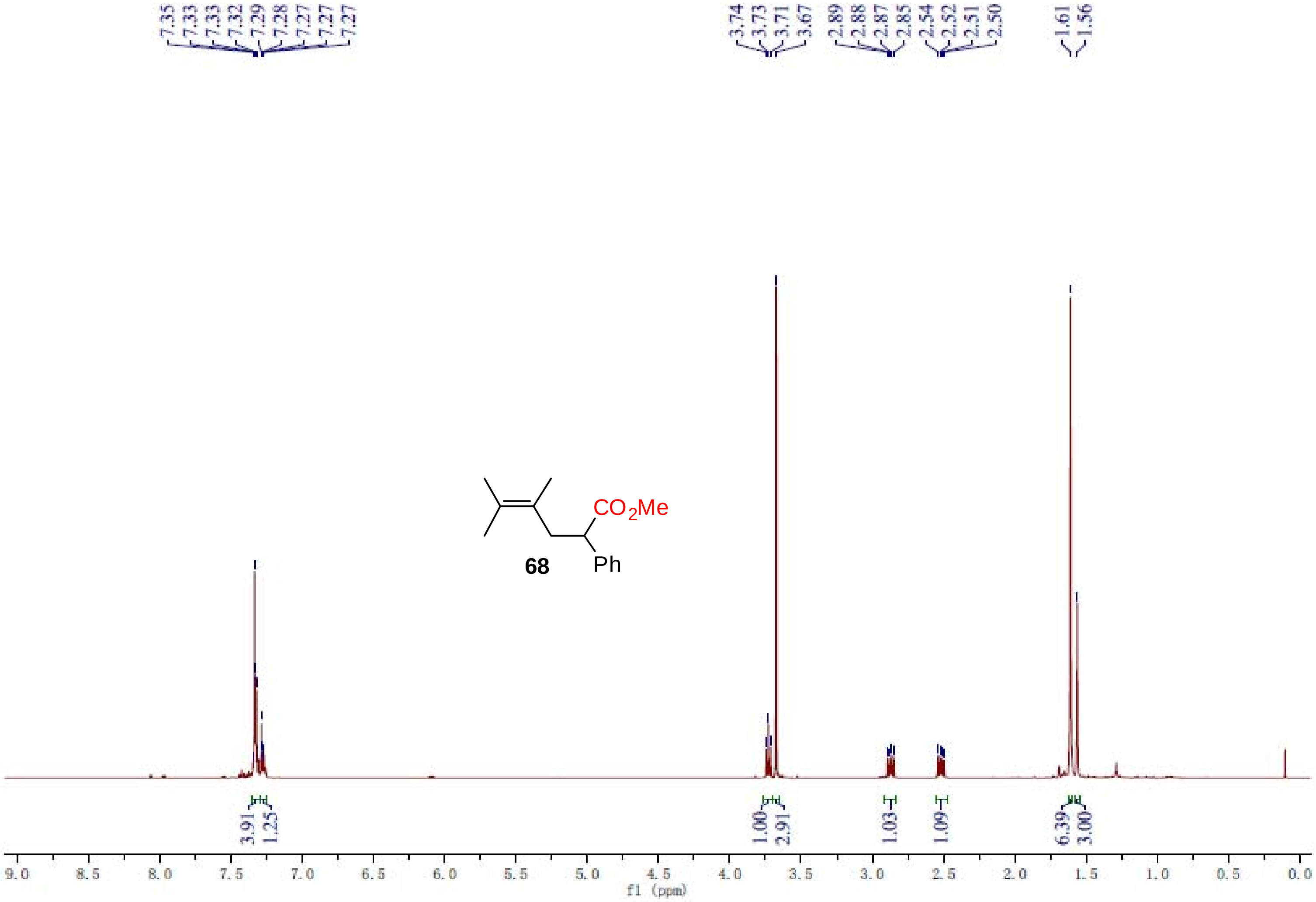
7.35
7.33
7.33
7.32
7.29
7.28
7.27
7.27
7.27
3.74
3.73
3.71
3.67
2.89
2.88
2.87
2.85
2.54
2.52
2.51
2.50
1.61
1.56
CO2Me
68
Ph
3.91
1.25
1.00
2.91
1.03
1.09
6.39
3.00
9.0
8.5
8.0
7.5
7.0
6.5
6.0
5.5
5.0
4.5
4.0
3.5
3.0
2.5
2.0
1.5
1.0
0.5
0.0
f1 (ppm)

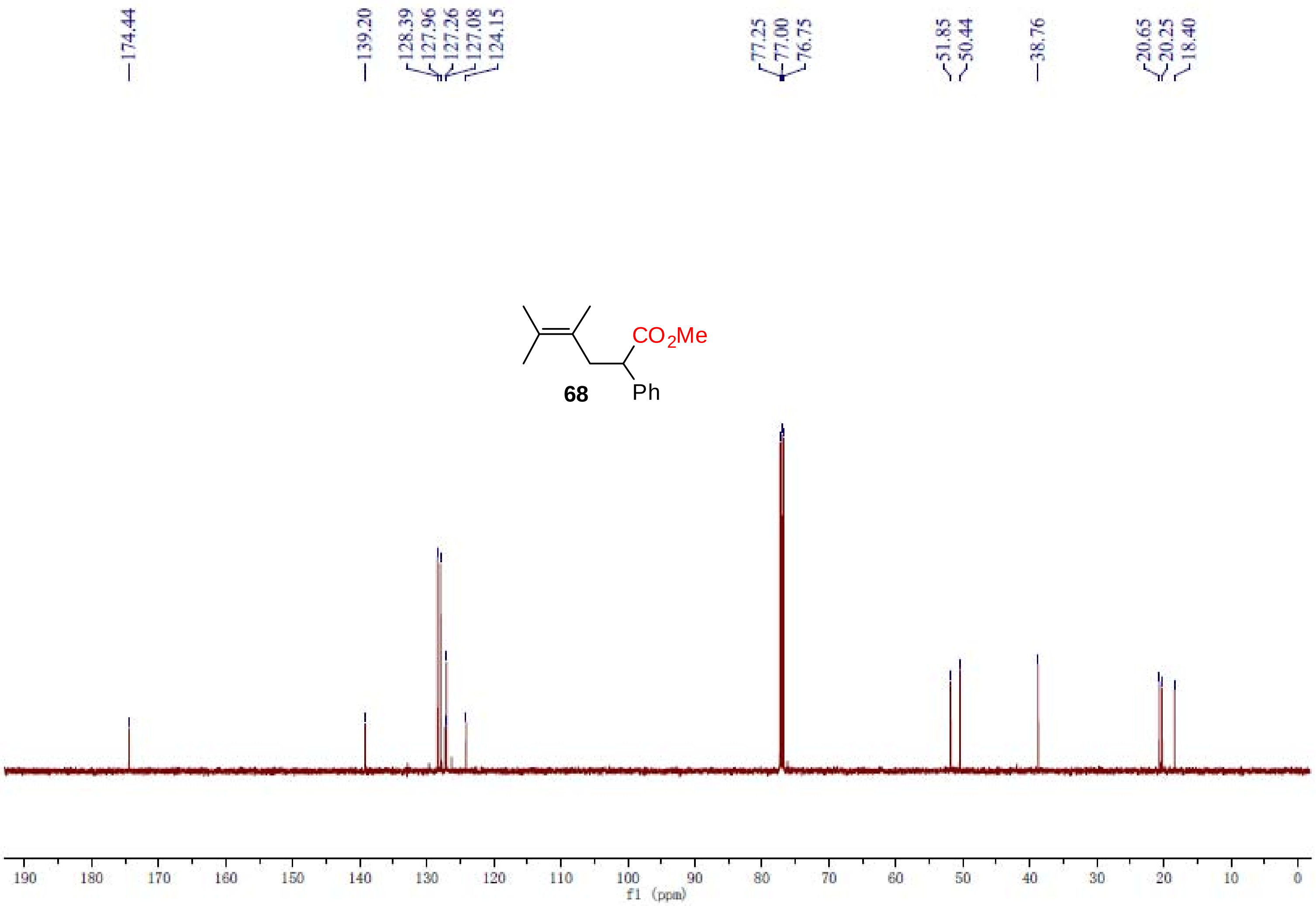
174.44
139.20
128.39
127.96
127.26
127.08
124.15
77.25
77.00
76.75
51.85
50.44
38.76
20.65
20.25
18.40
CO2Me
68
Ph
190 180 170 160 150 140 130 120 110 100 90 80 70 60 50 40 30 20 10 0
f1 (ppm)

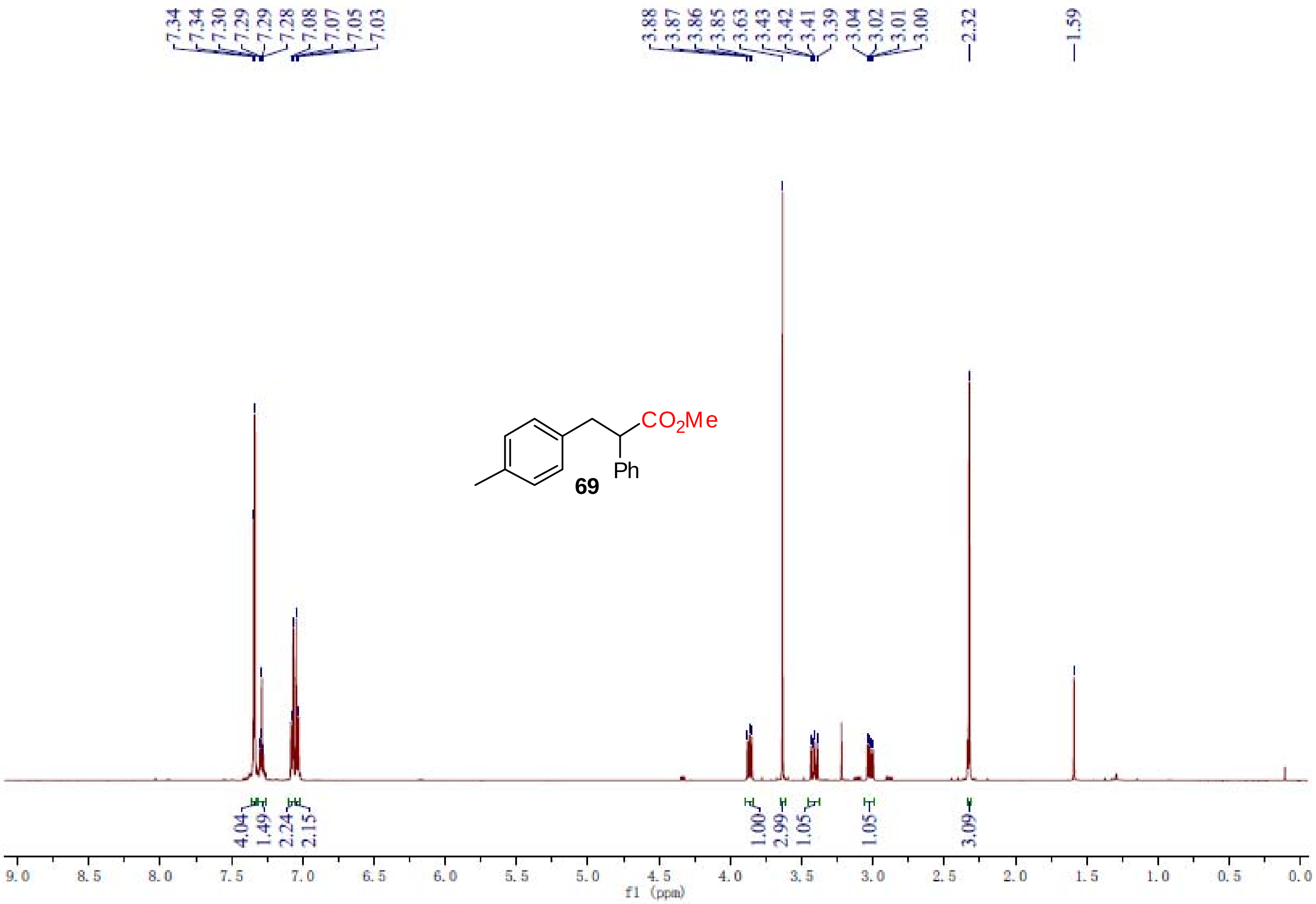
7.34
7.34
7.30
7.29
7.29
7.28
7.08
7.07
7.05
7.03
3.88
3.87
3.86
3.85
3.63
3.43
3.42
3.41
3.39
3.04
3.02
3.01
3.00
2.32
1.59
CO2Me
Ph
69
4.04
1.49
2.24
2.15
1.00
2.99
1.05
1.05
3.09
9.0
8.5
8.0
7.5
7.0
6.5
6.0
5.5
5.0
4.5
4.0
3.5
3.0
2.5
2.0
1.5
1.0
0.5
0.0
f1 (ppm)

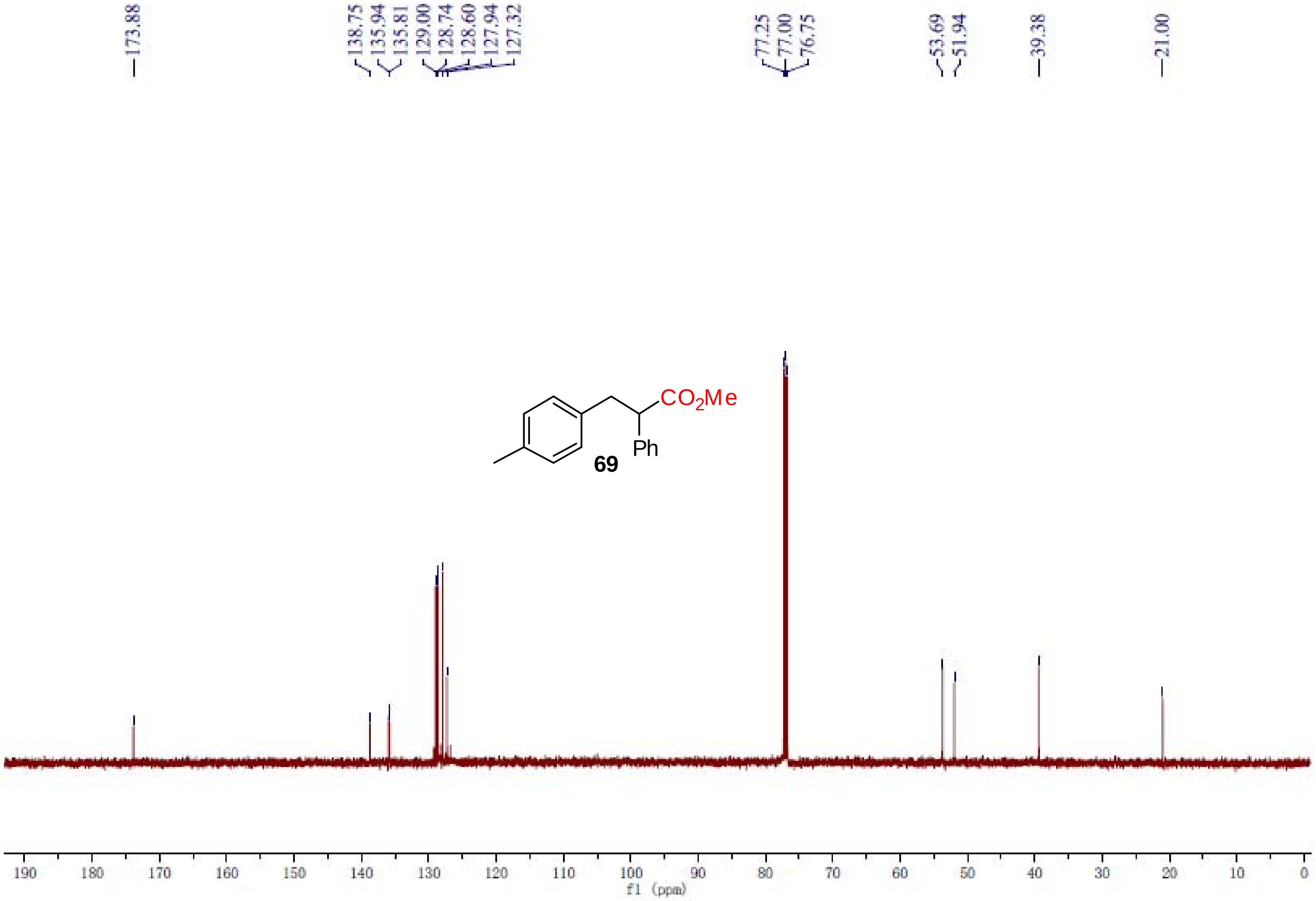
173.88
138.75
135.94
135.81
129.00
128.74
128.60
127.94
127.32
77.25
77.00
76.75
53.69
51.94
39.38
21.00
CO2Me
Ph
69
190
180
170
160
150
140
130
120
110
100
90
80
70
60
50
40
30
20
10
0
f1 (ppm)

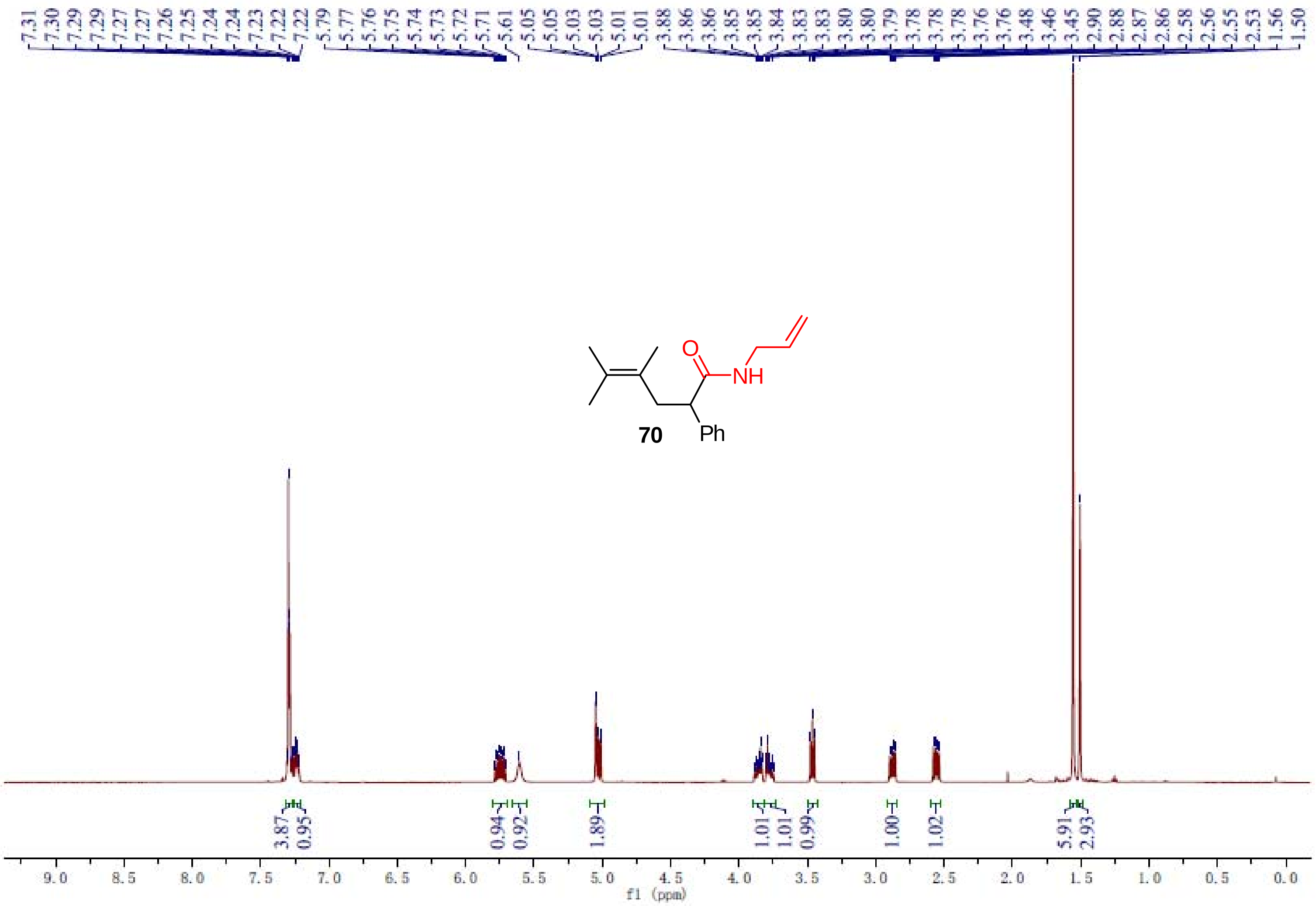
7.31 7.30 7.29 7.29 7.27 7.27 7.26 7.25 7.24 7.24 7.23 7.22 7.22 5.79 5.77 5.76 5.75 5.74 5.73 5.72 5.71 5.61 5.05 5.05 5.03 5.03 5.01 5.01 3.88 3.86 3.86 3.85 3.85 3.84 3.83 3.83 3.80 3.80 3.79 3.78 3.78 3.78 3.76 3.76 3.48 3.46 3.45 2.90 2.88 2.87 2.86 2.58 2.56 2.55 2.53 1.56 1.50
O
NH
70
Ph
3.87
0.95
0.94
0.92
1.89
1.01
1.01
0.99
1.00
1.02
5.91
2.93
9.0 8.5 8.0 7.5 7.0 6.5 6.0 5.5 5.0 4.5 4.0 3.5 3.0 2.5 2.0 1.5 1.0 0.5 0.0
f1 (ppm)

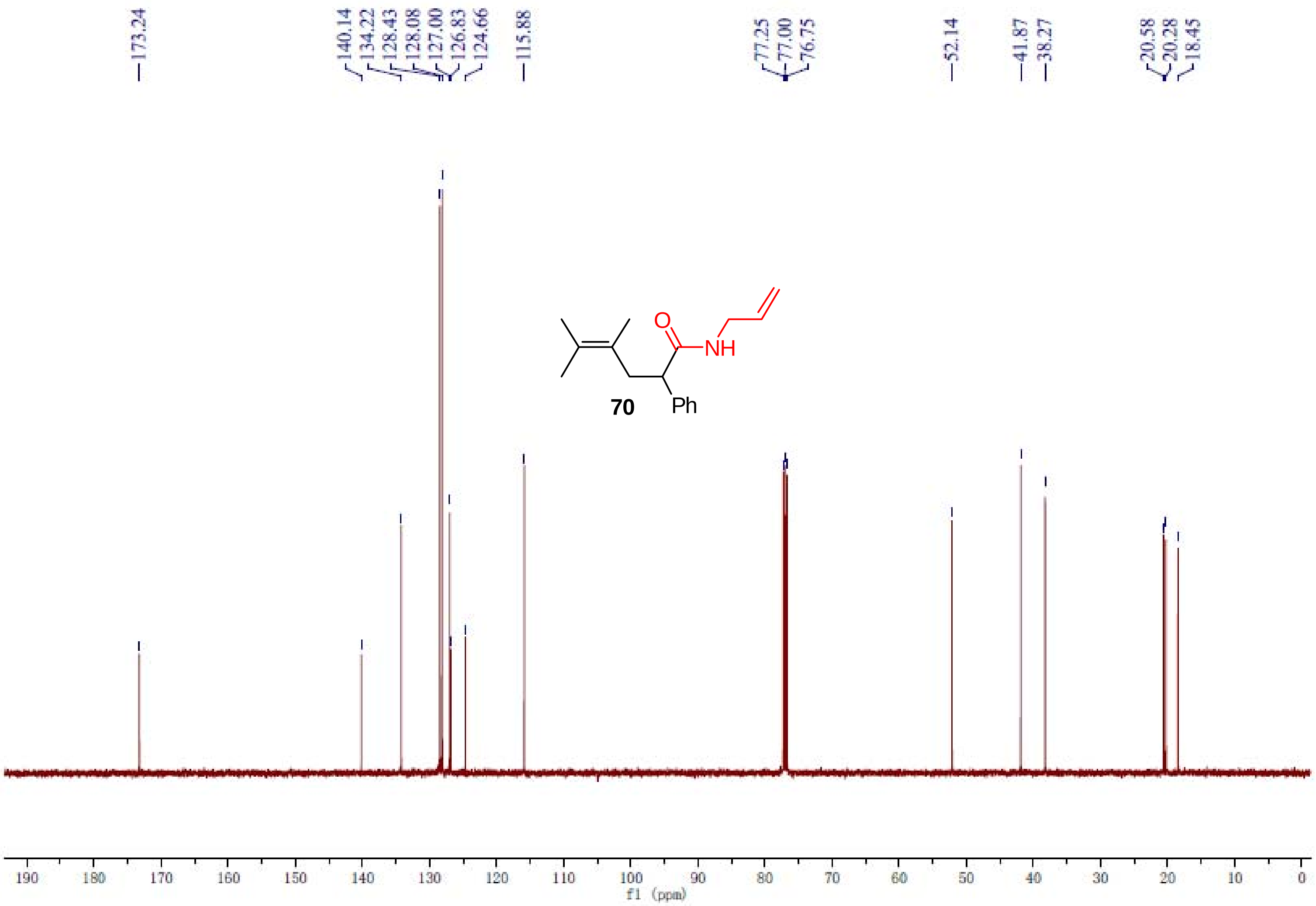

173.24
140.14
134.22
128.43
128.08
127.00
126.83
124.66
115.88
77.25
77.00
76.75
52.14
41.87
38.27
20.58
20.28
18.45
O
NH
Ph
70
190
180
170
160
150
140
130
120
110
100
90
80
70
60
50
40
30
20
10
0
f1 (ppm)

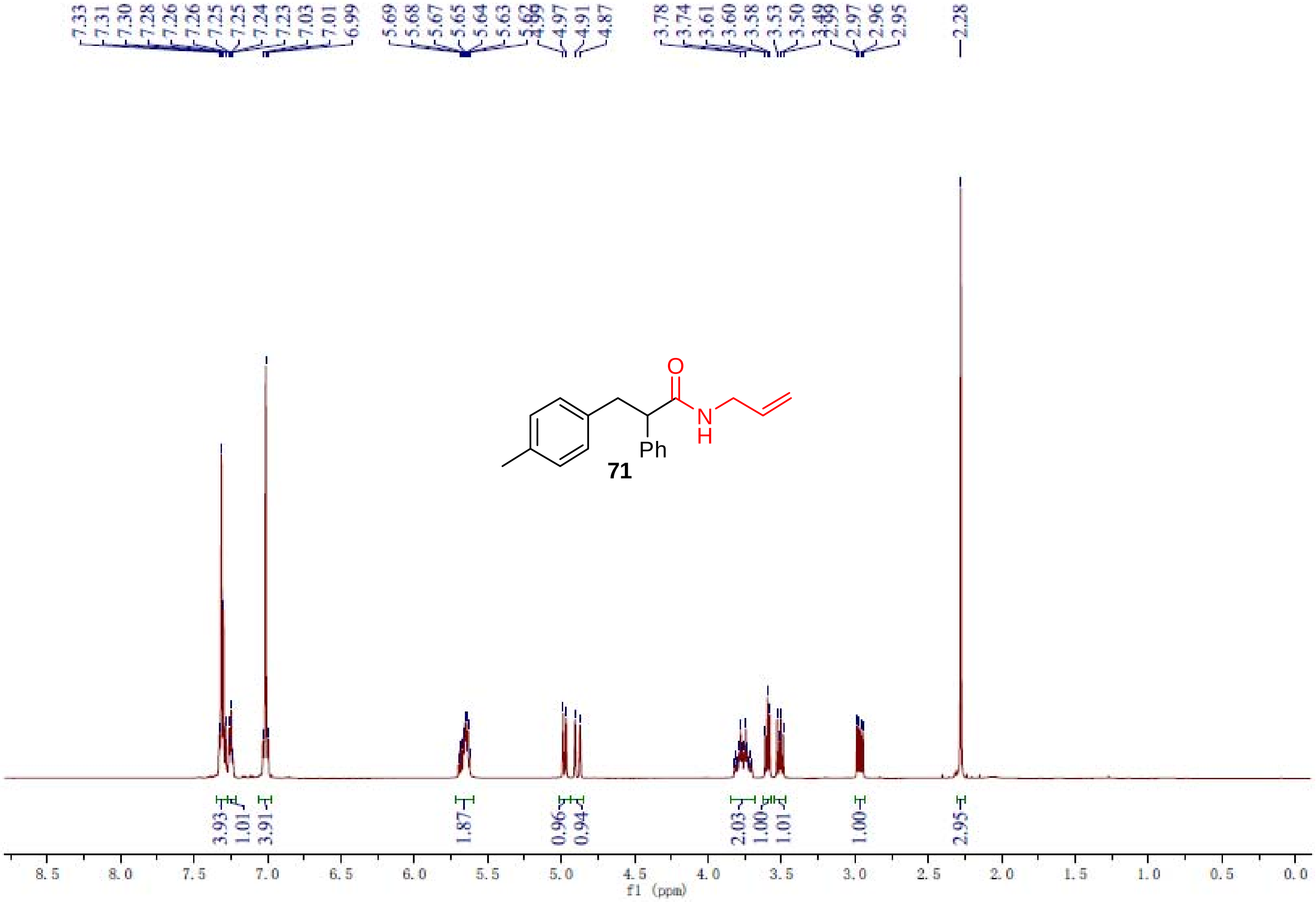
7.33 7.31 7.30 7.28 7.26 7.26 7.25 7.25 7.24 7.23 7.03 7.01 6.99
5.69 5.68 5.67 5.65 5.64 5.63 4.97 4.91 4.87
3.78 3.74 3.61 3.60 3.58 3.53 3.50 2.97 2.96 2.95
2.28
O
N
H
Ph
71
3.93 1.01 3.91 1.87 0.96 0.94 2.03 1.00 1.01 1.00 2.95
8.5 8.0 7.5 7.0 6.5 6.0 5.5 5.0 4.5 4.0 3.5 3.0 2.5 2.0 1.5 1.0 0.5 0.0
f1 (ppm)

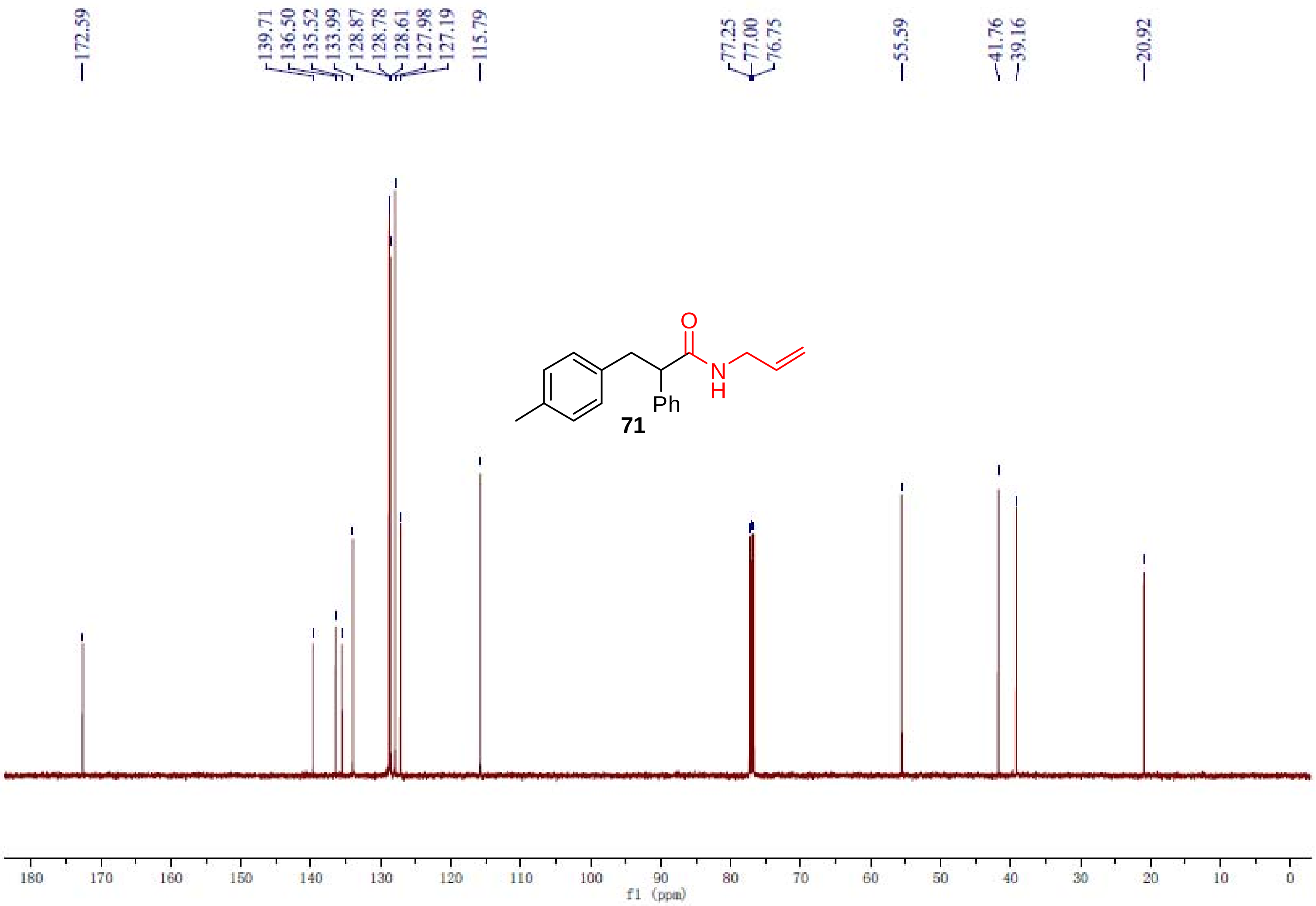

172.59
139.71
136.50
135.52
133.99
128.87
128.78
128.61
127.98
127.19
115.79
77.25
77.00
76.75
55.59
41.76
39.16
20.92
O
N
H
Ph
71
180
170
160
150
140
130
120
110
100
90
80
70
60
50
40
30
20
10
0
f1 (ppm)

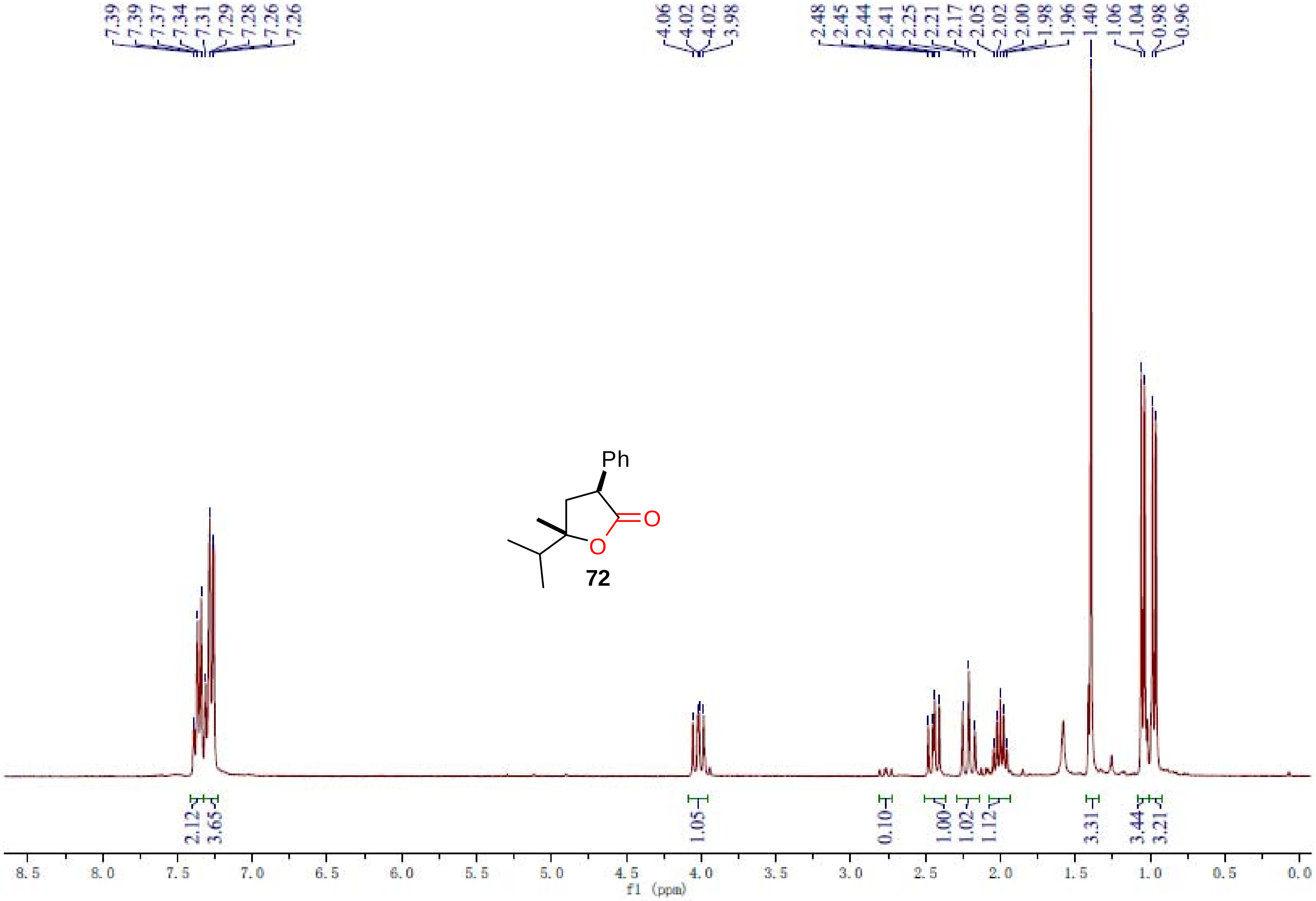
7.39
7.39
7.37
7.34
7.31
7.29
7.28
7.26
7.26
4.06
4.02
4.02
3.98
2.48
2.45
2.44
2.41
2.25
2.21
2.17
2.05
2.02
2.00
1.98
1.96
1.40
1.06
1.04
0.98
0.96
Ph
O
O
72
2.12
3.65
1.05
0.10
1.00
1.02
1.12
3.31
3.44
3.21
8.5
8.0
7.5
7.0
6.5
6.0
5.5
5.0
4.5
4.0
3.5
3.0
2.5
2.0
1.5
1.0
0.5
0.0
f1 (ppm)

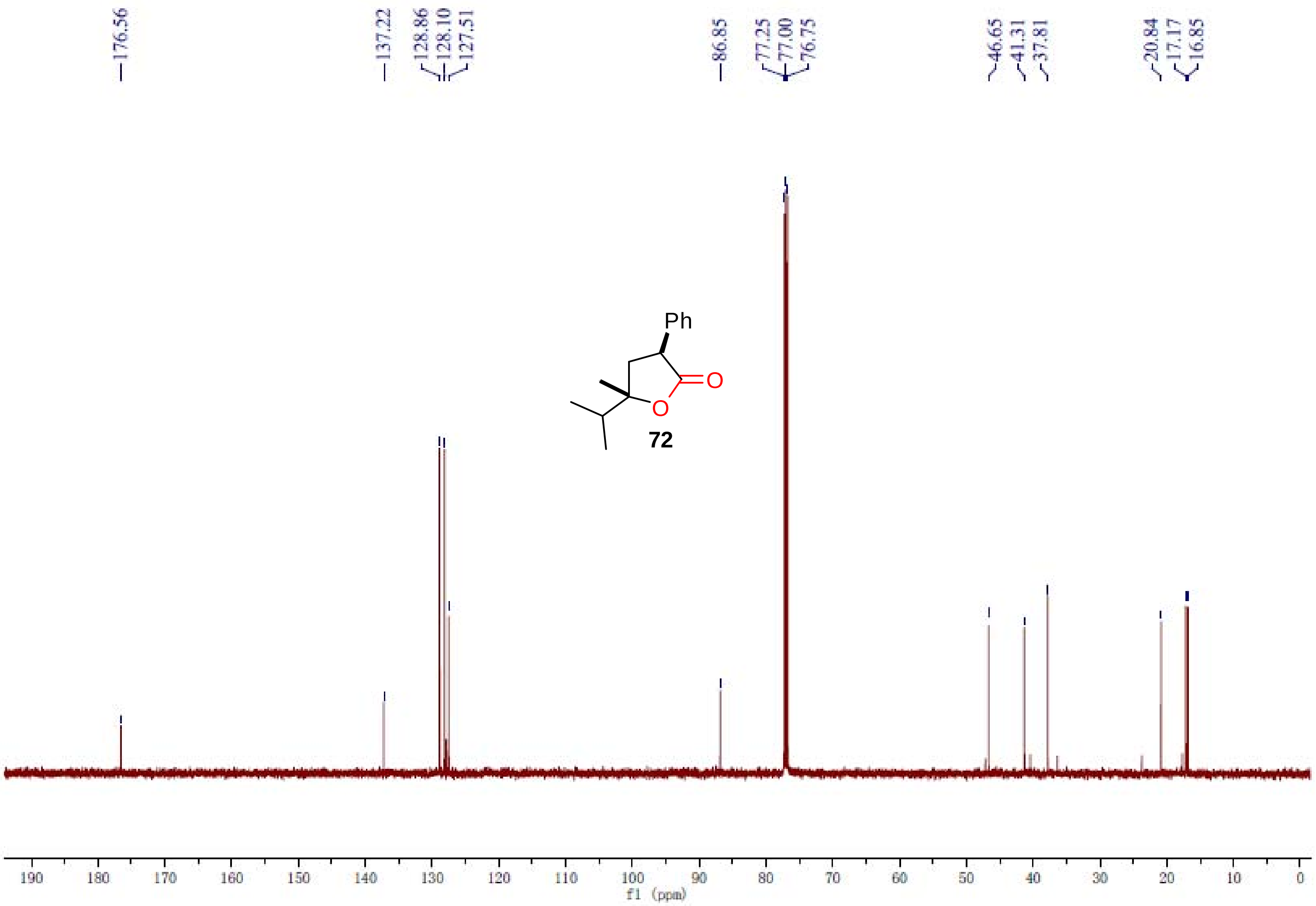
176.56
137.22
128.86
128.10
127.51
86.85
77.25
77.00
76.75
46.65
41.31
37.81
20.84
17.17
16.85
Ph
O
O
72
190 180 170 160 150 140 130 120 110 100 90 80 70 60 50 40 30 20 10 0
f1 (ppm)

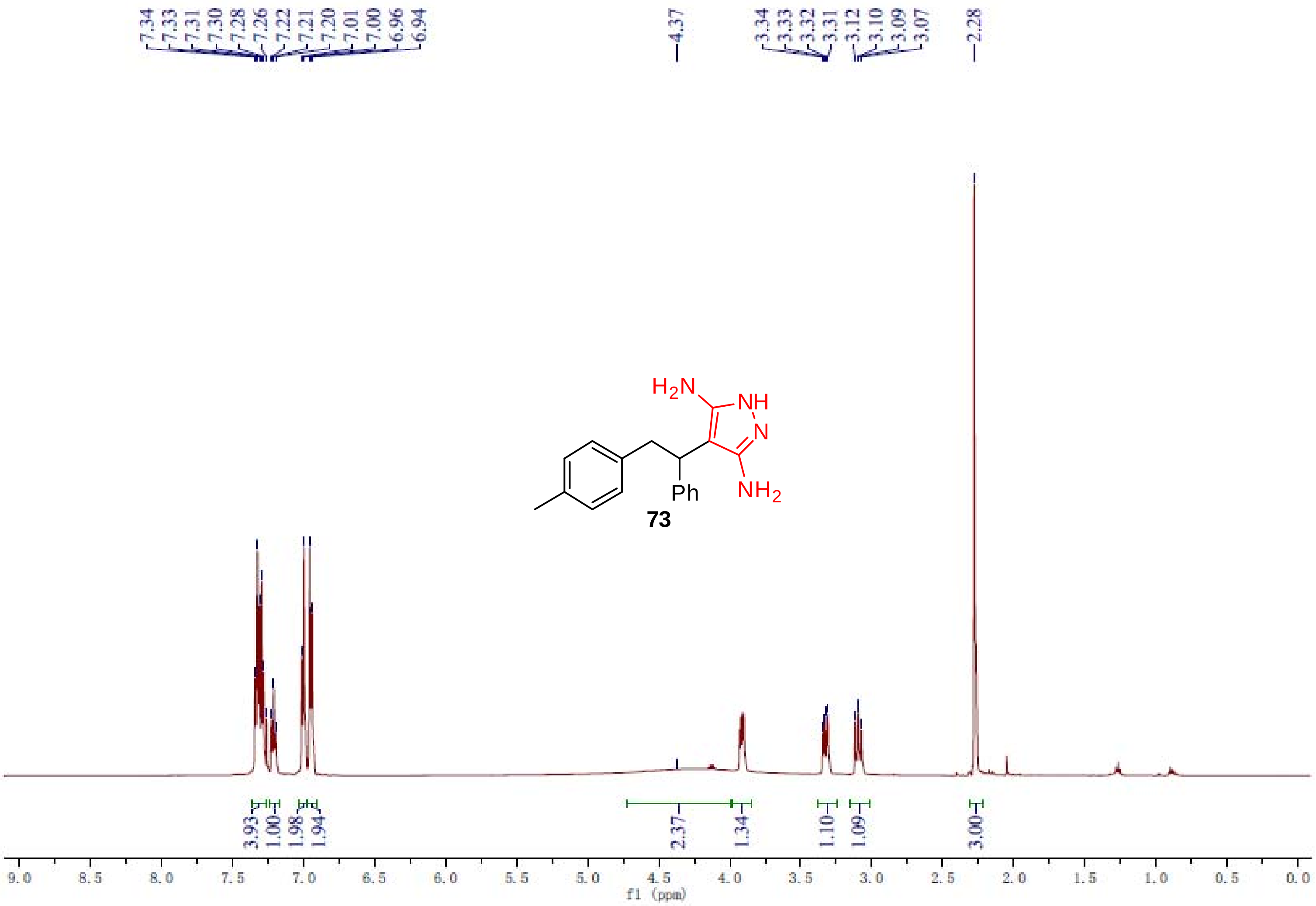

7.34
7.33
7.31
7.30
7.28
7.26
7.22
7.21
7.20
7.01
7.00
6.96
6.94
4.37
3.34
3.33
3.32
3.31
3.12
3.10
3.09
3.07
2.28
H2N
NH
N
NH2
Ph
73
3.93
1.00
1.98
1.94
2.37
1.34
1.10
1.09
3.00
9.0
8.5
8.0
7.5
7.0
6.5
6.0
5.5
5.0
4.5
4.0
3.5
3.0
2.5
2.0
1.5
1.0
0.5
0.0
f1 (ppm)

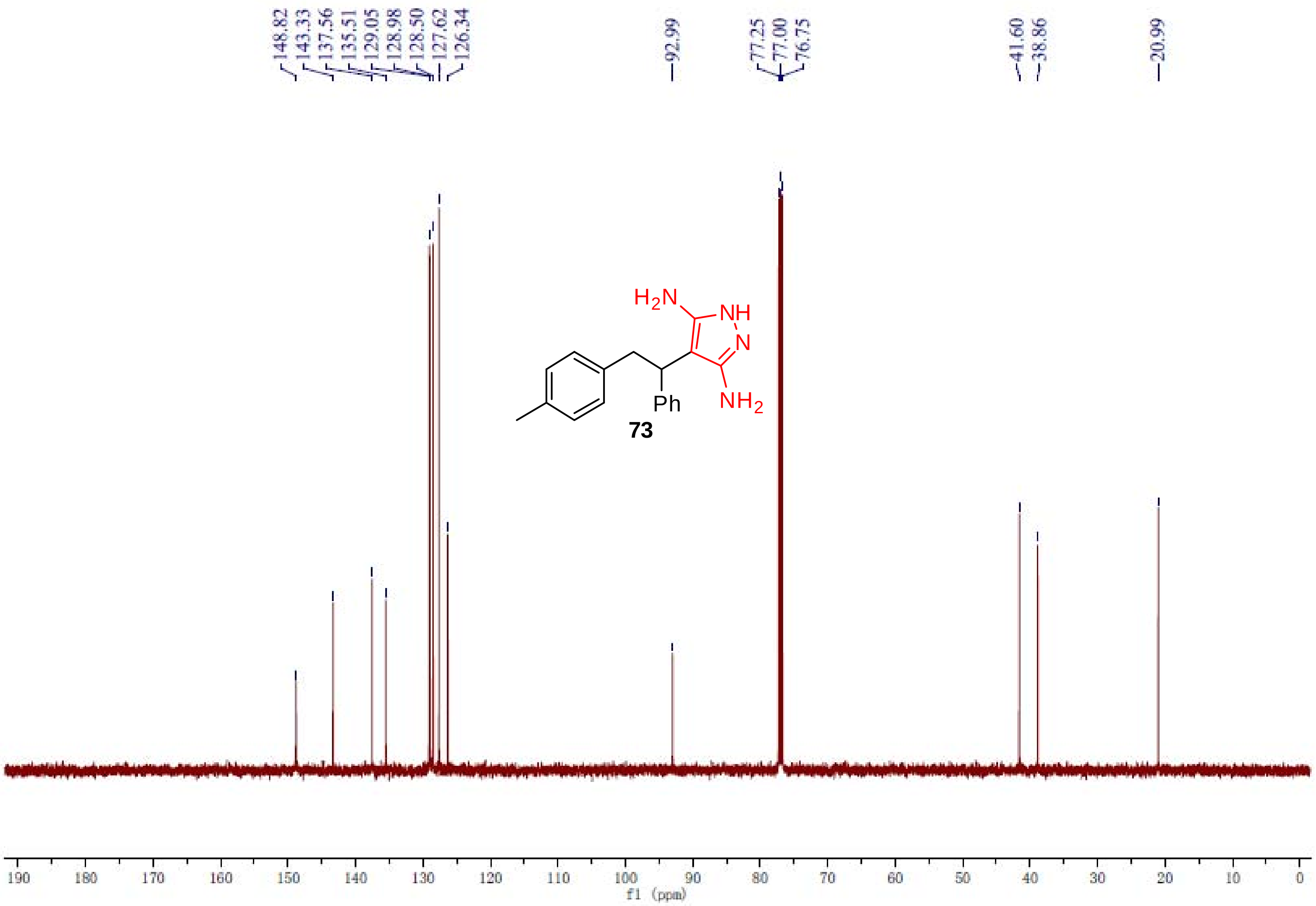
148.82
143.33
137.56
135.51
129.05
128.98
128.50
127.62
126.34
92.99
77.25
77.00
76.75
41.60
38.86
20.99
H2N
NH
N
NH2
Ph
73
f1 (ppm)

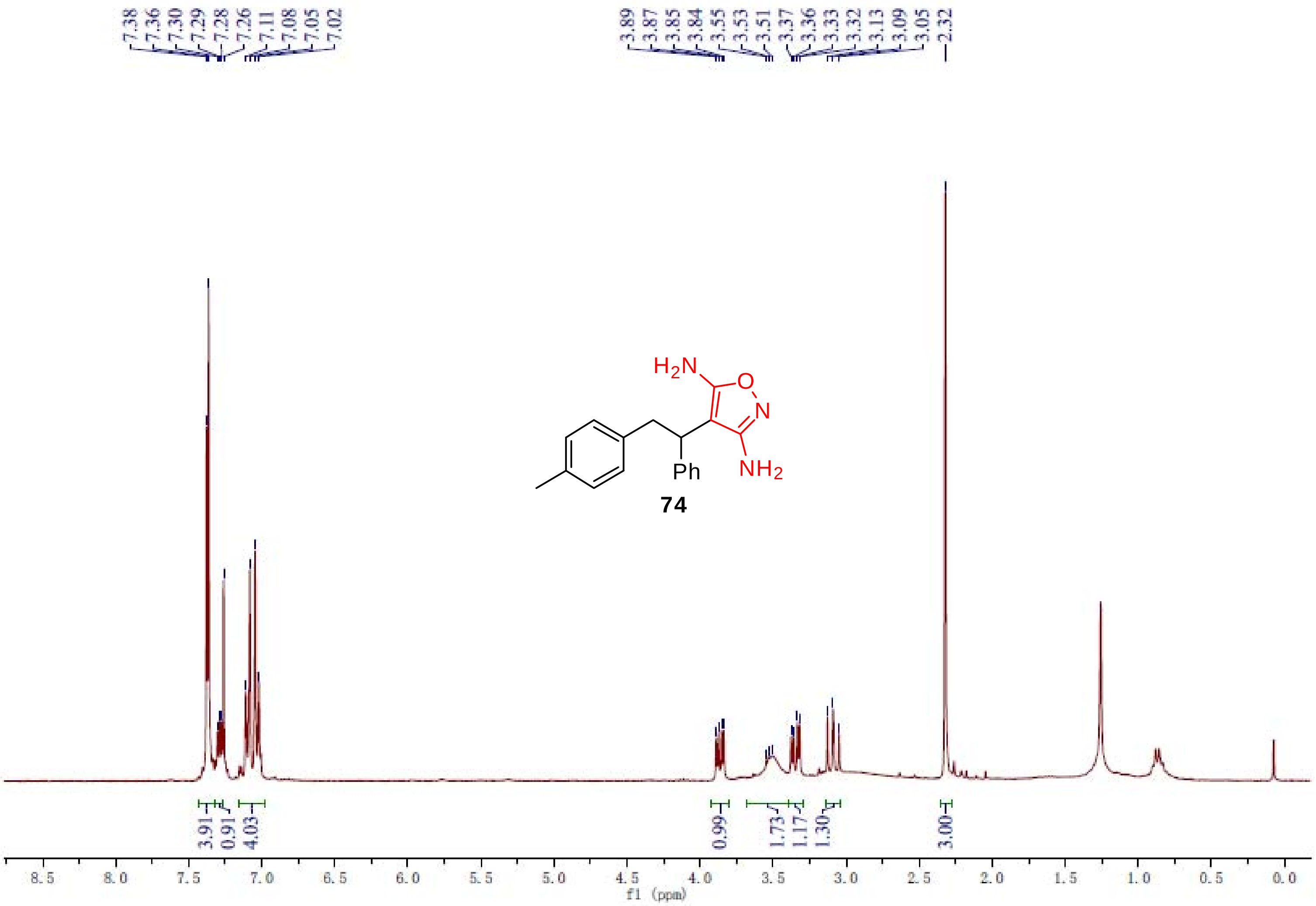

7.38
7.36
7.30
7.29
7.28
7.26
7.11
7.08
7.05
7.02
3.89
3.87
3.85
3.84
3.55
3.53
3.51
3.37
3.36
3.33
3.32
3.13
3.09
3.05
2.32
H_2N
O
N
NH_2
Ph
74
3.91
0.91
4.03
0.99
1.73
1.17
1.30
3.00
8.5
8.0
7.5
7.0
6.5
6.0
5.5
5.0
4.5
4.0
3.5
3.0
2.5
2.0
1.5
1.0
0.5
0.0
f1 (ppm)

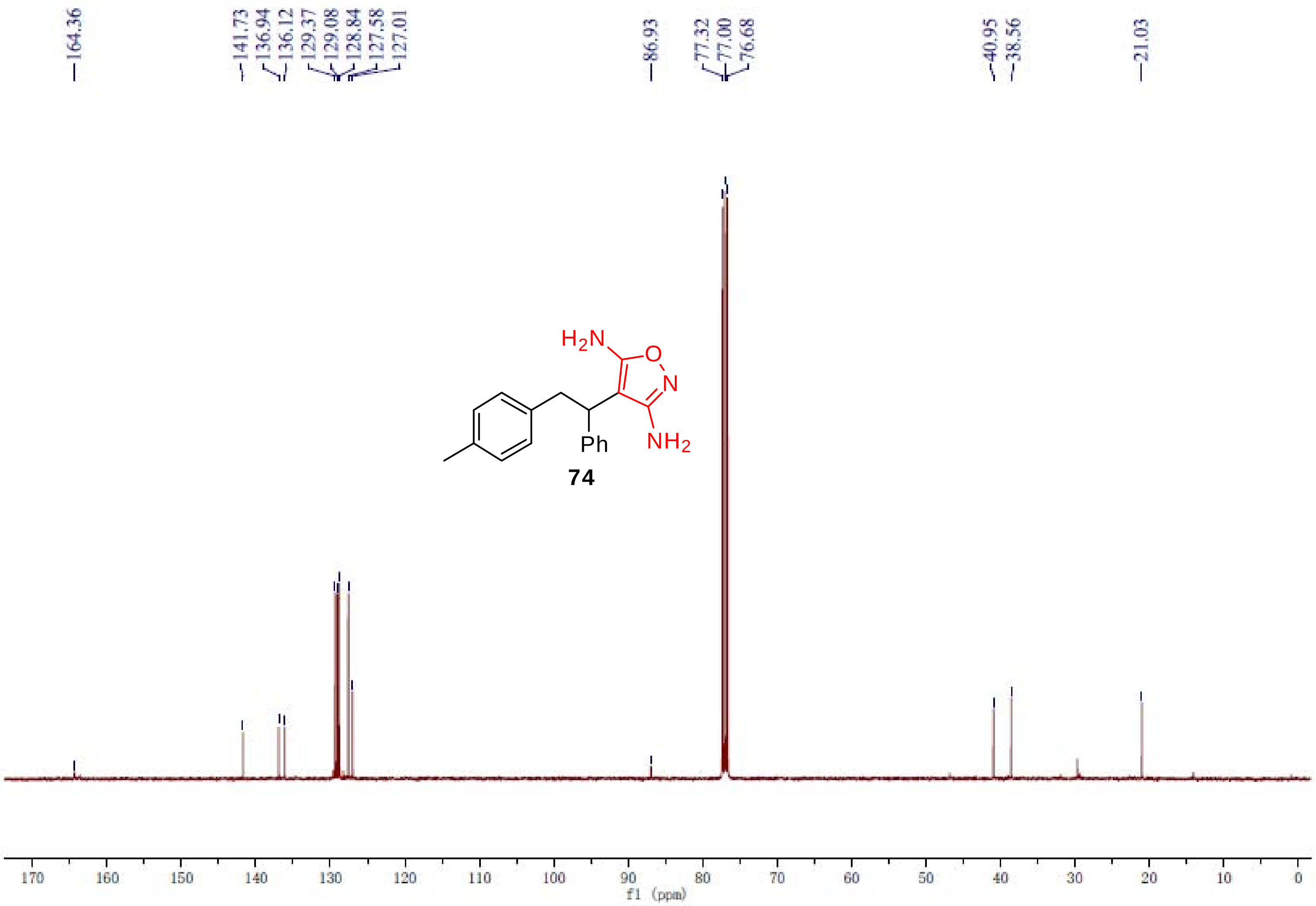
164.36
141.73
136.94
136.12
129.37
129.08
128.84
127.58
127.01
86.93
77.32
77.00
76.68
40.95
38.56
21.03
H2N
O
N
NH2
Ph
74
170 160 150 140 130 120 110 100 90 80 70 60 50 40 30 20 10 0
f1 (ppm)

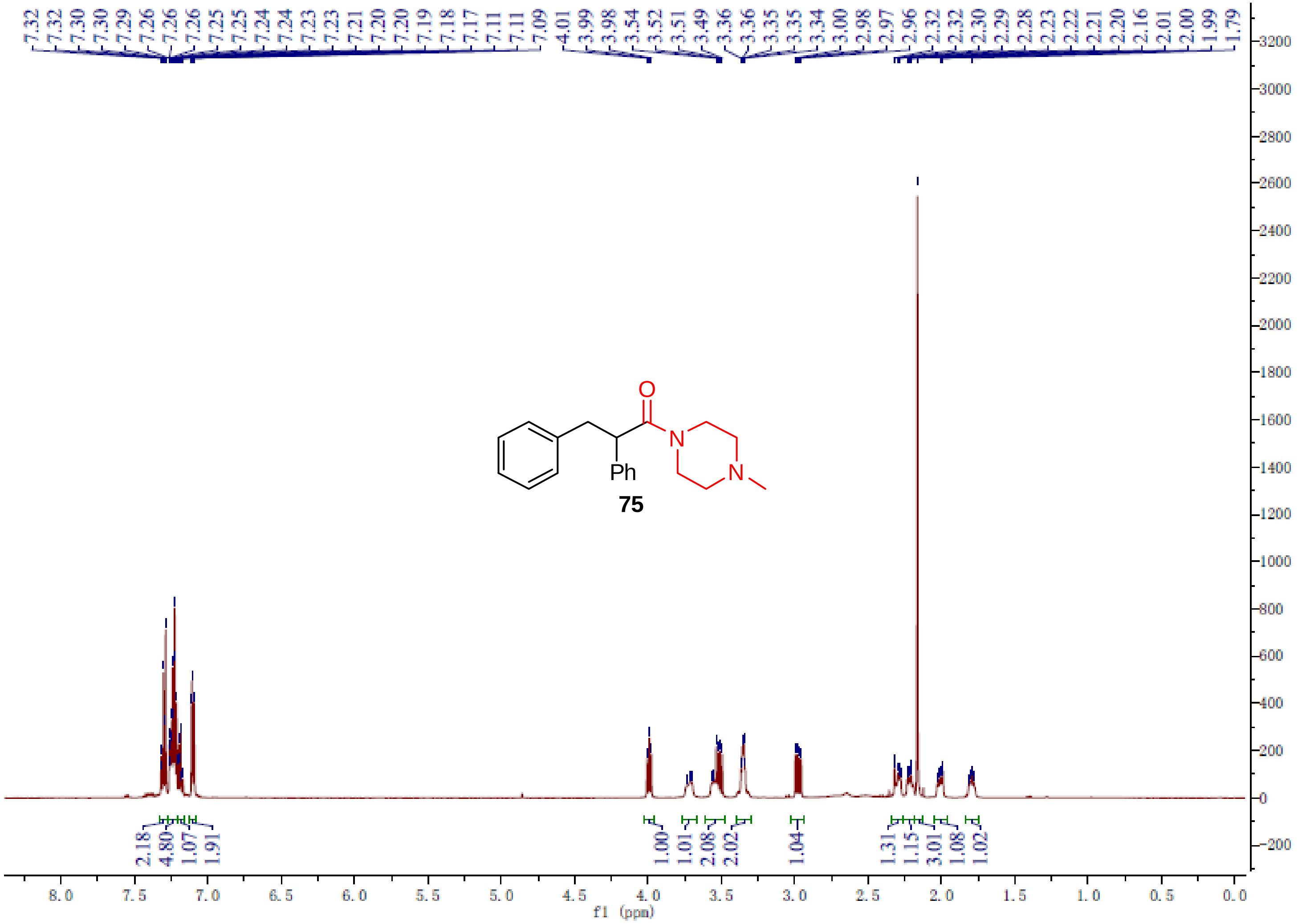
7.32 7.32 7.30 7.30 7.29 7.26 7.26 7.26 7.25 7.25 7.24 7.24 7.23 7.23 7.21 7.20 7.20 7.19 7.18 7.17 7.11 7.11 7.09 4.01 3.99 3.98 3.54 3.52 3.51 3.49 3.36 3.36 3.35 3.35 3.34 3.00 2.98 2.97 2.96 2.32 2.32 2.30 2.29 2.28 2.23 2.22 2.21 2.20 2.16 2.01 2.00 1.99 1.79
2.18 4.80 1.07 1.91 1.00 1.01 2.08 2.02 1.04 1.31 1.15 3.01 1.08 1.02
O
N
N
Ph
75
f1 (ppm)

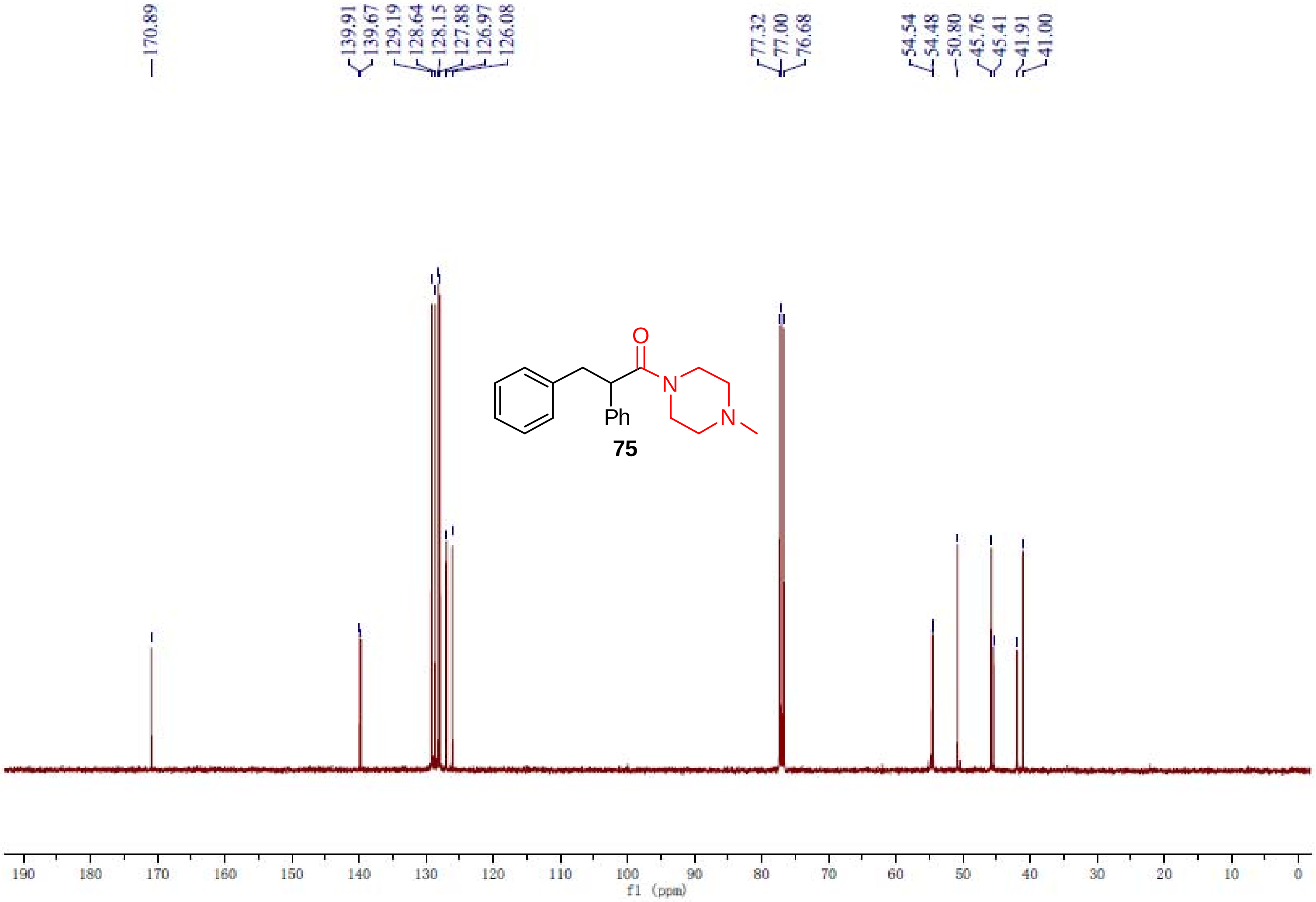
170.89
139.91
139.67
129.19
128.64
128.15
127.88
126.97
126.08
77.32
77.00
76.68
54.54
54.48
50.80
45.76
45.41
41.91
41.00
O
N
N
Ph
75
190 180 170 160 150 140 130 120 110 100 90 80 70 60 50 40 30 20 10 0
f1 (ppm)

XIV. NOESY, HMQC, and HMBC Spectra of Representative Compounds

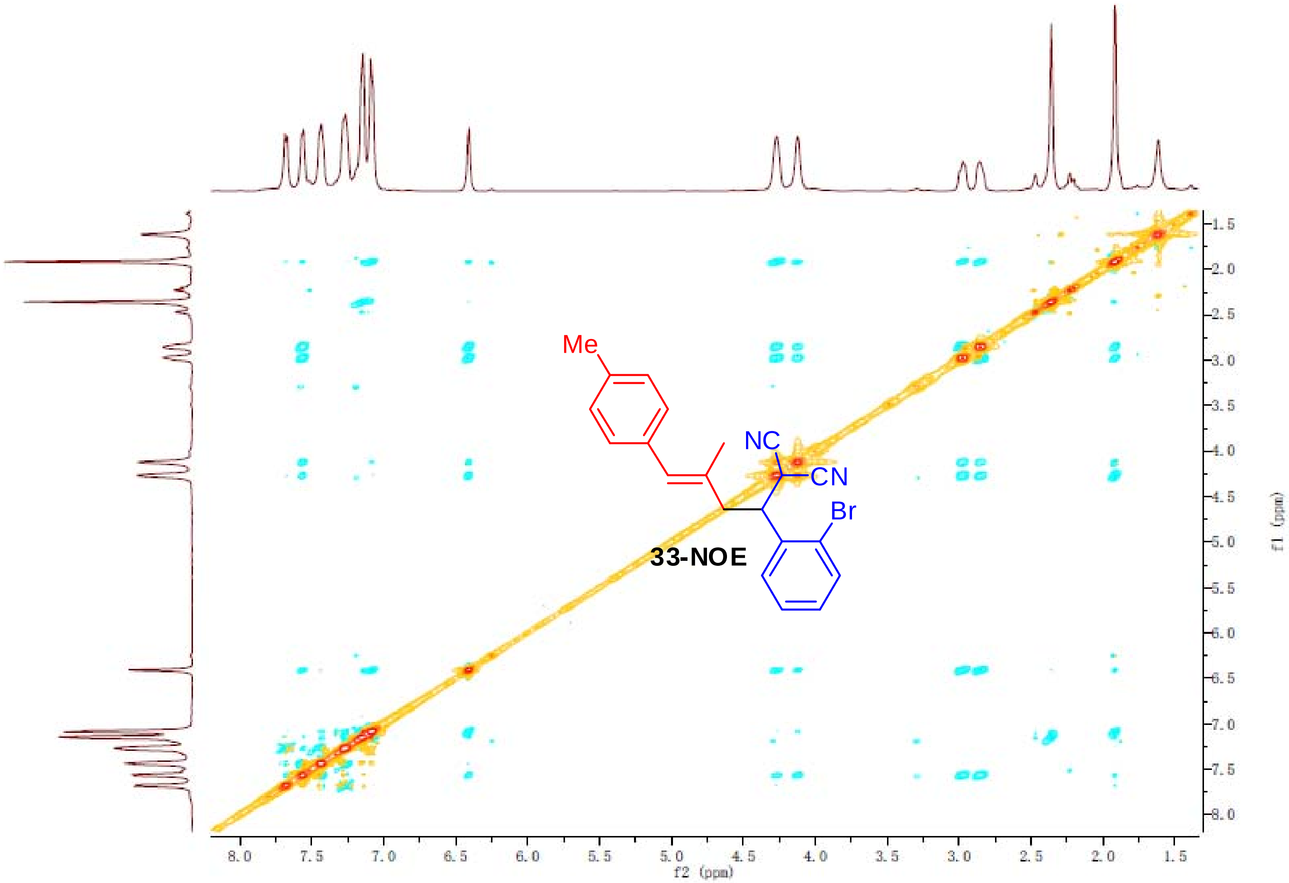

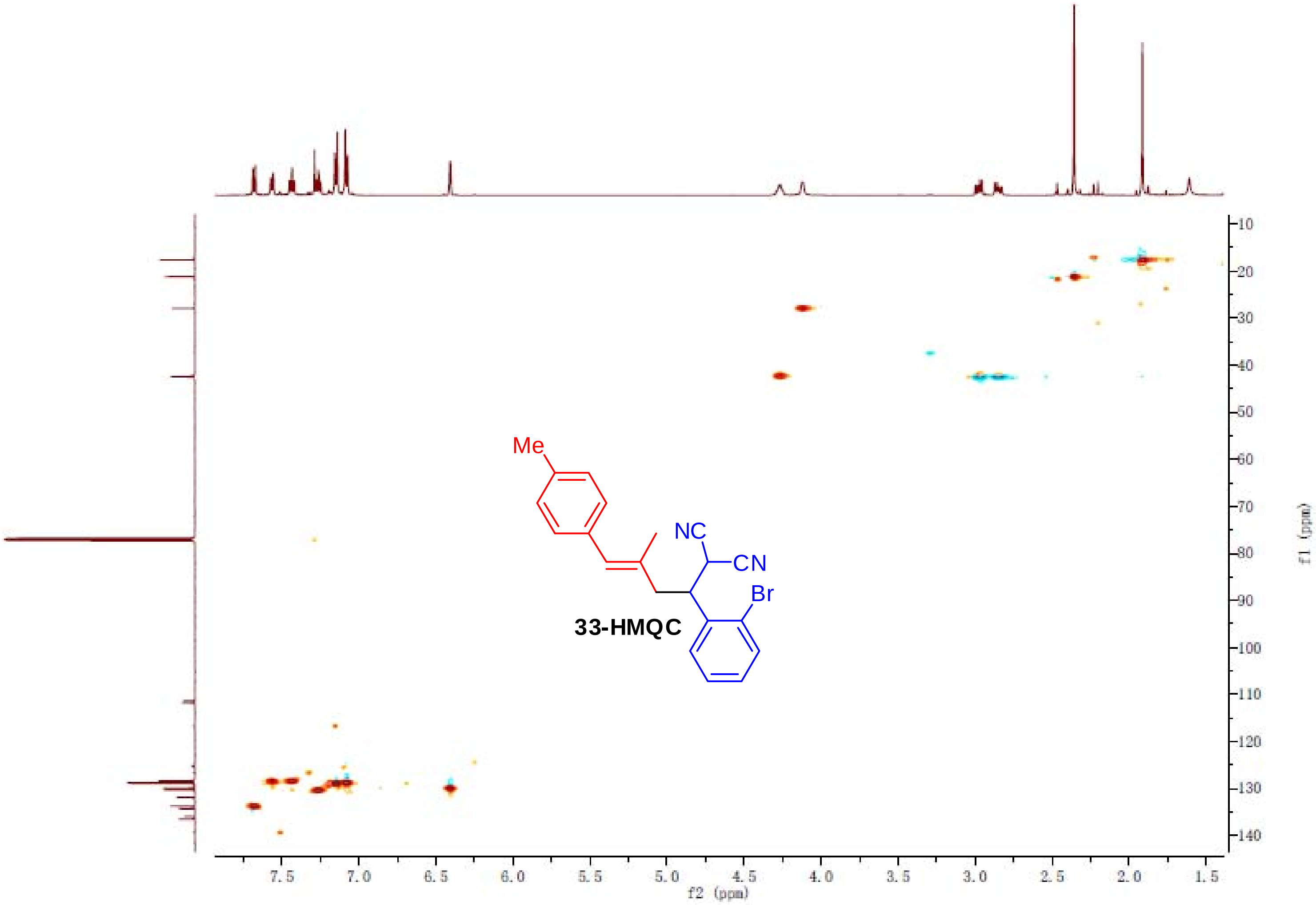
Me
NC
CN
Br
33-HMQC
f2 (ppm)
f1 (ppm)

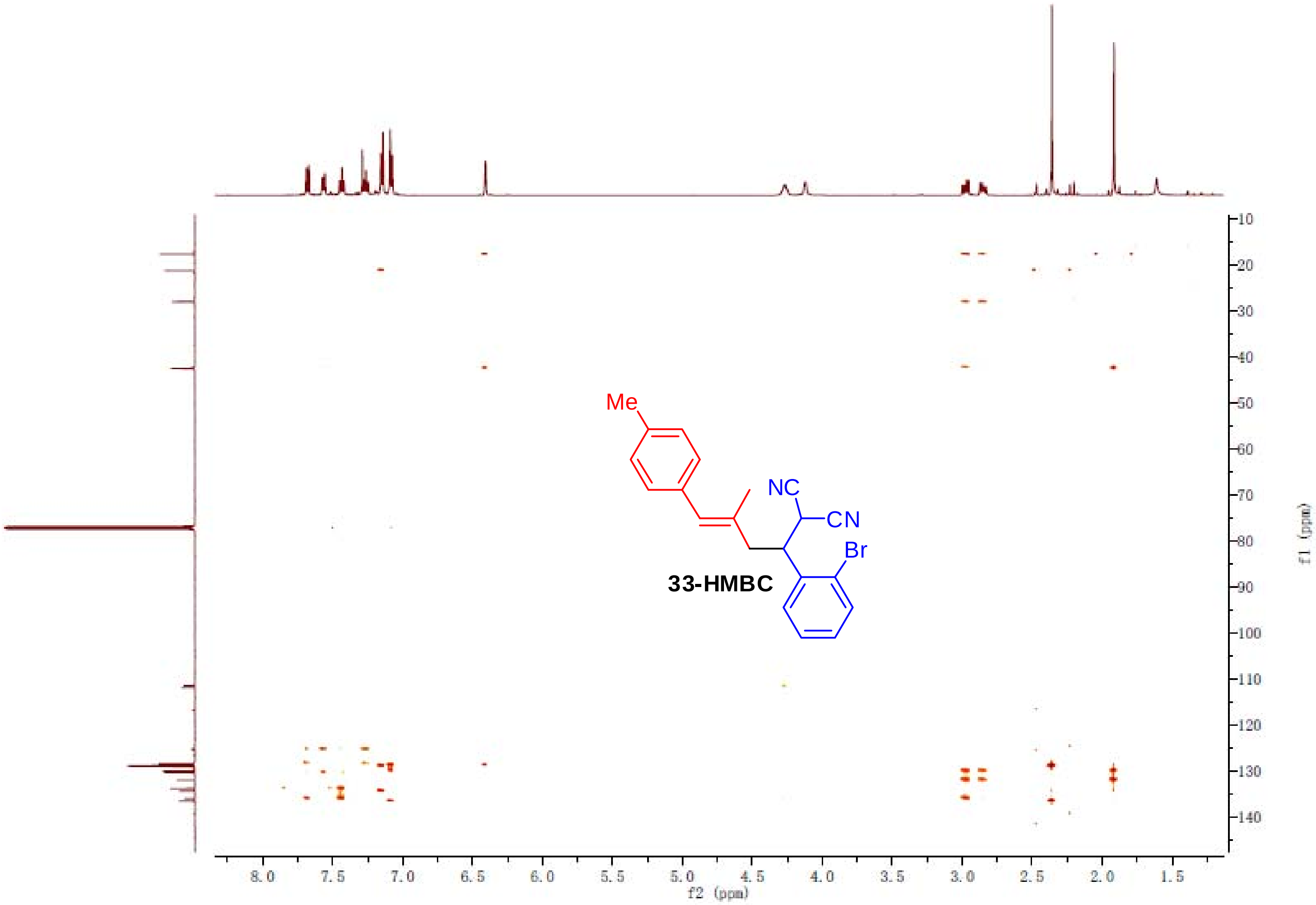
33-HMBC
Me
NC
CN
Br
f2 (ppm)
f1 (ppm)

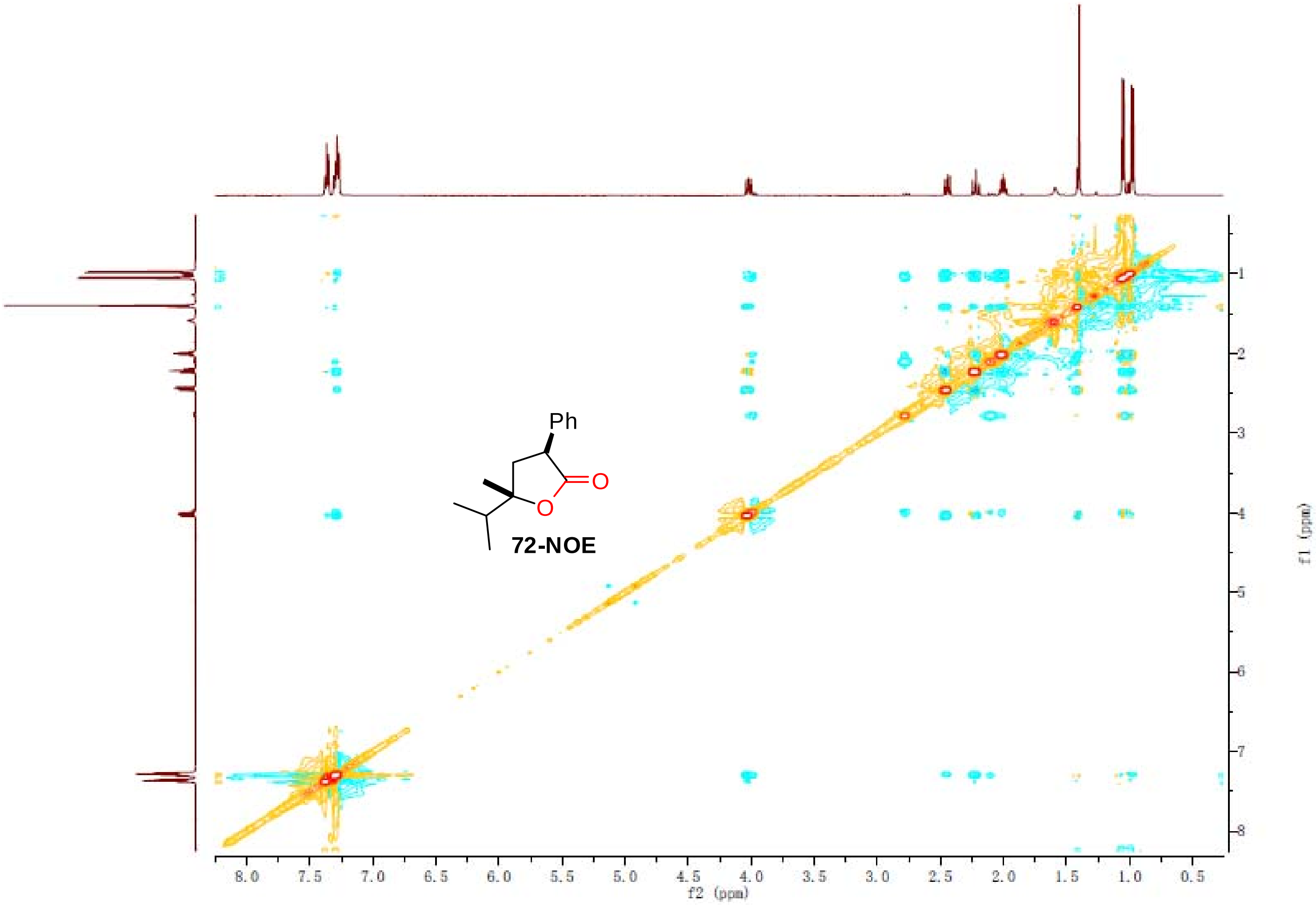
Ph
O
O
72-NOE
f1 (ppm)
f2 (ppm)
8.0
7.5
7.0
6.5
6.0
5.5
5.0
4.5
4.0
3.5
3.0
2.5
2.0
1.5
1.0
0.5
1
2
3
4
5
6
7
8
